# Mapping of a blood pressure QTL on chromosome 17 in American Indians of the strong heart family study

**DOI:** 10.1186/1471-2261-14-158

**Published:** 2014-11-11

**Authors:** Nora Franceschini, Ran Tao, Lan Liu, Sue Rutherford, Karin Haack, Laura Almasy, Harald HH Göring, Sandra Laston, Elisa T Lee, Lyle G Best, Richard Fabsitz, Shelley A Cole, Kari E North

**Affiliations:** Department of Epidemiology, University of North Carolina, Chapel Hill, NC USA; Department of Biostatistics, University of North Carolina, Chapel Hill, NC USA; Department of Biochemistry and Molecular Biology, Penn State University, Hershey, PA USA; Department of Genetics, Texas Biomedical Research Institute, San Antonio, TX USA; Center for American Indian Health Research, College of Public Health, University of Oklahoma Health Sciences Center, Oklahoma City, OK USA; Missouri Breaks Industries Research, Inc, Timber Lake, SD USA; Epidemiology and Biometry Program, National Heart, Lung, and Blood Institute, Bethesda, MD USA; Center for Human Genetics, Chapel Hill, NC USA

## Abstract

**Background:**

Blood pressure (BP) is a complex trait, with a heritability of 30 to 40%. Several genome wide associated BP loci explain only a small fraction of the phenotypic variation. Family studies can provide an important tool for gene discovery by utilizing trait and genetic transmission information among relative-pairs. We have previously described a quantitative trait locus at chromosome 17q25.3 influencing systolic BP in American Indians of the Strong Heart Family Study (SHFS). This locus has been reported to associate with variation in BP traits in family studies of Europeans, African Americans and Hispanics.

**Methods:**

To follow-up persuasive linkage findings at this locus, we performed comprehensive genotyping in the 1-LOD unit support interval region surrounding this QTL using a multi-step strategy. We first genotyped 1,334 single nucleotide polymorphisms (SNPs) in 928 individuals from families that showed evidence of linkage for BP. We then genotyped a second panel of 306 SNPs in all SHFS participants (N = 3,807) for genes that displayed the strongest evidence of association in the region, and, in a third step, included additional genotyping to better cover the genes of interest and to interrogate plausible candidate genes in the region.

**Results:**

Three genes had multiple SNPs marginally associated with systolic BP (*TBC1D16*, *HRNBP3* and *AZI1*). In *BQTN* analysis, used to estimate the posterior probability that any variant in each gene had an effect on the phenotype*, AZI1* showed the most prominent findings (posterior probability of 0.66). Importantly, upon correction for multiple testing, none of our study findings could be distinguished from chance.

**Conclusion:**

Our findings demonstrate the difficulty of follow-up studies of linkage studies for complex traits, particularly in the context of low powered studies and rare variants underlying linkage peaks.

**Electronic supplementary material:**

The online version of this article (doi:10.1186/1471-2261-14-158) contains supplementary material, which is available to authorized users.

## Background

Blood pressure (BP) is a complex trait and genetic factors account for 30 to 40% of the blood pressure variation in a population [1]. Recent progress has been made in the identification of common variants associated with BP and hypertension risk in populations [2–7], with over 50 loci for BP traits identified in genome-wide association studies (GWAS) [2–5, 8–10]. However, these findings only explain a small fraction of the phenotypic variation attributable to genetic effects [4]. Evidence for a role of rare variants in BP is well documented in monogenic forms of hypertensive and hypotensive syndromes [11, 12].

Family studies can provide an important tool for gene/loci discovery by utilizing trait and genetic transmission information among relative-pairs. Several genome scans of BP and hypertension have been published but few overlapping regions have been identified [recently reviewed in [1, 13]]. Many of these studies failed to show genome-wide significant linkage. Those demonstrating strong evidence for linkage have identified quantitative trait loci (QTLs) on chromosomes 2p [Mexican Americans [14] and families from Sardinia [15]] ,2q [African Americans [16] and Amish [17]], 3 [Finnish families [18]], 4p [Dutch families [19]], 6q [European Americans [20, 21], white Europeans [22]], 7 [African Americans [23]], 17q [European Americans [24] and Hispanics [25, 23]], 18q [Icelandic families [26] and European Americans [27]], 20 [Hispanics [23]] and 21 [European Americans [23]].

We have previously described a QTL-specific genotype-by-sex interaction for systolic BP on chromosome 17q25.3 in American Indians participants of the Strong Heart Family Study (SHFS) [28]. This is the same region identified in European Americans [24] and Hispanics [23, 25] for BP traits. This QTL became more significant when we accounted for an interaction by sex (LOD =3.4 in women in comparison to men). To follow-up persuasive linkage findings at this locus, we performed comprehensive genotyping in the chromosome 17q region using a three-stage strategy. We first genotyped a panel of single nucleotide variants (SNVs) in individuals belonging to families that showed strong evidence of linkage for systolic BP and then genotyped a second panel of SNV in all SHFS participants for the genes displaying the most prominent evidence for association in the region. As a third stage we typed additional variants in the genes of interest as well as further characterized additional candidate genes from the region. Finally, because our linkage findings were strongest in the female only sample, we examined the associations in males and females separately. Here we report the results from these analyses.

## Methods

### SHFS study design, population and phenotypes

We used data from the SHFS, a large family-based genetic component of the Strong Heart Study (SHS), a population-based study of cardiovascular disease and its risk factors in American Indians 45 years or older recruited from tribes in Arizona (AZ), Oklahoma (OK) and North and South Dakota (DK). The SHFS began as a pilot study in 1998 when ~900 members of extended families of the SHS cohort were examined. Additional family members were recruited from 2001 to 2003 for a total of 3,807 individuals in 94 multigenerational families (mean family size of 40 individuals, range 5 to 110). The SHFS protocols were approved by the Indian Health Services (IHS) Institutional Review Board, by Institutional Review Boards of all Institutions, and by the Indian tribes [29, 30]. All participants gave informed consent for genetic testing. The study was conducted according to the principles expressed in the Declaration of Helsinki.

Baseline socio-demographic, medical history, lifestyle and behaviors (smoking and alcohol intake) and medications were obtained through an interview using standardized questionnaires. Physical exams collected data on height, weight, systolic and diastolic BPs. Body mass index (BMI) was estimated using height and weight (kg/m^2^). BP was measured using a standard protocol across the three recruiting centers [30]. Brachial seated BPs were measured three times by a trained technician using a mercury column sphygmomanometer (WA Baum Co Inc, Copiague, NY) and size-adjusted cuffs. The average of the last two of the three measures was used in the analyses. Hypertension is defined by a BP of 140/90 mm Hg or higher, or use of antihypertensive drugs [31].

### Genotyping strategy, methods and quality control

The genotyping strategy is shown in Additional file 1: Figure S1.

#### Stage 1: SHFS panel 1

SNVs were selected within the 1-LOD unit drop support interval of the chromosome 17 QTL from 69,509,00 to 77,946,426 bp (genome build 35). We identified all polymorphic variants in HapMap CEU and JPN/HCB. We used linkage disequilibrium (LD) metrics (r^2^) and minor allele frequency (MAF) (SNVs with a MAF <0.001 in the HapMap data were removed) to select SNVs for genotyping. We also included 2 variants located in miRNA. A total of 1,536 SNVs were genotyped in 933 SHFS participants who are members of families showing evidence of linkage for systolic BP. Of the 1,536 SNVs on chromosome 17 that were included, 1334 SNVs were heterozygous, 18 SNVs were not polymorphic, and 184 SNVs failed genotyping. Five individuals had call rates < 95% and were removed from further analyses. Therefore, 1,334 heterozygous SNVs in 920 individuals were available for analyses.

#### Stage 2: SHFS panel 2

To provide evidence of replication for the genes on chromosome 17 that show the highest association, we genotyped 639 of the most significant SNVs of the Panel 1 analysis in all SHFS members (n = 3,800) and included additional SNVs in regions where we had relatively low coverage in Panel 1 (n = 30 SNVs). We also genotyped additional SNVs in these genes and in several other candidate genes in the region (23 SNVs in *AZI1*; 156 in *HRNB3;* 52 in *TBC1D16*; 8 in *ACTG1*; 5 in *UTS2R*; 34 in *ACE*; 33 in *SCL39A11*), for a total of 980 SNVs.

#### Stage 3: SHS cohort genotyping

We genotyped 91 of the most prominent SNVs in additional 3,516 SHS cohort members.

For the strategies described above, genotyping was performed using the multiplex Golden Gate genotyping technology from Illumina, based on allele-specific primer extension, according to the manufacturer’s protocol (Illumina, San Diego, CA). Briefly, genomic DNA (250 ng) was activated with biotin, hybridized to a pool of locus-specific oligos. PCR amplified using fluorescent-labeled primers and hybridized to the Sentrix Array Matrix, and then fluorescence intensities were analyzed using the Illumina BeadArray Reader and BeadStudio software. Cluster calls were checked for accuracy and genotypes were exported as text files for further use in association analysis. Additional samples were typed in replica as controls for genotyping and allele calling consistencies.

#### Existing genetic data in the SHFS

The SHFS has existing genotypic data on ~400 microsatellite markers [32]. MAFs were derived from pedigree founders. Mendelian inconsistencies and spurious double recombinants were detected using the SimWalk2 package [33] with the overall blanking rate for both types of errors of less than 1%. Multipoint identity-by-descent (IBD) sharing was estimated using Loki [34]. Pedigree relationships were verified using the PREST package [35]. This information was used in the implementation of the Quantitative Trait Nucleotide (*QTN*) analysis.

### Statistical analyses

We evaluated quantitative variation in systolic BP. To account for the use of anti-hypertensive medications, we added a constant to treated measures of systolic BP (10 mm Hg). Systolic BP was log-transformed due to non-normality of the data. Models also adjusted for age, sex, age^2^, and BMI and stratified by study center.

### Association analysis

We implemented a single marker test for each SNV. To evaluate the association of SNVs with BP traits among family members, we fitted linear mixed effects models to account for within pedigree correlations (implemented in Genome-Wide Association analyses with Family [GWAF]) [36]. Genotypes were tested for additive association using a 1-df Wald test. Analyses report beta and standard error (se) per copy number of the coded allele. Summary results of each center were combined using fixed effects meta-analysis. Significant p-value thresholds were determined using a Bonferroni correction (Stage 1: 1,334 SNPs, p < 3.7 × 10^−5^).

### Population stratification

The SHFS does not have ancestry informative markers to adjust for population stratification. Therefore, we tested for the evidence of population stratification for each variant using the quantitative transmission disequilibrium test (QTDT) [37] and a test for stratification described by Havill *et al.* [38], both implemented in SOLAR. To control for spurious associations due to population stratification and admixture, genotype scores are decomposed into between-family and within-family components, and the likelihood of a model in which the association parameters of these two components are estimated is compared to the likelihood of a model in which they are constrained to be equal, as expected in the absence of population stratification.

### Bayesian Quantitative Trait Nucleotide (*BQTN*) method

For genes showing the most significant associations with systolic blood pressure, we used the *BQTN* to estimate the probability that each SNV is functional [39]. The *BQTN* method is designed to separate potentially functional variants from neutral variants in LD with them based on a displacement in the observed phenotype values and it incorporates each variant one by one, evaluating the likelihood of a model in which the trait mean varies by genotype. SNVs having a LD r^2^ higher than 0.90 are treated as in one group and only one SNV in each group will be used in the analysis. Bayesian model averaging/model selection was used based on additive *QTN* effects, for which there are 2^m^ possible models, where *m* is the number of *QTN*s considered (*m* was restricted to ≤ 15 SNVs). The approach evaluates all such models and utilizes Bayesian methods to estimate the posterior probability that each SNV is functional. It then evaluates models with all possible combinations of two variants, three variants, and so on. Each model will also have the effect size estimate and its standard error for each SNV appearing in that model, the averaged effect size and standard error for that SNV in that gene and the sum of posterior model probabilities across all models containing the SNV.

## Results

**Figure 1:**
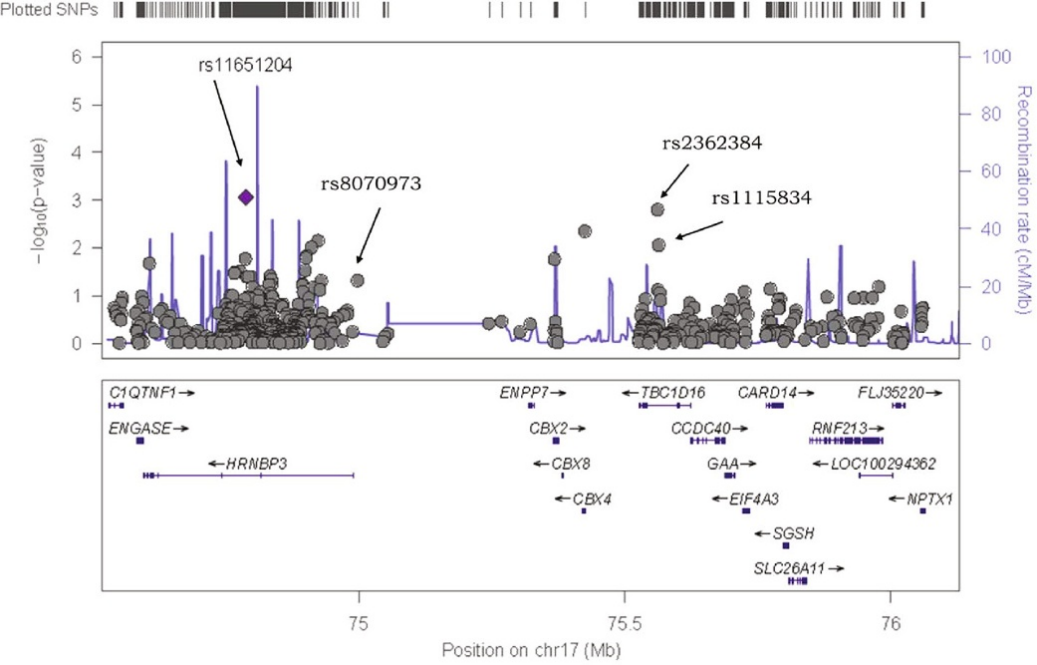
**Chromosome 17 single nucleotide variant associations among linked family members (n = 920 family members; 1,334 SNVs):** ***HRNBP3-TBC1D16*** **locus.**

**Figure 2:**
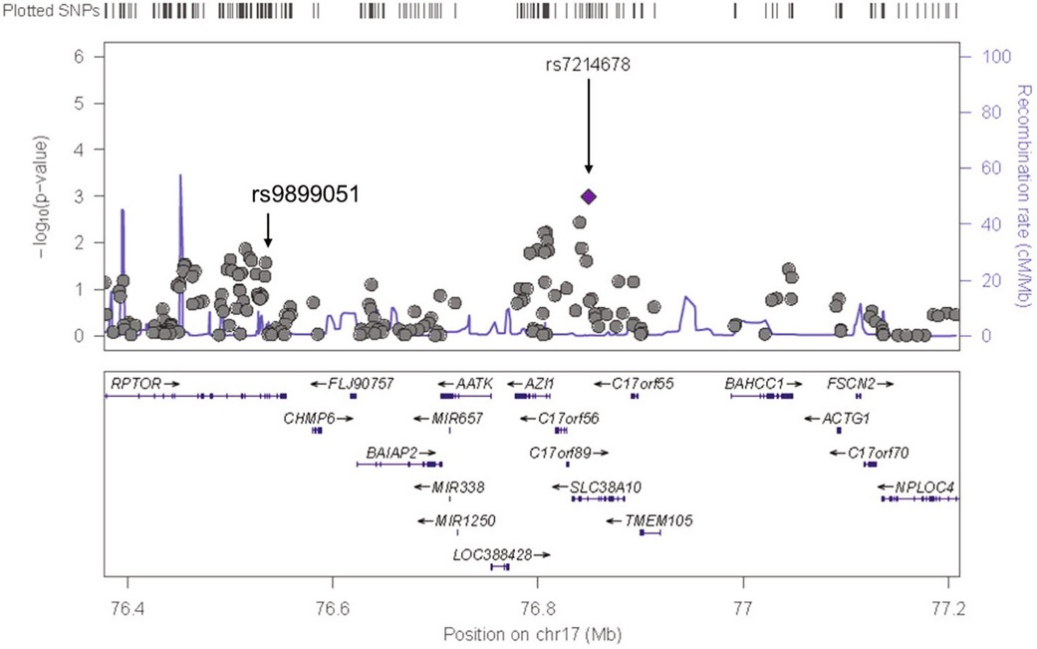
**Chromosome 17 single nucleotide variant associations among linked family members (n = 920 family members; 1,334 SNVs):** ***AZI1*** **locus.**

**Figure 3:**
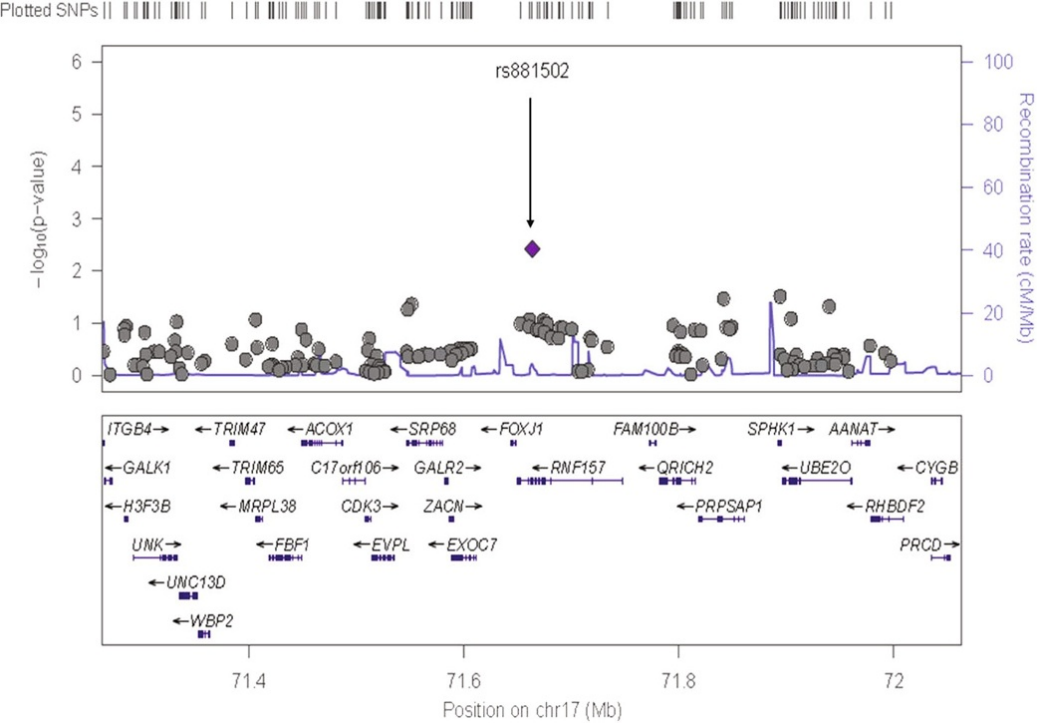
**Chromosome 17 single nucleotide variant associations among linked family members (n = 920 family members; 1,334 SNVs):** ***RNF157*** **locus.**

Descriptive characteristics of individuals genotyped in the SHFS and SHS are shown in Additional file 1: Table S1. The panel 1 sample was comprised of individuals from families with evidence of linkage for systolic BP (stage 1), and Panel 2 included all participants of the SHFS (Stage 2). Single SNV association results for Panel 1 are shown in Additional file 1: Table S2. Three genes had multiple SNVs marginally associated with systolic BP (*TBC1D16*, *HRNBP3* and *AZI1*) and low evidence for heterogeneity across centers (Figures 1, 2 and 3). There was no evidence of population stratification for these associations (p > 0.10) except for the SNP rs8070973 in the Oklahoma sample (p = 0.03 for the stratification test). However, the p-value for association of this SNP with systolic BP was 0.008 using the QTDT test, which accounts for population stratification. The pattern of LD of these genomic regions in the three centers is shown in Additional file 1: Figure S2. These three genes were prioritized for further characterization in the second genotyping panel in the entire SHFS sample. Association findings from Panel 2 for systolic BP also pointed to the same three genes (Additional file 1: Table S3). Table 1 shows the meta-analyses main results across centers for systolic BP in these Panels and in the SHS cohort study. For SNVs genotyped in both Panels, we noticed higher effect estimates in family members selected based on the linkage results (Panel 1) compared to the overall individuals genotyped in Panel 2 (Table 2). Allele frequencies in the SHFS and SHS are shown in Additional file 1: Tables S4 and S5, respectively. Association findings for females and males are shown in Additional file 1: Tables S6 and S7.

**Table 1:** **Results (p-value) of association analyses of single nucleotide variants with systolic blood pressure**

Gene	SNV	SHFS linked families (n=920)	SHFS without linked families (n=2,880)	SHS cohort (n=3,516)
*HRNBP3*	rs11651204	8.7 × 10–4	0.52	−
*HRNBP3*	rs8070973	2.4 × 10–3	7.8 × 10–3	0.71
*TBC1D16*	rs1115834	8.8 × 10–3	0.11	0.48
*TBC1D16*	rs2362384	1.6 × 10−3	0.50	0.76
*AZI1*	rs12939525	6.1 × 10−3	0.96	0.13

Analyses of natural log-transformed systolic BP, adjusted for age, age2, sex, age*sex and BMI. Meta-analyses using center-specific estimates. *Abbreviations: SNV* single nucleotide variant, *SHFS* Strong Heart Family Study, *SHS* Strong Heart Study.

**Table 2:** **Comparison of effect size and p-values from analyses using individuals selected from families showing linkage for SBP (Panel 1) and analyses using the entire SHFS (Panel 2)**

					Panel 1				Panel 2		
Gene	SNV	Effect allele	Other allele	Frequency effect allele	Beta	SE	P-value	P-value heterogeneity	Beta	SE	P-value
*TBC1D16*	rs2362384	G	A	0.05	0.0469	0.0149	0.002	0.6897	0.0065	0.0076	0.39
*AZI1*	rs12939525	G	A	0.17	−0.0231	0.0084	0.006	0.3663	−0.0063	0.0040	0.12
*AZI1*	rs9896850	G	A	0.82	0.0227	0.0083	0.006	0.336	0.0065	0.0040	0.11
*TBC1D16*	rs1115834	G	A	0.86	−0.0223	0.0085	0.009	0.3448	0.0005	0.0042	0.91
*HRNBP3*	rs11656673	G	A	0.86	−0.0239	0.0093	0.01	0.9068	−0.0082	0.0052	0.12
*AZI1*	rs9913021	G	A	0.17	−0.0204	0.0083	0.01	0.5889	−0.0062	0.0040	0.12
*AZI1*	rs9789009	G	A	0.11	−0.0248	0.0103	0.02	0.2532	−0.0134	0.0051	0.009
*BRAP*	rs11065987	G	A	0.18	−0.0228	0.0095	0.02	0.266	−0.0056	0.0046	0.22
*UTS2R*	rs11658052	G	A	0.91	0.0232	0.0099	0.02	0.3044	0.0037	0.0044	0.39
*HRNBP3*	rs11651204	G	A	0.40	−0.0133	0.0057	0.02	0.00073	−0.004	0.003	0.18
*LOC344371*	rs6711736	G	A	0.77	−0.0151	0.0067	0.02	0.5462	−0.0012	0.0033	0.72
*LOC344371*	rs9308945	G	A	0.23	0.0151	0.0067	0.02	0.5462	0.001	0.0033	0.77
*HRNBP3*	rs1563448	G	A	0.89	−0.0244	0.0113	0.03	0.4723	−0.0106	0.0063	0.09
*ATP2B1*	rs2681472	G	A	0.08	−0.0254	0.0119	0.03	0.2504	−0.0174	0.0062	0.005
*ATP2B1*	rs17249754	G	A	0.92	0.0253	0.0118	0.03	0.2626	0.0177	0.0062	0.004
*ATP2B1*	rs2681492	G	A	0.08	−0.0252	0.0118	0.03	0.263	−0.0172	0.0062	0.005
*HRNBP3*	rs4789976	G	A	0.84	0.0177	0.0083	0.03	0.9945	0.0071	0.0039	0.07
*LOC344371*	rs6729869	T	A	0.76	−0.0143	0.0067	0.03	0.5041	−0.0007	0.0033	0.84
*HRNBP3*	rs12937212	G	A	0.84	0.0176	0.0083	0.03	0.995	0.0068	0.0039	0.08
*HRNBP3*	rs12940295	C	A	0.16	−0.0182	0.0088	0.04	0.8109	−0.01	0.0043	0.02
*SLC39A11*	rs2567494	C	A	0.59	0.0121	0.0059	0.04	0.9385	−0.0012	0.003	0.69
*HRNBP3*	rs866414	C	A	0.81	0.0149	0.0075	0.05	0.3581	0.0014	0.0037	0.71
*SLC39A11*	rs7210946	G	A	0.53	−0.0116	0.0059	0.05	0.9928	0.0023	0.003	0.44
*HRNBP3*	rs10445220	C	A	0.73	0.013	0.0066	0.05	0.1092	0.0043	0.0034	0.20

SNV, single nucleotide polymorphism; het, heterogeneity; SE, standard error.

We used the *BQTN* method to estimate the posterior probability that any variant in each gene had an effect on the phenotype in Panel 1 which showed stronger association estimates (Table 3). This analysis showed marginal posterior probability for a SNV on the *AZI1* gene (>60%) but no strong probabilities of effects for any of the typed SNVs. *BQTN* analyses of Panel 2 SNVs did not reveal any SNV with a notable posterior probability of effect (data not shown).

**Table 3:** **Results from** ***BQTN*** **analyses for the panel 1 single nucleotide variants with the highest associations with systolic blood pressure**

Gene	SNV	Average	SE	Posterior probability	N SNVs eligible	N SNVs ***BQTN***
*AZI1*	rs12939525	−0.0165	0.0137	0.66	8	4
*HRNBP3*	rs8070973	−0.0099	0.0095	0.59	79	15
*TBC1D16*	rs1115834	0.0055	0.0110	0.23	12	10
*TBC1D16*	rs2362384	0.0290	0.0272	0.59	12	10
*SLC38A10*	rs7214678	−0.0146	0.0140	0.56	15	8
*RNF157*	rs12950642	0.0150	0.0102	0.77	20	8
*RNF157*	rs881502	−0.0193	0.0121	0.77	20	8
*RPTOR*	rs9899051	0.0098	0.0095	0.58	94	15

SNV, single nucleotide variant; SE, standard error; N, number.

## Discussion

To follow up persuasive evidence of genome wide linkage findings for systolic BP in American Indians, we performed a comprehensive fine mapping of a chromosome 17 genomic region. We identified evidence for locus heterogeneity in association analyses, with suggestive (nominal) associations of SNVs in three genes (*TBC1D16*, *HRNBP3* and *AZI1*) in single test analyses. Importantly, upon Bonferroni correction for multiple testing, none of these study findings can be distinguished from chance. Using the *BQTN* method to estimate the posterior probability that any SNV in each gene had an effect on the systolic BP, the *AZI1* rs12939525 SNV showed the most prominent findings (posterior probability of 0.66).

The three genes that displayed the strongest evidence for association with blood pressure have not been previously associated with blood pressure traits. *AZI1* encodes the 5-azacytidine induced 1 protein. This cell cycle protein is thought to play a role in spermatogenesis, through the recruitment of mitotic centrosome proteins and complexes. *HRNBP3* encodes hexaribonucleotide binding protein 3. This complex gene encodes 10 different mRNAs, 9 alternatively spliced variants and 1 unspliced form. Functionally, the gene has been proposed to participate in mRNA processing/splicing and functions to bind RNA and to localize in the extracellular space, cytoplasm, and nucleus [40–42]. *TBC1D16* encodes the protein TBC1 Domain Family, Member 16, which is up-regulated in melanoma. Recent studies have shown that TBC1D16 enhances the intrinsic rate of GTP hydrolysis by Rab4A, a master regulator of receptor recycling from endocytic compartments to the plasma membrane [43].

While a plausible story for a gene-phenotype relationship is often easy to make, especially given the general cell cycle functions of the identified candidate circulating proteins implicated here, no single gene nor SNV displayed a Bonferroni corrected p-value supporting statistical significance, suggesting that perhaps multiple variants of small individual effect account for the linkage evidence to chromosome 17q. Unfortunately, these findings are rather standard when viewed in the context of the many previous fine mapping studies of complex traits like blood pressure, where there has been an inherent failure to identify a single variant that accounts for a linkage peak [44, 45]. Reasons for such failures include locus heterogeneity as hypothesized here, lack of statistical power, population stratification, as well as a lack of consideration of rare variants, in particular variants with MAF < 0.01.

The sample size in these families precludes us from definitively identifying the exact set of causal genes and variants, particularly as they likely are low frequency or rare, even in this population. Novel methods are needed to map rare genes in the context of low power. These findings support the hypothesis that there are likely multiple underlying genes and/or variants and that those segregating in these pedigrees may be specific to families and not easily identified in studies of unrelated individuals. Further interrogation of these regions with sequencing to detect rare variants is warranted.

We observed differences in the patterns of SNV -BP association by sex-strata but the findings were difficult to differentiate from chance (Additional file 1: Tables S6 and S7). This is relevant because our initial linkage peak displayed evidence of sex-specific effects [28].

## Conclusions

Our results illustrate the challenges of gene discovery using association analyses in the presence of locus and allelic heterogeneity, which may have implications for the study of complex traits across ancestries. American Indian population-specific variants and low frequency/rare variants not included in the HapMap were not evaluated in this study, and could account for some of the non-significant findings. The 1000 genome project data was not available at the time of SNV selection; however the American Indian gene pool is not captured very well by the 1000 genome data. Sequencing of this region could provide further information on functional SNVs at this locus. Our results also suggest that a single genetic variant is not likely to be the cause of the linkage signal on 17q for blood pressure, which represents a major challenge for variant discovery with association analysis as power to detect effects becomes much diminished.

## Electronic supplementary material

Additional file 1: **Genotyping strategy for fine mapping of the chromosome 17 locus using family (SHFS) and cohort (SHS) data.** (PDF 4 MB)
