# Supplementary material for: Mapping of a blood pressure QTL on chromosome 17 in American Indians of the strong heart family study
Source: BMC Cardiovasc Disord. 2014 Nov 11;14:158. doi: 10.1186/1471-2261-14-158 (PMC4246441; doi:10.1186/1471-2261-14-158)
Supplement: Supplementary file 1 — Additional file 1: Genotyping strategy for fine mapping of the chromosome 17 locus using family (SHFS) and cohort (SHS) data. (PDF 4 MB) [file 12872_2014_802_MOESM1_ESM.pdf]

Supplementary Material

Figures

Figure S1. Genotyping strategy for fine mapping of the chromosome 17 locus using family (SHFS) and cohort (SHS) data

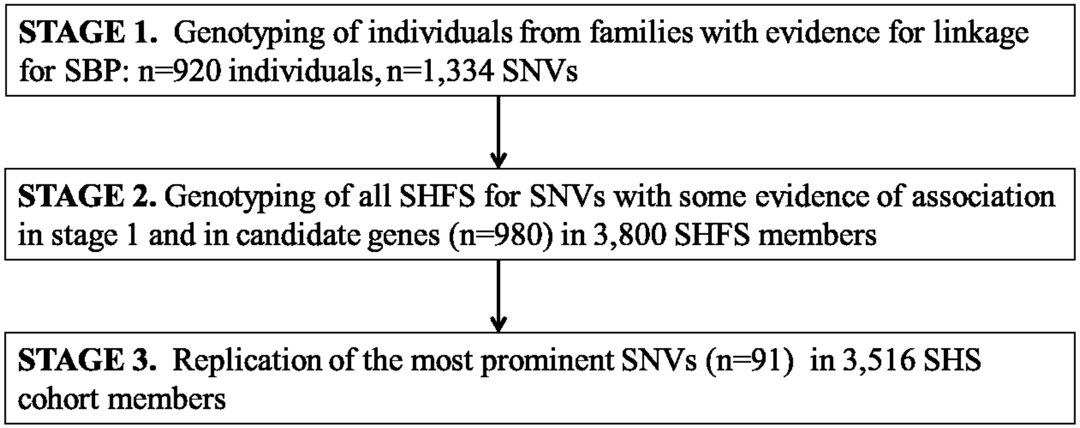

Figure S2. Linkage disequilibrium of identified gene regions by geographic region of American Indian recruitment

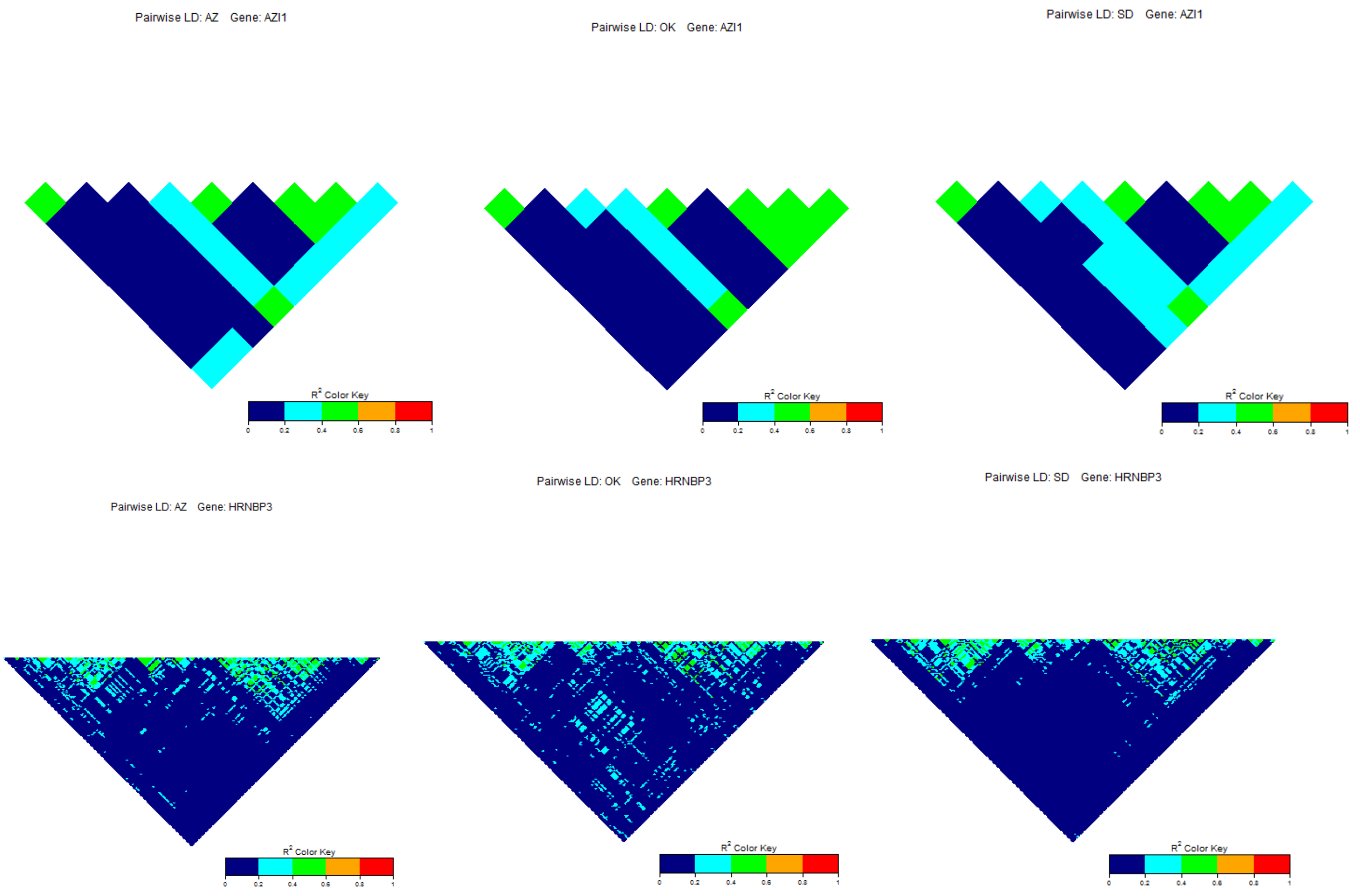

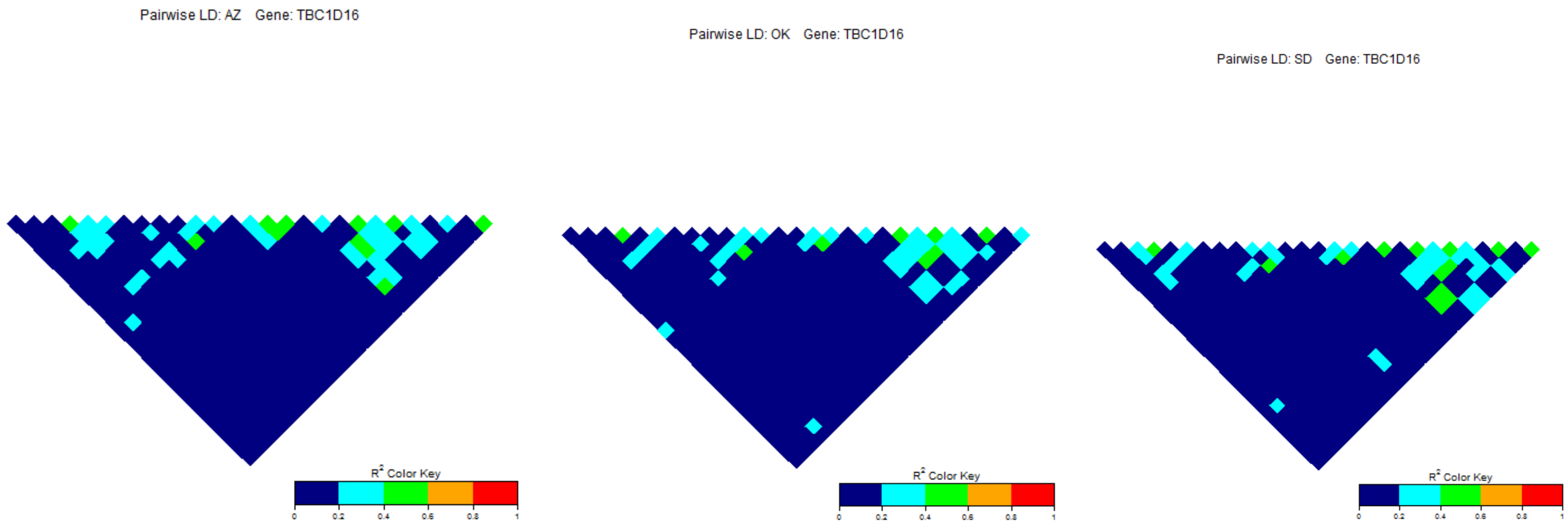

Legend: Plots show pairwise correlations (r^2) among SNVs in three genomic regions: A. the *AZ1* gene, B. the *HRNBP3* gene and C. the *TBC1D16* gene for individuals recruited from Arizona, Oklahoma and the Dakotas, respectively.

Supplementary Tables

Table S1. Descriptive characteristics of SHFS participants used in Stage 1 and Stage 2 analyses SHFS and SHS cohort replication

		SHFS Stage 1				SHFS Stage 2				SHS Replication			
Traits		CENTER				CENTER				CENTER			
		AZ	OK	SD	All	AZ	OK	SD	All	AZ	OK	SD	All
DBP	Mean	77.09	77.62	74.88	76.09	76.54	76.81	75.28	76.21	79.08	78.55	76.09	77.88
	Std	11.33	9.97	10.7	10.95	11.48	11.51	10.55	11.21	10.25	10.82	10.74	10.69
SBP	Mean	120.82	124.4	120.14	120.86	120.89	126.72	120.26	122.63	133.79	131.66	124.49	129.91
	Std	15.97	14.56	17.2	16.47	17.02	17.29	16.36	17.14	22.29	20.11	19.61	21.08
BMI	Mean	34.79	31.31	31.11	32.68	35.41	31.14	30.17	32.23	32.43	30.76	29.26	30.79
	Std	8.38	7.42	6.88	7.8	8.79	6.89	6.84	7.89	7.12	6.03	5.46	6.37
AGE	Mean	38.48	40.72	41.15	39.98	37.16	43.66	39.02	39.96	55.74	56.86	56.44	56.34
	Std	15.34	15.53	16.91	16.16	15.94	17.29	17.08	17	7.91	8.38	8.09	8.14
HTN	%	0.33	0.4	0.28	0.32	0.34	0.37	0.24	0.32	0.27	0.28	0.17	0.24
MALE	%	0.35	0.36	0.38	0.37	0.38	0.41	0.41	0.4	0.36	0.42	0.43	0.40
Number	n	387	94	439	920	1200	1207	1184	3591	1169	1132	1215	3516

Abbreviations: AZ, Arizona; BMI, body mass index; DBP, diastolic blood pressure; HTN, hypertension; OK, Oklahoma; SBP, systolic blood pressure; SD, Dakotas; Std, standard deviation

Table S2. Main meta-analysis findings of association for SBP in Panel 1

SNP	Coded allele	Other allele	Coded allele frequency	beta	se	P	Direction	Phet	Position	Present in Panel 1&2	Gene symbol
rs581157	a	c	0.35	0.0127	0.006	0.035	-++	0.139	72613589	0	*CD300E*
rs8081669	a	g	0.66	-0.0127	0.006	0.034	+--	0.134	72614611	0	*CD300E*
rs12950039	a	g	0.65	-0.0137	0.006	0.023	---	0.189	72615293	0	*CD300E*
rs1699607	a	g	0.64	-0.0124	0.006	0.039	---	0.088	72619225	0	*CD300E*
rs8082005	a	g	0.63	0.0125	0.0062	0.044	+++	0.987	73113141	0	*ARMC7*
rs7502835	a	g	0.66	0.0167	0.0063	0.0081	+++	0.683	73498623	0	*CASKIN2*
rs1671033	c	g	0.3	-0.0135	0.0063	0.033	+--	0.117	73554523	0	*LLGL2*
rs820201	a	g	0.6	-0.0117	0.0059	0.049	-+-	0.242	73634687	0	*LOC643008*
rs820202	a	g	0.6	-0.0117	0.0059	0.048	-+-	0.241	73636623	0	*LOC643008*
rs820210	a	g	0.41	0.0119	0.0059	0.043	+-+	0.233	73650495	0	*RECQL5*
rs820155	c	g	0.6	-0.0128	0.0059	0.03	-+-	0.171	73663778	0	*SAP30BP*
rs820129	a	g	0.4	0.0129	0.0059	0.028	+-+	0.209	73668107	0	*SAP30BP*
rs707707	a	g	0.6	-0.0131	0.0059	0.026	-+-	0.204	73669521	0	*SAP30BP*
rs2305345	a	c	0.75	-0.0134	0.0066	0.044	-+-	0.149	74040293	0	*SRP68*
rs881502	a	g	0.39	-0.0175	0.006	0.0038	---	0.559	74152366	0	*RNF157*
rs385689	a	g	0.8	-0.0171	0.0081	0.034	-+-	0.672	74330638	0	*PRPSAP1*
rs3744037	a	g	0.24	0.015	0.0069	0.031	+++	0.212	74383475	0	*SPHK1*

rs9903640	c	g	0.69	-0.0126	0.0064	0.048	---	0.278	74429003	0	*UBE2O*
rs8067984	a	g	0.73	-0.015	0.0066	0.024	--+	0.006	74942478	0	*MGAT5B*
rs1465983	a	g	0.73	-0.0153	0.0066	0.021	--+	0.006	74942809	0	*MGAT5B*
rs11077879	a	g	0.73	-0.0148	0.0066	0.026	--+	0.006	74943446	0	*MGAT5B*
rs8074685	a	g	0.41	-0.0133	0.006	0.026	---	0.035	74946286	0	*MGAT5B*
rs11657098	a	g	0.77	0.0137	0.0069	0.048	+-+	0.209	76549899	0	
rs8065523	a	g	0.94	0.0298	0.0128	0.02	+++	0.903	76555010	0	
rs11651201	a	g	0.4	-0.0138	0.0057	0.016	--+	1E-03	77276463	0	*HRNBP3*
rs9895586	c	g	0.54	0.0118	0.0058	0.043	+++	0.752	77325470	0	*HRNBP3*
rs8070973	a	c	0.52	0.0176	0.0057	0.0022	+++	0.741	77381431	0	*HRNBP3*
rs12601898	a	g	0.82	0.0207	0.0087	0.018	+++	0.664	77389101	0	*HRNBP3*
rs7226158	a	g	0.78	0.0179	0.0077	0.02	+++	0.392	77390725	0	*HRNBP3*
rs7225663	a	c	0.25	-0.017	0.0075	0.022	-+-	0.261	77391145	0	*HRNBP3*
rs884652	a	c	0.88	-0.0204	0.0091	0.025	--+	0.101	77413429	0	*HRNBP3*
rs3751955	a	c	0.52	-0.0139	0.0058	0.016	---	0.608	77754073	0	*CBX2*
rs7217395	a	g	0.63	0.014	0.0059	0.017	+++	0.554	77754569	0	*CBX2*
rs2289728	a	g	0.77	0.0196	0.0069	0.0045	+++	0.406	77811854	0	*CBX4*
rs2362384	a	g	0.05	0.0469	0.0149	0.0016	+++	0.691	77947690	1	*TBC1D16*
rs1115834	a	g	0.87	-0.0232	0.0085	0.0064	---	0.381	77950014	1	*TBC1D16*
rs11150738	a	g	0.08	0.0211	0.0105	0.045	+++	0.961	78648127	0	*KIAA1303*

rs11651587	a	g	0.27	0.0139	0.0068	0.04	+-+	0.514	78840250	0	*KIAA1303*
rs7210951	a	t	0.28	0.0144	0.0067	0.031	+++	0.755	78840944	0	*KIAA1303*
rs9912092	a	g	0.72	-0.0147	0.0067	0.029	-+-	0.552	78841693	0	*KIAA1303*
rs7222366	a	g	0.28	0.0139	0.0068	0.041	+++	0.718	78851634	0	*KIAA1303*
rs1468030	a	g	0.22	0.0143	0.0069	0.037	+++	0.919	78882840	0	*KIAA1303*
rs4969301	a	g	0.62	-0.014	0.0062	0.023	-+-	0.117	78885904	0	*KIAA1303*
rs1012117	a	g	0.78	-0.0142	0.0069	0.039	---	0.913	78887602	0	*KIAA1303*
rs7215994	a	g	0.23	0.0133	0.0068	0.049	+++	0.873	78893671	0	*KIAA1303*
rs11657655	a	g	0.77	-0.0136	0.0068	0.044	---	0.884	78895788	0	*KIAA1303*
rs2271602	a	g	0.77	-0.0136	0.0068	0.044	---	0.884	78896488	0	*KIAA1303*
rs3817292	c	g	0.77	-0.0136	0.0068	0.044	---	0.884	78897056	0	*KIAA1303*
rs4969227	a	g	0.74	0.0164	0.0066	0.014	+-+	0.072	78900598	0	*KIAA1303*
rs9899051	a	g	0.64	-0.0139	0.006	0.021	-+-	0.174	78904680	0	*KIAA1303*
rs4969311	c	g	0.36	0.0136	0.006	0.024	+-+	0.184	78906360	0	*KIAA1303*
rs1877926	a	g	0.77	-0.0134	0.0068	0.047	---	0.902	78911041	0	*KIAA1303*
rs7503779	a	g	0.67	-0.0141	0.0064	0.027	---	0.496	78920172	0	*KIAA1303*
rs8078643	a	g	0.45	0.0127	0.0059	0.031	+++	0.804	78922683	0	*KIAA1303*
rs1468035	a	g	0.45	0.0121	0.0059	0.039	+++	0.882	78923953	0	*KIAA1303*
rs7218122	a	g	0.44	0.0115	0.0058	0.05	+++	0.93	78924882	0	*KIAA1303*
rs2659030	a	g	0.24	-0.0167	0.0072	0.021	---	0.944	79177974	0	*AZI1*

rs9896850	a	g	0.83	0.0228	0.0083	0.0062	-++	0.336	79191726	1	*AZI1*
rs12939525	a	g	0.17	-0.0231	0.0084	0.006	+--	0.366	79194005	1	*AZI1*
rs8073077	a	g	0.82	0.0218	0.0084	0.0097	-++	0.423	79195021	0	*AZI1*
rs969413	a	t	0.76	0.0179	0.0073	0.015	+++	0.755	79195814	0	*AZI1*
rs3803767	a	g	0.95	0.0451	0.0156	0.0037	-++	9E-04	79226362	0	*MGC15523*
rs9892257	a	c	0.93	0.0304	0.0123	0.013	-++	0.219	79228183	0	*MGC15523*
rs9901361	a	g	0.83	0.0177	0.0079	0.025	-++	0.09	79232941	0	*MGC15523*
rs7214678	a	c	0.22	-0.0227	0.0069	0.001	+--	4E-04	79234588	0	*MGC15523*
rs8746	a	g	0.88	-0.0192	0.0092	0.037	---	0.992	79429852	0	*BAHCC1*
rs4789763	a	g	0.37	-0.0123	0.0061	0.045	---	0.08	80289284	0	*SECTM1*

Abbreviations: Phet, P for heterogeneity of effects in meta-analysis across centers; SNP, single nucleotide polymorphism. SNPs are ordered by chromosome position.

Table S3. Main meta-analysis findings of association for SBP in Panel 2

SNP	Coded allele	Other allele	Coded allele frequency	beta	se	P	Direction	Phet	Position	Present in Panel 1&2	Gene symbol
rs8073197	a	g	0.07	-0.0113	0.0056	0.04567	---	0.40	70763353	0	*SLC39A11*
rs2411131	a	g	0.68	0.0071	0.0032	0.02598	+++	0.42	75577377	0	*LOC728414*
rs9898803	a	g	0.90	-0.0115	0.0049	0.01933	---	0.78	75656898	0	*LOC728414*
rs7209618	a	c	0.44	-0.0074	0.0029	0.01246	---	0.64	77278953	1	*HRNBP3/RBFOX3*
rs7222861	a	c	0.56	0.0068	0.0029	0.02159	+++	0.41	77283217	0	*HRNBP3*
rs9905852	a	g	0.51	0.0060	0.0029	0.03955	+++	0.42	77286825	0	*HRNBP3*
rs11308563	a	g	0.57	0.0059	0.0030	0.04645	+++	0.25	77295234	0	*HRNBP3*
rs12940295	a	c	0.16	-0.0100	0.0043	0.02076	---	0.26	77323221	0	*HRNBP3*
rs4790024	a	g	0.37	-0.0064	0.0032	0.04223	---	0.87	77380037	0	*HRNBP3*
rs12453207	a	g	0.77	-0.0076	0.0035	0.02936	---	0.59	77383748	0	*HRNBP3*
rs1983283	a	g	0.99	-0.0513	0.0238	0.03115	?-?	1.00	77415086	0	*HRNBP3*
rs7221608	c	g	0.12	-0.0102	0.0046	0.025	---	0.85	77928017	0	*TBC1D16*
rs7211532	a	g	0.42	-0.0065	0.0030	0.03211	---	0.92	77931491	0	*TBC1D16*
rs1631707	a	g	0.85	0.0089	0.0045	0.04544	+++	0.66	77945878	0	*TBC1D16*
rs34768269	a	g	0.09	-0.0114	0.0050	0.02267	+--	0.16	77952751	0	*TBC1D16*
rs9789009	a	g	0.10	-0.0134	0.0051	0.00867	---	0.73	79192304	0	*AZI1*

Abbreviations: Phet, P for heterogeneity of effects in meta-analysis across centers; SNP, single nucleotide polymorphism. SNPs are ordered by chromosome position.

Table S4. Allele frequencies in the Strong Heart Family Study

SNP	AZ			SD			OK		
	Minor allele	Major allele	Minor allele frequency	Minor allele	Major allele	Minor allele frequency	Minor allele	Major allele	Minor allele frequency
rs1004467	G	A	0.224	G	A	0.187	G	A	0.200
rs1007464	A	G	0.494	G	A	0.429	A	G	0.500
rs1028060	G	A	0.164	G	A	0.247	G	A	0.217
rs10445219	G	C	0.087	G	C	0.119	G	C	0.166
rs10445220	A	C	0.285	A	C	0.196	A	C	0.235
rs10491334	A	G	0.046	A	G	0.108	A	G	0.087
rs10493340	G	A	0.385	G	A	0.332	G	A	0.248
rs10495809	A	G	0.140	A	G	0.225	A	G	0.226
rs1055086	A	G	0.301	A	G	0.304	A	G	0.289
rs10775361	A	C	0.086	A	C	0.090	A	C	0.114
rs1077693	A	G	0.156	A	G	0.269	A	G	0.257
rs11014166	A	T	0.048	A	T	0.141	A	T	0.128
rs11024074	G	A	0.423	G	A	0.256	G	A	0.304
rs1106221	G	C	0.006	G	C	0.035	G	C	0.025
rs1106281	G	A	0.037	G	A	0.146	G	A	0.115
rs11065987	G	A	0.046	G	A	0.174	G	A	0.166
rs11077416	A	G	0.303	A	G	0.256	A	G	0.327
rs11077420	A	C	0.148	A	C	0.256	A	C	0.222
rs11077421	A	T	0.372	T	A	0.449	A	T	0.458
rs11077435	G	A	0.017	G	A	0.048	G	A	0.051
rs11077628	A	G	0.042	A	G	0.055	A	G	0.054
rs11077637	A	G	0.488	G	A	0.430	G	A	0.428

rs11077919	G	A	0.375	G	A	0.198	G	A	0.300
rs11077938	G	A	0.325	G	A	0.196	G	A	0.240
rs11150821	A	C	0.366	A	C	0.381	A	C	0.256
rs11150823	A	G	0.011	A	G	0.022	A	G	0.053
rs1115834	A	G	0.100	A	G	0.176	A	G	0.168
rs11191548	G	A	0.224	G	A	0.187	G	A	0.199
rs1124736	A	C	0.115	A	C	0.163	A	C	0.248
rs11308563	A	G	0.360	G	A	0.485	A	G	0.455
rs11646213	A	T	0.351	A	T	0.385	A	T	0.357
rs11650154	G	A	0.303	G	A	0.256	G	A	0.329
rs11650345	A	C	0.015	A	C	0.070	A	C	0.072
rs11650709	A	G	0.462	G	A	0.277	G	A	0.411
rs11651204	G	A	0.391	G	A	0.452	G	A	0.347
rs11651302	G	A	0.017	G	A	0.073	G	A	0.090
rs11651611	G	A	0.015	G	A	0.071	G	A	0.074
rs11651690	G	A	0.015	G	A	0.071	G	A	0.074
rs11653700	A	G	0.092	A	G	0.181	A	G	0.226
rs11656673	A	G	0.036	A	G	0.108	A	G	0.099
rs11656929	A	G	0.102	A	G	0.031	A	G	0.081
rs11657440	G	A	0.054	G	A	0.162	G	A	0.170
rs11657500	G	A	0.203	G	A	0.148	G	A	0.164
rs11658052	A	G	0.065	A	G	0.172	A	G	0.161
rs11658442	A	G	0.015	A	G	0.033	A	G	0.047
rs11775334	A	G	0.410	A	G	0.310	A	G	0.386
rs11871435	A	G	0.008	A	G	0.065	A	G	0.061
rs12046278	A	G	0.228	A	G	0.436	A	G	0.379
rs12051619	A	G	0.303	A	G	0.259	A	G	0.329

rs12051621	G	A	0.006	G	A	0.049	G	A	0.058
rs12051632	G	A	0.407	G	A	0.449	G	A	0.493
rs12165050	G	G	1.000	G	G	1.000	A	G	0.004
rs12325869	C	G	0.002	G	G	1.000	C	G	0.016
rs12449970	A	G	0.500	A	G	0.240	A	G	0.287
rs12450030	A	G	0.471	A	G	0.214	A	G	0.292
rs12450059	A	G	0.379	G	A	0.438	A	G	0.377
rs12450588	A	G	0.011	A	G	0.029	A	G	0.047
rs12451318	A	G	0.075	A	G	0.066	A	G	0.116
rs12451668	C	A	0.310	C	A	0.189	C	A	0.206
rs12453207	A	G	0.262	A	G	0.215	A	G	0.165
rs12453606	C	A	0.295	C	A	0.222	C	A	0.309
rs12602330	T	A	0.102	T	A	0.027	T	A	0.076
rs12602412	A	G	0.337	A	G	0.381	A	G	0.395
rs12602415	A	G	0.337	A	G	0.379	A	G	0.397
rs12603697	G	A	0.011	G	A	0.038	G	A	0.058
rs12603748	G	A	0.017	G	A	0.053	G	A	0.081
rs12709426	A	A	1.000	A	A	1.000	A	A	1.000
rs12935880	A	G	0.004	A	G	0.020	A	G	0.016
rs12937212	A	G	0.250	A	G	0.142	A	G	0.167
rs12939128	G	A	0.004	G	A	0.009	G	A	0.016
rs12939525	G	A	0.044	G	A	0.225	G	A	0.240
rs12940295	C	A	0.052	C	A	0.187	C	A	0.186
rs12940302	A	G	0.291	A	G	0.430	A	G	0.395
rs12942038	A	G	0.174	A	G	0.369	A	G	0.329
rs12943496	C	G	0.086	C	G	0.125	C	G	0.151
rs12943617	G	A	0.498	A	G	0.473	A	G	0.394

rs12944642	G	A	0.412	G	A	0.451	A	G	0.483
rs12945425	C	A	0.004	C	A	0.011	C	A	0.014
rs12946454	T	A	0.054	T	A	0.222	T	A	0.175
rs12946859	A	G	0.025	A	G	0.127	A	G	0.128
rs12948969	A	C	0.017	A	C	0.071	A	C	0.090
rs12950551	A	G	0.017	A	G	0.071	A	G	0.090
rs12952105	A	G	0.212	A	G	0.304	A	G	0.304
rs1378905	A	G	0.379	G	A	0.441	A	G	0.366
rs1378942	A	C	0.077	A	C	0.282	A	C	0.260
rs1530440	A	G	0.282	A	G	0.206	A	G	0.237
rs1550186	G	A	0.170	G	A	0.139	G	A	0.222
rs1563448	A	G	0.090	A	G	0.016	A	G	0.051
rs1563449	G	A	0.015	G	A	0.055	G	A	0.081
rs1631707	A	G	0.054	A	G	0.194	A	G	0.121
rs1632673	A	G	0.052	A	G	0.194	A	G	0.119
rs16948048	G	A	0.054	G	A	0.154	G	A	0.150
rs1696754	A	G	0.052	A	G	0.195	A	G	0.119
rs16970576	G	A	0.186	G	A	0.103	G	A	0.152
rs16977406	G	G	1.000	A	G	0.009	A	G	0.004
rs16982520	G	A	0.006	G	A	0.039	G	A	0.047
rs1708861	A	G	0.211	A	G	0.136	A	G	0.209
rs17248007	G	A	0.110	G	A	0.091	G	A	0.069
rs17248371	G	G	1.000	A	G	0.037	A	G	0.029
rs17249754	A	G	0.021	A	G	0.110	A	G	0.072
rs17367504	G	A	0.098	G	A	0.163	G	A	0.103
rs17545956	A	G	0.117	A	G	0.309	A	G	0.234
rs17632208	G	A	0.163	G	A	0.103	G	A	0.105

rs17742707	G	A	0.454	G	A	0.293	G	A	0.394
rs1869934	A	G	0.383	A	G	0.315	A	G	0.301
rs1918974	G	A	0.098	G	A	0.194	G	A	0.224
rs1963982	G	A	0.348	G	A	0.476	G	A	0.437
rs1965780	G	A	0.366	A	G	0.436	G	A	0.428
rs1983283	G	G	1.000	G	G	1.000	A	G	0.007
rs2004885	T	T	1.000	A	T	0.013	A	T	0.007
rs2004887	G	A	0.015	G	A	0.068	G	A	0.090
rs2033715	A	G	0.414	G	A	0.430	A	G	0.482
rs2060097	G	A	0.008	G	A	0.024	G	A	0.032
rs2121070	A	G	0.021	A	G	0.073	A	G	0.100
rs2124606	G	A	0.205	G	A	0.222	G	A	0.197
rs2279920	A	G	0.011	A	G	0.024	A	G	0.045
rs2362384	G	A	0.019	G	A	0.057	G	A	0.067
rs2377394	T	A	0.019	T	A	0.057	T	A	0.076
rs2384550	A	G	0.090	A	G	0.249	A	G	0.195
rs2398162	A	G	0.481	G	A	0.326	G	A	0.415
rs2410427	G	A	0.245	G	A	0.366	G	A	0.258
rs2411131	A	G	0.295	A	G	0.329	A	G	0.300
rs2411137	G	A	0.414	G	A	0.436	G	A	0.473
rs2435974	G	A	0.094	G	A	0.081	G	A	0.101
rs2456582	G	A	0.056	G	A	0.040	G	A	0.085
rs2466511	G	A	0.084	G	A	0.040	G	A	0.042
rs2466517	A	G	0.006	A	G	0.005	A	G	0.005
rs2509458	G	A	0.011	G	A	0.064	G	A	0.040
rs2567488	A	G	0.004	A	G	0.002	A	G	0.009
rs2567494	C	A	0.479	A	C	0.344	A	C	0.412

rs2659028	G	G	1.000	A	G	0.009	A	G	0.018
rs2681472	G	A	0.021	G	A	0.110	G	A	0.074
rs2681492	G	A	0.021	G	A	0.108	G	A	0.074
rs2713991	A	G	0.375	A	G	0.244	A	G	0.330
rs2714011	G	A	0.464	G	A	0.293	G	A	0.363
rs2725391	G	A	0.316	G	A	0.443	G	A	0.419
rs2820037	A	T	0.042	A	T	0.063	A	T	0.082
rs28607597	G	A	0.305	G	A	0.161	G	A	0.226
rs28730839	C	G	0.002	G	G	1.000	C	G	0.002
rs28758335	A	A	1.000	C	A	0.002	C	A	0.004
rs2889622	G	A	0.248	G	A	0.167	G	A	0.205
rs2889645	A	G	0.015	A	G	0.048	A	G	0.049
rs296139	G	A	0.462	A	G	0.330	A	G	0.442
rs3096277	A	G	0.306	A	G	0.200	A	G	0.238
rs3184504	A	G	0.048	A	G	0.198	A	G	0.179
rs34603877	A	G	0.002	A	G	0.004	A	G	0.005
rs34664664	A	G	0.140	A	G	0.104	A	G	0.181
rs34680431	C	C	1.000	G	C	0.004	C	C	1.000
rs34768269	G	A	0.092	G	A	0.087	G	A	0.132
rs34939985	A	G	0.017	A	G	0.073	A	G	0.092
rs35110805	G	C	0.373	C	G	0.482	G	C	0.457
rs35141294	A	G	0.002	G	G	1.000	G	G	1.000
rs35393459	G	G	1.000	G	G	1.000	A	G	0.004
rs35496088	C	A	0.136	C	A	0.150	C	A	0.193
rs35863760	G	A	0.326	G	A	0.183	G	A	0.291
rs35926122	G	A	0.015	G	A	0.051	G	A	0.047
rs36013558	A	G	0.004	A	G	0.011	A	G	0.013

rs3729659	C	C	1.000	C	C	1.000	C	C	1.000
rs3730025	G	A	0.004	G	A	0.004	G	A	0.005
rs3730043	G	G	1.000	A	G	0.002	G	G	1.000
rs3730044	A	G	0.002	A	G	0.009	A	G	0.005
rs3754777	A	G	0.257	A	G	0.397	A	G	0.285
rs381815	A	G	0.418	A	G	0.246	A	G	0.283
rs4073996	A	G	0.029	A	G	0.035	A	G	0.054
rs4074021	G	G	1.000	G	G	1.000	G	G	1.000
rs4074469	A	G	0.161	A	G	0.245	A	G	0.226
rs4239025	A	G	0.465	G	A	0.275	G	A	0.399
rs4295	C	G	0.128	C	G	0.256	C	G	0.188
rs4305	A	G	0.138	A	G	0.291	A	G	0.227
rs4309	G	A	0.146	G	A	0.311	G	A	0.271
rs4331	A	G	0.307	A	G	0.315	A	G	0.303
rs4333	A	G	0.305	A	G	0.304	A	G	0.292
rs4335	G	A	0.305	G	A	0.311	G	A	0.294
rs4341	C	G	0.307	C	G	0.315	C	G	0.303
rs4344	G	A	0.307	G	A	0.317	G	A	0.307
rs4351	G	A	0.303	G	A	0.307	G	A	0.297
rs4353	A	G	0.306	A	G	0.314	A	G	0.310
rs4362	A	G	0.304	A	G	0.304	A	G	0.302
rs4363	G	A	0.305	G	A	0.311	G	A	0.298
rs4365	G	G	1.000	A	G	0.016	A	G	0.013
rs4370013	A	T	0.492	T	A	0.368	T	A	0.313
rs4444373	G	C	0.019	G	C	0.060	G	C	0.089
rs4462668	A	C	0.464	C	A	0.467	C	A	0.458
rs448378	G	A	0.108	G	A	0.203	G	A	0.231

rs4533317	G	A	0.188	G	A	0.068	G	A	0.091
rs4592695	A	G	0.250	A	G	0.174	A	G	0.222
rs4789462	A	C	0.456	A	C	0.370	A	C	0.375
rs4789874	A	G	0.383	A	G	0.313	A	G	0.305
rs4789875	G	A	0.226	G	A	0.335	G	A	0.258
rs4789883	G	A	0.017	G	A	0.048	G	A	0.081
rs4789962	G	A	0.224	G	A	0.336	G	A	0.254
rs4789963	G	A	0.339	G	A	0.322	G	A	0.316
rs4789965	A	G	0.226	A	G	0.327	A	G	0.256
rs4789966	A	G	0.383	A	G	0.313	A	G	0.307
rs4789976	A	G	0.250	A	G	0.141	A	G	0.165
rs4789977	T	A	0.425	T	A	0.218	T	A	0.294
rs4789978	A	G	0.339	A	G	0.158	A	G	0.177
rs4789980	G	C	0.400	G	C	0.460	G	C	0.401
rs4789997	G	A	0.464	G	A	0.361	G	A	0.381
rs4790004	G	A	0.010	G	A	0.035	G	A	0.045
rs4790018	G	A	0.219	G	A	0.295	G	A	0.269
rs4790024	A	G	0.490	G	A	0.245	G	A	0.332
rs4790036	C	G	0.021	C	G	0.004	C	G	0.002
rs4790048	A	G	0.242	A	G	0.134	A	G	0.250
rs4793297	A	G	0.140	A	G	0.057	A	G	0.099
rs4793316	G	A	0.488	A	G	0.430	A	G	0.428
rs4889811	A	G	0.232	A	G	0.189	A	G	0.161
rs4889938	A	G	0.295	A	G	0.293	A	G	0.236
rs4889944	G	A	0.215	G	A	0.189	G	A	0.161
rs4975	G	G	1.000	C	G	0.002	C	G	0.004
rs6495122	C	A	0.153	C	A	0.297	C	A	0.309

rs6501298	A	G	0.008	A	G	0.020	A	G	0.032
rs6501302	A	G	0.004	A	G	0.002	A	G	0.013
rs6501571	A	G	0.471	G	A	0.396	G	A	0.440
rs6501584	G	A	0.467	G	A	0.364	A	G	0.493
rs6501587	A	G	0.462	G	A	0.451	G	A	0.486
rs6501993	G	A	0.496	G	A	0.330	G	A	0.372
rs653178	G	A	0.048	G	A	0.198	G	A	0.179
rs6565697	A	G	0.176	A	G	0.172	A	G	0.179
rs6711736	A	G	0.251	A	G	0.262	A	G	0.300
rs6729869	A	T	0.253	A	T	0.262	A	T	0.305
rs6749447	A	C	0.421	A	C	0.388	A	C	0.486
rs7209618	C	A	0.381	A	C	0.462	C	A	0.449
rs7209710	G	A	0.109	G	A	0.093	G	A	0.152
rs7210539	A	G	0.265	A	G	0.240	A	G	0.224
rs7210702	G	A	0.239	A	G	0.427	G	A	0.462
rs7210946	A	G	0.410	A	G	0.495	A	G	0.350
rs7211350	A	A	1.000	A	A	1.000	G	A	0.002
rs7211532	G	A	0.454	G	A	0.295	G	A	0.444
rs7212688	G	A	0.250	G	A	0.174	G	A	0.220
rs7213859	A	G	0.046	A	G	0.013	A	G	0.031
rs7215498	G	A	0.196	G	A	0.217	G	A	0.187
rs7215765	G	A	0.264	G	A	0.474	G	A	0.344
rs7216240	G	A	0.014	G	A	0.051	G	A	0.049
rs7216635	C	A	0.282	C	A	0.467	C	A	0.388
rs7216806	G	A	0.167	G	A	0.185	G	A	0.253
rs7217172	G	A	0.017	G	A	0.092	G	A	0.096
rs7218755	A	G	0.002	A	G	0.004	A	G	0.013

rs7219452	C	A	0.015	C	A	0.119	C	A	0.097
rs7221608	G	C	0.136	G	C	0.090	G	C	0.147
rs7222531	T	A	0.352	T	A	0.269	T	A	0.341
rs7222861	A	C	0.383	C	A	0.452	A	C	0.449
rs7223264	A	G	0.236	A	G	0.194	A	G	0.166
rs749714	A	G	0.004	A	G	0.002	A	G	0.009
rs7501499	A	G	0.165	A	G	0.075	A	G	0.130
rs7503865	G	A	0.473	A	G	0.390	A	G	0.431
rs7591163	G	A	0.125	G	A	0.326	G	A	0.276
rs8066768	A	G	0.008	A	G	0.051	A	G	0.060
rs8066857	A	G	0.052	A	G	0.118	A	G	0.154
rs8067409	A	G	0.002	A	G	0.018	A	G	0.011
rs8068871	A	G	0.288	A	G	0.202	A	G	0.273
rs8070274	G	A	0.015	G	A	0.048	G	A	0.049
rs8070383	G	G	1.000	A	G	0.022	A	G	0.020
rs8070406	C	A	0.006	C	A	0.024	C	A	0.025
rs8071004	G	A	0.010	G	A	0.075	G	A	0.085
rs8072027	C	A	0.011	C	A	0.018	C	A	0.014
rs8073197	G	A	0.082	G	A	0.062	G	A	0.076
rs8073529	A	G	0.270	A	G	0.201	A	G	0.257
rs8073791	A	G	0.008	A	G	0.051	A	G	0.060
rs8075300	A	G	0.276	A	G	0.242	A	G	0.305
rs8076416	A	G	0.042	A	G	0.218	A	G	0.181
rs8076595	G	A	0.256	G	A	0.310	G	A	0.312
rs8077205	A	G	0.247	A	G	0.234	A	G	0.269
rs8077911	C	G	0.295	C	G	0.325	C	G	0.275
rs8078791	A	G	0.034	A	G	0.168	A	G	0.157

rs8079383	A	G	0.042	A	G	0.168	A	G	0.206
rs8079757	G	A	0.284	G	A	0.090	G	A	0.078
rs8082181	A	G	0.015	A	G	0.061	A	G	0.096
rs8082289	A	G	0.251	A	G	0.172	A	G	0.217
rs866414	A	C	0.152	A	C	0.227	A	C	0.227
rs883890	C	G	0.015	C	G	0.060	C	G	0.096
rs897588	A	G	0.195	A	G	0.299	A	G	0.287
rs897593	C	A	0.299	C	A	0.463	C	A	0.439
rs907898	G	A	0.212	G	A	0.227	G	A	0.203
rs907901	A	G	0.015	A	G	0.048	A	G	0.049
rs9302890	A	C	0.235	A	C	0.465	A	C	0.329
rs9308945	G	A	0.251	G	A	0.262	G	A	0.301
rs934668	G	A	0.383	G	A	0.410	G	A	0.359
rs935334	A	G	0.021	A	G	0.073	A	G	0.097
rs938283	G	A	0.157	G	A	0.163	G	A	0.151
rs968493	C	A	0.239	C	A	0.341	C	A	0.267
rs971625	G	A	0.339	A	G	0.436	G	A	0.406
rs9789009	G	A	0.027	G	A	0.121	G	A	0.096
rs9815354	A	G	0.165	A	G	0.081	A	G	0.096
rs9893556	G	A	0.371	G	A	0.441	G	A	0.338
rs9895647	C	A	0.363	C	A	0.267	C	A	0.321
rs9896850	A	G	0.048	A	G	0.231	A	G	0.244
rs9897367	C	G	0.475	C	G	0.426	C	G	0.465
rs9897914	G	A	0.485	A	G	0.474	A	G	0.485
rs9898803	A	G	0.069	A	G	0.136	A	G	0.132
rs9899531	A	G	0.019	A	G	0.007	A	G	0.005
rs9901169	G	G	1.000	G	G	1.000	A	G	0.002

rs9902818	G	A	0.463	G	A	0.463	A	G	0.475
rs9902912	A	G	0.004	A	G	0.007	A	G	0.020
rs9904630	G	A	0.226	G	A	0.336	G	A	0.257
rs9905852	A	G	0.391	G	A	0.394	G	A	0.468
rs9907318	A	C	0.460	A	C	0.429	A	C	0.462
rs9910192	A	G	0.490	G	A	0.430	G	A	0.428
rs9910792	C	A	0.418	C	A	0.346	C	A	0.397
rs9911346	A	G	0.290	A	G	0.244	A	G	0.287
rs9912724	G	A	0.002	A	A	1.000	G	A	0.004
rs9913021	G	A	0.050	G	A	0.235	G	A	0.244
rs9914068	G	C	0.176	G	C	0.214	G	C	0.198
rs9914201	A	G	0.046	A	G	0.225	A	G	0.181
rs9914874	G	A	0.485	A	G	0.471	A	G	0.493
rs9915000	A	G	0.033	A	G	0.079	A	G	0.074
rs1000791	T	A	0.198	T	A	0.249	T	A	0.157
rs1002068	G	C	0.477	C	G	0.336	C	G	0.357
rs10073	G	A	0.250	G	A	0.253	G	A	0.252
rs1042393	G	A	0.397	G	A	0.404	G	A	0.440
rs1042395	A	G	0.398	A	G	0.411	A	G	0.440
rs1042396	A	G	0.278	A	G	0.269	A	G	0.327
rs1042397	A	G	0.346	A	G	0.349	A	G	0.393
rs1106645	A	C	0.125	A	C	0.336	A	C	0.286
rs11077409	G	A	0.282	A	G	0.429	A	G	0.451
rs11077410	A	G	0.285	G	A	0.435	G	A	0.452
rs11077426	A	G	0.302	G	A	0.494	A	G	0.484
rs11077427	G	A	0.172	G	A	0.471	G	A	0.411
rs11077428	A	G	0.415	G	A	0.489	A	G	0.498

rs11077441	A	T	0.288	A	T	0.222	A	T	0.307
rs1110734	G	A	0.400	G	A	0.352	G	A	0.456
rs11150822	A	G	0.364	A	G	0.374	A	G	0.244
rs11150824	T	A	0.471	A	T	0.489	A	T	0.426
rs11150827	A	G	0.087	A	G	0.178	A	G	0.217
rs115850223	G	G	1.000	G	G	1.000	G	G	1.000
rs11649977	A	G	0.189	A	G	0.177	A	G	0.170
rs11650313	G	A	0.165	G	A	0.151	G	A	0.195
rs11650784	A	C	0.477	C	A	0.262	C	A	0.387
rs11651201	G	A	0.390	G	A	0.454	G	A	0.349
rs11654261	G	A	0.156	G	A	0.201	G	A	0.155
rs11654770	A	G	0.288	A	G	0.405	A	G	0.341
rs11655167	T	A	0.208	T	A	0.223	T	A	0.201
rs11655455	C	A	0.054	C	A	0.264	C	A	0.270
rs11656298	G	A	0.162	G	A	0.223	G	A	0.195
rs11657933	C	G	0.065	C	G	0.351	C	G	0.319
rs11658680	A	G	0.385	A	G	0.355	A	G	0.432
rs11658702	G	A	0.304	G	A	0.227	G	A	0.232
rs117000719	G	G	1.000	A	G	0.011	A	G	0.009
rs117014247	A	G	0.002	A	G	0.005	A	G	0.005
rs117203086	A	A	1.000	G	A	0.016	G	A	0.007
rs117419007	A	G	0.002	A	G	0.007	A	G	0.002
rs118151864	A	G	0.002	G	G	1.000	A	G	0.002
rs11870326	G	A	0.054	G	A	0.053	G	A	0.077
rs11870711	A	G	0.229	A	G	0.234	A	G	0.212
rs11871318	A	G	0.285	A	G	0.207	A	G	0.315
rs11871357	A	G	0.004	A	G	0.004	A	G	0.002

rs12051618	G	A	0.302	G	A	0.258	G	A	0.323
rs12149977	G	A	0.403	G	A	0.365	G	A	0.359
rs12449822	G	A	0.242	G	A	0.294	G	A	0.294
rs12449949	A	G	0.300	A	G	0.141	A	G	0.208
rs12450239	A	G	0.437	A	G	0.358	A	G	0.319
rs12450839	A	G	0.473	A	G	0.393	A	G	0.354
rs12451047	A	G	0.350	A	G	0.421	A	G	0.449
rs12451395	C	A	0.290	C	A	0.136	C	A	0.219
rs12451560	A	G	0.355	G	A	0.476	A	G	0.485
rs12451638	A	G	0.367	A	G	0.379	A	G	0.256
rs12452616	A	G	0.250	A	G	0.176	A	G	0.231
rs12452661	G	A	0.232	G	A	0.218	G	A	0.194
rs12453678	A	G	0.263	A	G	0.216	A	G	0.161
rs12600765	A	G	0.212	A	G	0.264	A	G	0.215
rs12601898	A	G	0.057	A	G	0.227	A	G	0.198
rs12602618	G	A	0.498	G	A	0.407	G	A	0.418
rs12603040	A	G	0.490	A	G	0.283	A	G	0.316
rs12937891	C	A	0.050	C	A	0.149	C	A	0.148
rs12938262	A	G	0.293	A	G	0.428	A	G	0.396
rs12938422	C	G	0.297	C	G	0.463	C	G	0.440
rs12942476	G	A	0.210	G	A	0.185	G	A	0.158
rs12943410	A	C	0.286	A	C	0.162	A	C	0.206
rs12944002	C	A	0.335	C	A	0.359	C	A	0.436
rs12944016	G	A	0.241	G	A	0.237	G	A	0.221
rs12944841	C	G	0.031	C	G	0.122	C	G	0.122
rs12945787	A	G	0.077	A	G	0.097	A	G	0.120
rs12945963	G	A	0.488	A	G	0.483	G	A	0.471

rs12946426	A	C	0.364	C	A	0.496	A	C	0.429
rs12949118	A	G	0.494	G	A	0.313	G	A	0.361
rs12951389	A	G	0.388	G	A	0.449	A	G	0.471
rs12952612	A	G	0.452	G	A	0.487	A	G	0.457
rs1317421	G	A	0.031	G	A	0.145	G	A	0.148
rs1318314	G	A	0.113	G	A	0.213	G	A	0.235
rs1318315	A	G	0.202	A	G	0.223	A	G	0.193
rs13342272	G	A	0.168	G	A	0.240	G	A	0.214
rs1378906	G	A	0.440	G	A	0.357	A	G	0.496
rs1467979	A	G	0.046	A	G	0.099	A	G	0.088
rs15538	A	G	0.276	G	A	0.432	G	A	0.439
rs1561810	A	C	0.045	A	C	0.103	A	C	0.081
rs1561811	A	T	0.490	A	T	0.337	A	T	0.418
rs1563447	A	G	0.456	A	G	0.393	A	G	0.403
rs1663196	A	G	0.275	A	G	0.240	A	G	0.227
rs1663199	A	G	0.052	A	G	0.194	A	G	0.117
rs1675262	G	A	0.212	G	A	0.117	G	A	0.130
rs16972227	C	A	0.285	C	A	0.266	C	A	0.204
rs1800299	A	G	0.006	A	G	0.013	A	G	0.007
rs1800303	A	T	0.008	A	T	0.031	A	T	0.035
rs1800304	A	G	0.377	A	G	0.406	A	G	0.440
rs1800305	A	G	0.008	A	G	0.031	A	G	0.033
rs1800307	A	G	0.027	A	G	0.018	A	G	0.018
rs1800310	G	A	0.104	G	A	0.150	G	A	0.129
rs1800315	G	G	1.000	G	G	1.000	A	G	0.005
rs1869932	G	A	0.279	G	A	0.278	G	A	0.277
rs1870625	A	C	0.260	A	C	0.388	A	C	0.387

rs1877677	G	A	0.420	G	A	0.265	G	A	0.285
rs1993681	G	C	0.171	G	C	0.326	G	C	0.328
rs2004381	G	A	0.269	A	G	0.491	G	A	0.451
rs2013350	G	A	0.203	G	A	0.224	G	A	0.202
rs2014989	A	G	0.223	A	G	0.184	A	G	0.173
rs2076949	G	A	0.037	G	A	0.149	G	A	0.113
rs2085351	G	C	0.164	G	C	0.211	G	C	0.241
rs2124603	G	A	0.375	A	G	0.489	G	A	0.456
rs2124604	C	A	0.360	C	A	0.489	C	A	0.476
rs2165994	G	A	0.184	G	A	0.227	G	A	0.223
rs2256881	A	G	0.058	A	G	0.063	A	G	0.097
rs2279914	A	G	0.010	A	G	0.024	A	G	0.044
rs2289527	G	C	0.194	G	C	0.068	G	C	0.099
rs2289529	A	G	0.019	A	G	0.099	A	G	0.113
rs2289531	A	G	0.012	A	G	0.039	A	G	0.040
rs2289536	A	G	0.002	A	G	0.009	A	G	0.007
rs2290566	A	G	0.008	A	G	0.033	A	G	0.040
rs2304852	G	A	0.069	G	A	0.223	G	A	0.193
rs2304854	G	A	0.068	G	A	0.224	G	A	0.186
rs2361701	A	G	0.046	A	G	0.099	A	G	0.085
rs2377309	A	G	0.310	A	G	0.137	A	G	0.182
rs2377397	G	A	0.395	G	A	0.377	G	A	0.337
rs2377400	A	G	0.035	A	G	0.184	A	G	0.149
rs2377404	G	A	0.428	G	A	0.265	G	A	0.274
rs2606183	G	A	0.263	G	A	0.300	G	A	0.208
rs2606188	G	A	0.258	G	A	0.310	G	A	0.203
rs2612753	A	C	0.390	A	C	0.493	A	C	0.405

rs2612771	G	A	0.358	G	A	0.438	G	A	0.491
rs2612773	A	G	0.279	A	G	0.440	A	G	0.392
rs2612782	A	G	0.219	A	G	0.136	A	G	0.151
rs2612788	C	A	0.247	C	A	0.353	C	A	0.358
rs2659003	C	G	0.058	C	G	0.062	C	G	0.093
rs2659029	A	G	0.052	A	G	0.082	A	G	0.104
rs2659030	G	A	0.113	G	A	0.314	G	A	0.363
rs2707031	C	G	0.341	C	G	0.437	C	G	0.401
rs2889619	T	A	0.322	T	A	0.228	T	A	0.237
rs2889620	G	A	0.463	A	G	0.275	A	G	0.401
rs34237952	G	C	0.083	G	C	0.097	G	C	0.115
rs34262564	A	T	0.215	A	T	0.189	A	T	0.169
rs34520486	G	A	0.280	G	A	0.237	G	A	0.269
rs34547368	A	G	0.373	A	G	0.275	A	G	0.350
rs34874100	G	G	1.000	G	G	1.000	A	G	0.004
rs35035542	A	G	0.002	A	G	0.013	A	G	0.004
rs35435268	A	G	0.004	A	G	0.005	A	G	0.007
rs35578653	C	A	0.304	C	A	0.310	C	A	0.366
rs3744181	G	A	0.446	A	G	0.359	A	G	0.383
rs3744182	A	G	0.006	A	G	0.053	A	G	0.029
rs3744186	C	A	0.448	A	C	0.429	A	C	0.422
rs3751947	A	G	0.008	A	G	0.057	A	G	0.103
rs3764438	A	G	0.015	A	G	0.051	A	G	0.068
rs3764440	G	A	0.021	G	A	0.106	G	A	0.133
rs3803780	G	A	0.004	G	A	0.022	G	A	0.011
rs3809713	G	A	0.315	G	A	0.277	G	A	0.301
rs3934967	G	A	0.348	G	A	0.240	G	A	0.296

rs4074022	C	G	0.367	C	G	0.392	C	G	0.407
rs4074023	G	A	0.319	G	A	0.330	G	A	0.385
rs4239026	G	C	0.463	C	G	0.271	C	G	0.400
rs4243249	A	G	0.221	A	G	0.209	A	G	0.177
rs4441315	A	G	0.131	A	G	0.344	A	G	0.299
rs4482327	G	A	0.250	G	A	0.174	G	A	0.224
rs4485403	G	A	0.019	G	A	0.104	G	A	0.115
rs4541108	A	G	0.359	A	G	0.380	A	G	0.405
rs4622540	C	A	0.465	A	C	0.449	C	A	0.485
rs4789853	G	C	0.242	G	C	0.491	G	C	0.485
rs4789878	G	C	0.183	G	C	0.170	G	C	0.162
rs4789879	A	G	0.292	A	G	0.162	A	G	0.176
rs4789885	G	A	0.485	A	G	0.430	A	G	0.423
rs4789887	A	C	0.191	A	C	0.264	A	C	0.250
rs4789888	A	G	0.285	A	G	0.201	A	G	0.241
rs4789890	C	A	0.168	C	A	0.125	C	A	0.123
rs4789892	A	G	0.063	A	G	0.154	A	G	0.110
rs4789893	C	G	0.246	C	G	0.476	C	G	0.443
rs4789911	A	G	0.292	G	A	0.450	G	A	0.495
rs4789949	T	A	0.337	T	A	0.471	T	A	0.495
rs4789951	A	G	0.246	A	G	0.423	A	G	0.435
rs4789964	C	G	0.342	C	G	0.325	C	G	0.325
rs4789967	C	G	0.047	C	G	0.036	C	G	0.107
rs4789968	A	G	0.013	A	G	0.057	A	G	0.075
rs4789979	A	G	0.402	G	A	0.382	G	A	0.441
rs4789994	G	A	0.485	G	A	0.432	A	G	0.489
rs4789998	G	A	0.461	G	A	0.364	G	A	0.395

rs4789999	A	C	0.162	A	C	0.337	A	C	0.341
rs4790000	A	G	0.038	A	G	0.183	A	G	0.161
rs4790001	A	G	0.315	G	A	0.465	G	A	0.456
rs4790002	A	T	0.296	A	T	0.449	A	T	0.432
rs4790005	A	G	0.290	A	G	0.435	A	G	0.420
rs4790007	A	G	0.469	G	A	0.487	A	G	0.500
rs4790009	A	G	0.494	A	G	0.269	A	G	0.304
rs4790013	G	A	0.494	A	G	0.484	G	A	0.473
rs4790019	A	G	0.366	A	G	0.339	A	G	0.330
rs4790032	G	A	0.279	G	A	0.355	G	A	0.362
rs4889809	A	C	0.256	A	C	0.225	A	C	0.175
rs4889810	A	G	0.129	A	G	0.190	A	G	0.250
rs4889815	A	G	0.106	A	G	0.070	A	G	0.057
rs4889940	A	G	0.426	A	G	0.248	A	G	0.367
rs4889954	G	A	0.456	A	G	0.452	G	A	0.474
rs4889961	C	G	0.456	G	C	0.485	C	G	0.453
rs55714027	G	A	0.446	A	G	0.426	A	G	0.480
rs56107536	C	A	0.315	A	C	0.318	A	C	0.346
rs56259513	A	G	0.221	A	G	0.270	A	G	0.270
rs56353542	G	A	0.006	G	A	0.027	G	A	0.031
rs56407805	G	A	0.058	G	A	0.149	G	A	0.131
rs59152781	G	A	0.002	G	A	0.004	G	A	0.005
rs59652033	G	A	0.140	G	A	0.386	G	A	0.338
rs59896146	G	G	1.000	G	G	1.000	G	G	1.000
rs59978698	G	G	1.000	A	G	0.004	G	G	1.000
rs60582626	A	G	0.367	G	A	0.463	A	G	0.495
rs60684213	A	G	0.014	A	G	0.043	A	G	0.037

rs61729127	G	A	0.006	G	A	0.005	G	A	0.007
rs61734950	G	G	1.000	G	G	1.000	A	G	0.007
rs61740509	A	G	0.037	A	G	0.052	A	G	0.055
rs61749027	G	G	1.000	G	G	1.000	A	G	0.002
rs61756761	G	A	0.014	G	A	0.038	G	A	0.019
rs61998241	G	G	1.000	G	G	1.000	G	G	1.000
rs62000409	G	G	1.000	G	G	1.000	G	G	1.000
rs62063818	A	T	0.006	A	T	0.027	A	T	0.031
rs62063824	A	G	0.006	A	G	0.022	A	G	0.027
rs62063831	G	A	0.296	G	A	0.266	G	A	0.255
rs62074551	C	A	0.487	C	A	0.418	C	A	0.434
rs6501285	A	G	0.219	A	G	0.275	A	G	0.359
rs6565633	A	C	0.202	A	C	0.393	A	C	0.296
rs6565635	A	G	0.221	A	G	0.397	A	G	0.308
rs67296984	A	G	0.021	A	G	0.110	A	G	0.122
rs715041	A	G	0.033	A	G	0.060	A	G	0.060
rs7207088	A	C	0.251	A	C	0.172	A	C	0.225
rs7208049	T	A	0.027	T	A	0.103	T	A	0.119
rs7209293	A	G	0.250	A	G	0.170	A	G	0.219
rs7209428	A	G	0.087	A	G	0.170	A	G	0.214
rs7210391	A	C	0.135	A	C	0.097	A	C	0.144
rs7210947	G	A	0.437	G	A	0.498	G	A	0.489
rs7211021	A	G	0.081	A	G	0.106	A	G	0.119
rs7212305	A	G	0.251	A	G	0.173	A	G	0.224
rs7212486	G	A	0.360	G	A	0.337	G	A	0.321
rs7213410	G	A	0.012	G	A	0.040	G	A	0.055
rs7213735	A	G	0.440	A	G	0.372	A	G	0.330

rs7215470	C	A	0.258	C	A	0.297	C	A	0.258
rs7217721	G	G	1.000	C	G	0.022	C	G	0.018
rs7217829	A	T	0.324	A	T	0.307	A	T	0.339
rs7218261	A	C	0.107	A	C	0.208	A	C	0.158
rs7218918	A	G	0.140	A	G	0.206	A	G	0.196
rs7220493	A	C	0.271	A	C	0.239	A	C	0.221
rs7220592	G	C	0.437	G	C	0.360	G	C	0.354
rs7220985	C	G	0.338	C	G	0.379	C	G	0.396
rs7221604	A	G	0.377	A	G	0.407	A	G	0.440
rs7223756	A	G	0.477	A	G	0.425	A	G	0.465
rs7224728	G	A	0.046	G	A	0.044	G	A	0.076
rs7225364	C	A	0.330	C	A	0.478	A	C	0.428
rs7225655	G	A	0.314	A	G	0.477	A	G	0.468
rs7225663	C	A	0.138	C	A	0.312	C	A	0.319
rs7226158	A	G	0.127	A	G	0.260	A	G	0.277
rs72849330	G	C	0.207	G	C	0.189	G	C	0.150
rs72852409	A	G	0.123	A	G	0.226	A	G	0.190
rs72853543	A	G	0.187	A	G	0.181	A	G	0.181
rs73412102	G	A	0.004	G	A	0.018	G	A	0.029
rs7350896	G	A	0.317	A	G	0.467	A	G	0.463
rs73999901	A	G	0.012	A	G	0.038	A	G	0.040
rs74000351	G	G	1.000	G	G	1.000	A	G	0.004
rs74006007	G	G	1.000	A	G	0.002	A	G	0.013
rs74692882	A	G	0.008	G	G	1.000	G	G	1.000
rs751848	A	C	0.239	A	C	0.303	A	C	0.279
rs75560495	G	A	0.008	G	A	0.066	G	A	0.044
rs76290800	A	C	0.405	A	C	0.487	C	A	0.428

rs76299544	A	G	0.137	A	G	0.086	A	G	0.141
rs76524459	G	A	0.006	G	A	0.068	G	A	0.057
rs79087642	A	G	0.035	A	G	0.128	A	G	0.095
rs79487324	G	G	1.000	G	G	1.000	G	G	1.000
rs79861199	G	G	1.000	G	G	1.000	G	G	1.000
rs80198744	A	G	0.047	A	G	0.060	A	G	0.097
rs8064357	A	G	0.420	A	G	0.239	A	G	0.387
rs8064769	A	G	0.250	A	G	0.173	A	G	0.224
rs8065431	G	A	0.217	G	A	0.394	G	A	0.387
rs8067167	A	G	0.429	A	G	0.336	A	G	0.491
rs8067885	G	A	0.262	G	A	0.206	G	A	0.207
rs8069502	A	G	0.223	A	G	0.223	A	G	0.211
rs8070973	C	A	0.425	A	C	0.443	C	A	0.438
rs8071693	A	G	0.012	A	G	0.024	A	G	0.046
rs8072347	G	A	0.435	A	G	0.434	A	G	0.440
rs8073077	A	G	0.044	A	G	0.225	A	G	0.241
rs8073550	A	G	0.269	A	G	0.463	A	G	0.409
rs8074418	G	A	0.239	G	A	0.216	G	A	0.217
rs8075376	A	G	0.229	A	G	0.235	A	G	0.215
rs8075657	A	G	0.254	A	G	0.304	A	G	0.304
rs8076283	G	A	0.266	G	A	0.463	G	A	0.427
rs8076334	A	G	0.174	A	G	0.264	A	G	0.222
rs8078334	G	A	0.319	G	A	0.274	G	A	0.300
rs8078752	A	G	0.496	A	G	0.368	A	G	0.387
rs8079646	A	G	0.188	A	G	0.179	A	G	0.175
rs8080101	G	A	0.325	G	A	0.465	G	A	0.454
rs8080155	G	A	0.319	G	A	0.275	G	A	0.301

rs8080597	A	C	0.028	A	C	0.096	A	C	0.103
rs8081143	C	A	0.375	A	C	0.487	C	A	0.454
rs8081466	G	A	0.250	G	A	0.180	G	A	0.231
rs8082640	A	G	0.362	G	A	0.489	A	G	0.456
rs871741	A	G	0.440	A	G	0.375	A	G	0.326
rs884446	A	G	0.180	A	G	0.185	A	G	0.190
rs884652	A	C	0.167	A	C	0.093	A	C	0.155
rs894310	A	C	0.021	A	C	0.110	A	C	0.122
rs897587	A	G	0.114	A	G	0.218	A	G	0.236
rs897595	G	A	0.475	A	G	0.277	A	G	0.383
rs897597	C	G	0.412	G	C	0.242	G	C	0.261
rs897600	A	G	0.203	A	G	0.221	A	G	0.198
rs898525	A	T	0.240	A	T	0.495	A	T	0.369
rs898533	G	A	0.170	G	A	0.244	G	A	0.179
rs898534	A	G	0.171	A	G	0.238	A	G	0.166
rs907899	G	A	0.408	G	A	0.427	G	A	0.446
rs907911	A	G	0.176	A	G	0.172	A	G	0.181
rs907915	G	A	0.269	G	A	0.239	G	A	0.223
rs925607	G	A	0.049	G	A	0.163	G	A	0.146
rs9302897	G	A	0.113	G	A	0.206	G	A	0.223
rs9319623	G	C	0.483	G	C	0.465	G	C	0.447
rs9635664	G	A	0.479	A	G	0.335	A	G	0.367
rs9675239	G	A	0.227	G	A	0.342	G	A	0.324
rs969413	A	T	0.099	A	T	0.306	A	T	0.344
rs971626	A	G	0.337	G	A	0.439	A	G	0.403
rs978415	A	G	0.228	A	G	0.225	A	G	0.223
rs981645	G	C	0.177	G	C	0.284	G	C	0.252

rs9890248	G	C	0.106	G	C	0.167	G	C	0.123
rs9894131	A	C	0.035	A	C	0.119	A	C	0.124
rs9894139	A	G	0.390	G	A	0.393	G	A	0.471
rs9894899	C	A	0.227	C	A	0.225	C	A	0.214
rs9895586	C	G	0.410	G	C	0.487	C	G	0.495
rs9896146	A	T	0.160	A	T	0.297	A	T	0.294
rs9897730	A	G	0.251	A	G	0.246	A	G	0.242
rs9898046	A	C	0.240	A	C	0.236	A	C	0.217
rs9898469	A	G	0.305	A	G	0.161	A	G	0.230
rs9900690	C	A	0.199	C	A	0.326	C	A	0.276
rs9904414	G	A	0.371	G	A	0.339	G	A	0.378
rs9904772	G	A	0.033	G	A	0.139	G	A	0.125
rs9904783	G	A	0.265	G	A	0.251	G	A	0.243
rs9904969	C	G	0.305	C	G	0.176	C	G	0.234
rs9905685	C	G	0.456	G	C	0.452	C	G	0.474
rs9906023	A	G	0.306	A	G	0.165	A	G	0.237
rs9906525	G	A	0.394	A	G	0.385	A	G	0.464
rs9906700	G	A	0.435	G	A	0.240	G	A	0.316
rs9907094	T	A	0.387	A	T	0.452	T	A	0.469
rs9907544	A	G	0.081	A	G	0.264	A	G	0.299
rs9907837	A	C	0.485	A	C	0.460	A	C	0.447
rs9910615	G	A	0.421	G	A	0.169	G	A	0.259
rs9911245	G	A	0.317	G	A	0.368	G	A	0.422
rs9911538	A	G	0.319	A	G	0.271	A	G	0.286
rs9916688	G	A	0.021	G	A	0.106	G	A	0.139
rs9944501	A	T	0.025	A	T	0.141	A	T	0.150

Table S5. Allele frequencies in the Strong Heart Study cohort

	AZ			SD			OK		
SNP	Minor allele	Major allele	Minor allele frequency	Minor allele	Major allele	Minor allele frequency	Minor allele	Major allele	Minor allele frequency
rs1106645	A	C	0.086	A	C	0.271	A	C	0.250
rs11077416	A	G	0.301	A	G	0.203	A	G	0.308
rs11077421	A	T	0.333	A	T	0.490	A	T	0.490
rs11077428	A	G	0.428	A	G	0.457	A	G	0.477
rs11150821	A	C	0.384	A	C	0.421	A	C	0.297
rs11150824	T	A	0.427	A	T	0.488	A	T	0.439
rs1115834	A	G	0.074	A	G	0.119	A	G	0.152
rs11650154	G	A	0.046	G	A	0.025	G	A	0.046
rs11650709	A	G	0.431	G	A	0.302	G	A	0.378
rs11651201	G	A	0.385	G	A	0.488	G	A	0.388
rs11653700	A	G	0.057	A	G	0.121	A	G	0.188
rs11656929	A	G	0.131	A	G	0.020	A	G	0.071
rs12051619	A	G	0.302	A	G	0.203	A	G	0.306
rs12051621	G	A	0.004	G	A	0.044	G	A	0.048
rs12450030	G	A	0.489	A	G	0.259	A	G	0.293
rs12450059	A	G	0.399	G	A	0.411	A	G	0.431
rs12451668	C	A	0.367	C	A	0.206	C	A	0.197
rs12453207	A	G	0.297	A	G	0.253	A	G	0.220
rs12601898	A	G	0.034	A	G	0.222	A	G	0.186
rs12602330	T	A	0.136	T	A	0.014	T	A	0.073
rs12935880	G	G	1.000	A	G	0.022	A	G	0.009
rs12937212	A	G	0.252	A	G	0.140	A	G	0.159

rs12939525	G	A	0.027	G	A	0.165	G	A	0.158
rs12940295	C	A	0.033	C	A	0.157	C	A	0.141
rs12942038	A	G	0.137	A	G	0.331	A	G	0.296
rs12943496	C	G	0.044	C	G	0.109	C	G	0.133
rs12946426	A	C	0.364	A	C	0.477	A	C	0.449
rs12951389	A	G	0.370	G	A	0.490	G	A	0.490
rs1378905	A	G	0.399	G	A	0.411	A	G	0.424
rs1563448	A	G	0.104	A	G	0.035	A	G	0.047
rs2004381	G	A	0.214	G	A	0.452	G	A	0.450
rs2279914	A	G	0.009	A	G	0.058	A	G	0.044
rs2279920	A	G	0.009	A	G	0.059	A	G	0.045
rs2290566	A	G	0.006	A	G	0.047	A	G	0.031
rs2362384	G	A	0.006	G	A	0.031	G	A	0.057
rs2456582	G	A	0.046	G	A	0.040	G	A	0.094
rs2659028	A	G	0.001	A	G	0.005	A	G	0.010
rs2659030	G	A	0.076	G	A	0.244	G	A	0.286
rs2725391	G	A	0.304	G	A	0.404	G	A	0.399
rs28607597	G	A	0.314	G	A	0.173	G	A	0.235
rs2889620	G	A	0.431	A	G	0.302	A	G	0.369
rs34664664	A	G	0.141	A	G	0.081	A	G	0.155
rs34768269	G	A	0.077	G	A	0.069	G	A	0.108
rs35110805	G	C	0.334	G	C	0.461	G	C	0.486
rs4239025	A	G	0.430	G	A	0.303	G	A	0.370
rs4239026	G	C	0.431	C	G	0.300	C	G	0.368
rs4789875	G	A	0.220	G	A	0.338	G	A	0.296
rs4789878	G	C	0.200	G	C	0.175	G	C	0.143
rs4789890	C	A	0.163	C	A	0.147	C	A	0.151

rs4789965	A	G	0.218	A	G	0.331	A	G	0.287
rs4789977	T	A	0.457	T	A	0.267	T	A	0.304
rs4789979	A	G	0.376	G	A	0.385	G	A	0.433
rs4790004	G	A	0.006	G	A	0.048	G	A	0.034
rs4790018	G	A	0.172	G	A	0.275	G	A	0.270
rs6501297	A	G	0.469	A	G	0.452	A	G	0.446
rs7209618	C	A	0.368	A	C	0.499	C	A	0.486
rs7211532	G	A	0.476	G	A	0.292	G	A	0.420
rs7216635	C	A	0.266	C	A	0.443	C	A	0.368
rs7217172	G	A	0.007	G	A	0.088	G	A	0.077
rs7219452	C	A	0.007	C	A	0.119	C	A	0.096
rs7221608	G	C	0.143	G	C	0.071	G	C	0.138
rs7225364	C	A	0.256	C	A	0.363	C	A	0.486
rs7225663	C	A	0.118	C	A	0.297	C	A	0.300
rs7226158	A	G	0.109	A	G	0.264	A	G	0.271
rs7501499	A	G	0.169	A	G	0.095	A	G	0.144
rs8066768	A	G	0.004	A	G	0.044	A	G	0.048
rs8068871	A	G	0.296	A	G	0.167	A	G	0.254
rs8070406	C	A	0.001	C	A	0.015	C	A	0.017
rs8070973	C	A	0.433	A	C	0.426	C	A	0.478
rs8071004	G	A	0.006	G	A	0.076	G	A	0.069
rs8071693	A	G	0.009	A	G	0.058	A	G	0.044
rs8073077	A	G	0.026	A	G	0.159	A	G	0.152
rs8073791	A	G	0.005	A	G	0.043	A	G	0.048
rs8076416	A	G	0.020	A	G	0.160	A	G	0.166
rs8079383	A	G	0.017	A	G	0.085	A	G	0.115
rs8082640	A	G	0.327	A	G	0.453	A	G	0.468

rs884652	A	C	0.180	A	C	0.084	A	C	0.141
rs897597	C	G	0.363	G	C	0.316	G	C	0.334
rs925607	G	A	0.026	G	A	0.131	G	A	0.107
rs969413	A	T	0.071	A	T	0.232	A	T	0.267
rs971625	G	A	0.304	A	G	0.408	G	A	0.396
rs9789009	G	A	0.014	G	A	0.119	G	A	0.085
rs9893556	G	A	0.358	A	G	0.500	G	A	0.337
rs9894139	A	G	0.342	G	A	0.442	G	A	0.461
rs9895586	C	G	0.428	C	G	0.457	C	G	0.478
rs9896850	A	G	0.027	A	G	0.167	A	G	0.158
rs9905852	A	G	0.341	G	A	0.441	G	A	0.461
rs9906525	G	A	0.343	A	G	0.435	A	G	0.457
rs9907094	T	A	0.370	A	T	0.490	A	T	0.489
rs9907544	A	G	0.045	A	G	0.211	A	G	0.235
rs9911245	G	A	0.266	G	A	0.340	G	A	0.382

Table S6. Meta-analysis of sex-specific estimates for SBP – females

	Individuals genotyped in Panel 1					Individuals genotyped in Panel 2 but not in Panel 1 (replication)				Individuals genotyped in Panel 2 (includes individuals in Panel 1)				SHS Cohort (replication)			
**SNP**	**Coded allele**	**Freq coded allele**	**beta**	**SE**	**P**	**Freq coded allele**	**beta**	**SE**	**P**	**Freq coded allele**	**beta**	**SE**	**P**	**Freq coded allele**	**beta**	**SE**	**P**
rs1000791	A	0.75	-0.6585	0.7038	0.35	0.78	-0.0328	0.4543	0.94	0.77	-0.1786	0.3883	0.65				
rs1000821	A	0.42	-0.3613	0.6467	0.58												
rs1002068	C	0.42	0.2529	0.6395	0.69	0.41	0.1924	0.3883	0.62	0.41	0.1587	0.337	0.64				
rs1004467	A	0.81	1.2183	0.7697	0.11	0.79	-0.3393	0.4600	0.46	0.79	-0.0763	0.4039	0.85				
rs1006809	A	0.19	0.2596	0.8332	0.76												
rs10073	A	0.76	-1.4035	0.7033	0.05	0.76	-0.5986	0.4408	0.17	0.76	-0.7839	0.3773	0.04				
rs1007464	A	0.53	-0.7785	0.6126	0.20	0.51	-0.1430	0.3706	0.70	0.51	-0.3035	0.3223	0.35				
rs1007850	A	0.83	0.7191	0.8687	0.41												
rs1008177	A	0.47	-0.167	0.6079	0.78												
rs1010161	A	0.48	0.5085	0.6178	0.41												
rs1010774	A	0.67	0.0621	0.6616	0.93												
rs1012117	A	0.23	0.3278	0.7318	0.65												
rs1013013	A	0.14	0.5173	1.0265	0.61												
rs1014390	A	0.15	-1.3909	0.9598	0.15												
rs1028060	A	0.79	1.1793	0.776	0.13	0.78	0.048	0.4616	0.92	0.78	0.1772	0.4006	0.66				
rs10338	A	0.63	0.6026	0.7007	0.39												
rs103550	C	0.62	2.5372	0.6694	0.00												
rs10401019	A	0.52	1.5148	0.6208	0.01												
rs1042393	A	0.63	-0.0188	0.6402	0.98	0.62	-0.099	0.3740	0.79	0.62	-0.0324	0.3299	0.92				
rs1042395	A	0.38	0.0473	0.6393	0.94	0.38	-0.1474	0.3735	0.69	0.38	-0.0563	0.3294	0.86				
rs1042396	A	0.29	0.6881	0.7607	0.37	0.28	0.0966	0.4610	0.83	0.28	0.3363	0.4028	0.40				
rs1042397	A	0.32	0.4086	0.6663	0.54	0.32	0.0715	0.3935	0.86	0.32	0.2357	0.3457	0.50				
rs1042489	A	0.56	-0.0461	0.6147	0.94												
rs1042542	A	0.42	0.6269	0.6378	0.33												
rs1044228	A	0.44	-0.0259	0.6212	0.97												
rs1044282	A	0.53	0.4287	0.6185	0.49												

rs1044433	A	0.36	0.7795	0.6549	0.23												
rs10445219	C	0.88	1.102	1.0642	0.30	0.87	-1.0365	0.57	0.07	0.88	-0.5461	0.511	0.29				
rs10445220	A	0.27	-0.9267	0.6962	0.18	0.24	0.0783	0.4328	0.86	0.25	-0.2071	0.3726	0.58				
rs10445407	A	0.34	-0.8699	0.6575	0.19												
rs1046446	A	0.31	0.6674	0.6942	0.34												
rs1047743	C	0.39	-0.1172	0.6636	0.86												
rs1048775	C	0.78	-2.1052	0.8208	0.01												
rs10491334	A	0.11	-1.037	1.048	0.32	0.06	-0.71	0.7613	0.35	0.07	-0.672	0.6206	0.28				
rs10493340	A	0.67	1.367	0.6613	0.04	0.68	-0.6959	0.4035	0.08	0.68	-0.1535	0.3502	0.66				
rs10495809	A	0.20	0.9893	0.7789	0.20	0.19	0.6112	0.4846	0.21	0.20	0.7392	0.416	0.08				
rs10512597	A	0.53	0.0979	0.6179	0.87												
rs10512601	C	0.20	-0.0552	0.8375	0.95												
rs10512604	A	0.29	-0.2215	0.7622	0.77												
rs10512613	A	0.72	-0.2781	0.6963	0.69												
rs10512617	C	0.44	1.1682	0.6225	0.06												
rs1055086	A	0.30	0.0319	0.6778	0.96	0.28	0.3514	0.4103	0.39	0.28	0.3195	0.3566	0.37				
rs1055129	A	0.80	-0.006	0.7744	0.99												
rs1057040	A	0.43	1.0936	0.6272	0.08												
rs1062935	A	0.38	0.25	0.6409	0.70												
rs1065768	A	0.37	-1.1248	1.1117	0.31												
rs1071664	A	0.29	-0.9525	0.782	0.22												
rs10775361	A	0.12	-1.014	1.0298	0.32	0.11	0.4142	0.6204	0.50	0.11	0.0619	0.5354	0.91				
rs10775365	A	0.41	1.2297	0.6426	0.06												
rs1077693	A	0.22	0.1521	0.7693	0.84	0.27	-0.102	0.4462	0.82	0.26	-0.0435	0.3931	0.91				
rs1079133	A	0.13	0.2455	1.1175	0.83												
rs10852766	A	0.40	0.2626	0.6515	0.69												
rs10852778	A	0.26	-0.2775	0.7072	0.69												
rs10871489	A	0.86	1.2092	1.0262	0.24												
rs11014166	A	0.12	-0.3446	1.0011	0.73	0.11	-0.3565	0.6204	0.57	0.11	-0.3915	0.5381	0.47				
rs11024074	A	0.61	0.8867	0.6533	0.17	0.66	0.2057	0.4032	0.61	0.65	0.4108	0.3485	0.24				
rs1106221	C	0.98	-0.2902	2.96	0.92	0.98	-0.5586	1.6643	0.74	0.98	-0.0423	1.4277	0.98				
rs1106281	A	0.87	-1.0772	1.0702	0.31	0.87	-0.5041	0.6165	0.41	0.88	-0.7112	0.5407	0.19				
rs11065987	A	0.81	-0.8836	1.0652	0.41	0.87	-0.2442	0.6045	0.69	0.86	-0.4156	0.5285	0.43				
rs1106645	A	0.26	0.8861	0.7693	0.25	0.26	0.0059	0.4509	0.99	0.26	0.2604	0.3934	0.51	0.23	-0.2888	0.4001	0.47

rs11077405	A	0.79	1.1152	0.7575	0.14												
rs11077409	A	0.54	-0.5807	0.6547	0.38	0.50	0.1216	0.3937	0.76	0.52	-0.1114	0.3435	0.75				
rs11077410	A	0.46	-0.6702	0.6466	0.30	0.49	0.2238	0.3909	0.57	0.48	-0.0556	0.3406	0.87				
rs11077416	A	0.32	1.0318	0.6652	0.12	0.28	-0.2882	0.4208	0.49	0.29	0.1069	0.3618	0.77	0.27	-0.0608	0.3498	0.86
rs11077420	A	0.19	-0.2433	0.8041	0.76	0.22	-0.2107	0.4825	0.66	0.21	-0.1287	0.4191	0.76				
rs11077421	A	0.42	-1.1815	0.6221	0.06	0.44	-0.0671	0.3695	0.86	0.44	-0.38	0.3233	0.24	0.43	-0.3039	0.3180	0.34
rs11077426	A	0.38	1.2119	0.6235	0.05												
rs11077427	A	0.61	0.0168	0.6773	0.98	0.64	-0.3757	0.4039	0.35	0.63	-0.2623	0.3517	0.46				
rs11077428	A	0.48	-0.316	0.6238	0.61	0.46	-0.4193	0.3748	0.26	0.46	-0.3449	0.3244	0.29	0.45	0.1162	0.3221	0.72
rs11077435	A	0.95	-1.5939	2.1347	0.46	0.96	-1.2104	0.9871	0.22	0.96	-1.6415	0.8934	0.07				
rs11077441	A	0.30	-0.337	0.6919	0.63	0.28	-0.4135	0.4152	0.32	0.29	-0.3201	0.3598	0.37				
rs11077628	A	0.07	-0.7629	1.8146	0.67	0.05	0.5424	0.8871	0.54	0.05	0.5572	0.7786	0.47				
rs11077637	A	0.57	-0.0418	0.6439	0.95	0.54	0.5384	0.3779	0.15	0.54	0.4611	0.3308	0.16				
rs11077736	A	0.23	0.4287	0.7599	0.57												
rs11077772	A	0.10	-0.993	1.0342	0.34												
rs11077773	A	0.88	-0.3899	0.9618	0.69												
rs11077787	C	0.10	0.7197	1.2123	0.55												
rs11077793	A	0.27	0.5958	0.7242	0.41												
rs11077799	A	0.75	-0.8679	0.7361	0.24												
rs11077813	A	0.72	0.483	0.7187	0.50												
rs11077815	A	0.47	-0.5691	0.6251	0.36												
rs11077817	A	0.39	0.3478	0.6393	0.59												
rs11077829	A	0.15	-0.8712	0.8916	0.33												
rs11077874	A	0.50	-1.0059	0.6328	0.11												
rs11077876	A	0.64	0.6325	0.6369	0.32												
rs11077879	A	0.27	1.9409	0.7222	0.01												
rs11077880	A	0.60	0.2412	0.6467	0.71												
rs11077892	A	0.28	-0.2086	0.6979	0.77												
rs11077909	A	0.86	-0.6008	0.8772	0.49												
rs11077919	A	0.69	0.3718	0.7294	0.61	0.68	-0.3022	0.4234	0.48	0.68	-0.0645	0.3678	0.86				
rs11077938	A	0.74	1.1578	0.7361	0.12	0.75	0.0005	0.4369	1.00	0.75	0.2773	0.3791	0.46				
rs11077964	A	0.55	0.5205	0.6059	0.39												
rs11077969	A	0.78	1.2594	0.7341	0.09												
rs1108355	A	0.09	-0.2289	1.3874	0.87												

rs1108365	A	0.56	0.2581	0.6284	0.68												
rs1108366	A	0.28	-0.9179	0.675	0.17												
rs1109033	A	0.10	0.7197	1.2123	0.55												
rs1110274	A	0.43	0.4815	0.6414	0.45												
rs1110734	A	0.64	-0.1706	0.6589	0.80	0.60	-0.3795	0.3815	0.32	0.61	-0.2333	0.3338	0.48				
rs1113758	A	0.72	-0.2634	0.7422	0.72												
rs11150736	A	0.11	0.06	1.0781	0.96												
rs11150738	A	0.91	0.6793	1.1526	0.56												
rs11150739	A	0.92	1.1525	1.5234	0.45												
rs11150746	A	0.81	0.0821	0.8319	0.92												
rs11150780	A	0.76	-1.8961	0.7133	0.01												
rs11150784	A	0.71	-1.2903	0.6874	0.06												
rs11150821	A	0.39	0.4071	0.6197	0.51	0.34	0.4661	0.3954	0.24	0.35	0.4577	0.3395	0.18	0.37	-0.2281	0.3213	0.48
rs11150822	A	0.38	0.4215	0.6235	0.50	0.34	0.3676	0.3966	0.35	0.35	0.3833	0.3409	0.26				
rs11150823	A					0.03	-0.4898	1.316	0.71	0.02	-0.7588	1.1016	0.49				
rs11150824	A	0.54	0.829	0.6005	0.17	0.48	0.038	0.37	0.92	0.50	0.3126	0.3196	0.33	0.51	0.1659	0.3156	0.60
rs11150827	A	0.14	0.3745	0.9448	0.69	0.15	-1.091	0.5278	0.04	0.15	-0.7301	0.4717	0.12				
rs11150847	A	0.37	-0.1943	0.6492	0.76												
rs11150849	C	0.74	0.0719	0.7056	0.92												
rs1115834	A	0.13	1.0045	0.9116	0.27	0.15	-0.285	0.5314	0.59	0.14	0.0672	0.4665	0.89	0.12	0.143	0.5005	0.78
rs11191548	A	0.81	1.2568	0.7712	0.10	0.79	-0.6474	0.4629	0.16	0.80	-0.276	0.406	0.50				
rs1124736	A	0.19	-0.0089	0.819	0.99	0.18	-0.5028	0.4812	0.30	0.18	-0.3166	0.4225	0.45				
rs1126690	A	0.43	0.14	0.6033	0.82												
rs1127678	A	0.07	0.8319	1.3994	0.55												
rs1128889	A	0.42	0.1603	0.6351	0.80												
rs11308563	A	0.42	-1.2633	0.6254	0.04	0.42	-0.3242	0.3752	0.39	0.42	-0.4579	0.3284	0.16				
rs1137582	C	0.40	0.2829	0.6525	0.66												
rs11646213	A	0.31	0.1016	0.6801	0.88	0.37	0.2096	0.384	0.59	0.35	0.1863	0.3379	0.58				
rs11649977	A	0.22	-1.0014	0.736	0.17	0.17	-0.1071	0.5001	0.83	0.19	-0.3774	0.424	0.37				
rs11650154	A	0.68	1.0318	0.6652	0.12	0.72	-0.3275	0.4207	0.44	0.71	0.0776	0.3618	0.83	0.96	0.4916	0.8317	0.55
rs11650313	A	0.85	0.4083	0.9004	0.65	0.82	-0.4557	0.4909	0.35	0.83	-0.2597	0.4348	0.55				
rs11650316	A	0.73	-0.817	0.7078	0.25												
rs11650345	A	0.06	0.5593	1.7572	0.75	0.05	-0.0384	0.9024	0.97	0.05	0.0209	0.811	0.98				
rs11650709	A	0.58	0.7897	0.6536	0.23	0.61	0.5552	0.4048	0.17	0.60	0.6534	0.3496	0.06	0.57	-0.2751	0.3286	0.40

rs11650784	A	0.59	0.8702	0.671	0.19	0.63	0.5181	0.4242	0.22	0.62	0.6287	0.3637	0.08				
rs11651201	A	0.59	-1.6905	0.6143	0.01	0.59	-0.1149	0.3829	0.76	0.59	-0.49	0.33	0.14	0.58	-0.1104	0.315	0.73
rs11651204	A	0.59	-1.1294	0.6164	0.07	0.59	-0.0926	0.3811	0.81	0.59	-0.4396	0.3289	0.18				
rs11651302	A	0.91	1.6451	1.6425	0.32	0.94	-0.1798	0.8323	0.83	0.94	0.2714	0.7574	0.72				
rs11651514	C	0.03	-2.037	2.49	0.41												
rs11651587	A	0.71	0.5387	0.7152	0.45												
rs11651611	A	0.94	0.5593	1.7572	0.75	0.95	-0.178	0.8929	0.84	0.95	-0.0982	0.8041	0.90				
rs11651690	A	0.94	0.5593	1.7572	0.75	0.95	-0.178	0.8929	0.84	0.95	-0.0982	0.8041	0.90				
rs11651707	A	0.22	0.1338	0.7806	0.86												
rs11652075	A	0.26	-0.0157	0.7099	0.98												
rs11652082	A	0.49	-0.0501	0.6571	0.94												
rs11652856	C	0.15	0.2748	0.9341	0.77												
rs11652975	A	0.61	0.6054	0.6227	0.33												
rs11652985	C	0.42	-0.3394	0.6498	0.60												
rs11652993	C	0.35	-0.4509	0.6787	0.51												
rs11653700	A	0.14	0.4216	0.934	0.65	0.16	-0.8884	0.5224	0.09	0.15	-0.5662	0.4656	0.22	0.14	-0.3486	0.4795	0.47
rs11654115	A	0.34	-0.7878	0.6511	0.23												
rs11654261	A	0.78	0.071	0.7886	0.93	0.81	0.0344	0.4842	0.94	0.80	-0.0891	0.4165	0.83				
rs11654508	A	0.31	0.153	0.7315	0.83												
rs11654770	A	0.38	-0.0885	0.6495	0.89	0.36	-0.2251	0.392	0.57	0.37	-0.227	0.3405	0.51				
rs11654881	A	0.27	-0.2624	0.714	0.71												
rs11655167	A	0.76	-1.0912	0.7143	0.13	0.79	0.1106	0.4678	0.81	0.78	-0.193	0.4006	0.63				
rs11655435	C	0.92	1.1525	1.5234	0.45												
rs11655455	A	0.76	0.8279	0.8683	0.34	0.75	-0.7095	0.4787	0.14	0.75	-0.2746	0.4285	0.52				
rs11655650	A	0.44	-0.0774	0.6165	0.90												
rs11656298	A	0.77	0.4129	0.7655	0.59	0.78	0.1158	0.4578	0.80	0.77	0.1185	0.3976	0.77				
rs11656673	A	0.14	-0.9238	1	0.36	0.09	-0.0607	0.7008	0.93	0.10	-0.2891	0.5819	0.62				
rs11656929	A	0.15	-0.4759	1.0074	0.64	0.11	-0.8842	0.6768	0.19	0.12	-0.7047	0.5698	0.22	0.10	0.2298	0.6032	0.70
rs11657098	A	0.24	-0.5466	0.7385	0.46												
rs11657217	C	0.14	-0.356	0.895	0.69												
rs11657360	C	0.48	-0.2389	0.626	0.70												
rs11657440	A	0.87	0.3185	0.9748	0.74	0.87	-0.5084	0.5658	0.37	0.87	-0.2394	0.5033	0.63				
rs11657500	A	0.78	-0.313	0.7605	0.68	0.84	0.6934	0.5053	0.17	0.82	0.3659	0.4231	0.39				
rs11657630	A	0.78	0.5513	0.7433	0.46												

rs11657635	A	0.30	-0.9796	0.6844	0.15								
rs11657655	A	0.24	0.4323	0.7146	0.55								
rs11657933	C	0.31	0.0626	0.8059	0.94	0.31	-0.8804	0.4561	0.05	0.31	-0.5915	0.4053	0.14
rs11658052	A	0.09	-0.6285	1.1641	0.59	0.15	0.3678	0.5441	0.50	0.13	0.141	0.4928	0.77
rs11658299	A	0.61	0.1647	0.658	0.80								
rs11658442	A	0.05	-1.1288	2.1799	0.60	0.04	0.0122	1.0746	0.99	0.04	-0.2254	0.9511	0.81
rs11658622	A	0.49	0.1712	0.6545	0.79								
rs11658680	A	0.37	-0.3096	0.6568	0.64	0.42	0.2092	0.3764	0.58	0.41	0.0047	0.3315	0.99
rs11658702	A	0.71	0.1776	0.6674	0.79	0.72	-0.0109	0.4164	0.98	0.72	-0.0927	0.3576	0.80
rs11701424	A									0.01	-2.0629	3.0525	0.50
rs11720308	A					0.99	0.3919	3.3774	0.91	0.99	-0.0239	2.2742	0.99
rs11775334	A	0.35	0.2517	0.6621	0.70	0.38	-0.2457	0.3796	0.52	0.38	-0.2037	0.3342	0.54
rs11867344	A	0.67	0.7088	0.6473	0.27								
rs11867708	A	0.80	-0.1359	0.8594	0.87								
rs11867785	A	0.21	-0.1978	0.8217	0.81								
rs11868442	A	0.12	0.665	0.9456	0.48								
rs11868471	A	0.29	-0.3888	0.7423	0.60								
rs11869115	A	0.72	-0.775	0.7308	0.29								
rs11869363	A	0.46	1.2737	0.738	0.08								
rs11869453	A	0.08	1.1525	1.5234	0.45								
rs11869620	A	0.75	-0.3885	0.7837	0.62								
rs11869626	A	0.22	0.5574	0.7414	0.45								
rs11869629	A	0.72	-1.3204	0.7086	0.06								
rs11870238	A	0.19	0.2685	0.8127	0.74								
rs11870326	A	0.92	-3.0315	1.3633	0.03	0.92	-0.1594	0.6936	0.82	0.92	-0.86	0.6182	0.16
rs11870711	A	0.25	0.0811	0.7074	0.91	0.24	-0.1547	0.4383	0.72	0.25	-0.0073	0.3784	0.98
rs11870849	A	0.04	-4.3419	2.4083	0.07								
rs11871056	A	0.28	-0.1654	0.6967	0.81								
rs11871318	A	0.28	0.9333	0.7179	0.19	0.28	-0.0772	0.4171	0.85	0.28	0.1432	0.3645	0.69
rs11871435	A	0.07	-1.3831	1.7527	0.43	0.06	-1.4661	0.9406	0.12	0.06	-1.4594	0.8239	0.08
rs11871595	A	0.22	0.6506	0.8198	0.43								
rs11871688	C	0.78	0.5538	0.741	0.45								
rs11891	A	0.05	0.6427	1.4821	0.66								
rs11903	A	0.46	0.5088	0.7043	0.47								

rs12046278	A	0.35	-0.2836	0.6753	0.67	0.33	-0.2457	0.3962	0.54	0.33	-0.3021	0.3466	0.38				
rs12051618	A	0.68	1.0732	0.6651	0.11	0.72	-0.1958	0.4219	0.64	0.71	0.1831	0.3628	0.61				
rs12051619	A	0.32	1.0318	0.6652	0.12	0.28	-0.2705	0.4199	0.52	0.29	0.115	0.3613	0.75	0.27	-0.0286	0.3505	0.94
rs12051621	A					0.94	-0.4843	0.9518	0.61	0.94	0.1934	0.9131	0.83	0.95	-1.1532	0.8839	0.19
rs12051632	A	0.58	-0.1636	0.6387	0.80	0.54	0.3183	0.3803	0.40	0.55	0.1722	0.3317	0.60				
rs12051678	A	0.21	-0.8888	0.7997	0.27												
rs12051723	A	0.05	-1.3555	1.5907	0.39												
rs12149977	A	0.62	0.0859	0.6226	0.89	0.57	0.1393	0.3784	0.71	0.59	0.1136	0.329	0.73				
rs12150348	A	0.19	-1.2147	0.8241	0.14												
rs12325869	C					0.03	2.17	2.0059	0.28	0.03	1.9469	2.0123	0.33				
rs12430	A	0.27	1.0144	0.7737	0.19												
rs12449322	A	0.57	1.4137	0.6366	0.03												
rs12449492	A	0.45	-0.232	0.6215	0.71												
rs12449669	A	0.52	0.3714	0.6284	0.55												
rs12449676	A	0.72	-0.4646	0.73	0.52												
rs12449822	A	0.72	-0.0532	0.6842	0.94	0.74	-0.4027	0.4374	0.36	0.74	-0.3818	0.374	0.31				
rs12449949	A	0.25	1.7274	0.774	0.03	0.22	0.5375	0.4634	0.25	0.23	0.8474	0.4046	0.04				
rs12449970	A	0.38	0.079	0.6879	0.91	0.35	0.1805	0.4042	0.66	0.36	0.3319	0.3512	0.34				
rs12449983	A	0.49	0.2562	0.6141	0.68												
rs12450030	A	0.41	-0.0701	0.7149	0.92	0.34	-0.7299	0.4288	0.09	0.36	-0.5403	0.3725	0.15	0.38	-0.1557	0.3428	0.65
rs12450059	A	0.50	-0.941	0.6411	0.14	0.45	0.0068	0.3817	0.99	0.46	-0.2009	0.3328	0.55	0.47	-0.1313	0.3198	0.68
rs12450128	A	0.55	-0.4581	0.6317	0.47												
rs12450239	A	0.41	-0.8728	0.632	0.17	0.35	0.4008	0.3862	0.30	0.37	0.1368	0.3359	0.68				
rs12450432	A	0.20	-0.8283	0.7894	0.29												
rs12450478	A	0.33	-0.0937	0.6461	0.88												
rs12450588	A	0.04	-1.6062	2.3607	0.50	0.03	-0.5093	1.233	0.68	0.03	-0.6003	1.0158	0.55				
rs12450753	A	0.27	0.2367	0.7344	0.75												
rs12450839	A	0.41	0.1154	0.6245	0.85	0.42	0.1073	0.3792	0.78	0.42	0.0656	0.33	0.84				
rs12450876	A	0.91	0.2711	1.1293	0.81												
rs12450888	C	0.54	1.2621	0.632	0.05												
rs12450989	A	0.20	0.6511	0.7555	0.39												
rs12451047	A	0.41	0.6868	0.626	0.27	0.39	-0.0045	0.3816	0.99	0.40	0.2921	0.331	0.38				
rs12451318	A	0.09	0.3523	1.2195	0.77	0.09	-1.1835	0.6856	0.08	0.09	-0.7448	0.6047	0.22				
rs12451395	A	0.76	-0.3974	0.7567	0.60	0.74	0.2255	0.4437	0.61	0.75	0.0728	0.3875	0.85				

rs12451487	A	0.61	0.0451	0.6189	0.94												
rs12451560	A	0.47	0.4468	0.6309	0.48	0.47	0.3101	0.3785	0.41	0.47	0.246	0.3285	0.45				
rs12451638	A	0.39	0.5166	0.6224	0.41	0.34	0.437	0.3964	0.27	0.35	0.4611	0.3405	0.18				
rs12451668	A	0.73	-0.1336	0.7559	0.86	0.77	0.5171	0.4627	0.26	0.76	0.3596	0.3977	0.37	0.71	0.0883	0.3604	0.81
rs12451808	A	0.28	-0.33	0.716	0.64												
rs12452184	A	0.49	-0.8064	0.6318	0.20												
rs12452616	A	0.24	0.7857	0.7666	0.31	0.21	0.1866	0.4576	0.68	0.22	0.4706	0.3999	0.24				
rs12452652	A	0.26	-0.752	0.7131	0.29												
rs12452661	A	0.77	-0.3243	0.7318	0.66	0.80	-0.0899	0.4696	0.85	0.79	-0.2415	0.4024	0.55				
rs12452891	A	0.15	1.161	1.0364	0.26												
rs12453011	A	0.53	-0.6882	0.632	0.28												
rs12453034	A	0.09	0.2783	1.1282	0.81												
rs12453207	A	0.26	0.3981	0.7204	0.58	0.22	-0.0731	0.4487	0.87	0.23	0.0536	0.3845	0.89	0.26	0.0545	0.3596	0.88
rs12453606	A	0.69	-0.5519	0.694	0.43	0.72	-0.5044	0.4121	0.22	0.71	-0.4411	0.3584	0.22				
rs12453678	A	0.26	0.4948	0.7225	0.49	0.22	-0.0561	0.4523	0.90	0.23	0.1019	0.3876	0.79				
rs12453809	A	0.11	-1.1908	0.9757	0.22												
rs1254787	A	0.08	-1.7393	1.5641	0.27												
rs1254795	A	0.28	-0.2781	0.6963	0.69												
rs12600564	A	0.14	0.4848	0.9208	0.60												
rs12600765	A	0.22	-1.2823	0.7311	0.08	0.27	-0.0374	0.4195	0.93	0.25	-0.5347	0.3689	0.15				
rs12600868	A	0.59	-0.1762	0.6557	0.79												
rs12600908	A	0.04	1.0107	2.1404	0.64												
rs12601162	C	0.26	-0.1796	0.8201	0.83												
rs12601177	A	0.18	-0.5561	0.8946	0.53												
rs12601423	A	0.09	0.0514	1.1084	0.96												
rs12601803	A	0.24	0.5579	0.7391	0.45												
rs12601898	A	0.19	-1.427	0.9183	0.12	0.19	-1.0483	0.5182	0.04	0.19	-1.167	0.4567	0.01	0.19	0.3536	0.4583	0.44
rs12601949	A	0.26	0.5438	0.8366	0.52												
rs12602330	A	0.88	-0.4508	1.3139	0.73	0.91	0.7898	0.71	0.27	0.90	0.814	0.6289	0.20	0.89	-0.3846	0.5872	0.51
rs12602412	A	0.39	-0.2689	0.6421	0.68	0.38	0.2341	0.3895	0.55	0.38	0.1542	0.3382	0.65				
rs12602415	A	0.39	-0.1452	0.6408	0.82	0.38	0.2211	0.3889	0.57	0.38	0.1733	0.3379	0.61				
rs12602618	A	0.56	-0.262	0.616	0.67	0.55	0.4947	0.3841	0.20	0.56	0.2602	0.331	0.43				
rs12602885	A	0.08	0.2913	1.1634	0.80												
rs12603040	A	0.39	0.403	0.6366	0.53	0.39	0.6168	0.387	0.11	0.39	0.459	0.3355	0.17				

rs12603074	A	0.09	0.2494	1.1291	0.83												
rs12603194	A	0.42	1.2607	0.6254	0.04												
rs12603265	A	0.78	-0.104	0.8145	0.90												
rs12603697	A	0.88	5.2878	2.9084	0.07	0.95	0.8078	0.9679	0.40	0.95	1.0554	0.8938	0.24				
rs12603748	A	0.91	2.0257	1.9649	0.30	0.93	1.275	0.831	0.12	0.93	1.6214	0.7701	0.04				
rs12604076	A	0.56	1.0242	0.6235	0.10												
rs12797	A	0.41	0.9325	0.6346	0.14												
rs1285293	A	0.23	-0.645	0.7367	0.38												
rs12935880	A	0.04	1.1931	2.4821	0.63	0.02	-0.6371	1.6814	0.70	0.02	-0.118	1.4004	0.93	0.02	0.2702	1.6609	0.87
rs12936076	A	0.14	-0.6584	0.9551	0.49												
rs12936473	A	0.80	1.3537	0.8013	0.09												
rs12936687	A	0.19	0.2544	0.848	0.76												
rs12937212	A	0.16	-1.0868	0.9471	0.25	0.19	-0.2742	0.492	0.58	0.18	-0.5548	0.437	0.20	0.20	0.2503	0.4012	0.53
rs12937891	A	0.89	0.9799	1.1069	0.38	0.88	-0.6971	0.6071	0.25	0.88	-0.1205	0.5411	0.82				
rs12938262	A	0.35	0.93	0.6511	0.15	0.41	-0.1982	0.3842	0.61	0.40	0.0624	0.3357	0.85				
rs12938422	C	0.36	1.0132	0.6364	0.11	0.43	-0.0708	0.3825	0.85	0.41	0.1655	0.3324	0.62				
rs12938889	A	0.10	-0.9345	1.0305	0.36												
rs12939128	A	0.98	1.1436	3.5397	0.75	0.99	-1.2672	2.2544	0.57	0.99	0.2922	1.6075	0.86				
rs12939413	A	0.09	0.2783	1.1282	0.81												
rs12939525	A	0.84	-2.5501	0.9463	0.01	0.80	-0.1055	0.5137	0.84	0.81	-0.673	0.4609	0.14	0.85	0.3128	0.4998	0.53
rs12939549	A	0.86	-0.6584	0.9551	0.49												
rs12940068	A	0.91	0.3785	1.1139	0.73												
rs12940226	A	0.53	1.0067	0.7552	0.18												
rs12940295	A	0.83	-0.1575	0.9269	0.87	0.84	-0.2298	0.5532	0.68	0.84	-0.3243	0.4821	0.50	0.87	-0.6823	0.5309	0.20
rs12940302	A	0.35	0.891	0.6496	0.17	0.41	-0.1668	0.3823	0.66	0.40	0.0758	0.334	0.82				
rs12940622	A	0.14	-0.6104	0.9693	0.53												
rs12941504	A	0.34	0.3586	0.6861	0.60												
rs12941958	A	0.09	0.2783	1.1282	0.81												
rs12941999	A	0.47	0.8123	0.5883	0.17												
rs12942038	A	0.29	0.9013	0.7178	0.21	0.31	0.372	0.4176	0.37	0.30	0.5477	0.3638	0.13	0.27	0.2073	0.3693	0.57
rs12942476	A	0.82	-0.3058	0.814	0.71	0.81	-0.0535	0.4718	0.91	0.81	-0.1278	0.4121	0.76				
rs12943128	A	0.06	-1.0858	1.8117	0.55												
rs12943136	C	0.51	0.0192	0.606	0.97												
rs12943410	A	0.26	1.4393	0.7525	0.06	0.23	0.3217	0.4592	0.48	0.24	0.64	0.3979	0.11				

rs12943496	C	0.12	1.1199	1.0464	0.28	0.13	-0.1413	0.5789	0.81	0.13	0.1362	0.5173	0.79	0.10	1.1988	0.5487	0.03
rs12943617	A	0.48	0.1503	0.6374	0.81	0.43	0.4434	0.3694	0.23	0.45	0.2432	0.3253	0.45				
rs12943620	A	0.14	0.5013	0.931	0.59												
rs12944002	A	0.64	0.6224	0.6508	0.34	0.60	-0.6052	0.3806	0.11	0.61	-0.3157	0.3338	0.34				
rs12944016	A	0.74	0.6726	0.7029	0.34	0.74	-0.4088	0.4321	0.34	0.74	-0.0179	0.3727	0.96				
rs12944642	A	0.51	0.1142	0.6129	0.85	0.55	0.1457	0.3829	0.70	0.54	0.0867	0.3301	0.79				
rs12944841	C	0.09	-0.966	1.6026	0.55	0.09	-1.589	0.708	0.02	0.09	-1.1565	0.6355	0.07				
rs12944983	A	0.87	0.2172	0.9343	0.82												
rs12945231	A	0.06	-2.2396	1.5891	0.16												
rs12945425	A	0.98	1.1311	3.5441	0.75	0.99	-0.9255	2.2509	0.68	0.99	0.4303	1.6062	0.79				
rs12945469	A	0.92	-0.4648	1.4192	0.74												
rs12945787	A	0.12	2.2087	1.065	0.04	0.11	0.3467	0.6272	0.58	0.11	0.6998	0.5534	0.21				
rs12945963	A	0.51	0.2069	0.6098	0.73	0.50	-0.5949	0.3734	0.11	0.51	-0.3307	0.3233	0.31				
rs12946115	A	0.91	0.2783	1.1282	0.81												
rs12946426	A	0.41	-1.4058	0.6345	0.03	0.42	-0.308	0.381	0.42	0.41	-0.5071	0.3329	0.13	0.43	-0.533	0.3159	0.09
rs12946454	A	0.88	-1.0526	1.1087	0.34	0.82	-0.2481	0.5149	0.63	0.84	-0.3604	0.4737	0.45				
rs12946859	A	0.15	-0.2048	1.1537	0.86	0.11	0.2941	0.6763	0.66	0.11	0.2715	0.5946	0.65				
rs12947653	A	0.91	0.5416	1.141	0.64												
rs12947901	A	0.91	0.405	1.1447	0.72												
rs12948040	A	0.91	0.2783	1.1282	0.81												
rs12948969	A	0.09	1.6451	1.6425	0.32	0.06	-0.1798	0.8323	0.83	0.06	0.2714	0.7574	0.72				
rs12949063	A	0.90	0.632	1.0678	0.55												
rs12949118	A	0.60	0.2684	0.6283	0.67	0.59	0.6811	0.3809	0.07	0.59	0.4487	0.3307	0.17				
rs12950039	A	0.35	0.3844	0.6482	0.55												
rs12950541	A	0.09	0.2783	1.1282	0.81												
rs12950551	A	0.09	1.6451	1.6425	0.32	0.06	-0.1414	0.8346	0.87	0.06	0.3078	0.7591	0.69				
rs12950642	A	0.69	0.7329	0.6572	0.26												
rs12950752	A	0.10	0.7197	1.2123	0.55												
rs12951389	A	0.43	-1.5191	0.6279	0.02	0.46	0.0821	0.3721	0.83	0.45	-0.2638	0.3264	0.42	0.46	-0.0249	0.3169	0.94
rs12951541	A	0.47	0.4186	0.6256	0.50												
rs12951778	A	0.66	0.5647	0.6796	0.41												
rs12952105	A	0.25	1.1691	0.7477	0.12	0.26	-0.2064	0.439	0.64	0.25	0.2174	0.3824	0.57				
rs12952612	A	0.49	-1.4908	0.6166	0.02	0.50	-0.0916	0.3683	0.80	0.50	-0.5089	0.3213	0.11				
rs12952770	A	0.59	1.3262	0.608	0.03												

rs1317421	A	0.84	-0.735	1.1455	0.52	0.85	0.0457	0.5824	0.94	0.85	-0.1439	0.5226	0.78				
rs1318314	A	0.81	-0.1931	0.8743	0.83	0.78	-0.5814	0.477	0.22	0.79	-0.5195	0.4234	0.22				
rs1318315	A	0.24	-1.2563	0.7147	0.08	0.20	0.1019	0.4726	0.83	0.21	-0.2337	0.4034	0.56				
rs13342272	A	0.80	1.2343	0.7682	0.11	0.79	0.0583	0.4632	0.90	0.79	0.2268	0.3995	0.57				
rs1369299	A	0.09	-0.1118	1.4094	0.94												
rs1378905	A	0.50	-0.888	0.6415	0.17	0.44	-0.0404	0.38	0.92	0.46	-0.2295	0.3316	0.49	0.46	-0.1499	0.3191	0.64
rs1378906	A	0.65	-0.8665	0.6573	0.19	0.56	-0.5601	0.3812	0.14	0.59	-0.5591	0.333	0.09				
rs1378942	A	0.24	-1.249	0.8587	0.15	0.22	-0.8856	0.4757	0.06	0.23	-0.9192	0.427	0.03				
rs1384367	A	0.44	1.0974	0.6224	0.08												
rs1388512	A	0.72	0.0036	0.7263	1.00												
rs1436138	A	0.56	-0.9641	0.6394	0.13												
rs14640	A	0.88	1.6673	0.976	0.09												
rs1465983	A	0.27	1.9117	0.7189	0.01												
rs1466003	A	0.25	-0.993	0.7352	0.18												
rs1466681	A	0.90	-0.9345	1.0305	0.36												
rs1467979	A	0.07	-1.6513	1.3052	0.21	0.08	-0.4673	0.7302	0.52	0.07	-0.7329	0.6378	0.25				
rs1468030	A	0.77	0.3718	0.7312	0.61												
rs1468032	A	0.25	0.1598	0.7043	0.82												
rs1468033	A	0.65	-0.7681	0.6505	0.24												
rs1468035	A	0.54	0.0523	0.6327	0.93												
rs1470941	A	0.76	0.531	0.7706	0.49												
rs1473312	A	0.32	0.3542	0.6516	0.59												
rs1476788	A	0.24	0.5075	0.7738	0.51												
rs1485329	A	0.88	-0.3605	1.0415	0.73												
rs1485330	A	0.08	-0.4826	1.2081	0.69												
rs1530440	A	0.28	0.9505	0.6742	0.16	0.26	0.1001	0.4188	0.81	0.26	0.3888	0.3619	0.28				
rs1533570	A	0.47	-0.6882	0.632	0.28												
rs1546407	A	0.81	0.0442	0.7998	0.96												
rs1550186	A	0.86	0.2735	0.9054	0.76	0.84	-0.0672	0.5162	0.90	0.84	-0.0362	0.453	0.94				
rs1551619	A	0.18	-0.7074	0.8127	0.38												
rs1551858	A	0.25	0.755	0.7181	0.29												
rs1552173	A	0.45	0.4585	0.628	0.47												
rs15538	A	0.46	-0.4208	0.6762	0.53	0.49	0.1836	0.4003	0.65	0.48	0.0135	0.3507	0.97				
rs1561810	A	0.07	-1.8422	1.329	0.17	0.08	-0.6719	0.7345	0.36	0.07	-0.8906	0.6456	0.17				

rs1561811	A	0.44	0.3065	0.6146	0.62	0.41	0.0771	0.3821	0.84	0.42	0.201	0.3308	0.54				
rs1563447	A	0.46	0.8471	0.635	0.18	0.45	0.4488	0.3674	0.22	0.45	0.4782	0.3231	0.14				
rs1563448	A	0.12	-0.9214	1.4127	0.51	0.07	0.2132	0.8141	0.79	0.08	0.1356	0.6873	0.84	0.08	1.1861	0.6436	0.07
rs1563449	A	0.92	4.0494	1.9665	0.04	0.95	-0.348	0.9288	0.71	0.95	0.5848	0.8413	0.49				
rs1564868	A	0.18	0.0257	0.8842	0.98												
rs1567960	C	0.75	0.3038	0.742	0.68												
rs1567962	A	0.28	-0.2657	0.7003	0.70												
rs1568448	C	0.33	-0.2479	0.7696	0.75												
rs1629034	A	0.80	1.4101	0.7855	0.07												
rs1631707	A	0.17	-0.1661	0.9026	0.85	0.14	-0.691	0.5856	0.24	0.15	-0.3591	0.4967	0.47				
rs1632673	A	0.18	-0.0115	0.9074	0.99	0.14	-0.6793	0.5822	0.24	0.15	-0.3124	0.495	0.53				
rs164009	A	0.47	0.7591	0.6307	0.23												
rs164106	A	0.54	0.7117	0.6291	0.26												
rs1661714	A	0.27	0.3562	0.7596	0.64												
rs1661721	A	0.27	0.3983	0.7647	0.60												
rs1663196	A	0.26	-0.3509	0.7175	0.62	0.26	0.1848	0.4281	0.67	0.26	-0.0274	0.3718	0.94				
rs1663199	A	0.18	-0.0058	0.9037	0.99	0.14	-0.6162	0.5876	0.29	0.15	-0.2752	0.4983	0.58				
rs1671019	A	0.27	-0.1857	0.7046	0.79												
rs1671021	A	0.73	-0.1997	0.7012	0.78												
rs1671032	A	0.76	-0.0904	0.7231	0.90												
rs1671033	C	0.71	-0.9024	0.6721	0.18												
rs1671036	A	0.29	0.3587	0.7174	0.62												
rs1675262	A	0.82	-0.2785	0.7892	0.72	0.85	0.4282	0.5295	0.42	0.84	0.2061	0.4501	0.65				
rs16948048	A	0.88	0.5463	1.1005	0.62	0.86	0.1786	0.5493	0.75	0.86	0.2819	0.5028	0.58				
rs1696754	A	0.18	-0.0115	0.9074	0.99	0.14	-0.5966	0.5882	0.31	0.15	-0.2531	0.4988	0.61				
rs16967764	A	0.02	-4.5675	3.181	0.15												
rs16967789	A	0.05	0.469	2.0786	0.82												
rs16968129	C	0.35	0.5375	0.6422	0.40												
rs16968692	A	0.74	-1.1596	0.7489	0.12												
rs16968702	A	0.26	-1.1596	0.7489	0.12												
rs16968960	A	0.63	0.3265	0.6591	0.62												
rs16969138	A	0.89	-0.498	1.0631	0.64												
rs16969262	A	0.83	-0.5048	0.8822	0.57												
rs16969510	A	0.35	-0.4545	0.6612	0.49												

rs16970576	A	0.83	-1.043	0.8809	0.24	0.84	-0.0943	0.5204	0.86	0.84	-0.3502	0.4557	0.44
rs16970774	A	0.19	-0.2328	0.9201	0.80								
rs16970784	A	0.21	-0.6633	0.8295	0.42								
rs16970787	A	0.79	-0.8395	0.7968	0.29								
rs16970792	A	0.35	-0.5752	0.6946	0.41								
rs16970802	A	0.19	-0.0869	0.975	0.93								
rs16970803	A	0.81	-1.2147	0.8241	0.14								
rs16970811	A	0.81	-0.0869	0.975	0.93								
rs16971269	C	0.89	-1.8429	1.1699	0.12								
rs16971526	A	0.86	-2.2248	0.9653	0.02								
rs16971682	A	0.59	1.1514	0.6391	0.07								
rs16972227	A	0.70	-0.4579	0.6439	0.48	0.72	0.0104	0.4114	0.98	0.72	-0.1478	0.352	0.67
rs16978176	A	0.59	0.7741	0.6373	0.22								
rs16978193	A	0.80	-0.0444	0.8449	0.96								
rs16978197	A	0.19	-0.2113	0.8629	0.81								
rs16978198	A	0.80	-0.0645	0.8563	0.94								
rs16982520	A	0.98	-4.0103	3.2089	0.21	0.96	2.8058	1.1851	0.02	0.97	2.1328	1.0383	0.04
rs1699607	A	0.36	0.5957	0.6388	0.35								
rs1708861	A	0.18	2.0533	0.8284	0.01	0.20	0.2773	0.4664	0.55	0.20	0.5661	0.4115	0.17
rs17248007	A	0.88	0.5769	1.0123	0.57	0.90	-0.3374	0.6157	0.58	0.89	-0.1991	0.5293	0.71
rs17248371	A	0.03	2.6742	2.5295	0.29	0.03	-0.4307	1.3565	0.75	0.03	0.3913	1.1825	0.74
rs17249754	A	0.07	-2.3474	1.2752	0.07	0.06	-1.0999	0.865	0.20	0.06	-1.4541	0.7276	0.05
rs17367504	A	0.85	1.45	0.9927	0.14	0.89	-0.342	0.5993	0.57	0.89	-0.0249	0.5184	0.96
rs17545956	A	0.25	0.5955	0.7563	0.43	0.23	-0.344	0.4624	0.46	0.24	-0.0972	0.4003	0.81
rs17553512	A	0.33	0.6251	0.6411	0.33								
rs17557592	C	0.75	-0.4536	0.7741	0.56								
rs17561950	A	0.59	0.2489	0.6219	0.69								
rs17581498	A	0.93	-0.6761	1.233	0.58								
rs17614280	A	0.39	1.4442	0.6299	0.02								
rs17632208	A	0.86	-0.4428	1.047	0.67	0.87	0.0965	0.5693	0.87	0.87	0.0411	0.5017	0.93
rs17656528	A	0.88	1.2744	1.0533	0.23								
rs17657522	A	0.41	1.168	0.6246	0.06								
rs17657767	A	0.44	1.1036	0.6224	0.08								
rs17736494	A	0.44	1.0974	0.6224	0.08								

rs17739056	A	0.57	-0.5298	0.6308	0.40												
rs17742707	A	0.62	1.1492	0.664	0.08	0.65	0.03	0.4104	0.94	0.64	0.3437	0.3531	0.33				
rs1800299	A					0.02	-2.7102	2.5098	0.28	0.01	-1.1574	2.4872	0.64				
rs1800303	A	0.03	-0.0599	2.7805	0.98	0.03	-0.0603	1.3433	0.96	0.03	0.1689	1.1954	0.89				
rs1800304	A	0.36	0.0281	0.6436	0.97	0.37	-0.0974	0.3776	0.80	0.37	-0.0108	0.3324	0.97				
rs1800305	A	0.03	-0.0413	2.7748	0.99	0.03	-0.0681	1.3425	0.96	0.03	0.1686	1.1944	0.89				
rs1800307	A	0.04	-2.1064	2.2658	0.35	0.02	-0.0379	1.3751	0.98	0.02	-0.1701	1.1389	0.88				
rs1800310	A	0.90	-1.6785	1.1189	0.13	0.89	-0.3811	0.5927	0.52	0.90	-0.5598	0.528	0.29				
rs1800315	A					0.01	-1.7014	3.4442	0.62	0.01	-1.8904	3.4443	0.58				
rs1868821	A	0.32	-0.2743	0.6536	0.67												
rs1869932	A	0.72	-0.4972	0.6928	0.47	0.71	-0.4087	0.4055	0.31	0.72	-0.3691	0.3544	0.30				
rs1869934	A	0.38	0.1131	0.6475	0.86	0.32	-0.3167	0.4037	0.43	0.34	-0.1515	0.3468	0.66				
rs1870625	A	0.32	0.6346	0.654	0.33	0.35	-0.8667	0.3898	0.03	0.34	-0.4259	0.3438	0.22				
rs1870990	A	0.22	0.4196	0.7656	0.58												
rs1871935	A	0.52	-0.2368	0.6266	0.71												
rs1873588	A	0.34	-0.1783	0.77	0.82												
rs1877677	A	0.67	-1.0505	0.6735	0.12	0.69	0.1709	0.414	0.68	0.68	-0.1758	0.3588	0.62				
rs1877926	A	0.24	0.3979	0.7149	0.58												
rs1878061	A	0.33	-0.8188	0.6425	0.20												
rs1879455	C	0.44	1.3334	0.6355	0.04												
rs1879967	A	0.20	0.2675	0.8385	0.75												
rs1879968	A	0.33	0.5454	0.6421	0.40												
rs1905160	A	0.04	2.5117	1.8108	0.17												
rs1918974	A	0.85	1.142	0.9034	0.21	0.82	-0.2763	0.4924	0.57	0.83	0.0941	0.4409	0.83				
rs1963982	A	0.57	0.3258	0.6031	0.59	0.56	-0.5678	0.3723	0.13	0.56	-0.3656	0.3211	0.26				
rs1965780	A	0.53	-0.3143	0.6364	0.62	0.53	0.5047	0.3761	0.18	0.53	0.2235	0.3292	0.50				
rs1976492	A	0.32	0.3378	0.6651	0.61												
rs1979370	A	0.97	-0.7981	2.5291	0.75												
rs1983283	A					0.02	2.1599	2.4717	0.38	0.02	2.0253	2.4806	0.41				
rs1993681	C	0.75	0.5973	0.7283	0.41	0.69	-0.1209	0.4211	0.77	0.70	0.0766	0.3729	0.84				
rs1997978	A	0.46	0.6084	0.6031	0.31												
rs2002573	A	0.26	-1.449	0.752	0.05												
rs2003241	A	0.97	1.1439	2.0136	0.57												
rs2004381	A	0.64	-0.7646	0.655	0.24	0.57	-0.2715	0.3836	0.48	0.59	-0.418	0.3376	0.22	0.62	-0.5471	0.333	0.10

rs2004885	A									0.01	-0.9244	2.0461	0.65
rs2004887	A	0.94	-2.6513	1.8896	0.16	0.94	-0.4989	0.8673	0.57	0.94	-1.0338	0.7665	0.18
rs2009196	C	0.85	1.2699	0.8859	0.15								
rs2013350	A	0.75	-0.9039	0.7159	0.21	0.80	0.1553	0.4693	0.74	0.78	-0.1173	0.4018	0.77
rs2014989	A	0.21	0.2565	0.7385	0.73	0.19	-0.3154	0.4791	0.51	0.19	-0.1766	0.4102	0.67
rs2016126	A	0.44	1.369	0.6322	0.03								
rs2028551	A	0.20	0.1974	0.819	0.81								
rs2033715	A	0.52	-0.178	0.6455	0.78	0.53	-0.0576	0.3699	0.88	0.52	0.1442	0.3245	0.66
rs2034310	A	0.29	-0.3766	0.7138	0.60								
rs2035181	C	0.25	0.1773	0.699	0.80								
rs2044102	A	0.62	0.4776	0.6414	0.46								
rs2044103	A	0.48	-1.8432	0.6197	0.00								
rs2048058	A	0.24	0.4946	0.8726	0.57								
rs2048753	A	0.07	0.7076	1.4185	0.62								
rs2053156	A	0.91	-0.2289	1.3874	0.87								
rs2053159	A	0.10	0.7197	1.2123	0.55								
rs2053160	A	0.10	0.8335	1.2373	0.50								
rs2053508	A	0.53	-0.437	0.627	0.49								
rs2053748	A	0.44	1.3311	0.6312	0.03								
rs2056439	C	0.82	-0.1264	0.8244	0.88								
rs2060097	A					0.97	-0.0051	1.2168	1.00	0.98	-0.0577	1.2139	0.96
rs2063785	A	0.91	-0.2225	1.5191	0.88								
rs2066964	C	0.71	0.1727	0.6968	0.80								
rs2069528	A	0.81	0.3786	0.7927	0.63								
rs2069534	A	0.39	0.4967	0.6538	0.45								
rs2069536	A	0.17	0.8829	0.8784	0.31								
rs2070871	A	0.20	0.3228	0.7448	0.66								
rs2071148	A	0.40	0.3632	0.6269	0.56								
rs2071192	A	0.33	0.5752	0.6725	0.39								
rs2071195	A	0.40	0.3167	0.6475	0.62								
rs2071214	A	0.85	0.3712	1.064	0.73								
rs2071654	A	0.25	0.286	0.7979	0.72								
rs2073285	A	0.54	0.4661	0.6026	0.44								
rs2076949	A	0.87	-0.9515	1.065	0.37	0.87	-0.3857	0.6198	0.53	0.87	-0.6025	0.5442	0.27

rs2077948	A	0.28	-0.2781	0.6963	0.69								
rs2085351	C	0.82	0.4247	0.8098	0.60	0.78	0.1108	0.4528	0.81	0.79	0.2304	0.401	0.57
rs2090204	A	0.05	0.2448	1.6584	0.88								
rs2090205	A	0.12	1.2512	1.5029	0.41								
rs2100896	A	0.64	0.4634	0.6431	0.47								
rs2120886	A	0.20	-0.4326	0.8172	0.60								
rs2121070	A	0.08	0.5328	1.5689	0.73	0.08	0.3435	0.7359	0.64	0.08	0.3747	0.6691	0.58
rs2124603	A	0.58	-1.3441	0.6283	0.03	0.58	-0.2367	0.3725	0.53	0.58	-0.4161	0.3273	0.20
rs2124604	A	0.55	-0.4957	0.6309	0.43	0.57	0.7734	0.377	0.04	0.56	0.3693	0.3294	0.26
rs2124606	A	0.76	-0.898	0.7144	0.21	0.80	0.0472	0.4688	0.92	0.78	-0.1837	0.4005	0.65
rs2125345	A	0.60	-0.2547	0.6583	0.70								
rs2131049	A	0.50	-0.0434	0.629	0.95								
rs2138125	A	0.86	0.2281	1.013	0.82								
rs2138126	A	0.86	0.0063	1.0188	1.00								
rs2139461	A	0.28	-0.243	0.6992	0.73								
rs2165994	A	0.78	-0.2787	0.7439	0.71	0.78	-0.8409	0.4588	0.07	0.78	-0.6478	0.3965	0.10
rs2166703	A	0.44	1.4155	0.6375	0.03								
rs2240769	A	0.50	-1.7048	0.6056	0.00								
rs2241886	A	0.24	0.724	0.7778	0.35								
rs2242462	C	0.33	0.6251	0.6411	0.33								
rs2243538	A	0.38	-0.3686	0.6552	0.57								
rs2244377	A	0.33	0.5752	0.6725	0.39								
rs2246632	A	0.62	-0.356	0.6548	0.59								
rs2247007	A	0.38	-0.356	0.6548	0.59								
rs2255166	A	0.24	-2.1407	0.7518	0.00								
rs2256881	A	0.06	-1.0879	1.3824	0.43	0.07	-0.5515	0.7441	0.46	0.07	-0.6149	0.6483	0.34
rs2257020	A	0.37	-0.3493	0.7221	0.63								
rs2269374	A	0.60	0.1892	0.6193	0.76								
rs2271090	A	0.73	-1.2366	0.7129	0.08								
rs2271602	A	0.24	0.4323	0.7146	0.55								
rs2277698	A	0.12	0.243	0.9714	0.80								
rs2277700	A	0.85	0.8658	0.8861	0.33								
rs2278826	A	0.68	-0.7008	0.6519	0.28								
rs2279052	C	0.72	1.014	0.6771	0.13								

rs2279053	A	0.72	1.014	0.6771	0.13												
rs2279056	A	0.52	0.8422	0.6571	0.20												
rs2279308	A	0.53	0.4787	0.6215	0.44												
rs2279914	A	0.02	-9.6021	3.4155	0.00	0.05	0.133	1.0909	0.90	0.04	-0.5113	0.9443	0.59	0.05	1.2753	0.8707	0.14
rs2279920	A	0.02	-9.6021	3.4155	0.00	0.04	0.2786	0.9989	0.78	0.04	-0.1184	0.9047	0.90	0.05	1.3384	0.8681	0.12
rs2280147	A	0.51	-2.5288	1.6909	0.13												
rs2280177	A	0.05	-0.4418	1.6153	0.78												
rs2280269	A	0.28	-0.2781	0.6963	0.69												
rs2280271	A	0.04	2.4636	2.4509	0.31												
rs2286586	A	0.52	-2.1335	0.6271	0.00												
rs2286593	A	0.28	-1.3919	0.715	0.05												
rs2289527	C	0.82	-0.0946	0.9241	0.92	0.87	0.5494	0.6032	0.36	0.85	0.3657	0.5105	0.47				
rs2289529	A	0.08	-0.1704	1.3399	0.90	0.09	-0.1936	0.7202	0.79	0.09	-0.2074	0.646	0.75				
rs2289531	A	0.03	0.3018	2.7772	0.91	0.03	-0.6962	1.305	0.59	0.03	0.1864	1.0733	0.86				
rs2289535	A	0.76	0.0863	0.7322	0.91												
rs2289536	A					0.01	-1.3356	3.1614	0.67	0.01	-0.8666	2.8625	0.76				
rs2289603	A	0.26	-1.6473	0.7781	0.03												
rs2289606	A	0.74	-1.1596	0.7489	0.12												
rs2289728	A	0.23	-0.071	0.749	0.92												
rs2289751	C	0.47	-0.8041	0.6144	0.19												
rs2289759	A	0.79	0.0648	0.7782	0.93												
rs2289762	A	0.22	0.2004	0.7768	0.80												
rs2290251	A	0.64	0.4439	0.6699	0.51												
rs2290455	A	0.13	0.2656	0.9552	0.78												
rs2290460	A	0.34	-0.0324	0.6573	0.96												
rs2290566	A	0.02	0.1223	3.1073	0.97	0.03	1.6894	1.2975	0.19	0.03	1.337	1.203	0.27	0.04	-0.4611	0.9495	0.63
rs2290769	C	0.88	1.5376	0.9502	0.11												
rs2290771	A	0.71	-0.0683	0.6665	0.92												
rs2290906	A	0.64	-0.4772	0.6925	0.49												
rs2290907	A	0.63	-0.4843	0.7058	0.49												
rs2291021	A	0.51	-1.9979	1.1314	0.08												
rs2291028	A	0.44	0.8862	0.6636	0.18												
rs2291029	A	0.98	-4.5675	3.181	0.15												
rs2291359	A	0.72	-0.1183	0.7171	0.87												

rs2292184	A	0.73	-1.4387	0.7323	0.05												
rs2292642	A	0.62	0.4651	0.6368	0.47												
rs2292645	A	0.88	0.8474	1.4853	0.57												
rs2293189	A	0.76	-0.1735	0.7479	0.82												
rs2293192	A	0.75	-0.0865	0.7434	0.91												
rs2304830	A	0.49	-1.494	0.6185	0.02												
rs2304852	A	0.87	-0.8932	0.9585	0.35	0.84	-0.8174	0.5392	0.13	0.85	-0.8577	0.4805	0.07				
rs2304854	A	0.87	-0.9194	0.9614	0.34	0.84	-0.7498	0.5442	0.17	0.85	-0.8036	0.4844	0.10				
rs2304855	A	0.47	-1.1614	0.6364	0.07												
rs2304856	A	0.72	-0.1701	0.6949	0.81												
rs2304921	A	0.08	-1.3393	1.3149	0.31												
rs2305214	A	0.41	0.9282	0.6348	0.14												
rs2305345	A	0.24	-0.3019	0.7236	0.68												
rs2305346	A	0.24	-0.4009	0.7294	0.58												
rs2305912	A	0.76	0.4484	0.805	0.58												
rs2305913	A	0.60	0.1986	0.6508	0.76												
rs2305936	A	0.62	-0.1966	0.704	0.78												
rs2306219	A	0.94	0.7404	1.2221	0.54												
rs2306690	A	0.79	-0.1103	0.8222	0.89												
rs2307008	A	0.11	-1.0094	0.9823	0.30												
rs2307010	A	0.05	1.7202	1.539	0.26												
rs2311001	C	0.54	-0.1105	0.6789	0.87												
rs2311442	A	0.81	-0.2328	0.9201	0.80												
rs2311443	C	0.19	-0.2096	0.9217	0.82												
rs2315921	A	0.93	0.5325	1.3725	0.70												
rs2316058	C	0.79	-0.1978	0.8217	0.81												
rs2333988	A	0.25	0.6698	0.7494	0.37												
rs2361701	A	0.07	-1.6511	1.3064	0.21	0.07	-0.6539	0.7332	0.37	0.07	-0.8653	0.6402	0.18				
rs2361710	A	0.53	-0.5134	0.6321	0.42												
rs2362384	A	0.95	2.5625	1.6213	0.11	0.96	-0.4761	0.9906	0.63	0.96	0.3712	0.8629	0.67	0.95	0.9659	0.8967	0.28
rs2376999	A	0.97	0.6159	1.8295	0.74												
rs2377000	A	0.47	0.4186	0.6256	0.50												
rs2377003	A	0.03	2.1449	2.0977	0.31												
rs2377309	A	0.27	-1.0854	0.7612	0.15	0.22	0.0257	0.4659	0.96	0.23	-0.2525	0.4036	0.53				

rs2377394	A	0.92	4.0494	1.9665	0.04	0.94	0.5561	0.8876	0.53	0.94	1.0992	0.8184	0.18				
rs2377397	A	0.64	-1.4008	0.6554	0.03	0.65	0.2833	0.3988	0.48	0.65	-0.0979	0.3433	0.78				
rs2377400	A	0.14	0.7455	1.0627	0.48	0.17	-1.5775	0.5636	0.01	0.16	-0.9543	0.5083	0.06				
rs2377404	A	0.66	-0.7597	0.6752	0.26	0.70	0.0909	0.4161	0.83	0.69	-0.1838	0.3603	0.61				
rs2384550	A	0.24	0.7662	0.7686	0.32	0.21	0.0344	0.4993	0.95	0.22	0.151	0.4271	0.72				
rs2385067	A	0.58	-1.1331	0.6385	0.08												
rs2385264	A	0.91	-0.2289	1.3874	0.87												
rs2398162	A	0.63	-1.0458	0.6372	0.10	0.57	-0.092	0.3812	0.81	0.59	-0.0047	0.3319	0.99				
rs2410427	A	0.69	-1.2688	0.6786	0.06	0.70	-0.3575	0.4099	0.38	0.70	-0.6814	0.3569	0.06				
rs2411109	A	0.53	0.4704	0.628	0.45												
rs2411122	A	0.23	-1.3163	0.7404	0.08												
rs2411131	A	0.31	-0.8901	0.6652	0.18	0.33	-0.3812	0.4011	0.34	0.32	-0.5116	0.3487	0.14				
rs2411137	A	0.53	0.5185	0.6113	0.40	0.55	-0.4493	0.3712	0.23	0.54	-0.255	0.3212	0.43				
rs2435974	A	0.92	-2.0341	1.2262	0.10	0.89	1.1701	0.6016	0.05	0.90	0.5311	0.5429	0.33				
rs2456582	A	0.95	-2.2598	1.4949	0.13	0.94	-0.1123	0.7723	0.88	0.94	-0.5017	0.6804	0.46	0.93	0.4924	0.6678	0.46
rs2457692	A	0.38	-0.4188	0.6577	0.52												
rs2466511	A	0.95	-0.8976	1.6054	0.58	0.93	0.5775	0.788	0.46	0.94	0.1586	0.7067	0.82				
rs2509458	A	0.92	-3.5971	1.7565	0.04	0.95	-0.9474	1.0576	0.37	0.95	-1.6803	0.8676	0.05				
rs2515815	A	0.55	1.5763	0.6107	0.01												
rs2567494	A	0.41	-0.5919	0.6331	0.35	0.44	0.3886	0.3879	0.32	0.43	-0.3868	0.3354	0.25				
rs2574852	A	0.18	0.2284	0.8549	0.79												
rs2584100	A	0.48	0.1015	0.6347	0.87												
rs2589118	A	0.34	-0.1894	0.6339	0.77												
rs2589119	A	0.04	3.4079	2.1801	0.12												
rs2589143	A	0.28	0.8375	0.6988	0.23												
rs2589148	A	0.04	3.4079	2.1801	0.12												
rs2589150	A	0.96	3.4079	2.1801	0.12												
rs2589153	A	0.03	4.9763	2.554	0.05												
rs2589155	A	0.96	3.4079	2.1801	0.12												
rs2589157	A	0.04	4.385	2.2717	0.05												
rs2598414	A	0.59	-0.212	0.6336	0.74												
rs2598420	C	0.45	0.2972	0.6318	0.64												
rs2598435	A	0.40	-0.2425	0.6389	0.70												
rs2598444	A	0.40	-0.2425	0.6389	0.70												

rs2598450	A	0.41	-0.1086	0.6401	0.87												
rs2606183	A	0.70	0.5718	0.6807	0.40	0.76	0.2791	0.4352	0.52	0.74	0.29	0.3729	0.44				
rs2606188	A	0.70	0.6057	0.6909	0.38	0.76	0.1491	0.4403	0.73	0.75	0.2062	0.3776	0.59				
rs2608880	A	0.40	0.2626	0.6515	0.69												
rs2608882	A	0.29	0.7233	0.7393	0.33												
rs2612753	A	0.41	0.4353	0.6297	0.49	0.43	0.1784	0.372	0.63	0.42	0.1801	0.3267	0.58				
rs2612771	A	0.62	-0.0275	0.6593	0.97	0.58	-0.2556	0.3827	0.50	0.59	-0.2699	0.3353	0.42				
rs2612773	A	0.36	-0.3307	0.6389	0.60	0.37	0.2889	0.3922	0.46	0.37	0.0987	0.3413	0.77				
rs2612782	A	0.18	-0.3073	0.8139	0.71	0.17	0.2762	0.4994	0.58	0.17	0.0894	0.4342	0.84				
rs2612788	A	0.70	0.6886	0.662	0.30	0.67	-0.5649	0.3995	0.16	0.68	-0.1581	0.3508	0.65				
rs2613514	A	0.77	-0.0476	0.9275	0.96												
rs2613516	A	0.10	-1.3133	1.1509	0.25												
rs2659003	C	0.06	-0.8104	1.3712	0.55	0.07	-0.3952	0.751	0.60	0.07	-0.4306	0.6508	0.51				
rs2659028	A	0.02	-4.9876	3.2975	0.13	0.01	0.5008	2.8429	0.86	0.01	-1.9536	1.9108	0.31	0.01	-0.2377	2.128	0.91
rs2659029	A	0.07	-0.6526	1.3125	0.62	0.08	-0.7053	0.688	0.31	0.08	-0.6141	0.6041	0.31				
rs2659030	A	0.77	-2.3117	0.7902	0.00	0.71	-0.5155	0.4352	0.24	0.73	-0.9704	0.3886	0.01	0.76	0.4553	0.3967	0.25
rs2661686	A	0.42	0.6269	0.6378	0.33												
rs2661694	A	0.16	-1.0814	0.8831	0.22												
rs2665972	A	0.40	-0.2425	0.6389	0.70												
rs2665983	C	0.38	-0.3857	0.6534	0.56												
rs2665993	A	0.49	0.1604	0.6479	0.80												
rs2665998	A	0.60	-0.2279	0.638	0.72												
rs2666011	A	0.38	-0.356	0.6548	0.59												
rs2670827	A	0.75	-0.2463	0.7268	0.73												
rs2672886	A	0.81	0.7383	0.8084	0.36												
rs2672890	A	0.57	-0.6078	0.6202	0.33												
rs2672893	A	0.11	1.9381	1.2192	0.11												
rs2678770	A	0.46	-1.0818	0.616	0.08												
rs2681472	A	0.93	-2.4461	1.2848	0.06	0.94	-1.0139	0.8665	0.24	0.94	-1.4348	0.7302	0.05				
rs2681492	A	0.93	-2.3474	1.2752	0.07	0.94	-1.0972	0.8663	0.21	0.94	-1.4521	0.7283	0.05				
rs2707031	C	0.41	0.1676	0.633	0.79	0.39	0.3899	0.387	0.31	0.39	0.4221	0.3342	0.21				
rs2713991	A	0.33	0.6194	0.6925	0.37	0.32	0.2123	0.4063	0.60	0.32	0.3691	0.3544	0.30				
rs2714011	A	0.66	-0.52	0.6517	0.42	0.62	-0.1616	0.3942	0.68	0.63	-0.2307	0.3404	0.50				
rs2725391	A	0.65	-0.8625	0.6571	0.19	0.60	0.2268	0.376	0.55	0.62	-0.0496	0.3328	0.88	0.64	0.179	0.3241	0.58

rs2820037	A	0.08	0.7269	1.2979	0.58	0.07	-0.2579	0.7276	0.72	0.07	-0.0602	0.6395	0.92				
rs2854701	A	0.34	-0.8702	0.657	0.19												
rs2854704	A	0.71	-0.8965	0.75	0.23												
rs28607597	A	0.74	1.2703	0.7474	0.09	0.76	0.5389	0.4497	0.23	0.76	0.7225	0.3927	0.07	0.74	0.0672	0.3701	0.86
rs2889619	A	0.70	0.4805	0.6816	0.48	0.74	-0.209	0.4341	0.63	0.72	0.0263	0.371	0.94				
rs2889620	A	0.42	0.8157	0.6554	0.21	0.39	0.368	0.4078	0.37	0.39	0.5224	0.352	0.14	0.43	-0.2263	0.3292	0.49
rs2889622	A	0.78	0.1692	0.7263	0.82	0.77	0.1706	0.4452	0.70	0.77	0.211	0.3842	0.58				
rs2889645	A	0.05	-1.5972	2.1382	0.46	0.03	-1.6104	1.0482	0.12	0.04	-1.9702	0.9332	0.03				
rs2891607	A	0.81	0.1716	0.8423	0.84												
rs2891713	C	0.09	-0.5767	1.4117	0.68												
rs2891714	A	0.90	0.7197	1.2123	0.55												
rs2898569	A	0.35	0.3879	0.6505	0.55												
rs2898577	A	0.25	0.3511	0.7417	0.64												
rs296139	A	0.42	-0.5521	0.6457	0.39	0.45	-0.2604	0.3804	0.49	0.44	-0.4539	0.331	0.17				
rs3087664	A	0.59	-0.0134	0.6595	0.98												
rs3096277	A	0.30	1.2913	0.6707	0.05	0.28	0.4105	0.4182	0.33	0.29	0.5516	0.3599	0.13				
rs312828	A	0.44	0.8337	0.671	0.21												
rs312834	A	0.55	0.8791	0.6728	0.19												
rs312853	A	0.86	-0.5975	0.8812	0.50												
rs312858	A	0.31	0.0558	0.6531	0.93												
rs312863	A	0.38	0.115	0.6164	0.85												
rs312884	A	0.23	-1.6942	0.7465	0.02												
rs312895	A	0.90	0.0709	1.1988	0.95												
rs312896	C	0.91	0.4042	1.2479	0.75												
rs3178300	A	0.73	-0.8057	0.8721	0.36												
rs3184504	A	0.20	-0.6804	1.0358	0.51	0.14	-0.535	0.573	0.35	0.15	-0.5944	0.5052	0.24				
rs3185057	A	0.02	-3.757	3.0004	0.21												
rs3186520	A	0.13	0.7753	0.9261	0.40												
rs3208785	C	0.68	-0.0857	0.6559	0.90												
rs3208787	A	0.90	0.2076	1.0382	0.84												
rs34237952	C	0.91	2.2663	1.1621	0.05	0.89	-0.1665	0.6278	0.79	0.90	0.3127	0.5601	0.58				
rs34262564	A	0.18	-0.2559	0.8119	0.75	0.21	0.1525	0.4716	0.75	0.20	0.0018	0.4108	1.00				
rs3432	A	0.18	-0.344	0.8925	0.70												
rs34520486	A	0.76	0.3238	0.728	0.66	0.75	0.2537	0.4284	0.55	0.75	0.2899	0.3769	0.44				

rs34547368	A	0.30	-0.8409	0.6856	0.22	0.33	-0.4123	0.4004	0.30	0.33	-0.4158	0.3504	0.24				
rs34664664	A	0.12	0.0039	0.9753	1.00	0.15	-0.1551	0.5231	0.77	0.15	-0.085	0.4662	0.86	0.14	-0.2419	0.4701	0.61
rs346789	A	0.70	-0.3119	0.7555	0.68												
rs346801	A	0.16	0.2324	0.8768	0.79												
rs34768269	A	0.92	-1.0125	1.1441	0.38	0.91	-0.2387	0.6231	0.70	0.91	-0.4221	0.5503	0.44	0.91	-1.2286	0.5629	0.03
rs34939985	A	0.09	1.6451	1.6425	0.32	0.07	-0.1796	0.8203	0.83	0.06	0.0744	0.752	0.92				
rs35110805	C	0.59	-1.0883	0.6254	0.08	0.57	-0.1929	0.3707	0.60	0.57	-0.3279	0.325	0.31	0.58	-0.2163	0.317	0.50
rs35496088	A	0.84	0.1697	0.8771	0.85	0.83	-0.6129	0.487	0.21	0.83	-0.3352	0.432	0.44				
rs35578653	A	0.69	0.6396	0.6514	0.33	0.68	0.3206	0.396	0.42	0.68	0.4237	0.3454	0.22				
rs35863760	A	0.73	-0.7234	0.7424	0.33	0.72	0.1375	0.4218	0.74	0.72	-0.0816	0.3693	0.83				
rs35926122	A	0.88	-6.548	3.1748	0.04	0.96	-1.3106	1.0503	0.21	0.96	-0.6223	0.9509	0.51				
rs36013558	A	0.02	1.1436	3.5397	0.75	0.01	-0.6516	2.3057	0.78	0.01	0.6332	1.6259	0.70				
rs363796	A	0.41	-0.2299	0.6457	0.72												
rs3643	A	0.91	-1.8968	1.2262	0.12												
rs372558	C	0.62	0.3964	0.6416	0.54												
rs3744026	A	0.91	-1.311	1.1302	0.25												
rs3744032	A	0.03	-0.2462	1.8276	0.89												
rs3744036	A	0.25	-0.6177	0.7615	0.42												
rs3744037	A	0.77	1.743	0.7553	0.02												
rs3744043	A	0.07	0.0964	1.2353	0.94												
rs3744054	A	0.16	-0.7504	0.8825	0.40												
rs3744166	A	0.11	0.5683	1.0217	0.58												
rs3744171	A	0.05	0.5153	1.4572	0.72												
rs3744173	A	0.43	-0.5324	0.6355	0.40												
rs3744181	A	0.44	-0.448	0.6366	0.48	0.43	0.3008	0.3808	0.43	0.43	0.0822	0.3331	0.81				
rs3744182	A	0.04	-3.4972	2.1703	0.11	0.03	-0.7079	1.2746	0.58	0.03	-1.4734	1.1172	0.19				
rs3744186	A	0.46	-0.7341	0.6198	0.24	0.46	0.452	0.3748	0.23	0.46	0.1168	0.3261	0.72				
rs3744189	A	0.15	-0.6869	1.242	0.58												
rs3744198	A	0.22	0.1106	0.7522	0.88												
rs3744203	A	0.49	-0.429	0.627	0.49												
rs3744204	A	0.46	-0.2502	0.625	0.69												
rs3744215	A	0.20	-0.3378	0.8842	0.70												
rs3744216	A	0.92	-1.8582	1.4444	0.20												
rs3744793	A	0.46	0.4103	0.6401	0.52												

rs3751932	A	0.89	0.2186	1.4302	0.88								
rs3751934	A	0.57	0.2771	0.6387	0.66								
rs3751947	A	0.07	-1.8776	1.7458	0.28	0.06	-2.1787	0.9055	0.02	0.06	-1.9944	0.8009	0.01
rs3751955	A	0.48	-1.4538	0.6132	0.02								
rs3751956	A	0.14	-0.0337	0.9442	0.97								
rs3751957	A	0.40	0.0804	0.6525	0.90								
rs3751962	A	0.81	-0.0869	0.975	0.93								
rs3754777	A	0.34	-0.1906	0.6583	0.77	0.33	0.0663	0.397	0.87	0.33	-0.0424	0.3445	0.90
rs3764377	A	0.81	-0.0869	0.975	0.93								
rs3764438	A	0.06	2.3414	1.8969	0.22	0.04	-0.3681	0.9888	0.71	0.05	-0.0915	0.8673	0.92
rs3764440	A	0.91	-0.6005	1.2995	0.64	0.90	-0.1477	0.6949	0.83	0.90	-0.2515	0.624	0.69
rs3765123	A	0.08	-0.7245	1.1114	0.51								
rs3785446	A	0.10	0.8892	1.5628	0.57								
rs3785529	A	0.43	-0.878	0.6278	0.16								
rs379465	A	0.16	-0.7119	0.9047	0.43								
rs3803737	A	0.28	1.0208	0.6776	0.13								
rs3803739	A	0.48	-0.6616	0.6495	0.31								
rs3803742	A	0.23	0.0927	0.9508	0.92								
rs3803767	A	0.05	-3.9401	1.9176	0.04								
rs3803780	A					0.99	-1.7841	1.9553	0.36	0.99	-1.9697	1.9289	0.31
rs3803783	A	0.80	-0.1879	0.8592	0.83								
rs3803786	C	0.96	-0.7938	2.2156	0.72								
rs3803792	A	0.37	0.8906	0.6385	0.16								
rs3809691	C	0.37	0.4551	0.6547	0.49								
rs3809713	A	0.72	0.5534	0.7036	0.43	0.70	0.4532	0.4053	0.26	0.71	0.432	0.3568	0.23
rs3813063	A	0.26	-0.0618	0.7073	0.93								
rs3816427	A	0.74	-1.1596	0.7489	0.12								
rs3817292	C	0.24	0.4323	0.7146	0.55								
rs381815	A	0.37	0.8174	0.6646	0.22	0.33	0.3296	0.4112	0.42	0.34	0.4895	0.3551	0.17
rs3826274	A	0.27	1.1367	0.7814	0.15								
rs3826314	A	0.20	-0.1542	0.8801	0.86								
rs3826552	A	0.44	1.0208	0.6217	0.10								
rs3829574	A	0.09	-0.9399	1.1313	0.41								
rs3829611	A	0.30	-0.4667	0.6725	0.49								

rs3829612	A	0.23	0.4338	0.7459	0.56								
rs3851021	A	0.15	-0.1923	0.8538	0.82								
rs385689	A	0.20	1.8163	0.9415	0.05								
rs3869467	A	0.41	0.9713	0.6516	0.14								
rs387601	A	0.50	-0.3949	0.6167	0.52								
rs387774	A	0.84	-0.744	0.9006	0.41								
rs388090	A	0.74	-1.45	0.7546	0.05								
rs3889146	A	0.72	-0.8497	0.7297	0.24								
rs3893391	C	0.89	-0.5498	0.9898	0.58								
rs3924327	A	0.42	-0.5253	1.5364	0.73								
rs3934492	C	0.80	1.5506	0.77	0.04								
rs3934967	A	0.68	0.4613	0.6631	0.49	0.72	0.3515	0.422	0.40	0.71	0.4166	0.3597	0.25
rs3935192	A	0.15	0.6887	0.8689	0.43								
rs3935543	A	0.24	-0.5233	0.7829	0.50								
rs3935648	C	0.78	-0.3025	0.7441	0.68								
rs3935674	C	0.66	-0.408	0.6808	0.55								
rs3936118	A	0.52	-0.2389	0.626	0.70								
rs3936523	A	0.48	-0.153	0.6236	0.81								
rs397481	A	0.10	-1.3965	1.2676	0.27								
rs402273	A	0.25	1.575	0.7511	0.04								
rs403483	A	0.16	-0.6904	0.902	0.44								
rs4073111	C	0.50	0.2997	0.6271	0.63								
rs4073996	A	0.03	-1.6656	1.9548	0.39	0.04	-0.3309	0.9655	0.73	0.04	-0.8288	0.8649	0.34
rs4073997	C	0.58	-0.1755	0.7089	0.80								
rs4074022	C	0.39	-0.2467	0.6259	0.69	0.36	-0.4027	0.3902	0.30	0.37	-0.3478	0.3378	0.30
rs4074023	A	0.67	0.0054	0.6511	0.99	0.68	-0.1448	0.3961	0.71	0.68	-0.0635	0.3456	0.85
rs4074469	A	0.22	-0.0201	0.7354	0.98	0.22	0.6995	0.4555	0.12	0.22	0.5664	0.3944	0.15
rs4075482	A	0.25	-0.3604	0.6978	0.61								
rs4076427	C	0.25	-0.1262	0.6883	0.85								
rs4076967	C	0.79	0.63	0.7732	0.42								
rs4077126	A	0.43	-0.4795	0.6149	0.44								
rs4077240	A	0.30	0.5595	0.6773	0.41								
rs4077719	A	0.91	1.7447	1.5519	0.26								
rs4078259	A	0.33	0.5585	0.6883	0.42								

rs4078429	A	0.96	-2.0051	2.3612	0.40												
rs4078474	C	0.68	0.5428	0.687	0.43												
rs4082919	A	0.62	0.618	0.6403	0.33												
rs4103047	C	0.48	-0.153	0.6236	0.81												
rs4129767	A	0.59	0.1357	0.6231	0.83												
rs4239025	A	0.58	0.7468	0.655	0.25	0.61	0.3651	0.4064	0.37	0.61	0.5038	0.3509	0.15	0.57	-0.2114	0.3294	0.52
rs4239026	C	0.42	1.0044	0.653	0.12	0.38	0.3727	0.4082	0.36	0.39	0.5886	0.3522	0.09	0.43	-0.1803	0.33	0.58
rs4243249	A	0.23	-0.9492	0.7358	0.20	0.19	0.2999	0.4801	0.53	0.20	-0.0111	0.406	0.98				
rs4243251	A	0.78	0.2493	0.7672	0.75												
rs4255830	A	0.17	0.1354	0.8639	0.88												
rs4295	C	0.22	0.0034	0.8005	1.00	0.19	0.1668	0.4913	0.73	0.20	0.0624	0.4253	0.88				
rs4305	A	0.23	-0.4836	0.7788	0.53	0.21	0.0612	0.4698	0.90	0.22	-0.1308	0.408	0.75				
rs4309	A	0.77	-0.2295	0.7589	0.76	0.77	-0.1058	0.46	0.82	0.77	-0.188	0.3996	0.64				
rs4318268	A	0.28	-0.2634	0.7422	0.72												
rs4331	A	0.31	-0.0709	0.6645	0.92	0.29	0.1791	0.4079	0.66	0.30	0.1439	0.3532	0.68				
rs4333	A	0.31	-0.0793	0.6665	0.91	0.28	0.2954	0.4103	0.47	0.29	0.2293	0.3549	0.52				
rs4335	A	0.70	-0.0873	0.6722	0.90	0.72	0.3077	0.4094	0.45	0.71	0.2491	0.3553	0.48				
rs4341	C	0.31	-0.1248	0.6665	0.85	0.29	0.1786	0.4077	0.66	0.30	0.1314	0.3533	0.71				
rs4344	A	0.69	-0.1303	0.6675	0.85	0.71	0.2157	0.4076	0.60	0.70	0.1534	0.3534	0.66				
rs4350602	A	0.88	0.1925	1.1008	0.86												
rs4351	A	0.69	0.0304	0.6665	0.96	0.71	0.3017	0.4093	0.46	0.71	0.2616	0.3543	0.46				
rs4352096	A	0.57	0.7504	0.6445	0.24												
rs4353	A	0.31	-0.0431	0.6702	0.95	0.29	0.2885	0.4072	0.48	0.30	0.2325	0.3534	0.51				
rs4362	A	0.31	0.043	0.6711	0.95	0.29	0.3195	0.4131	0.44	0.29	0.2885	0.3574	0.42				
rs4363	A	0.69	0.1166	0.6711	0.86	0.71	0.2159	0.4077	0.60	0.71	0.2398	0.354	0.50				
rs4365	A					0.01	1.0703	2.0515	0.60	0.01	1.8373	1.7757	0.30				
rs4370013	A	0.54	-0.0759	0.6116	0.90	0.55	0.0172	0.3794	0.96	0.55	-0.1552	0.3267	0.63				
rs4375697	A	0.61	0.7231	0.6169	0.24												
rs4411562	A	0.22	-0.0798	0.9042	0.93												
rs4424945	A	0.22	-1.6458	0.7566	0.03												
rs443970	A	0.49	0.6236	0.6466	0.33												
rs4441315	A	0.27	0.8338	0.7591	0.27	0.27	-0.0385	0.443	0.93	0.26	0.2032	0.3876	0.60				
rs4444373	C	0.92	3.9671	1.8934	0.04	0.93	0.6142	0.8382	0.46	0.93	1.1128	0.7761	0.15				
rs445507	A	0.84	-0.0004	0.9613	1.00												

rs445683	A	0.52	-0.6196	0.6373	0.33								
rs4459614	A	0.22	1.2594	0.7341	0.09								
rs4462668	A	0.50	-0.3568	0.6287	0.57	0.53	-0.5506	0.3793	0.15	0.52	-0.5514	0.3298	0.09
rs4465632	A	0.75	0.2674	0.7377	0.72								
rs448203	A	0.17	-0.1317	0.9014	0.88								
rs4482327	A	0.77	0.0005	0.7222	1.00	0.76	0.2756	0.4348	0.53	0.76	0.2371	0.3772	0.53
rs448378	A	0.83	0.9438	0.877	0.28	0.82	-0.3173	0.4868	0.51	0.82	0.0286	0.4336	0.95
rs4485403	A	0.91	-0.4262	1.3005	0.74	0.92	-0.5048	0.7256	0.49	0.92	-0.4965	0.6467	0.44
rs4490057	A	0.41	0.3395	0.6389	0.60								
rs4491585	A	0.18	-0.4868	0.9419	0.61								
rs4491586	A	0.62	0.0902	0.6678	0.89								
rs4505373	A	0.47	0.1394	0.6503	0.83								
rs4516263	A	0.62	0.4956	0.6427	0.44								
rs4523953	A	0.62	0.364	0.6442	0.57								
rs4531782	A	0.68	0.4954	0.6618	0.45								
rs4533317	A	0.84	0.0288	0.9349	0.98	0.87	0.8868	0.6084	0.15	0.86	0.6394	0.5136	0.21
rs4538044	A	0.13	-1.7646	0.928	0.06								
rs454138	C	0.27	-0.346	0.6994	0.62								
rs4542691	A	0.11	0.6164	1.1997	0.61								
rs454845	A	0.16	-0.744	0.9006	0.41								
rs4558471	A	0.86	0.1318	0.8944	0.88								
rs4564643	A	0.19	-0.0869	0.975	0.93								
rs4592695	A	0.22	0.0479	0.7269	0.95	0.24	0.2654	0.4325	0.54	0.24	0.2287	0.3762	0.54
rs4622540	A	0.48	-0.5341	0.6205	0.39	0.51	0.1737	0.3787	0.65	0.50	-0.0457	0.3268	0.89
rs4624215	A	0.90	0.7197	1.2123	0.55								
rs4788837	A	0.76	-0.2034	0.7308	0.78								
rs4788840	A	0.86	0.1512	1.0508	0.89								
rs4788887	C	0.11	0.6164	1.1997	0.61								
rs4788889	A	0.09	-0.5767	1.4117	0.68								
rs4788890	A	0.09	-0.5767	1.4117	0.68								
rs4788891	A	0.09	-0.6193	1.413	0.66								
rs4788893	A	0.32	-0.38	0.7011	0.59								
rs4788931	A	0.87	-1.1881	1.0456	0.26								
rs4789012	A	0.42	0.38	0.6523	0.56								

rs4789084	A	0.67	0.3192	0.647	0.62								
rs4789096	C	0.44	1.3621	0.6345	0.03								
rs4789102	A	0.19	-1.6794	0.8003	0.04								
rs4789114	A	0.31	0.4624	0.6901	0.50								
rs4789145	A	0.46	1.2935	0.6337	0.04								
rs4789170	A	0.10	0.7197	1.2123	0.55								
rs4789172	A	0.36	1.4168	0.6404	0.03								
rs4789173	A	0.09	-0.1121	1.4106	0.94								
rs4789176	A	0.09	-0.1118	1.4094	0.94								
rs4789178	C	0.91	-0.2289	1.3874	0.87								
rs4789181	A	0.09	-0.2289	1.3874	0.87								
rs4789182	A	0.88	0.1185	1.0913	0.91								
rs4789183	A	0.91	-0.2289	1.3874	0.87								
rs4789186	A	0.91	-0.5767	1.4117	0.68								
rs4789188	A	0.09	-0.6323	1.4125	0.65								
rs4789213	C	0.29	0.3587	0.7174	0.62								
rs4789251	A	0.53	0.1026	0.6498	0.87								
rs4789274	A	0.28	1.0326	0.679	0.13								
rs4789291	A	0.46	-0.626	0.6401	0.33								
rs4789302	A	0.71	-0.644	0.6897	0.35								
rs4789366	A	0.71	-0.1535	0.7214	0.83								
rs4789378	A	0.30	-1.1089	0.7139	0.12								
rs4789380	A	0.14	0.6854	0.9445	0.47								
rs4789418	C	0.13	-0.7386	0.9139	0.42								
rs4789462	A	0.42	0.2465	0.6034	0.68	0.41	0.214	0.3774	0.57	0.41	0.3321	0.3247	0.31
rs4789531	A	0.65	-0.5752	0.6946	0.41								
rs4789533	C	0.81	-1.2147	0.8241	0.14								
rs4789559	A	0.44	-0.0774	0.6165	0.90								
rs4789649	A	0.25	0.7121	0.7163	0.32								
rs4789671	A	0.77	1.1305	0.7329	0.12								
rs4789763	A	0.63	0.6989	0.6709	0.30								
rs4789853	C	0.58	-0.3307	0.6847	0.63	0.57	0.3856	0.3958	0.33	0.57	0.2296	0.3486	0.51
rs4789860	A	0.56	0.0118	0.6037	0.98								
rs4789874	A	0.38	0.0385	0.6339	0.95	0.32	-0.3258	0.4029	0.42	0.34	-0.1749	0.3449	0.61

rs4789875	A	0.74	0.3599	0.7194	0.62	0.72	-0.0059	0.425	0.99	0.73	0.1309	0.3718	0.72	0.72	0.0224	0.359	0.95
rs4789878	C	0.82	-1.3726	0.8359	0.10	0.83	-0.2442	0.4989	0.62	0.83	-0.6545	0.4324	0.13	0.82	0.5818	0.4212	0.17
rs4789879	A	0.20	0.0237	0.8216	0.98	0.22	0.2839	0.4599	0.54	0.21	0.2218	0.4073	0.59				
rs4789883	A	0.97	-0.0148	1.8513	0.99	0.95	0.4531	0.9565	0.64	0.95	0.7152	0.8479	0.40				
rs4789885	A	0.45	-0.1791	0.6126	0.77	0.48	0.1302	0.3758	0.73	0.47	0.0181	0.3244	0.96				
rs4789887	A	0.22	-0.2609	0.7221	0.72	0.27	0.679	0.427	0.11	0.25	0.3462	0.3762	0.36				
rs4789888	A	0.28	0.0218	0.6764	0.97	0.26	0.081	0.4215	0.85	0.27	0.0757	0.3636	0.84				
rs4789890	A	0.88	0.1202	0.9281	0.90	0.83	0.5081	0.5058	0.32	0.84	0.3105	0.4519	0.49	0.85	-0.689	0.4473	0.12
rs4789892	A	0.16	0.5251	0.9754	0.59	0.13	-0.0329	0.5888	0.96	0.14	0.1526	0.5111	0.77				
rs4789893	C	0.38	0.4265	0.6615	0.52	0.41	0.0026	0.3927	0.99	0.40	-0.0367	0.3437	0.92				
rs4789904	A	0.43	-0.5313	0.6315	0.40												
rs4789911	A	0.47	-0.446	0.6324	0.48	0.46	0.417	0.3914	0.29	0.46	0.1841	0.3401	0.59				
rs4789939	A	0.07	-0.1507	1.4304	0.92												
rs4789940	A	0.85	0.9344	0.8859	0.29												
rs4789949	A	0.58	1.1206	0.6284	0.07	0.57	-0.4515	0.3795	0.23	0.57	0.0552	0.3301	0.87				
rs4789951	A	0.32	-0.1976	0.6671	0.77	0.39	-0.2941	0.3811	0.44	0.37	-0.3161	0.3348	0.35				
rs4789962	A	0.75	0.048	0.7273	0.95	0.73	0.0044	0.431	0.99	0.74	0.063	0.3765	0.87				
rs4789963	A	0.64	0.1113	0.6438	0.86	0.68	-0.6322	0.4029	0.12	0.67	-0.3661	0.3464	0.29				
rs4789964	C	0.36	0.1102	0.6471	0.86	0.32	-0.4562	0.3988	0.25	0.33	-0.241	0.3447	0.48				
rs4789965	A	0.26	0.3596	0.7213	0.62	0.27	-0.0032	0.4278	0.99	0.26	0.1488	0.3745	0.69	0.27	-0.0234	0.3629	0.95
rs4789966	A	0.38	0.0385	0.6339	0.95	0.32	-0.2823	0.4031	0.48	0.34	-0.1435	0.3451	0.68				
rs4789967	C	0.07	-0.9926	1.3423	0.46	0.07	-0.394	0.7481	0.60	0.07	-0.5606	0.6635	0.40				
rs4789968	A					0.08	-0.0025	0.8854	1.00	0.07	0.4188	0.818	0.61				
rs4789976	A	0.16	-1.1213	0.947	0.24	0.19	-0.2863	0.4916	0.56	0.18	-0.5689	0.4367	0.19				
rs4789977	A	0.61	0.3343	0.6788	0.62	0.67	0.0455	0.4129	0.91	0.65	0.066	0.3571	0.85	0.65	0.4095	0.3362	0.22
rs4789978	A	0.27	0.4692	0.7426	0.53	0.23	0.7299	0.4906	0.14	0.24	0.6997	0.4143	0.09				
rs4789979	A	0.50	0.6164	0.6071	0.31	0.52	-0.1461	0.3763	0.70	0.51	0.1323	0.3264	0.69	0.52	-0.3761	0.3158	0.23
rs4789980	C	0.57	-0.7705	0.6101	0.21	0.57	-0.1105	0.3775	0.77	0.57	-0.3264	0.3248	0.32				
rs4789981	A	0.26	0.2845	0.7216	0.69												
rs4789986	A	0.58	0.9237	0.6615	0.16												
rs4789994	A	0.51	1.284	0.6272	0.04	0.51	0.4779	0.3693	0.20	0.51	0.661	0.3239	0.04				
rs4789997	A	0.56	0.8027	0.6385	0.21	0.58	0.4169	0.3734	0.26	0.57	0.427	0.3279	0.19				
rs4789998	A	0.55	0.9625	0.6381	0.13	0.58	0.4806	0.3751	0.20	0.57	0.5177	0.3291	0.12				
rs4789999	A	0.23	-0.4078	0.7484	0.59	0.28	-0.3891	0.4266	0.36	0.26	-0.294	0.3784	0.44				

rs4790000	A	0.14	0.7915	1.0581	0.45	0.19	-0.7152	0.5399	0.19	0.17	-0.3034	0.4892	0.54				
rs4790001	A	0.44	0.9236	0.6198	0.14	0.48	0.0579	0.3811	0.88	0.47	0.3265	0.3308	0.32				
rs4790002	A	0.35	1.0064	0.6503	0.12	0.43	-0.0571	0.3842	0.88	0.41	0.1783	0.3354	0.60				
rs4790004	A	0.98	0.121	3.1161	0.97	0.97	1.0769	1.1707	0.36	0.97	0.7752	1.0793	0.47	0.96	-0.2425	0.938	0.80
rs4790005	A	0.37	1.1875	0.6701	0.08	0.42	-0.2085	0.3958	0.60	0.41	0.1099	0.3444	0.75				
rs4790007	A	0.50	-0.0845	0.6168	0.89	0.48	0.4648	0.3718	0.21	0.49	0.2113	0.3227	0.51				
rs4790009	A	0.38	0.676	0.658	0.30	0.38	0.5254	0.393	0.18	0.38	0.4767	0.3417	0.16				
rs4790013	A	0.51	0.2634	0.6061	0.66	0.50	-0.6377	0.3727	0.09	0.50	-0.351	0.3225	0.28				
rs4790015	A	0.25	-0.0013	0.7159	1.00												
rs4790018	A	0.72	-0.5515	0.6938	0.43	0.75	0.0555	0.4381	0.90	0.74	-0.1432	0.377	0.70	0.76	0.2944	0.3696	0.43
rs4790019	A	0.22	-0.8846	0.7353	0.23												
rs4790024	A	0.63	-1.3231	0.6842	0.05	0.62	0.0045	0.4024	0.99	0.62	-0.1092	0.3512	0.76				
rs4790032	A	0.65	0.5422	0.6484	0.40	0.64	0.1214	0.3911	0.76	0.65	0.3041	0.3408	0.37				
rs4790036	C	0.03	1.8851	2.8636	0.51	0.01	2.2861	1.9409	0.24	0.02	2.3644	1.6151	0.14				
rs4790048	A	0.16	-0.0102	0.8445	0.99	0.22	1.2749	0.4656	0.01	0.21	0.9162	0.4097	0.03				
rs4793297	A	0.08	0.515	1.2828	0.69	0.11	-0.5483	0.6153	0.37	0.10	-0.2855	0.5581	0.61				
rs4793316	A	0.43	-0.0418	0.6439	0.95	0.46	0.5384	0.3779	0.15	0.46	0.4611	0.3308	0.16				
rs4796817	A	0.11	-0.1222	1.2301	0.92												
rs4889782	A	0.83	-0.1382	0.8556	0.87												
rs4889784	A	0.25	0.1131	0.784	0.89												
rs4889787	A	0.58	0.0641	0.636	0.92												
rs4889809	A	0.28	0.5316	0.6989	0.45	0.22	0.1159	0.4444	0.79	0.24	0.2367	0.3779	0.53				
rs4889810	A	0.16	0.7007	0.8578	0.41	0.18	-0.8477	0.4917	0.08	0.18	-0.4743	0.4365	0.28				
rs4889811	A	0.23	-0.7886	0.7511	0.29	0.18	0.183	0.4797	0.70	0.20	-0.0654	0.4067	0.87				
rs4889815	A	0.12	-0.2049	1.0246	0.84	0.08	-0.4014	0.7096	0.57	0.09	-0.3994	0.5851	0.49				
rs4889839	A	0.37	0.0451	0.6424	0.94												
rs4889852	A	0.13	-0.2606	0.9512	0.78												
rs4889872	A	0.78	-0.0914	0.8167	0.91												
rs4889875	A	0.80	-0.3813	0.8341	0.65												
rs4889882	A	0.79	-0.1704	0.8236	0.84												
rs4889938	A	0.33	1.0213	0.6545	0.12	0.26	0.1189	0.4207	0.78	0.28	0.361	0.3568	0.31				
rs4889940	A	0.34	0.2004	0.673	0.77	0.39	0.0706	0.3852	0.85	0.38	0.0709	0.34	0.83				
rs4889944	A	0.78	-0.8796	0.7536	0.24	0.82	0.1569	0.4842	0.75	0.81	-0.1187	0.4098	0.77				
rs4889951	A	0.29	0.454	0.8962	0.61												

rs4889954	A	0.50	-1.3931	0.6137	0.02	0.48	-0.1482	0.3683	0.69	0.49	-0.5036	0.3213	0.12
rs4889961	C	0.50	-1.4414	0.6191	0.02	0.50	-0.1305	0.367	0.72	0.50	-0.5257	0.3211	0.10
rs4889968	A	0.80	1.1813	0.9159	0.20								
rs4889990	A	0.29	-0.8954	0.733	0.22								
rs4889995	A	0.26	-0.0618	0.7073	0.93								
rs4889996	A	0.74	0.0166	0.7023	0.98								
rs4889998	A	0.60	0.3321	0.6257	0.60								
rs4890010	A	0.23	0.2333	0.7254	0.75								
rs4890012	C	0.76	0.1883	0.7251	0.80								
rs4890026	A	0.15	-0.465	0.8695	0.59								
rs493430	A	0.40	0.3197	0.6074	0.60								
rs4969147	A	0.32	0.6704	0.6465	0.30								
rs4969168	A	0.27	0.0109	0.7243	0.99								
rs4969178	A	0.38	0.5108	0.6353	0.42								
rs4969186	C	0.38	0.4651	0.6368	0.47								
rs4969187	A	0.37	0.2644	0.6447	0.68								
rs4969189	A	0.83	-0.5978	0.7895	0.45								
rs4969219	A	0.68	0.51	0.6918	0.46								
rs4969227	A	0.27	-1.0482	0.7065	0.14								
rs4969230	A	0.25	0.0148	0.7465	0.98								
rs4969235	A	0.25	0.0148	0.7465	0.98								
rs4969245	A	0.77	-0.5303	0.7121	0.46								
rs4969282	A	0.08	0.9537	1.7126	0.58								
rs4969301	A	0.38	0.5108	0.6703	0.45								
rs4969303	A	0.13	0.2669	0.9447	0.78								
rs4969310	A	0.13	0.2646	0.943	0.78								
rs4969311	C	0.63	0.3797	0.6396	0.55								
rs4969322	C	0.38	0.0607	0.6513	0.93								
rs4969331	A	0.66	-0.0754	0.6475	0.91								
rs4969355	A	0.18	-0.3243	0.8049	0.69								
rs4969358	A	0.43	-0.1693	0.6218	0.79								
rs4969364	A	0.43	-0.5249	0.6148	0.39								
rs4969381	A	0.77	-0.5476	0.7101	0.44								
rs4969382	A	0.51	-0.4488	0.6708	0.50								

rs4969384	A	0.13	0.0829	0.9839	0.93								
rs4969385	A	0.50	-0.4417	0.6506	0.50								
rs4969387	C	0.80	-0.3219	0.8314	0.70								
rs4969429	A	0.19	-0.3412	0.7749	0.66								
rs4969441	A	0.83	-0.7978	0.9501	0.40								
rs509574	A	0.31	-0.6812	0.6509	0.30								
rs509911	A	0.82	-0.546	0.9068	0.55								
rs521750	A	0.59	-0.7738	0.6199	0.21								
rs524536	A	0.91	-0.3146	1.217	0.80								
rs545652	A	0.11	-1.7688	1.4796	0.23								
rs546371	A	0.67	0.454	0.6551	0.49								
rs553781	A	0.40	0.229	0.62	0.71								
rs554012	A	0.61	0.491	0.6339	0.44								
rs55714027	A	0.46	0.3375	0.6376	0.60	0.50	-0.0696	0.38	0.85	0.49	0.0653	0.3311	0.84
rs56107536	A	0.46	-0.3859	0.6656	0.56	0.43	-0.0903	0.4095	0.83	0.44	-0.1563	0.3557	0.66
rs56259513	A	0.25	0.5215	0.7092	0.46	0.26	-0.1862	0.4245	0.66	0.26	0.0176	0.369	0.96
rs56353542	A					0.96	-0.0009	1.2498	1.00	0.97	-0.0055	1.1858	1.00
rs56407805	A	0.91	-1.5593	1.1089	0.16	0.90	-0.7219	0.6368	0.26	0.90	-0.9752	0.5617	0.08
rs567009	A	0.63	0.2683	0.6584	0.68								
rs579238	A	0.31	-0.6981	0.6499	0.28								
rs581157	A	0.66	0.3595	0.6427	0.58								
rs59652033	A	0.69	0.2705	0.7302	0.71	0.70	-0.3277	0.4297	0.45	0.70	-0.1575	0.3756	0.68
rs60582626	A	0.45	-0.8909	0.6117	0.15	0.46	-0.8964	0.3791	0.02	0.45	-0.8656	0.3277	0.01
rs60684213	A	0.04	0.1602	2.4961	0.95	0.03	0.2581	1.1039	0.82	0.03	0.0599	0.9935	0.95
rs613075	A	0.38	0.5175	0.6446	0.42								
rs61729127	A					0.99	4.1459	2.1065	0.05	0.99	3.2652	2.0891	0.12
rs61740509	A	0.06	1.3602	1.5205	0.37	0.06	-0.5828	0.818	0.48	0.06	-0.0041	0.729	1.00
rs61756761	A	0.98	-4.9615	3.1105	0.11	0.97	0.4366	1.3647	0.75	0.97	-0.4018	1.2687	0.75
rs62063818	A					0.04	-0.0072	1.2492	1.00	0.03	-0.0079	1.186	0.99
rs62063824	A					0.04	0.0687	1.2727	0.96	0.03	0.4312	1.231	0.73
rs62063831	A	0.74	0.5774	0.7089	0.42	0.73	0.4932	0.4237	0.24	0.73	0.4769	0.3701	0.20
rs62074551	A	0.59	0.247	0.6425	0.70	0.55	-0.011	0.3709	0.98	0.56	0.1035	0.3253	0.75
rs631730	C	0.27	0.0421	0.7037	0.95								
rs633672	A	0.42	-0.3232	0.6254	0.61								

rs6416847	A	0.33	0.4086	0.6909	0.55												
rs6420480	A	0.87	0.5534	0.9837	0.57												
rs6420481	A	0.54	0.0523	0.6327	0.93												
rs6420487	A	0.46	0.0668	0.6081	0.91												
rs647727	A	0.88	-1.8496	0.9635	0.05												
rs6495122	A	0.73	-1.3458	0.7505	0.07	0.75	-0.4509	0.4328	0.30	0.75	-0.6263	0.3837	0.10				
rs6501243	A	0.47	0.5189	0.6256	0.41												
rs6501265	A	0.52	-0.1315	0.6164	0.83												
rs6501267	A	0.58	0.4919	0.6288	0.43												
rs6501285	A	0.25	0.5044	0.7062	0.48	0.29	-0.6717	0.4208	0.11	0.28	-0.3087	0.3687	0.40				
rs6501297	A	0.49	0.8274	0.6271	0.19									0.45	0.2813	0.3177	0.38
rs6501298	A					0.02	3.0985	1.7354	0.07	0.02	2.7863	1.6377	0.09				
rs6501302	A					0.01	2.8566	2.7272	0.29	0.01	2.5578	2.7258	0.35				
rs6501571	A	0.57	0.1999	0.6236	0.75	0.55	0.4379	0.3779	0.25	0.56	0.0391	0.3292	0.91				
rs6501584	A	0.58	-0.9772	0.6322	0.12	0.56	-0.1897	0.3804	0.62	0.56	-0.3938	0.3299	0.23				
rs6501587	A	0.48	0.4009	0.6308	0.53	0.52	0.0494	0.3769	0.90	0.51	0.1149	0.3271	0.73				
rs6501760	A	0.35	-0.8691	0.6433	0.18												
rs6501805	A	0.34	-0.4106	0.6778	0.54												
rs6501812	A	0.35	-0.7128	0.7246	0.33												
rs6501826	A	0.52	-0.234	0.5995	0.70												
rs6501830	C	0.34	0.0695	0.6605	0.92												
rs6501868	A	0.54	0.231	0.6443	0.72												
rs6501878	A	0.72	1.01	0.6797	0.14												
rs6501882	A	0.28	0.997	0.6785	0.14												
rs6501927	A	0.74	0.0172	0.741	0.98												
rs6501993	A	0.58	0.4835	0.6199	0.44	0.57	-0.0326	0.3743	0.93	0.58	0.0864	0.3247	0.79				
rs6502097	C	0.76	0.9007	0.8032	0.26												
rs653178	A	0.80	-0.6804	1.0358	0.51	0.86	-0.5332	0.5728	0.35	0.85	-0.5933	0.5051	0.24				
rs6565469	A	0.78	-0.104	0.8145	0.90												
rs6565472	A	0.08	1.4095	1.6463	0.39												
rs6565476	A	0.08	0.997	1.6187	0.54												
rs6565478	A	0.79	-0.1978	0.8217	0.81												
rs6565480	A	0.86	0.1462	0.9489	0.88												
rs6565484	A	0.71	0.3714	0.7162	0.60												

rs6565495	A	0.87	0.7696	1.0136	0.45								
rs6565499	A	0.13	0.4241	0.9827	0.67								
rs6565507	A	0.13	-0.6939	1.1259	0.54								
rs6565531	A	0.50	0.5771	0.6214	0.35								
rs6565535	A	0.51	-0.709	0.6258	0.26								
rs6565548	A	0.73	-1.2242	0.7171	0.09								
rs6565549	A	0.62	-0.4864	0.6374	0.45								
rs6565550	A	0.86	0.6383	0.9114	0.48								
rs6565570	A	0.17	-0.0581	0.863	0.95								
rs6565571	A	0.18	-0.3197	0.8505	0.71								
rs6565593	A	0.71	0.2531	0.6814	0.71								
rs6565604	A	0.76	-0.5322	0.7816	0.50								
rs6565605	A	0.76	-0.5322	0.7816	0.50								
rs6565612	A	0.26	-0.5681	0.7488	0.45								
rs6565616	A	0.74	-0.5646	0.7535	0.45								
rs6565633	A	0.31	-0.9248	0.7168	0.20	0.31	0.3235	0.4328	0.45	0.31	0.0637	0.3766	0.87
rs6565635	A	0.31	-0.7294	0.6808	0.28	0.32	0.3276	0.4111	0.43	0.31	0.0801	0.3589	0.82
rs6565642	A	0.91	-0.4434	1.0476	0.67								
rs6565643	A	0.55	-1.0228	0.6437	0.11								
rs6565650	A	0.42	0.5359	0.5978	0.37								
rs6565651	A	0.42	0.5558	0.5987	0.35								
rs6565666	A	0.03	-4.349	2.6247	0.10								
rs6565677	A	0.73	-0.5045	0.7088	0.48								
rs6565680	A	0.29	0.301	0.6989	0.67								
rs6565681	A	0.65	-0.5008	0.6467	0.44								
rs6565683	A	0.65	-0.4136	0.6577	0.53								
rs6565686	A	0.68	0.5893	0.6645	0.38								
rs6565689	A	0.48	-0.4775	0.6379	0.45								
rs6565697	A	0.14	-0.2876	0.8602	0.74	0.19	0.9803	0.4731	0.04	0.17	0.6962	0.4206	0.10
rs6711736	A	0.24	1.5985	0.7105	0.02	0.27	0.5606	0.4309	0.19	0.26	0.862	0.3732	0.02
rs67296984	A	0.11	-0.2234	1.248	0.86	0.08	-0.9283	0.7153	0.19	0.09	-0.7472	0.6333	0.24
rs6729869	A	0.24	1.5985	0.7105	0.02	0.27	0.5387	0.4296	0.21	0.26	0.8428	0.3724	0.02
rs674402	A	0.24	-0.103	0.8629	0.91								
rs6749447	A	0.41	-0.6155	0.6295	0.33	0.41	-0.3804	0.3701	0.30	0.41	-0.3229	0.3238	0.32

rs685099	A	0.84	-2.1107	0.8461	0.01								
rs685441	A	0.72	-0.9878	0.6979	0.16								
rs689730	A	0.21	-0.9113	0.7539	0.23								
rs689895	C	0.79	-0.9279	0.8882	0.30								
rs690124	A	0.16	-0.4283	0.9845	0.66								
rs690371	A	0.81	-0.343	0.8837	0.70								
rs690418	A	0.19	-0.6793	0.9033	0.45								
rs690514	A	0.18	-0.5677	0.9065	0.53								
rs690533	A	0.80	-0.198	0.8793	0.82								
rs691797	C	0.15	-1.4197	0.9264	0.13								
rs707707	A	0.41	0.912	0.6246	0.14								
rs715041	A	0.05	-1.9756	1.446	0.17	0.05	-0.8651	0.9024	0.34	0.05	-1.1233	0.7721	0.15
rs719430	A	0.76	1.1933	0.769	0.12								
rs719781	A	0.08	1.1795	1.1794	0.32								
rs7198	C	0.12	-0.0352	1.0668	0.97								
rs7206926	A	0.42	1.0814	0.6475	0.09								
rs7207088	A	0.23	0.0691	0.7244	0.92	0.24	0.2118	0.4338	0.63	0.24	0.2195	0.3769	0.56
rs7207208	A	0.28	-0.2634	0.7422	0.72								
rs7207596	A	0.07	0.6364	1.3069	0.63								
rs7207683	A	0.71	-0.2509	0.6786	0.71								
rs7207955	A	0.11	0.0877	1.0762	0.94								
rs7208049	A	0.92	-0.2354	1.3303	0.86	0.91	-0.2948	0.6945	0.67	0.91	-0.3093	0.6262	0.62
rs7208114	C	0.73	-0.7921	0.7167	0.27								
rs7208218	C	0.60	0.3491	0.6242	0.58								
rs7208264	C	0.19	-0.6936	0.7803	0.37								
rs7208285	A	0.47	0.4186	0.6256	0.50								
rs7208391	C	0.38	-0.3578	0.6554	0.59								
rs7208422	A	0.36	-0.1816	0.6694	0.79								
rs7208502	A	0.15	0.4737	0.9132	0.60								
rs7208536	A	0.14	0.1462	0.9489	0.88								
rs7208831	A	0.14	0.1462	0.9489	0.88								
rs7208892	A	0.50	-0.4551	0.594	0.44								
rs7209183	C	0.37	0.3074	0.6499	0.64								
rs7209235	A	0.80	0.0518	0.7735	0.95								

rs7209293	A	0.23	0.0331	0.7249	0.96	0.24	0.3192	0.4402	0.47	0.23	0.2855	0.3806	0.45				
rs7209406	A	0.15	0.0727	1.0076	0.94												
rs7209428	A	0.14	0.4501	0.9433	0.63	0.15	-1.0634	0.5393	0.05	0.15	-0.6888	0.4773	0.15				
rs7209474	A	0.78	1.2763	0.7416	0.09												
rs7209618	A	0.58	-1.5607	0.6252	0.01	0.57	-0.4823	0.37	0.19	0.57	-0.6809	0.3245	0.04	0.55	-0.4313	0.3163	0.17
rs7209710	A	0.86	0.1934	0.939	0.84	0.87	0.2868	0.5573	0.61	0.86	0.3441	0.4873	0.48				
rs7210122	A	0.26	0.292	0.7201	0.69												
rs7210391	A	0.12	-0.0931	1.057	0.93	0.13	-0.2518	0.5689	0.66	0.12	-0.2236	0.5078	0.66				
rs7210539	A	0.27	0.1705	0.6909	0.81	0.26	-0.1355	0.4274	0.75	0.26	0.0148	0.3681	0.97				
rs7210574	A	0.60	-0.0099	0.6589	0.99												
rs7210702	A	0.54	0.6596	0.6418	0.30	0.57	-0.2599	0.4061	0.52	0.56	-0.1742	0.3474	0.62				
rs7210742	A	0.39	-0.6812	0.6518	0.30												
rs7210946	A	0.47	0.0122	0.6303	0.98	0.42	-0.6187	0.3785	0.10	0.43	-0.2021	0.3307	0.54				
rs7210947	A	0.54	-0.1187	0.6256	0.85	0.48	0.275	0.3728	0.46	0.50	0.1092	0.3258	0.74				
rs7210951	A	0.71	0.6809	0.713	0.34												
rs7211021	A	0.11	2.0259	1.0728	0.06	0.11	-0.1309	0.6213	0.83	0.11	0.2901	0.5507	0.60				
rs7211095	A	0.15	0.4737	0.9132	0.60												
rs7211532	A	0.64	-0.3878	0.6683	0.56	0.57	-0.3589	0.3784	0.34	0.59	-0.3638	0.3347	0.28	0.59	-0.2824	0.3184	0.38
rs7211674	A	0.53	0.1024	0.6267	0.87												
rs7211773	A	0.40	0.3491	0.6242	0.58												
rs7211818	A	0.92	1.1525	1.5234	0.45												
rs7211960	A	0.08	-0.2146	1.2873	0.87												
rs7211963	A	0.24	0.4928	0.7756	0.53												
rs7211994	A	0.37	-0.1943	0.6492	0.76												
rs7212069	C	0.89	-1.7688	1.4796	0.23												
rs7212142	A	0.12	-0.3099	1.1679	0.79												
rs7212201	A	0.62	0.4552	0.6373	0.48												
rs7212305	A	0.23	0.0874	0.7222	0.90	0.24	0.2315	0.4343	0.59	0.24	0.2342	0.3769	0.53				
rs7212486	A	0.65	-0.1871	0.6298	0.77	0.65	0.4108	0.3862	0.29	0.65	0.2612	0.3366	0.44				
rs7212620	A	0.29	0.4734	0.699	0.50												
rs7212662	A	0.53	-0.1187	0.6676	0.86												
rs7212688	A	0.78	0.1724	0.7436	0.82	0.76	0.1571	0.4356	0.72	0.76	0.1797	0.3795	0.64				
rs7212823	A	0.24	0.5408	0.777	0.49												
rs7213204	A	0.56	0.6317	0.6452	0.33												

rs7213410	A	0.98	-2.7773	3.0348	0.36	0.94	0.0178	0.9095	0.98	0.95	-0.7225	0.8654	0.40				
rs7213638	A	0.14	0.1462	0.9489	0.88												
rs7213696	A	0.76	0.3259	0.7507	0.66												
rs7213735	A	0.41	-0.8061	0.6453	0.21	0.36	0.2478	0.3805	0.52	0.37	0.0535	0.334	0.87				
rs7213859	A	0.05	1.7806	2.1124	0.40	0.04	1.6236	1.0121	0.11	0.04	1.6263	0.9066	0.07				
rs7214286	A	0.20	0.938	0.7713	0.22												
rs7214678	A	0.78	-2.4788	0.7507	0.00												
rs7215260	A	0.53	-0.1729	0.623	0.78												
rs7215451	A	0.39	-1.48	0.6206	0.02												
rs7215470	A	0.67	-0.3245	0.668	0.63	0.73	0.1403	0.4235	0.74	0.71	0.0003	0.3632	1.00				
rs7215498	A	0.76	-1.0187	0.713	0.15	0.81	0.335	0.4783	0.48	0.79	-0.0084	0.4047	0.98				
rs7215534	A	0.15	-0.8429	0.9432	0.37												
rs7215669	A	0.69	0.4376	0.6629	0.51												
rs7215765	A	0.60	0.283	0.6895	0.68	0.62	-0.014	0.3887	0.97	0.61	-0.0804	0.3437	0.82				
rs7215994	A	0.75	0.3985	0.7152	0.58												
rs7216240	A	0.96	0.4279	2.1811	0.84	0.95	1.0243	1.0503	0.33	0.95	1.0509	0.9505	0.27				
rs7216559	A	0.07	-1.2784	1.7832	0.47												
rs7216577	A	0.46	0.0632	0.6053	0.92												
rs7216635	A	0.64	-0.095	0.65	0.88	0.61	-0.041	0.3924	0.92	0.62	-0.0766	0.34	0.82	0.64	-0.0631	0.3343	0.85
rs7216806	A	0.85	-1.4467	0.8459	0.09	0.80	0.9432	0.4698	0.04	0.81	0.3629	0.4152	0.38				
rs7217172	A	0.94	2.5502	1.7956	0.16	0.91	0.1536	0.7338	0.83	0.92	0.4561	0.6834	0.50	0.92	-1.3929	0.6775	0.04
rs7217223	A	0.11	0.005	1.2958	1.00												
rs7217395	A	0.37	-0.913	0.6272	0.15												
rs7217421	A	0.49	0.3034	0.6378	0.63												
rs7217702	C	0.82	0.1381	0.8596	0.87												
rs7217721	C	0.02	3.2618	2.9252	0.26	0.02	0.8254	1.6381	0.61	0.02	1.5178	1.4317	0.29				
rs7217829	A	0.30	-0.1912	0.6886	0.78	0.33	0.3325	0.3945	0.40	0.32	0.1998	0.3483	0.57				
rs7218122	A	0.56	0.2892	0.6223	0.64												
rs7218261	A	0.15	-1.0241	0.9066	0.26	0.18	-0.318	0.5133	0.54	0.17	-0.4028	0.4523	0.37				
rs7218347	A	0.93	-1.9608	1.2966	0.13												
rs7218498	C	0.60	0.2626	0.6515	0.69												
rs7218605	A	0.43	0.4815	0.6414	0.45												
rs7218729	C	0.38	0.3009	0.6367	0.64												
rs7218755	A					0.02	2.2125	2.2374	0.32	0.02	2.0275	2.2395	0.37				

rs7218918	A	0.20	1.8938	0.7901	0.02	0.18	-0.4379	0.5051	0.39	0.18	0.2083	0.4351	0.63				
rs7218924	A	0.82	-0.0737	0.9014	0.93												
rs7219247	A	0.20	-0.1578	0.8807	0.86												
rs7219316	A	0.73	-1.2366	0.7129	0.08												
rs7219318	A	0.38	-0.736	0.6415	0.25												
rs7219370	C	0.76	0.5924	0.7104	0.40												
rs7219382	A	0.73	-1.2366	0.7129	0.08												
rs7219452	A	0.85	0.511	1.2688	0.69	0.89	-0.7207	0.6762	0.29	0.88	-0.6401	0.5924	0.28	0.89	-0.6884	0.6307	0.28
rs7219493	A	0.89	-0.9938	0.98	0.31												
rs7220261	C	0.09	0.7665	1.1453	0.50												
rs7220294	A	0.91	0.5213	1.1443	0.65												
rs7220465	A	0.38	0.5515	0.6443	0.39												
rs7220493	A	0.28	0.0049	0.6866	0.99	0.26	-0.1547	0.4301	0.72	0.26	-0.0194	0.3705	0.96				
rs7220592	C	0.61	0.0903	0.6316	0.89	0.57	0.0993	0.3811	0.79	0.58	0.1036	0.3322	0.76				
rs7220955	A	0.46	1.0906	0.623	0.08												
rs7220985	C	0.39	-0.2461	0.6402	0.70	0.38	0.1673	0.3913	0.67	0.38	0.1089	0.3396	0.75				
rs7221014	A	0.82	0.2837	0.8409	0.74												
rs7221291	A	0.70	0.4354	0.6754	0.52												
rs7221314	A	0.59	1.2571	0.6364	0.05												
rs7221365	A	0.68	-0.3127	0.6757	0.64												
rs7221604	A	0.36	0.1352	0.647	0.83	0.37	-0.0818	0.3787	0.83	0.36	0.0355	0.3337	0.92				
rs7221608	C	0.88	0.0594	1.0102	0.95	0.87	-0.3742	0.5617	0.51	0.87	-0.2362	0.4985	0.64	0.87	-0.1365	0.4838	0.78
rs7221610	A	0.86	0.1055	0.9526	0.91												
rs7221665	A	0.79	-0.0721	0.9983	0.94												
rs7221823	A	0.89	0.005	1.2958	1.00												
rs7221948	A	0.89	0.5677	1.2036	0.64												
rs7222014	A	0.21	0.2217	0.7615	0.77												
rs7222024	A	0.40	0.3584	0.6243	0.57												
rs7222366	A	0.72	0.4538	0.7276	0.53												
rs7222531	A	0.73	-0.2276	0.7012	0.75	0.68	-0.1868	0.4105	0.65	0.70	-0.3297	0.3562	0.35				
rs7222861	A	0.42	-1.4837	0.6267	0.02	0.44	-0.3596	0.3709	0.33	0.44	-0.5775	0.3248	0.08				
rs7222924	A	0.30	0.0321	0.6986	0.96												
rs7223219	A	0.85	-0.0369	0.8794	0.97												
rs7223264	A	0.24	-0.4794	0.7367	0.52	0.19	0.4321	0.4777	0.37	0.20	0.1528	0.403	0.70				

rs7223304	A	0.80	-0.3361	0.7936	0.67												
rs7223613	A	0.24	-0.5322	0.7816	0.50												
rs7223756	A	0.49	1.1092	0.6277	0.08	0.47	0.413	0.371	0.27	0.48	0.5695	0.325	0.08				
rs7223939	A	0.73	-1.2031	0.7159	0.09												
rs7223992	A	0.61	0.3674	0.6437	0.57												
rs7224239	A	0.83	-0.3905	0.8569	0.65												
rs7224615	A	0.23	0.0429	0.7518	0.95												
rs7224668	A	0.66	-0.2748	0.6605	0.68												
rs7224711	A	0.44	1.0974	0.6224	0.08												
rs7224728	A	0.92	-2.6696	1.3797	0.05	0.93	0.2293	0.7483	0.76	0.93	-0.3628	0.6682	0.59				
rs7225131	A	0.28	0.997	0.6785	0.14												
rs7225323	A	0.04	-0.7422	2.2961	0.75												
rs7225364	A	0.59	-1.4893	0.628	0.02	0.54	-0.3428	0.3796	0.37	0.56	-0.6635	0.3317	0.05	0.63	-0.4275	0.3281	0.19
rs7225574	A	0.18	-1.0013	0.8613	0.25												
rs7225592	A	0.53	-1.8189	0.6444	0.00												
rs7225655	A	0.56	0.9647	0.6328	0.13	0.53	-0.0458	0.3838	0.91	0.54	0.2649	0.3337	0.43				
rs7225663	A	0.75	-0.6499	0.7973	0.42	0.72	-1.143	0.4348	0.01	0.73	-1.0291	0.3863	0.01	0.75	0.5174	0.3737	0.17
rs7226135	A	0.11	-1.1908	0.9757	0.22												
rs7226158	A	0.22	-0.9487	0.8178	0.25	0.24	-1.0851	0.4546	0.02	0.23	-1.0619	0.402	0.01	0.23	0.3065	0.3884	0.43
rs72849330	C	0.82	-0.5443	0.8156	0.50	0.81	-0.0567	0.4738	0.90	0.81	-0.1894	0.4135	0.65				
rs72852409	A	0.19	1.231	0.8174	0.13	0.18	0.5474	0.5095	0.28	0.18	0.7281	0.4343	0.09				
rs72853543	A	0.15	-0.1048	0.8892	0.91	0.17	-0.1029	0.4972	0.84	0.16	-0.1539	0.4374	0.73				
rs729996	A	0.21	-0.1704	0.8236	0.84												
rs73412102	A					0.98	-1.5723	1.709	0.36	0.98	0.1806	1.4842	0.90				
rs7342	C	0.36	0.7808	0.7028	0.27												
rs7342880	A	0.03	1.8826	2.0686	0.36												
rs734338	A	0.14	0.1957	1.0197	0.85												
rs7350896	A	0.56	0.8875	0.6184	0.15	0.52	-0.0312	0.3825	0.94	0.53	0.2592	0.3311	0.43				
rs736100	A	0.16	0.0131	0.8964	0.99												
rs736523	A	0.71	0.0068	0.7328	0.99												
rs7370	A	0.33	0.9898	0.7012	0.16												
rs73999901	A					0.04	0.6729	1.0351	0.52	0.03	0.9331	0.9713	0.34				
rs74006007	A					0.02	0.9712	2.31	0.67	0.02	2.0024	2.1127	0.34				
rs7405450	A	0.53	-0.754	0.6439	0.24												

rs7405469	C	0.40	-0.8403	0.6577	0.20												
rs7405640	A	0.68	0.8065	0.7346	0.27												
rs7405901	A	0.24	-0.5322	0.7816	0.50												
rs7405966	A	0.40	-0.8403	0.6577	0.20												
rs7406026	A	0.71	-0.2369	0.6798	0.73												
rs7406991	C	0.59	-0.8299	0.6618	0.21												
rs7409	A	0.41	-0.2206	0.6399	0.73												
rs7420	A	0.68	-1.0122	0.6506	0.12												
rs745318	A	0.41	0.9819	0.6337	0.12												
rs745666	C	0.35	-0.9614	0.6508	0.14												
rs746405	A	0.66	0.4045	0.668	0.54												
rs747321	A	0.08	0.2418	1.2886	0.85												
rs747742	A	0.09	-0.7649	1.0525	0.47												
rs749714	A					0.01	4.8802	2.9194	0.09	0.01	4.6145	2.922	0.11				
rs7501499	A	0.15	1.3529	0.9772	0.17	0.15	0.6023	0.5411	0.27	0.15	0.7304	0.4772	0.13	0.14	0.3552	0.4696	0.45
rs7501644	A	0.65	-0.5524	0.6888	0.42												
rs7501740	A	0.78	-0.0404	0.8254	0.96												
rs7501761	A	0.28	-0.6957	0.7233	0.34												
rs7502620	A	0.42	0.1168	0.6706	0.86												
rs7502835	A	0.35	-1.408	0.6633	0.03												
rs7503034	A	0.57	0.503	0.6317	0.43												
rs7503237	A	0.62	-0.7519	0.6369	0.24												
rs7503779	A	0.35	-0.1095	0.6787	0.87												
rs7503819	A	0.49	-0.1858	0.6862	0.79												
rs7503865	A	0.42	0.3575	0.6267	0.57	0.44	0.4731	0.3793	0.21	0.44	0.1101	0.3301	0.74				
rs7503911	A	0.15	-0.4154	0.9837	0.67												
rs750844	A	0.51	1.6465	0.6106	0.01												
rs751848	A	0.28	0.4612	0.6946	0.51	0.28	0.1147	0.4107	0.78	0.28	0.2309	0.3581	0.52				
rs753403	A	0.25	-0.9817	0.7239	0.18												
rs754708	A	0.32	-0.3475	0.6548	0.60												
rs755340	A	0.46	-1.5855	0.6286	0.01												
rs75560495	A	0.94	2.9197	2.0299	0.15	0.94	-1.0512	0.9801	0.28	0.94	0.0351	0.9086	0.97				
rs756075	A	0.15	0.2748	0.9341	0.77												
rs7591163	A	0.71	0.4253	0.7143	0.55	0.72	-0.1364	0.4499	0.76	0.72	-0.039	0.3864	0.92				

rs76290800	A	0.48	-0.0754	0.6049	0.90	0.46	-0.0071	0.3769	0.98	0.47	-0.0765	0.3255	0.81				
rs76299544	A	0.11	0.1611	1.1024	0.88	0.12	-0.2753	0.5884	0.64	0.12	-0.1811	0.5257	0.73				
rs7651	A	0.89	-0.6138	0.985	0.53												
rs76524459	A	0.96	2.7342	2.137	0.20	0.93	0.0534	0.8636	0.95	0.94	0.5762	0.8161	0.48				
rs771722	A	0.22	1.135	0.8176	0.17												
rs783230	A	0.57	-0.1379	0.6287	0.83												
rs783239	A	0.09	-0.0933	1.2105	0.94												
rs783241	A	0.40	0.2894	0.6074	0.63												
rs783250	A	0.56	0.5568	0.6103	0.36												
rs79087642	A	0.12	-1.011	1.1137	0.36	0.11	-0.5788	0.6532	0.38	0.11	-0.8115	0.5712	0.16				
rs80198744	A	0.04	-0.1443	1.682	0.93	0.09	0.9279	0.7478	0.21	0.08	0.5855	0.6788	0.39				
rs8064345	A	0.06	-3.6931	1.6251	0.02												
rs8064357	A	0.32	-1.0001	0.6713	0.14	0.36	-0.5579	0.4108	0.17	0.35	-0.6255	0.3549	0.08				
rs8064529	A	0.29	0.6898	0.7207	0.34												
rs8064769	A	0.23	0.0005	0.7222	1.00	0.24	0.2783	0.4339	0.52	0.24	0.2382	0.3766	0.53				
rs8065144	A	0.65	0.295	0.6752	0.66												
rs8065364	A	0.90	-0.2682	0.9772	0.78												
rs8065422	A	0.86	-0.4729	0.8775	0.59												
rs8065431	A	0.68	0.5892	0.6799	0.39	0.67	-0.1418	0.3933	0.72	0.68	0.131	0.3466	0.71				
rs8065523	A	0.06	-0.05	1.3792	0.97												
rs8065598	A	0.08	1.1525	1.5234	0.45												
rs8065843	A	0.62	0.1121	0.669	0.87												
rs8066695	A	0.56	0.1402	0.6304	0.82												
rs8066768	A					0.06	-0.3804	0.9485	0.69	0.06	0.281	0.9098	0.76	0.04	-0.9719	0.8889	0.27
rs8066857	A	0.15	0.2388	1.059	0.82	0.11	0.4744	0.6496	0.47	0.11	0.4506	0.554	0.42				
rs8066867	A	0.09	1.4714	1.482	0.32												
rs8066993	A	0.25	-0.4197	0.7616	0.58												
rs8067167	A	0.34	-0.6738	0.6777	0.32	0.43	-0.426	0.3868	0.27	0.40	-0.3988	0.339	0.24				
rs8067235	A	0.17	0.1665	0.8413	0.84												
rs8067292	A	0.77	0.2869	0.7352	0.70												
rs8067409	A					0.02	-2.9635	1.6168	0.07	0.02	-2.1932	1.508	0.15				
rs8067574	A	0.30	0.7071	0.6727	0.29												
rs8067885	A	0.74	-0.2825	0.7183	0.69	0.79	0.1904	0.4571	0.68	0.78	0.0779	0.3886	0.84				
rs8067947	A	0.53	0.1808	0.6332	0.78												

rs8067984	A	0.27	1.8792	0.7189	0.01												
rs8068064	A	0.18	0.0257	0.8842	0.98												
rs8068137	A	0.32	-0.7769	0.6528	0.23												
rs8068511	A	0.27	-0.3352	0.6959	0.63												
rs8068514	A	0.95	1.7202	1.539	0.26												
rs8068637	A	0.11	-0.2904	1.05	0.78												
rs8068796	A	0.40	0.0399	0.6213	0.95												
rs8068871	A	0.31	0.7043	0.6631	0.29	0.24	0.0756	0.4453	0.87	0.26	0.2725	0.378	0.47	0.25	0.1351	0.3664	0.71
rs8069348	C	0.07	-1.9608	1.2966	0.13												
rs8069502	A	0.24	0.2195	0.7463	0.77	0.20	0.0501	0.4634	0.91	0.21	-0.0291	0.3998	0.94				
rs8069521	A	0.07	-1.1107	1.4046	0.43												
rs8070106	A	0.29	0.4886	0.6868	0.48												
rs8070127	A	0.13	0.2455	1.1175	0.83												
rs8070274	A	0.95	-1.5939	2.1347	0.46	0.96	-1.0802	1.0023	0.28	0.96	-1.5435	0.9042	0.09				
rs8070383	A	0.02	3.1889	2.9333	0.28	0.02	0.9033	1.6167	0.58	0.02	1.5685	1.4161	0.27				
rs8070406	A					0.97	1.4509	1.2861	0.26	0.97	1.3311	1.1977	0.27	0.98	4.3844	1.5017	0.00
rs8070488	A	0.87	-1.4273	1.0073	0.16												
rs8070953	A	0.75	0.1242	0.7452	0.87												
rs8070973	A	0.48	-0.0699	0.6188	0.91	0.52	0.1764	0.3819	0.64	0.51	-0.0152	0.3292	0.96	0.50	0.3272	0.3156	0.30
rs8071004	A	0.95	4.0303	1.9304	0.04	0.91	0.3084	0.7757	0.69	0.92	0.7557	0.7259	0.30	0.93	-1.6346	0.7241	0.02
rs8071015	A	0.91	1.3861	1.4683	0.35												
rs8071198	A	0.68	-0.6684	0.6836	0.33												
rs8071668	A	0.26	-0.1425	0.821	0.86												
rs8071693	A	0.02	-9.6281	3.4257	0.00	0.04	0.2449	1.1376	0.83	0.04	-0.4615	0.9738	0.64	0.05	1.4381	0.893	0.11
rs8071885	C	0.62	1.2795	0.6492	0.05												
rs8071975	A	0.59	-0.212	0.6336	0.74												
rs8072027	A					0.99	2.8832	2.8389	0.31	0.99	-1.1798	1.9517	0.55				
rs8072277	A	0.20	0.1741	0.8547	0.84												
rs8072347	A	0.46	-0.4963	0.6168	0.42	0.47	0.2004	0.3722	0.59	0.47	0.0048	0.3247	0.99				
rs8072592	A	0.22	-0.104	0.8145	0.90												
rs8073077	A	0.16	-2.6259	0.9484	0.01	0.20	-0.0657	0.5151	0.90	0.19	-0.675	0.463	0.14	0.14	0.4439	0.5139	0.39
rs8073182	A	0.61	-0.1027	0.6911	0.88												
rs8073197	A	0.93	-2.0883	1.2774	0.10	0.92	1.1538	0.711	0.10	0.92	0.3542	0.6232	0.57				
rs8073414	A	0.19	0.2422	0.8662	0.78												

rs8073529	A	0.24	0.2342	0.7493	0.75	0.23	0.0198	0.4471	0.96	0.23	0.0751	0.3893	0.85				
rs8073550	A	0.37	0.1362	0.6727	0.84	0.38	-0.3802	0.3911	0.33	0.37	-0.2772	0.3418	0.42				
rs8073615	A	0.34	-0.4829	0.6601	0.46												
rs8073660	A	0.74	0.277	0.7022	0.69												
rs8073763	A	0.24	0.8592	0.7644	0.26												
rs8073791	A					0.06	-0.3937	0.9517	0.68	0.05	0.5071	0.869	0.56	0.04	-1.1017	0.8956	0.22
rs8074418	A	0.76	-0.2302	0.7176	0.75	0.79	0.3367	0.4603	0.46	0.78	0.1676	0.3905	0.67				
rs8074685	A	0.58	-0.0997	0.6517	0.88												
rs8074821	A	0.72	1.0208	0.6776	0.13												
rs8074842	C	0.24	0.4362	0.7394	0.56												
rs8075102	A	0.40	-0.8403	0.6577	0.20												
rs8075298	A	0.47	-2.0724	0.6321	0.00												
rs8075300	A	0.26	-0.6218	0.7136	0.38	0.25	-0.562	0.42	0.18	0.25	-0.5785	0.3658	0.11				
rs8075376	A	0.25	0.115	0.7028	0.87	0.25	-0.0793	0.4387	0.86	0.25	0.0642	0.3779	0.87				
rs8075628	A	0.15	-0.9588	0.8652	0.27												
rs8075657	A	0.28	-0.0981	0.687	0.89	0.27	-0.6024	0.4252	0.16	0.27	-0.4889	0.3691	0.19				
rs8075839	A	0.82	-0.5745	0.9074	0.53												
rs8075897	A	0.18	0.5296	0.7911	0.50												
rs8076283	A	0.63	-0.0484	0.6681	0.94	0.62	-0.3778	0.3911	0.33	0.62	-0.3018	0.3412	0.38				
rs8076334	A	0.22	0.5715	0.7354	0.44	0.22	0.134	0.4536	0.77	0.22	0.146	0.39	0.71				
rs8076416	A	0.17	-0.5396	0.9801	0.58	0.20	-0.7644	0.5305	0.15	0.19	-0.5897	0.4753	0.21	0.15	-0.3645	0.5114	0.48
rs8076595	A	0.72	-0.0462	0.677	0.95	0.72	-0.5676	0.4193	0.18	0.72	-0.4472	0.3627	0.22				
rs8077024	A	0.47	-0.2826	0.6464	0.66												
rs8077046	A	0.31	-0.7305	0.6589	0.27												
rs8077205	A	0.22	0.6165	0.743	0.41	0.24	-0.6252	0.4432	0.16	0.23	-0.2963	0.387	0.44				
rs8077819	A	0.64	0.6652	0.6574	0.31												
rs8077901	A	0.14	0.1462	0.9489	0.88												
rs8077911	C	0.28	1.5383	0.6797	0.02	0.32	0.523	0.4021	0.19	0.31	0.7191	0.3506	0.04				
rs8078251	A	0.24	0.386	0.7107	0.59												
rs8078334	A	0.68	0.4598	0.6535	0.48	0.68	-0.0806	0.3995	0.84	0.68	-0.04	0.3455	0.91				
rs8078532	A	0.36	0.4634	0.6431	0.47												
rs8078577	A	0.33	0.4246	0.6897	0.54												
rs8078643	A	0.54	0.0643	0.6312	0.92												
rs8078752	A	0.42	0.3894	0.6153	0.53	0.42	0.269	0.3752	0.47	0.42	0.1927	0.325	0.55				

rs8078791	A	0.13	0.1689	1.1259	0.88	0.18	-0.7637	0.5519	0.17	0.16	-0.4787	0.5028	0.34				
rs8078851	A	0.22	0.1501	0.7444	0.84												
rs8078855	A	0.40	1.336	0.8647	0.12												
rs8079156	A	0.81	0.2795	0.8345	0.74												
rs8079383	A	0.12	-2.5862	1.1367	0.02	0.15	0.0933	0.5983	0.88	0.14	-0.5015	0.5398	0.35	0.10	0.3306	0.6025	0.58
rs8079537	A	0.86	-0.6796	0.9572	0.48												
rs8079626	A	0.68	-1.12	0.6651	0.09												
rs8079646	A	0.22	-0.8599	0.736	0.24	0.17	-0.1831	0.4999	0.71	0.19	-0.3637	0.4234	0.39				
rs8079757	A	0.82	-0.4716	0.907	0.60	0.80	0.4485	0.5412	0.41	0.81	0.2135	0.4718	0.65				
rs8079781	A	0.50	-0.4505	0.6503	0.49												
rs8080101	A	0.59	-1.1359	0.661	0.09	0.56	0.1128	0.3774	0.77	0.57	-0.2548	0.3336	0.44				
rs8080155	A	0.68	0.3713	0.6538	0.57	0.68	-0.0532	0.3998	0.89	0.68	-0.0446	0.3458	0.90				
rs8080265	A	0.08	2.9316	1.6316	0.07												
rs8080597	A	0.09	-0.3416	1.3527	0.80	0.09	-0.0681	0.741	0.93	0.09	-0.1205	0.6641	0.86				
rs8081143	A	0.58	-1.3263	0.6239	0.03	0.58	-0.2296	0.3738	0.54	0.58	-0.4105	0.3276	0.21				
rs8081168	A	0.81	0.3403	0.8445	0.69												
rs8081176	A	0.72	-0.3349	0.7161	0.64												
rs8081466	A	0.77	-0.0696	0.7172	0.92	0.76	0.2634	0.4325	0.54	0.76	0.2074	0.375	0.58				
rs8081669	A	0.34	0.3519	0.6443	0.59												
rs8081783	A	0.78	1.2594	0.7341	0.09												
rs8081928	C	0.25	0.0257	0.7238	0.97												
rs8082005	A	0.37	-1.1778	0.6538	0.07												
rs8082181	A	0.08	0.9012	1.4326	0.53	0.05	-0.3989	0.9436	0.67	0.05	0.0203	0.7998	0.98				
rs8082252	A	0.43	0.8347	0.6159	0.18												
rs8082289	A	0.23	0.02	0.7226	0.98	0.24	0.2546	0.4341	0.56	0.24	0.2203	0.3766	0.56				
rs8082303	A	0.78	-0.104	0.8145	0.90												
rs8082640	A	0.42	-1.3157	0.6253	0.04	0.42	-0.2882	0.3744	0.44	0.42	-0.4461	0.328	0.17	0.41	-0.1766	0.3183	0.58
rs809740	A	0.57	-0.1379	0.6287	0.83												
rs8150	C	0.29	-0.8602	0.684	0.21												
rs820129	A	0.59	0.9019	0.6196	0.15												
rs820134	A	0.10	1.8565	1.193	0.12												
rs820145	A	0.11	0.854	1.4982	0.57												
rs820146	A	0.12	1.0491	1.4185	0.46												
rs820152	A	0.83	-0.3058	0.8311	0.71												

rs820155	C	0.41	0.9343	0.6221	0.13												
rs820157	A	0.88	1.3915	1.3049	0.29												
rs820184	A	0.83	-0.3932	0.8329	0.64												
rs820186	A	0.12	0.5525	1.4626	0.71												
rs820190	A	0.11	0.9247	1.4187	0.51												
rs820196	A	0.63	0.5988	0.6467	0.35												
rs820201	A	0.41	0.9837	0.6267	0.12												
rs820202	A	0.41	0.9776	0.6257	0.12												
rs820210	A	0.58	1.0696	0.6174	0.08												
rs820216	A	0.87	1.5187	1.2867	0.24												
rs820233	A	0.53	-0.5098	0.6292	0.42												
rs820242	A	0.89	-2.1826	1.0167	0.03												
rs820264	A	0.10	-1.7902	1.1216	0.11												
rs866414	A	0.19	-0.4056	0.7872	0.61	0.22	0.6153	0.4751	0.20	0.21	0.2728	0.4112	0.51				
rs868432	A	0.55	-1.4188	0.6209	0.02												
rs870355	A	0.30	-0.9735	0.7463	0.19												
rs871443	A	0.33	-0.3648	0.7307	0.62												
rs871741	A	0.40	-0.7481	0.6399	0.24	0.36	0.2749	0.3798	0.47	0.37	0.0839	0.3331	0.80				
rs872640	A	0.79	-0.1176	0.8348	0.89												
rs8746	A	0.12	1.5824	0.9793	0.11												
rs878906	A	0.32	-1.0159	0.6469	0.12												
rs880827	A	0.32	-1.0159	0.6469	0.12												
rs881502	A	0.63	-0.6918	0.6404	0.28												
rs8836	C	0.60	-0.2097	0.6389	0.74												
rs883890	C	0.08	0.9012	1.4326	0.53	0.05	-0.42	0.9472	0.66	0.05	0.0076	0.802	0.99				
rs884204	A	0.79	-0.1103	0.8222	0.89												
rs884446	A	0.21	-0.7998	0.7463	0.28	0.18	0.6318	0.4867	0.19	0.19	0.2879	0.4178	0.49				
rs884652	A	0.12	1.1206	1.0228	0.27	0.15	-0.2365	0.5425	0.66	0.14	0.0263	0.482	0.96	0.15	-0.7282	0.463	0.12
rs892960	A	0.42	0.1926	0.6598	0.77												
rs894310	A	0.11	-0.2234	1.248	0.86	0.08	-0.9283	0.7153	0.19	0.09	-0.7472	0.6333	0.24				
rs894545	A	0.78	0.1043	0.7335	0.89												
rs894546	A	0.15	0.2906	0.8684	0.74												
rs894939	A	0.31	0.4648	0.7326	0.53												
rs894945	A	0.26	0.4872	0.7234	0.50												

rs894949	A	0.35	-0.9333	0.8896	0.29												
rs894956	C	0.86	-0.8613	1.0965	0.43												
rs897587	A	0.19	-0.359	0.8646	0.68	0.22	-0.802	0.4738	0.09	0.21	-0.723	0.4201	0.09				
rs897588	A	0.27	0.0602	0.7321	0.93	0.28	0.2677	0.4245	0.53	0.28	0.1438	0.372	0.70				
rs897593	A	0.64	0.9528	0.6368	0.13	0.56	-0.0753	0.3824	0.84	0.58	0.1503	0.332	0.65				
rs897594	A	0.28	1.0223	0.8646	0.24												
rs897595	A	0.44	0.2703	0.6273	0.67	0.38	0.2266	0.3945	0.57	0.39	0.2496	0.3382	0.46				
rs897597	C	0.59	-0.3388	0.6922	0.62	0.60	0.4609	0.4006	0.25	0.60	0.2155	0.3511	0.54	0.55	0.2355	0.3246	0.47
rs897600	A	0.24	-0.9351	0.7217	0.20	0.20	0.0352	0.4744	0.94	0.21	-0.2069	0.405	0.61				
rs898085	A	0.70	0.9881	0.6905	0.15												
rs898525	A	0.39	0.2269	0.6815	0.74	0.39	-0.1891	0.391	0.63	0.39	-0.0575	0.3451	0.87				
rs898533	A	0.77	-0.5031	0.7722	0.51	0.78	0.0916	0.4571	0.84	0.78	-0.0975	0.3976	0.81				
rs898534	A	0.23	-0.517	0.7677	0.50	0.21	0.022	0.4671	0.96	0.22	-0.1424	0.4037	0.72				
rs898537	A	0.41	0.9037	0.654	0.17												
rs899317	A	0.46	1.2498	0.6284	0.05												
rs901065	A	0.11	-0.3004	1.0591	0.78												
rs902731	A	0.52	-0.2956	0.6286	0.64												
rs9038	A	0.19	0.3401	0.8995	0.71												
rs907898	A	0.77	0.3417	0.7518	0.65	0.76	-0.3612	0.4538	0.43	0.77	-0.1181	0.3953	0.77				
rs907899	A	0.60	0.5668	0.6147	0.36	0.53	0.1961	0.378	0.60	0.55	0.2217	0.3285	0.50				
rs907901	A	0.05	-1.5939	2.1347	0.46	0.03	-1.6476	1.053	0.12	0.04	-1.998	0.9364	0.03				
rs907910	A	0.18	0.3836	0.8281	0.64												
rs907911	A	0.21	-0.7886	0.7517	0.29	0.18	0.3146	0.4939	0.52	0.19	0.0246	0.4235	0.95				
rs907915	A	0.72	0.1915	0.6833	0.78	0.74	-0.3297	0.426	0.44	0.73	-0.0875	0.3681	0.81				
rs908241	A	0.54	-0.6918	0.62	0.26												
rs910557	A	0.19	-1.6093	0.8748	0.07												
rs920131	A	0.22	-1.2442	0.7571	0.10												
rs922838	C	0.44	1.1682	0.6225	0.06												
rs925607	A	0.82	-0.1884	0.9376	0.84	0.85	-0.0708	0.5573	0.90	0.85	-0.1513	0.4867	0.76	0.89	-0.4519	0.556	0.42
rs9302890	A	0.37	-0.1027	0.6966	0.88	0.37	-0.3451	0.3912	0.38	0.37	-0.2855	0.347	0.41				
rs9302897	A	0.82	-0.1728	0.8974	0.85	0.80	-0.7331	0.494	0.14	0.81	-0.6243	0.4379	0.15				
rs9302989	A	0.91	-0.1118	1.4094	0.94												
rs9302990	A	0.09	-0.1118	1.4094	0.94												
rs9302994	A	0.40	0.1986	0.6508	0.76												

rs9308945	A	0.76	1.5985	0.7105	0.02	0.73	0.5422	0.4304	0.21	0.74	0.8496	0.3729	0.02				
rs9319623	C	0.49	-0.1989	0.6211	0.75	0.53	0.1492	0.3709	0.69	0.52	0.0432	0.3243	0.89				
rs934668	A	0.58	-0.0671	0.6453	0.92	0.64	-0.3294	0.3857	0.39	0.62	-0.2285	0.3345	0.49				
rs935334	A	0.08	0.542	1.5649	0.73	0.08	0.2811	0.7523	0.71	0.08	0.3404	0.6809	0.62				
rs936056	A	0.44	-0.3204	0.6196	0.61												
rs936393	A	0.84	-1.3349	0.8341	0.11												
rs938283	A	0.83	0.5248	0.8779	0.55	0.83	0.3034	0.5057	0.55	0.84	0.227	0.4414	0.61				
rs938348	A	0.59	0.2395	0.6244	0.70												
rs938350	A	0.61	0.5055	0.6317	0.42												
rs939540	A	0.27	-0.6386	0.7631	0.40												
rs939543	A	0.58	-1.2088	0.6416	0.06												
rs959260	A	0.91	-0.2289	1.3874	0.87												
rs9635664	A	0.42	0.1841	0.6416	0.77	0.41	0.2493	0.3906	0.52	0.41	0.1576	0.3388	0.64				
rs9660	A	0.89	-0.9938	0.98	0.31												
rs9674550	A	0.28	-0.3074	0.6971	0.66												
rs9674559	A	0.93	0.5525	1.6326	0.74												
rs9675230	A	0.35	-0.3902	0.6677	0.56												
rs9675239	A	0.70	-0.3961	0.6728	0.56	0.69	-0.3443	0.4109	0.40	0.69	-0.353	0.3573	0.32				
rs968493	A	0.73	0.496	0.7096	0.48	0.72	0.0082	0.4241	0.98	0.73	0.1873	0.3705	0.61				
rs969413	A	0.23	-2.2123	0.8168	0.01	0.27	-0.3303	0.4404	0.45	0.26	-0.7888	0.3957	0.05	0.22	0.3234	0.409	0.43
rs971625	A	0.53	-0.5346	0.6098	0.38	0.58	-0.0488	0.3791	0.90	0.56	-0.1675	0.327	0.61	0.57	0.1439	0.3341	0.67
rs971626	A	0.47	-0.5803	0.6123	0.34	0.42	-0.082	0.3823	0.83	0.43	-0.2007	0.3293	0.54				
rs9747668	A	0.12	1.2198	1.0905	0.26												
rs978415	A	0.24	-0.003	0.7242	1.00	0.21	0.0965	0.4569	0.83	0.22	-0.0354	0.3927	0.93				
rs9789009	A	0.89	-2.4168	1.1278	0.03	0.91	-0.902	0.6695	0.18	0.90	-1.3103	0.5853	0.03	0.91	-0.3467	0.651	0.59
rs9807041	A	0.92	0.997	1.6187	0.54												
rs9815354	A	0.14	0.795	0.9228	0.39	0.13	-0.5385	0.5735	0.35	0.13	-0.1386	0.496	0.78				
rs981645	C	0.76	0.276	0.7721	0.72	0.75	-0.518	0.4362	0.24	0.75	-0.4385	0.3832	0.25				
rs9890248	C	0.83	0.3058	0.929	0.74	0.86	0.5612	0.5414	0.30	0.85	0.5473	0.474	0.25				
rs9890550	A	0.53	0.1567	0.6388	0.81												
rs9890664	A	0.70	-0.1151	0.8064	0.89												
rs9891076	A	0.40	0.2626	0.6515	0.69												
rs9891160	A	0.07	0.5525	1.6326	0.74												
rs9892163	A	0.68	-1.6324	0.7002	0.02												

rs9892166	C	0.88	0.1185	1.0913	0.91												
rs9892257	A	0.06	-2.4343	1.3743	0.08												
rs9892688	C	0.27	-1.2011	0.7142	0.09												
rs9893035	A	0.56	1.1861	0.634	0.06												
rs9893556	A	0.58	0.3075	0.6103	0.61	0.62	-0.0381	0.3806	0.92	0.61	0.042	0.3292	0.90	0.59	0.0609	0.3277	0.85
rs9894131	A	0.14	-0.5444	1.1414	0.63	0.12	-0.5899	0.6319	0.35	0.12	-0.7445	0.5519	0.18				
rs9894139	A	0.46	-0.7442	0.6253	0.23	0.49	0.2422	0.3687	0.51	0.48	-0.0119	0.3236	0.97	0.47	-0.2424	0.3176	0.45
rs9894429	A	0.76	-0.5322	0.7816	0.50												
rs9894736	A	0.11	-0.4936	1.1745	0.67												
rs9894899	A	0.76	-0.003	0.7242	1.00	0.80	0.0806	0.4601	0.86	0.79	-0.0529	0.3948	0.89				
rs9895584	A	0.86	0.1462	0.9489	0.88												
rs9895586	C	0.47	-0.2723	0.6196	0.66	0.46	-0.4717	0.3729	0.21	0.46	-0.3832	0.3228	0.24	0.45	0.1013	0.3204	0.75
rs9895647	A	0.69	-0.1796	0.6896	0.79	0.70	0.0554	0.397	0.89	0.70	0.1106	0.348	0.75				
rs9895930	A	0.94	0.1365	1.8505	0.94												
rs9895947	A	0.60	0.2626	0.6515	0.69												
rs9896073	C	0.50	0.5505	0.6324	0.38												
rs9896146	A	0.20	-0.6728	0.7822	0.39	0.24	-0.4574	0.4459	0.31	0.23	-0.4268	0.3949	0.28				
rs9896771	A	0.79	-0.1224	0.8107	0.88												
rs9896850	A	0.16	-2.6129	0.94	0.01	0.20	-0.12	0.5131	0.82	0.19	-0.6978	0.4599	0.13	0.15	0.2358	0.5048	0.64
rs9897213	A	0.69	0.158	0.6642	0.81												
rs9897367	C	0.49	0.9755	0.6304	0.12	0.47	0.4276	0.3701	0.25	0.47	0.558	0.3244	0.09				
rs9897410	A	0.51	1.1958	0.6216	0.05												
rs9897453	A	0.22	-0.1343	0.8164	0.87												
rs9897730	A	0.24	0.7397	0.7319	0.31	0.27	0.1524	0.4229	0.72	0.26	0.2084	0.3712	0.57				
rs9897764	A	0.31	0.3138	0.6603	0.63												
rs9897826	A	0.06	2.8907	1.9927	0.15												
rs9897830	A	0.86	0.1462	0.9489	0.88												
rs9897914	A	0.49	-0.1145	0.6175	0.85	0.50	0.2743	0.3712	0.46	0.50	0.0905	0.3223	0.78				
rs9898046	A	0.26	0.2213	0.7018	0.75	0.25	0.004	0.4325	0.99	0.25	0.1457	0.3739	0.70				
rs9898178	A	0.86	0.531	0.8975	0.55												
rs9898301	A	0.12	-0.413	1.1679	0.72												
rs9898379	C	0.71	-0.7167	0.7305	0.33												
rs9898441	A	0.86	0.1462	0.9489	0.88												
rs9898469	A	0.26	1.2936	0.7492	0.08	0.24	0.468	0.4504	0.30	0.24	0.6811	0.394	0.08				

rs9898470	A	0.96	-2.0051	2.3612	0.40								
rs9898803	A	0.12	0.4692	1.0495	0.65	0.10	0.6699	0.6417	0.30	0.10	0.5352	0.555	0.33
rs9899051	A	0.37	0.4	0.641	0.53								
rs9899178	A	0.43	0.4626	0.6209	0.46								
rs9899531	A	0.04	0.0377	2.4676	0.99	0.02	-1.1092	1.4791	0.45	0.02	-0.9022	1.2712	0.48
rs9899673	A	0.26	-1.1494	0.7557	0.13								
rs9899687	A	0.91	-0.1118	1.4094	0.94								
rs9899843	A	0.07	0.5525	1.6326	0.74								
rs9899862	A	0.12	-0.4513	1.0177	0.66								
rs9900002	A	0.91	0.1723	1.4249	0.90								
rs9900506	A	0.02	-3.4154	2.7895	0.22								
rs9900586	A	0.10	0.7197	1.2123	0.55								
rs9900690	A	0.72	0.1246	0.7015	0.86	0.72	-0.4093	0.4225	0.33	0.72	-0.2939	0.3661	0.42
rs9900972	A	0.15	0.8229	0.8814	0.35								
rs9901049	A	0.86	0.1462	0.9489	0.88								
rs9901361	A	0.18	-2.0951	0.8575	0.01								
rs9901434	A	0.09	-0.5767	1.4117	0.68								
rs9901514	A	0.73	-1.2566	0.7119	0.08								
rs9901648	A	0.41	0.6575	0.6392	0.30								
rs9901846	A	0.39	-0.7472	0.6378	0.24								
rs9902013	A	0.74	0.4076	0.7622	0.59								
rs9902358	A	0.24	0.7291	0.7043	0.30								
rs9902690	A	0.72	0.9889	0.6768	0.14								
rs9902702	A	0.50	0.345	0.6271	0.58								
rs9902818	A	0.52	0.6048	0.5905	0.31	0.53	-0.3041	0.3769	0.42	0.53	-0.0601	0.3227	0.85
rs9902912	A					0.01	1.1074	3.1647	0.73	0.01	0.3863	2.1485	0.86
rs9903640	C	0.30	0.9761	0.6884	0.16								
rs9903812	A	0.87	-0.7386	0.9139	0.42								
rs9904341	C	0.50	1.8145	0.6281	0.00								
rs9904414	A	0.67	-0.7382	0.6491	0.26	0.66	-0.9012	0.3938	0.02	0.66	-0.8099	0.342	0.02
rs9904630	A	0.75	-0.0445	0.7252	0.95	0.73	0.0547	0.4311	0.90	0.73	0.061	0.3756	0.87
rs9904772	A	0.86	-2.358	1.2857	0.07	0.89	-0.5282	0.6472	0.41	0.89	-0.7619	0.5741	0.18
rs9904783	A	0.74	0.4793	0.7661	0.53	0.73	0.1453	0.4292	0.74	0.73	0.1679	0.3799	0.66
rs9904844	A	0.16	0.5325	0.8521	0.53								

rs9904969	C	0.27	1.2677	0.7284	0.08	0.24	0.559	0.4441	0.21	0.25	0.7567	0.3858	0.05				
rs9905663	A	0.44	-0.0798	0.6389	0.90												
rs9905685	C	0.50	-1.3893	0.6144	0.02	0.52	-0.15	0.3685	0.68	0.51	-0.5057	0.3216	0.12				
rs9905852	A	0.46	-0.8523	0.6183	0.17	0.49	0.2666	0.3674	0.47	0.48	-0.0174	0.3216	0.96	0.47	-0.1957	0.3182	0.54
rs9905991	A	0.83	1.6372	0.8811	0.06												
rs9906023	A	0.26	1.283	0.7459	0.09	0.24	0.6193	0.4494	0.17	0.25	0.7948	0.3926	0.04				
rs9906253	A	0.03	-4.0331	2.709	0.14												
rs9906330	A	0.63	1.2091	0.6647	0.07												
rs9906525	A	0.53	-0.5488	0.6233	0.38	0.51	0.2056	0.3681	0.58	0.52	0.0152	0.3228	0.96	0.52	-0.2758	0.3169	0.38
rs9906700	A	0.65	0.8562	0.6781	0.21	0.68	0.1992	0.427	0.64	0.67	0.4055	0.3657	0.27				
rs9906827	A	0.15	0.4854	0.9121	0.59												
rs9907094	A	0.57	-1.5659	0.6238	0.01	0.55	0.041	0.3723	0.91	0.55	-0.3131	0.3263	0.34	0.54	-0.1095	0.3166	0.73
rs9907115	A	0.47	-0.6148	0.63	0.33												
rs9907318	A	0.48	0.7012	0.6289	0.26	0.45	0.0907	0.3707	0.81	0.45	0.2017	0.3239	0.53				
rs9907519	A	0.68	-0.4852	0.6543	0.46												
rs9907544	A	0.23	0.191	0.8283	0.82	0.24	-0.602	0.4728	0.20	0.24	-0.5231	0.4183	0.21	0.19	-0.4533	0.4497	0.31
rs9907837	A	0.50	-0.2382	0.6252	0.70	0.47	-0.0208	0.3737	0.96	0.48	-0.0792	0.3263	0.81				
rs9908454	A	0.62	0.4384	0.6399	0.49												
rs9908495	A	0.82	-1.0074	0.8587	0.24												
rs9909462	A	0.23	0.5436	0.7527	0.47												
rs9909805	A	0.45	-0.3596	0.6373	0.57												
rs9910192	A	0.57	-0.0545	0.6438	0.93	0.54	0.544	0.3787	0.15	0.54	0.4611	0.3312	0.16				
rs9910454	C	0.21	0.4252	0.8102	0.60												
rs9910615	A	0.64	1.5046	0.6975	0.03	0.69	0.2953	0.4213	0.48	0.67	0.5561	0.3675	0.13				
rs9910745	A	0.14	-0.4842	0.9764	0.62												
rs9910792	A	0.64	0.5557	0.67	0.41	0.59	-0.5565	0.3749	0.14	0.61	-0.2621	0.3314	0.43				
rs9911063	A	0.83	0.4082	0.8318	0.62												
rs9911171	A	0.08	1.1525	1.5234	0.45												
rs9911245	A	0.63	-0.0769	0.639	0.90	0.62	0.2126	0.3822	0.58	0.62	0.1536	0.3353	0.65	0.68	-0.0205	0.3366	0.95
rs9911346	A	0.29	-0.0617	0.6628	0.93	0.29	0.153	0.4059	0.71	0.29	0.0969	0.3519	0.78				
rs9911502	C	0.28	0.7665	0.7225	0.29												
rs9911523	A	0.05	-2.7411	2.0486	0.18												
rs9911538	A	0.32	0.419	0.6583	0.52	0.31	0.0119	0.4011	0.98	0.31	0.0083	0.3471	0.98				
rs9911832	A	0.32	-0.7136	0.653	0.27												

rs9912051	A	0.91	1.1978	1.4994	0.42								
rs9912092	A	0.29	0.7017	0.7141	0.33								
rs9912608	C	0.90	0.7197	1.2123	0.55								
rs9913009	A	0.86	0.1462	0.9489	0.88								
rs9913021	A	0.85	-2.514	0.9421	0.01	0.80	-0.0727	0.5121	0.89	0.81	-0.6798	0.4598	0.14
rs9913162	A	0.79	-0.1978	0.8217	0.81								
rs9914011	C	0.29	-1.2967	0.6837	0.06								
rs9914068	C	0.76	-0.557	0.7256	0.44	0.81	0.3275	0.4775	0.49	0.80	0.1391	0.4068	0.73
rs9914201	A	0.26	-1.1263	0.8215	0.17	0.19	0.3906	0.5276	0.46	0.20	0.0217	0.4519	0.96
rs9914874	A	0.47	-0.4959	0.614	0.42	0.49	0.3459	0.3783	0.36	0.49	0.0997	0.3257	0.76
rs9915000	A	0.05	-1.4828	1.5502	0.34	0.06	1.0372	0.82	0.21	0.05	0.7093	0.7379	0.34
rs9915162	A	0.86	-0.6584	0.9551	0.49								
rs9916285	A	0.52	-0.395	0.6269	0.53								
rs9916688	A	0.90	-0.3462	1.2884	0.79	0.90	-0.098	0.6844	0.89	0.90	-0.1592	0.6151	0.80
rs9916764	A	0.48	-0.2157	0.6279	0.73								
rs9916886	A	0.53	-0.9872	0.6403	0.12								
rs9944501	A	0.09	-0.4436	1.2562	0.72	0.12	-0.7424	0.6405	0.25	0.11	-0.724	0.5824	0.21
rs9988	A	0.43	-0.6822	1.3647	0.62								
rs9989484	A	0.86	0.1462	0.9489	0.88								

Table S7. Meta-analysis of sex-specific estimates for SBP – males

		Individuals genotyped in Panel 1				Individuals genotyped in Panel 2 but not in Panel 1 (replication)				Individuals genotyped in Panel 2 (includes individuals in Panel 1)				SHS Cohort (replication)
**SNP**	**Coded allele**	**Freq coded allele**	**beta**	**SE**	**P**	**Freq coded allele**	**beta**	**SE**	**P**	**Freq coded allele**	**beta**	**SE**	**P**	**Freq coded allele**
rs1000791	A	0.77	-1.9042	0.8615	0.027	0.78	0.5109	0.5323	0.337	0.78	0.0369	0.4689	0.937	
rs1000821	A	0.38	-0.8683	0.8602	0.313									
rs1002068	C	0.38	-0.0182	0.8005	0.982	0.41	-0.3096	0.4655	0.506	0.40	-0.302	0.417	0.469	
rs1004467	A	0.79	-0.0044	1.0362	0.997	0.78	-0.7067	0.5426	0.193	0.79	-0.6889	0.495	0.164	
rs1006809	A	0.19	0.7938	1.0243	0.438									
rs10073	A	0.76	-0.2890	0.8622	0.738	0.74	-1.0031	0.5208	0.054	0.74	-0.5648	0.4598	0.219	
rs1007464	A	0.54	0.7291	0.7738	0.346	0.50	-0.5079	0.4447	0.253	0.51	-0.7535	0.393	0.055	
rs1007850	A	0.82	0.9391	1.0728	0.381									
rs1008177	A	0.48	0.3364	0.8152	0.680									
rs1010161	A	0.47	0.2512	0.7862	0.749									
rs1010774	A	0.65	-0.5301	0.8414	0.529									
rs1012117	A	0.22	1.9226	1.0059	0.056									
rs1013013	A	0.15	-1.9294	1.2237	0.115									
rs1014390	A	0.13	2.0526	1.5524	0.186									
rs1028060	A	0.77	0.0170	1.0694	0.987	0.77	0.0367	0.5371	0.946	0.78	0.0587	0.4858	0.904	
rs10338	A	0.62	0.576	0.8423	0.494									
rs103550	C	0.58	0.3923	0.8378	0.640									
rs10401019	A	0.47	0.1156	0.8096	0.887									
rs1042393	A	0.57	0.3539	0.7316	0.629	0.62	-0.843	0.4421	0.057	0.62	-0.579	0.3988	0.147	
rs1042395	A	0.43	0.2654	0.7281	0.716	0.38	-0.8526	0.4413	0.053	0.39	-0.6191	0.3973	0.119	
rs1042396	A	0.31	0.4256	0.857	0.620	0.27	-1.5962	0.5507	0.004	0.28	-1.0701	0.4922	0.030	
rs1042397	A	0.36	0.4451	0.7632	0.560	0.32	-1.2026	0.4649	0.010	0.32	-0.7653	0.4176	0.067	
rs1042489	A	0.55	-0.9842	0.814	0.227									
rs1042542	A	0.41	0.5436	0.8498	0.522									
rs1044228	A	0.45	0.751	0.8165	0.358									
rs1044282	A	0.51	-0.4883	0.8349	0.559									
rs1044433	A	0.36	-0.8137	0.8123	0.317									
rs10445219	C	0.89	1.2511	1.3952	0.370	0.87	-0.5091	0.6844	0.457	0.87	-0.0285	0.6299	0.964	

rs10445220	A	0.27	-1.4686	0.9266	0.113	0.25	0.1016	0.5253	0.847	0.25	-0.2629	0.4618	0.569	
rs10445407	A	0.33	0.343	0.8634	0.691									
rs1046446	A	0.33	-0.5587	0.8522	0.512									
rs1047743	C	0.43	0.3528	0.8328	0.672									
rs1048775	C	0.74	-0.916	0.956	0.338									
rs10491334	A	0.09	-0.5984	1.5791	0.705	0.07	-0.0853	0.9086	0.925	0.07	0.1137	0.7962	0.887	
rs10493340	A	0.64	-0.9343	0.9211	0.311	0.70	0.7334	0.4914	0.136	0.70	0.3003	0.4424	0.497	
rs10495809	A	0.18	0.8154	1.0783	0.450	0.20	-0.8007	0.5702	0.160	0.19	-0.4034	0.515	0.434	
rs10512597	A	0.52	0.6452	0.8166	0.430									
rs10512601	C	0.18	-0.5578	1.1008	0.612									
rs10512604	A	0.29	1.9713	0.9698	0.042									
rs10512613	A	0.74	0.8597	0.9218	0.351									
rs10512617	C	0.44	0.6208	0.8202	0.449									
rs1055086	A	0.31	-0.4051	0.862	0.638	0.27	-0.1397	0.5028	0.781	0.28	-0.202	0.4423	0.648	
rs1055129	A	0.76	-1.1518	1.0089	0.254									
rs1057040	A	0.43	0.6105	0.8161	0.454									
rs1062935	A	0.40	-0.4202	0.8272	0.611									
rs1065768	A	0.36	-2.7694	1.7535	0.114									
rs1071664	A	0.29	0.3115	0.9965	0.755									
rs10775361	A	0.11	-0.9102	1.3296	0.494	0.11	-1.3536	0.7442	0.069	0.11	-1.4277	0.6667	0.032	
rs10775365	A	0.43	-1.7181	0.8647	0.047									
rs1077693	A	0.22	-0.8009	0.9813	0.414	0.27	0.1652	0.5212	0.751	0.25	0.0944	0.4704	0.841	
rs1079133	A	0.18	-1.6305	1.6593	0.326									
rs10852766	A	0.46	0.1406	0.799	0.860									
rs10852778	A	0.24	0.4195	0.9167	0.647									
rs10871489	A	0.89	1.5904	1.4914	0.286									
rs11014166	A	0.11	-1.283	1.4032	0.361	0.12	-1.4225	0.7636	0.062	0.11	-1.2363	0.6846	0.071	
rs11024074	A	0.63	0.5805	0.8659	0.503	0.67	0.4193	0.4966	0.399	0.66	0.4429	0.4413	0.316	
rs1106221	C	0.96	0.0186	2.5968	0.994	0.98	-1.0055	2.6574	0.705	0.98	-0.1953	1.5382	0.899	
rs1106281	A	0.93	1.7512	1.7978	0.330	0.85	0.2182	0.69	0.752	0.86	0.5892	0.646	0.362	
rs11065987	A	0.80	-2.0485	1.2615	0.104	0.86	-0.2801	0.6665	0.674	0.85	-0.4944	0.6072	0.416	
rs1106645	A	0.28	2.0762	0.8138	0.011	0.28	-0.7073	0.5126	0.168	0.27	0.076	0.4554	0.868	0.23
rs11077405	A	0.78	-0.0082	1.0142	0.994									
rs11077409	A	0.49	-2.0116	0.7555	0.008	0.49	0.1641	0.4604	0.722	0.50	-0.405	0.4092	0.322	

rs11077410	A	0.51	-1.8609	0.734	0.011	0.51	0.186	0.4596	0.686	0.50	-0.3448	0.4053	0.395	
rs11077416	A	0.32	0.5085	0.9145	0.578	0.27	0.5817	0.5129	0.257	0.28	0.5969	0.4535	0.188	0.28
rs11077420	A	0.23	-0.8153	0.9759	0.404	0.22	0.042	0.5606	0.940	0.22	-0.06	0.4978	0.904	
rs11077421	A	0.42	0.1747	0.8211	0.832	0.48	-0.0104	0.4584	0.982	0.47	0.0123	0.4062	0.976	0.44
rs11077426	A	0.39	-0.2116	0.8467	0.803									
rs11077427	A	0.64	0.662	0.8282	0.424	0.63	-0.1318	0.4902	0.788	0.64	-0.0715	0.4387	0.871	
rs11077428	A	0.45	0.2053	0.756	0.786	0.47	-0.0941	0.4597	0.838	0.46	-0.1719	0.4051	0.671	0.45
rs11077435	A	0.97	0.1719	3.287	0.958	0.96	0.2634	1.1709	0.822	0.96	0.442	1.0863	0.684	
rs11077441	A	0.28	-0.2671	0.8521	0.754	0.26	-0.2881	0.5025	0.566	0.27	-0.4208	0.4523	0.352	
rs11077628	A	0.08	-1.0661	2.1819	0.625	0.04	-1.5281	1.0872	0.160	0.05	-1.4983	0.9542	0.116	
rs11077637	A	0.55	-0.0904	0.7986	0.910	0.53	-1.02	0.4503	0.024	0.53	-0.7351	0.3978	0.065	
rs11077736	A	0.25	0.3765	1.0125	0.710									
rs11077772	A	0.11	-2.7281	1.4346	0.057									
rs11077773	A	0.87	-2.5117	1.2009	0.036									
rs11077787	C	0.09	-2.8252	1.5961	0.077									
rs11077793	A	0.33	-0.9621	0.9319	0.302									
rs11077799	A	0.78	-0.0637	0.9542	0.947									
rs11077813	A	0.71	0.223	0.9034	0.805									
rs11077815	A	0.50	-0.08	0.8301	0.923									
rs11077817	A	0.44	-0.3831	0.8426	0.649									
rs11077829	A	0.17	0.6963	1.0414	0.504									
rs11077874	A	0.49	-0.5475	0.8079	0.498									
rs11077876	A	0.63	-1.0136	0.8488	0.232									
rs11077879	A	0.26	0.8172	0.9253	0.377									
rs11077880	A	0.61	-0.1669	0.8217	0.839									
rs11077892	A	0.26	0.9649	0.9207	0.295									
rs11077909	A	0.85	-0.3168	1.1766	0.788									
rs11077919	A	0.68	0.2482	0.9639	0.797	0.71	-0.4188	0.5244	0.425	0.71	-0.2409	0.4601	0.601	
rs11077938	A	0.77	-1.6493	1.0049	0.101	0.74	0.0629	0.517	0.903	0.75	-0.2974	0.4629	0.521	
rs11077964	A	0.56	-0.587	0.8199	0.474									
rs11077969	A	0.78	-1.091	0.9335	0.243									
rs1108355	A	0.07	-2.533	1.7971	0.159									
rs1108365	A	0.59	0.817	0.823	0.321									
rs1108366	A	0.26	0.027	0.9539	0.977									

rs1109033	A	0.09	-2.8252	1.5961	0.077									
rs1110274	A	0.44	1.1232	0.8224	0.172									
rs1110734	A	0.64	-0.4603	0.7963	0.563	0.61	-0.415	0.4569	0.364	0.62	-0.2245	0.4123	0.586	
rs1113758	A	0.72	1.2058	0.9076	0.184									
rs11150736	A	0.12	-0.5671	1.3821	0.682									
rs11150738	A	0.92	0.113	1.4955	0.940									
rs11150739	A	0.93	0.5663	1.6767	0.736									
rs11150746	A	0.80	0.7483	1.0113	0.459									
rs11150780	A	0.78	0.0869	0.9742	0.929									
rs11150784	A	0.66	0.8869	0.8212	0.280									
rs11150821	A	0.40	0.8272	0.8188	0.312	0.33	0.5575	0.4756	0.241	0.35	0.4476	0.4211	0.288	0.37
rs11150822	A	0.38	0.2756	0.7783	0.723	0.33	0.5274	0.4731	0.265	0.34	0.3595	0.4194	0.391	
rs11150823	A					0.03	-1.3092	1.3416	0.329	0.03	-0.6049	1.2519	0.629	
rs11150824	A	0.55	0.2584	0.7628	0.735	0.46	-0.0236	0.4528	0.958	0.48	-0.0541	0.4038	0.894	0.50
rs11150827	A	0.18	1.8992	1.0218	0.063	0.16	-0.6272	0.6506	0.335	0.16	0.1668	0.5718	0.771	
rs11150847	A	0.39	0.4102	0.8831	0.642									
rs11150849	C	0.70	0.1566	0.9236	0.865									
rs1115834	A	0.11	3.3303	1.3542	0.014	0.16	-1.5641	0.6216	0.012	0.15	-0.3601	0.5704	0.528	0.12
rs11191548	A	0.80	-0.0002	1.0368	1.000	0.79	-0.7016	0.5544	0.206	0.80	-0.6726	0.4988	0.178	
rs1124736	A	0.16	-0.4793	1.0753	0.656	0.17	-1.4277	0.596	0.017	0.17	-1.0702	0.5345	0.045	
rs1126690	A	0.48	1.0752	0.7751	0.165									
rs1127678	A	0.11	-1.6476	1.7252	0.340									
rs1128889	A	0.38	0.123	0.8047	0.879									
rs11308563	A	0.40	0.824	0.8286	0.320	0.46	0.2298	0.4633	0.620	0.45	0.1707	0.4119	0.679	
rs1137582	C	0.46	0.1406	0.799	0.860									
rs11646213	A	0.33	1.3289	0.8677	0.126	0.36	-0.4939	0.4689	0.292	0.35	0.1161	0.4196	0.782	
rs11649977	A	0.23	-1.9842	0.8384	0.018	0.16	0.327	0.6042	0.588	0.18	-0.2576	0.5152	0.617	
rs11650154	A	0.68	0.5085	0.9145	0.578	0.73	0.5683	0.5143	0.269	0.72	0.5852	0.4545	0.198	0.96
rs11650313	A	0.88	-0.006	1.1846	0.996	0.81	0.1981	0.5789	0.732	0.82	0.3273	0.5351	0.541	
rs11650316	A	0.76	0.6181	0.9394	0.511									
rs11650345	A	0.07	-2.4607	2.0818	0.237	0.06	-2.0809	1.0546	0.048	0.06	-2.0166	0.9491	0.034	
rs11650709	A	0.57	0.3259	0.8108	0.688	0.62	-0.9694	0.475	0.041	0.60	-0.6872	0.4178	0.100	0.58
rs11650784	A	0.60	0.6598	0.7922	0.405	0.63	-1.025	0.4836	0.034	0.61	-0.6565	0.4273	0.124	
rs11651201	A	0.61	-0.7646	0.7488	0.307	0.58	0.4151	0.4455	0.351	0.59	0.2129	0.395	0.590	0.58

rs11651204	A	0.60	0.251	0.7957	0.752	0.58	0.4651	0.4483	0.300	0.58	0.2689	0.3972	0.498	
rs11651302	A	0.93	-2.6857	2.0897	0.199	0.93	-0.6953	0.9806	0.478	0.94	-1.2778	0.877	0.145	
rs11651514	C	0.06	-1.0944	2.6711	0.682									
rs11651587	A	0.77	2.8356	0.9793	0.004									
rs11651611	A	0.93	-2.0772	2.0546	0.312	0.94	-2.0023	1.0506	0.057	0.94	-1.8762	0.9435	0.047	
rs11651690	A	0.93	-2.0772	2.0546	0.312	0.94	-2.0023	1.0506	0.057	0.94	-1.8762	0.9435	0.047	
rs11651707	A	0.20	1.5441	1.0113	0.127									
rs11652075	A	0.29	0.5834	0.9432	0.536									
rs11652082	A	0.52	-0.8699	0.8609	0.312									
rs11652856	C	0.16	-0.0646	1.0668	0.952									
rs11652975	A	0.60	1.0183	0.8321	0.221									
rs11652985	C	0.38	-0.8683	0.8602	0.313									
rs11652993	C	0.39	-0.0517	0.8435	0.951									
rs11653700	A	0.17	2.8612	1.0988	0.009	0.17	-0.6104	0.6331	0.335	0.16	0.2087	0.5607	0.710	0.14
rs11654115	A	0.34	1.1096	0.849	0.191									
rs11654261	A	0.80	-1.5518	0.9448	0.101	0.82	0.2565	0.5952	0.667	0.82	-0.314	0.5135	0.541	
rs11654508	A	0.30	0.162	0.922	0.861									
rs11654770	A	0.37	-0.584	0.7734	0.450	0.36	0.5499	0.4701	0.242	0.36	0.2676	0.4204	0.524	
rs11654881	A	0.25	1.0368	0.9272	0.264									
rs11655167	A	0.74	-1.6551	0.8064	0.040	0.81	0.4514	0.5807	0.437	0.79	-0.1163	0.4933	0.814	
rs11655435	C	0.93	0.5663	1.6767	0.736									
rs11655455	A	0.76	0.2931	0.9783	0.765	0.76	-0.5781	0.5579	0.300	0.77	-0.2577	0.5143	0.616	
rs11655650	A	0.45	-0.9842	0.814	0.227									
rs11656298	A	0.78	-0.6411	0.9203	0.486	0.79	0.7055	0.5579	0.206	0.78	0.3102	0.4916	0.528	
rs11656673	A	0.14	1.3522	1.404	0.336	0.09	-0.5459	0.816	0.504	0.10	0.076	0.7086	0.915	
rs11656929	A	0.15	0.3834	1.761	0.828	0.11	1.5679	0.8561	0.067	0.11	1.2474	0.7484	0.096	0.11
rs11657098	A	0.21	-0.18	0.9527	0.850									
rs11657217	C	0.18	0.0943	1.1519	0.935									
rs11657360	C	0.50	-1.2491	0.8455	0.140									
rs11657440	A	0.86	0.1958	1.2315	0.874	0.87	-0.3997	0.6748	0.554	0.86	-0.1027	0.6089	0.866	
rs11657500	A	0.81	0.6545	1.0661	0.539	0.83	0.0385	0.5936	0.948	0.83	0.0847	0.5237	0.872	
rs11657630	A	0.79	0.9598	0.972	0.323									
rs11657635	A	0.32	-0.0205	0.8949	0.982									
rs11657655	A	0.22	2.0418	0.9967	0.041									

rs11657933	C	0.29	0.0938	0.9306	0.920	0.29	-0.6741	0.5243	0.199	0.28	-0.4336	0.4855	0.372
rs11658052	A	0.11	-0.9311	1.4036	0.507	0.16	-0.1069	0.6359	0.867	0.14	-0.1018	0.5867	0.862
rs11658299	A	0.61	0.719	0.8297	0.386								
rs11658442	A	0.08	0.1504	2.4583	0.951	0.03	-0.5867	1.4418	0.684	0.04	-0.2481	1.2258	0.840
rs11658622	A	0.46	0.7644	0.798	0.338								
rs11658680	A	0.39	0.084	0.7538	0.911	0.44	0.1954	0.4581	0.670	0.42	0.1402	0.405	0.729
rs11658702	A	0.73	-1.1607	0.8269	0.160	0.73	0.325	0.528	0.538	0.73	-0.2183	0.4557	0.632
rs11701424	A					0.01	1.2215	3.3713	0.717	0.01	1.2706	3.342	0.704
rs11775334	A	0.36	-0.3109	0.9048	0.731	0.38	0.105	0.46	0.819	0.38	0.001	0.4086	0.998
rs11867344	A	0.68	-1.317	0.8713	0.131								
rs11867708	A	0.81	-0.7662	1.0804	0.478								
rs11867785	A	0.21	1.1747	0.9798	0.231								
rs11868442	A	0.13	-2.0905	1.2915	0.106								
rs11868471	A	0.27	1.201	0.909	0.186								
rs11869115	A	0.71	-0.859	0.9254	0.353								
rs11869363	A	0.48	0.9927	1.0921	0.363								
rs11869453	A	0.07	0.5663	1.6767	0.736								
rs11869620	A	0.75	-0.1686	0.9921	0.865								
rs11869626	A	0.21	0.9598	0.972	0.323								
rs11869629	A	0.70	0.6991	0.9159	0.445								
rs11870238	A	0.18	0.9268	1.1372	0.415								
rs11870326	A	0.92	0.366	1.5154	0.809	0.93	-0.1423	0.89	0.873	0.93	0.2909	0.7996	0.716
rs11870711	A	0.25	0.4848	0.877	0.580	0.24	0.6891	0.5362	0.199	0.25	0.6597	0.4699	0.160
rs11870849	A	0.06	-3.5484	3.0003	0.237								
rs11871056	A	0.27	0.8842	0.9234	0.338								
rs11871318	A	0.30	1.2927	0.8432	0.125	0.29	-0.0178	0.5005	0.972	0.29	0.2306	0.4485	0.607
rs11871435	A	0.05	-2.5576	2.6479	0.334	0.06	0.1781	1.0965	0.871	0.06	-0.1181	1.0253	0.908
rs11871595	A	0.23	0.8765	0.9775	0.370								
rs11871688	C	0.79	0.9598	0.972	0.323								
rs11891	A	0.07	-1.8867	2.2369	0.399								
rs11903	A	0.39	-0.7107	0.9533	0.456								
rs12046278	A	0.38	-1.5298	0.8375	0.068	0.36	-0.883	0.4664	0.058	0.36	-0.831	0.4223	0.049
rs12051618	A	0.67	0.7543	0.8522	0.376	0.73	0.6022	0.5078	0.236	0.71	0.6351	0.4503	0.158
rs12051619	A	0.32	0.5085	0.9145	0.578	0.27	0.5687	0.513	0.268	0.28	0.5872	0.4535	0.195

0.28

rs12051621	A					0.95	-0.2637	1.1394	0.817	0.96	-0.4142	1.0501	0.693	0.96
rs12051632	A	0.57	-0.3014	0.8135	0.711	0.53	0.2213	0.4426	0.617	0.54	0.0686	0.3963	0.863	
rs12051678	A	0.20	-0.4976	1.0591	0.639									
rs12051723	A	0.07	0.0775	1.7634	0.965									
rs12149977	A	0.61	0.6875	0.7771	0.376	0.59	0.6571	0.4733	0.165	0.59	0.6068	0.4197	0.148	
rs12150348	A	0.18	0.0302	1.0807	0.978									
rs12325869	C					0.02	5.5074	2.6742	0.039	0.02	5.4591	2.6618	0.040	
rs12430	A	0.28	0.2195	0.9209	0.812									
rs12449322	A	0.56	-1.3251	0.8594	0.123									
rs12449492	A	0.46	0.6603	0.8133	0.417									
rs12449669	A	0.50	-1.2491	0.8455	0.140									
rs12449676	A	0.72	0.1164	0.8995	0.897									
rs12449822	A	0.72	-0.1024	0.8165	0.900	0.74	-0.2161	0.5251	0.681	0.73	-0.1581	0.4558	0.729	
rs12449949	A	0.24	1.0513	0.9007	0.243	0.21	-0.7652	0.5571	0.170	0.22	-0.4308	0.4903	0.380	
rs12449970	A	0.34	-0.8966	0.9077	0.323	0.34	-0.3817	0.4785	0.425	0.35	-0.2246	0.4287	0.600	
rs12449983	A	0.47	-0.4345	0.7897	0.582									
rs12450030	A	0.38	0.2821	0.848	0.739	0.33	-0.2092	0.5171	0.686	0.34	-0.2109	0.4487	0.638	0.36
rs12450059	A	0.49	0.781	0.8112	0.336	0.45	-1.1489	0.4644	0.013	0.46	-0.5856	0.4107	0.154	0.48
rs12450128	A	0.57	-0.7481	0.7948	0.347									
rs12450239	A	0.40	-1.1325	0.7542	0.133	0.35	0.1884	0.4716	0.690	0.37	-0.186	0.4158	0.655	
rs12450432	A	0.21	0.2085	0.9823	0.832									
rs12450478	A	0.29	0.3063	0.9408	0.745									
rs12450588	A					0.03	-2.2243	1.3524	0.100	0.03	-1.1145	1.2484	0.372	
rs12450753	A	0.25	1.09	0.8934	0.223									
rs12450839	A	0.39	0.2571	0.7995	0.748	0.42	-0.1301	0.4665	0.780	0.41	-0.0855	0.417	0.838	
rs12450876	A	0.91	0.0024	1.469	0.999									
rs12450888	C	0.50	0.7907	0.8515	0.353									
rs12450989	A	0.21	1.083	0.9851	0.272									
rs12451047	A	0.42	0.2103	0.8083	0.795	0.41	-0.0569	0.4604	0.902	0.41	0.1507	0.4156	0.717	
rs12451318	A	0.05	1.1694	1.9609	0.551	0.09	0.2433	0.8068	0.763	0.09	0.5287	0.7344	0.472	
rs12451395	A	0.72	0.1025	0.8509	0.904	0.76	0.349	0.5503	0.526	0.74	0.3173	0.4783	0.507	
rs12451487	A	0.57	0.5577	0.8489	0.511									
rs12451560	A	0.50	-0.3224	0.7573	0.670	0.48	0.1502	0.4637	0.746	0.48	-0.0944	0.4142	0.820	
rs12451638	A	0.39	0.4499	0.781	0.565	0.33	0.5106	0.4718	0.279	0.35	0.3996	0.4181	0.339	

rs12451668	A	0.77	-0.6978	1.0481	0.506	0.78	-0.1956	0.5675	0.730	0.78	-0.4543	0.5073	0.371	0.75
rs12451808	A	0.30	-0.117	0.9452	0.902									
rs12452184	A	0.48	1.5679	0.8288	0.059									
rs12452616	A	0.25	0.5921	0.8423	0.482	0.21	-1.3264	0.5402	0.014	0.21	-0.7389	0.4811	0.125	
rs12452652	A	0.23	-0.0076	0.906	0.993									
rs12452661	A	0.77	-1.0728	0.8812	0.223	0.80	0.0947	0.579	0.870	0.79	-0.1992	0.504	0.693	
rs12452891	A	0.15	0.0331	1.2908	0.980									
rs12453011	A	0.57	-0.8921	0.8371	0.287									
rs12453034	A	0.09	0.0024	1.469	0.999									
rs12453207	A	0.28	-0.4069	0.903	0.652	0.23	0.2807	0.5493	0.609	0.24	0.2209	0.4798	0.645	0.25
rs12453606	A	0.72	-0.4535	0.9255	0.624	0.74	-0.3235	0.5072	0.524	0.73	-0.4541	0.4544	0.318	
rs12453678	A	0.26	-0.3143	0.8734	0.719	0.23	0.2617	0.5541	0.637	0.24	0.22	0.4866	0.651	
rs12453809	A	0.15	-0.6091	1.2218	0.618									
rs1254787	A	0.06	-0.1861	2.3021	0.936									
rs1254795	A	0.26	0.9579	0.9204	0.298									
rs12600564	A	0.14	0.4403	1.1678	0.706									
rs12600765	A	0.23	0.5144	0.8784	0.558	0.26	0.1476	0.5172	0.775	0.26	0.2433	0.4605	0.597	
rs12600868	A	0.57	0.3497	0.8417	0.678									
rs12600908	A	0.05	-1.5593	2.7207	0.567									
rs12601162	C	0.25	0.1529	1.109	0.890									
rs12601177	A	0.17	1.2051	1.186	0.310									
rs12601423	A	0.09	-0.22	1.4633	0.881									
rs12601803	A	0.30	1.879	0.9117	0.039									
rs12601898	A	0.19	0.1672	1.0676	0.876	0.22	-0.3117	0.5968	0.602	0.20	0.1819	0.5518	0.742	0.19
rs12601949	A	0.23	1.8248	1.1426	0.110									
rs12602330	A	0.91	-2.4195	2.2522	0.283	0.92	-0.8001	0.9435	0.396	0.92	-0.7854	0.8393	0.349	0.89
rs12602412	A	0.39	-0.5908	0.8019	0.461	0.38	0.6855	0.4742	0.148	0.38	0.3449	0.4178	0.409	
rs12602415	A	0.39	-0.4069	0.8023	0.612	0.38	0.6303	0.4747	0.184	0.38	0.3202	0.4179	0.444	
rs12602618	A	0.57	0.3323	0.7667	0.665	0.56	-0.4375	0.4631	0.345	0.56	-0.1951	0.4116	0.636	
rs12602885	A	0.08	0.5677	1.5544	0.715									
rs12603040	A	0.37	-0.0452	0.7848	0.954	0.39	0.5727	0.4679	0.221	0.39	0.2321	0.414	0.575	
rs12603074	A	0.09	-0.0137	1.4732	0.993									
rs12603194	A	0.41	-1.6568	0.8513	0.052									
rs12603265	A	0.78	1.2699	0.973	0.192									

rs12603697	A	0.96	-1.8785	3.0695	0.541	0.95	-1.4612	1.2132	0.228	0.95	-1.1186	1.1092	0.313	
rs12603748	A	0.96	-1.8785	3.0695	0.541	0.93	-0.8474	1.0098	0.401	0.94	-0.6435	0.913	0.481	
rs12604076	A	0.55	0.6136	0.8669	0.479									
rs12797	A	0.40	-1.0664	0.8556	0.213									
rs1285293	A	0.24	-0.2498	0.9505	0.793									
rs12935880	A	0.03	-2.8142	3.2645	0.389	0.02	-0.4219	1.8694	0.822	0.02	-1.2777	1.6988	0.452	0.02
rs12936076	A	0.14	0.8599	1.2581	0.494									
rs12936473	A	0.80	0.9888	1.0986	0.368									
rs12936687	A	0.18	1.1901	1.0912	0.275									
rs12937212	A	0.17	1.0151	1.1763	0.388	0.19	0.1086	0.5962	0.855	0.18	0.2591	0.5347	0.628	0.19
rs12937891	A	0.89	2.4199	1.1391	0.034	0.88	-0.4945	0.7097	0.486	0.88	0.2686	0.6306	0.670	
rs12938262	A	0.37	-0.6454	0.7889	0.413	0.41	-0.0437	0.455	0.923	0.40	-0.2007	0.4114	0.626	
rs12938422	C	0.39	-0.2903	0.7812	0.710	0.43	0.0467	0.4563	0.919	0.42	-0.0646	0.4102	0.875	
rs12938889	A	0.11	-2.6884	1.4243	0.059									
rs12939128	A					0.98	-0.3024	2.6078	0.908	0.98	-0.035	1.9736	0.986	
rs12939413	A	0.09	0.0024	1.469	0.999									
rs12939525	A	0.81	-0.9838	1.105	0.373	0.79	-0.7698	0.5993	0.199	0.79	-0.7915	0.5333	0.138	0.85
rs12939549	A	0.86	0.8599	1.2581	0.494									
rs12940068	A	0.91	-0.2936	1.4705	0.842									
rs12940226	A	0.55	0.4846	0.9791	0.621									
rs12940295	A	0.86	-0.7678	1.3986	0.583	0.84	-0.0171	0.6482	0.979	0.84	-0.1727	0.5924	0.771	0.86
rs12940302	A	0.36	-0.435	0.8591	0.613	0.41	-0.0895	0.4563	0.845	0.40	-0.2767	0.4127	0.503	
rs12940622	A	0.14	0.8599	1.2581	0.494									
rs12941504	A	0.32	-0.7566	0.847	0.372									
rs12941958	A	0.09	0.0024	1.469	0.999									
rs12941999	A	0.48	0.1502	0.791	0.849									
rs12942038	A	0.31	0.056	0.872	0.949	0.34	0.6708	0.4841	0.166	0.33	0.4373	0.4339	0.314	0.27
rs12942476	A	0.81	0.3655	0.975	0.708	0.80	0.1962	0.5793	0.735	0.80	0.2346	0.5157	0.649	
rs12943128	A	0.06	0.5611	2.4818	0.821									
rs12943136	C	0.47	-1.2495	0.828	0.131									
rs12943410	A	0.24	0.3908	0.8895	0.660	0.21	-0.4986	0.5536	0.368	0.22	-0.3955	0.4852	0.415	
rs12943496	C	0.13	1.1102	1.3223	0.401	0.15	-1.3614	0.6752	0.044	0.14	-0.6998	0.6155	0.256	0.11
rs12943617	A	0.50	1.1921	0.8052	0.139	0.43	0.0021	0.4577	0.996	0.44	-0.1651	0.4042	0.683	
rs12943620	A	0.13	0.7233	1.2662	0.568									

rs12944002	A	0.60	1.1851	0.7965	0.137	0.59	-0.2518	0.458	0.582	0.60	0.0231	0.4129	0.955	
rs12944016	A	0.74	0.4493	0.8833	0.611	0.75	0.7781	0.5284	0.141	0.75	0.6723	0.4683	0.151	
rs12944642	A	0.59	0.7781	0.8392	0.354	0.55	-0.0098	0.4686	0.983	0.56	0.0312	0.4167	0.940	
rs12944841	C	0.08	1.392	1.384	0.315	0.10	-0.6596	0.782	0.399	0.10	-0.0016	0.7166	0.998	
rs12944983	A	0.82	-0.2278	1.0987	0.836									
rs12945231	A	0.04	2.0751	3.0881	0.502									
rs12945425	A					0.98	-1.2351	2.5987	0.635	0.98	-0.4817	1.9335	0.803	
rs12945469	A	0.91	-2.6193	2.1468	0.222									
rs12945787	A	0.14	2.2283	1.1556	0.054	0.12	-1.5115	0.7149	0.035	0.12	-0.8724	0.6502	0.180	
rs12945963	A	0.50	0.0854	0.7568	0.910	0.49	0.3326	0.4586	0.468	0.50	0.2561	0.4064	0.529	
rs12946115	A	0.91	0.0024	1.469	0.999									
rs12946426	A	0.42	-0.6286	0.7961	0.430	0.44	0.1659	0.4553	0.716	0.43	0.1406	0.4101	0.732	0.43
rs12946454	A	0.85	-0.6202	1.4666	0.672	0.82	-0.0468	0.6297	0.941	0.83	0.0239	0.5863	0.968	
rs12946859	A	0.14	-0.36	1.6584	0.828	0.11	-0.8436	0.7697	0.273	0.12	-0.7122	0.6968	0.307	
rs12947653	A	0.92	0.113	1.4955	0.940									
rs12947901	A	0.91	-0.2936	1.4705	0.842									
rs12948040	A	0.91	0.0024	1.469	0.999									
rs12948969	A	0.07	-3.1133	2.1191	0.142	0.07	-0.6953	0.9806	0.478	0.06	-1.3423	0.8791	0.127	
rs12949063	A	0.86	-0.1595	1.1818	0.893									
rs12949118	A	0.62	0.3406	0.7638	0.656	0.59	0.6485	0.464	0.162	0.60	0.3702	0.4095	0.366	
rs12950039	A	0.36	-0.091	0.8431	0.914									
rs12950541	A	0.09	0.0024	1.469	0.999									
rs12950551	A	0.07	-3.1133	2.1191	0.142	0.06	-0.9687	0.9884	0.327	0.06	-1.5681	0.8845	0.076	
rs12950642	A	0.64	1.4728	0.867	0.089									
rs12950752	A	0.09	-2.8252	1.5961	0.077									
rs12951389	A	0.43	-0.354	0.7832	0.651	0.48	-0.1231	0.45	0.784	0.46	0.0201	0.4027	0.960	0.46
rs12951541	A	0.49	-1.4579	0.8447	0.084									
rs12951778	A	0.68	-0.0368	0.8614	0.966									
rs12952105	A	0.26	-0.584	0.9102	0.521	0.30	-0.314	0.5135	0.541	0.28	-0.2398	0.453	0.597	
rs12952612	A	0.45	-0.6368	0.7549	0.399	0.51	0.448	0.4332	0.301	0.49	0.3581	0.3906	0.359	
rs12952770	A	0.56	-0.4048	0.8519	0.635									
rs1317421	A	0.84	-1.4428	1.2425	0.246	0.86	0.7545	0.7071	0.286	0.86	0.5673	0.6394	0.375	
rs1318314	A	0.78	1.5886	1.0142	0.117	0.77	0.2876	0.5531	0.603	0.78	0.5989	0.5132	0.243	
rs1318315	A	0.26	-1.6926	0.8062	0.036	0.19	0.549	0.5935	0.355	0.21	-0.0692	0.5015	0.890	

rs13342272	A	0.81	0.2956	0.9717	0.761	0.78	-0.0709	0.534	0.894	0.79	0.0117	0.4822	0.981	
rs1369299	A	0.07	-3.0128	1.8154	0.097									
rs1378905	A	0.49	0.6251	0.811	0.441	0.45	-1.1579	0.464	0.013	0.45	-0.6429	0.4101	0.117	0.47
rs1378906	A	0.61	1.1304	0.7928	0.154	0.54	-0.2189	0.4647	0.638	0.56	0.17	0.411	0.679	
rs1378942	A	0.26	-0.4884	0.9385	0.603	0.26	-1.2083	0.5399	0.025	0.26	-0.929	0.491	0.059	
rs1384367	A	0.45	0.6962	0.8682	0.423									
rs1388512	A	0.70	-1.4999	0.9364	0.109									
rs1436138	A	0.57	0.5514	0.827	0.505									
rs14640	A	0.88	3.6733	1.2497	0.003									
rs1465983	A	0.26	0.7989	0.9237	0.387									
rs1466003	A	0.27	-0.6884	1.012	0.496									
rs1466681	A	0.89	-2.6884	1.4243	0.059									
rs1467979	A	0.09	-0.1845	1.2986	0.887	0.08	0.9867	0.8664	0.255	0.08	0.4688	0.75	0.532	
rs1468030	A	0.78	1.9226	1.0059	0.056									
rs1468032	A	0.23	1.5138	1.0045	0.132									
rs1468033	A	0.63	-0.5991	0.8714	0.492									
rs1468035	A	0.54	-0.8505	0.8138	0.296									
rs1470941	A	0.74	-0.5812	1.024	0.570									
rs1473312	A	0.35	-0.3929	0.8077	0.627									
rs1476788	A	0.18	0.1524	1.0955	0.889									
rs1485329	A	0.88	0.3884	1.3487	0.773									
rs1485330	A	0.07	0.634	1.6121	0.694									
rs1530440	A	0.27	-0.1882	0.9409	0.841	0.23	0.6619	0.5529	0.231	0.24	0.4681	0.4864	0.336	
rs1533570	A	0.43	-0.8921	0.8371	0.287									
rs1546407	A	0.78	-0.7865	0.9498	0.408									
rs1550186	A	0.83	0.6605	1.0212	0.518	0.83	-0.3545	0.6154	0.565	0.83	0.0369	0.5422	0.946	
rs1551619	A	0.23	-0.1695	1.1619	0.884									
rs1551858	A	0.26	-0.4025	0.9329	0.666									
rs1552173	A	0.47	1.2064	0.8575	0.160									
rs15538	A	0.52	-2.1185	0.7619	0.005	0.52	-0.1586	0.4672	0.734	0.51	-0.5795	0.4162	0.164	
rs1561810	A	0.09	-0.3299	1.3005	0.800	0.08	0.7319	0.873	0.402	0.08	0.2399	0.7531	0.750	
rs1561811	A	0.44	0.2897	0.7667	0.706	0.39	-1.0636	0.4524	0.019	0.41	-0.6959	0.405	0.086	
rs1563447	A	0.44	-0.2347	0.7393	0.751	0.41	0.0222	0.4611	0.962	0.42	-0.1928	0.4027	0.632	
rs1563448	A	0.14	-0.4602	1.7935	0.798	0.08	-1.0833	1.0184	0.288	0.09	-1.3958	0.8669	0.107	0.08

rs1563449	A	0.97	-2.7489	3.3595	0.413	0.94	-1.4085	1.1335	0.214	0.94	-0.7551	1.0175	0.458
rs1564868	A	0.19	0.7886	1.0142	0.437								
rs1567960	C	0.82	2.1837	1.0367	0.035								
rs1567962	A	0.26	1.0718	0.9217	0.245								
rs1568448	C	0.35	-0.3395	0.898	0.705								
rs1629034	A	0.77	0.5856	0.9979	0.557								
rs1631707	A	0.21	-0.6227	1.2959	0.631	0.14	0.0693	0.6727	0.918	0.16	-0.294	0.6013	0.625
rs1632673	A	0.21	-0.3418	1.3049	0.793	0.14	0.0546	0.6728	0.935	0.16	-0.2542	0.6021	0.673
rs164009	A	0.46	-0.0887	0.7991	0.912								
rs164106	A	0.54	-0.1992	0.7996	0.803								
rs1661714	A	0.27	-1.2784	0.9802	0.192								
rs1661721	A	0.28	-1.0381	0.9982	0.298								
rs1663196	A	0.23	-0.2213	0.8834	0.802	0.25	0.2416	0.5202	0.642	0.25	-0.0156	0.4644	0.973
rs1663199	A	0.19	-0.7038	1.124	0.531	0.14	0.0867	0.6719	0.897	0.15	-0.2284	0.6015	0.704
rs1671019	A	0.28	-1.5179	0.9184	0.098								
rs1671021	A	0.73	-1.3191	0.9424	0.162								
rs1671032	A	0.74	-1.2209	0.934	0.191								
rs1671033	C	0.69	-0.5958	0.8973	0.507								
rs1671036	A	0.34	-1.194	0.9076	0.188								
rs1675262	A	0.84	-0.313	1.0157	0.758	0.85	0.0612	0.6393	0.924	0.85	0.1059	0.5581	0.850
rs16948048	A	0.84	0.908	1.3405	0.498	0.87	-0.6536	0.6741	0.332	0.87	-0.0316	0.6065	0.958
rs1696754	A	0.21	-0.3418	1.3049	0.793	0.14	0.0447	0.6736	0.947	0.16	-0.2674	0.6027	0.657
rs16967789	A	0.05	-1.5593	2.7207	0.567								
rs16968129	C	0.32	-0.4805	0.9154	0.600								
rs16968692	A	0.72	1.0738	0.9334	0.250								
rs16968702	A	0.28	1.0738	0.9334	0.250								
rs16968960	A	0.60	-0.1505	0.8526	0.860								
rs16969138	A	0.88	-0.7464	1.3346	0.576								
rs16969262	A	0.81	1.045	1.2152	0.390								
rs16969510	A	0.37	0.3274	0.8485	0.700								
rs16970576	A	0.83	-0.1499	1.1758	0.899	0.84	0.6066	0.645	0.347	0.84	0.5137	0.5754	0.372
rs16970774	A	0.18	0.4536	1.3009	0.727								
rs16970784	A	0.20	-0.4738	1.1826	0.689								
rs16970787	A	0.80	-0.4976	1.0591	0.639								

rs16970792	A	0.32	-0.144	0.9083	0.874								
rs16970802	A	0.17	0.3668	1.3609	0.788								
rs16970803	A	0.81	-0.1117	1.0717	0.917								
rs16970811	A	0.83	0.3668	1.3609	0.788								
rs16971269	C	0.90	1.1986	1.8753	0.523								
rs16971526	A	0.88	0.5378	1.3756	0.696								
rs16971682	A	0.60	0.4268	0.8225	0.604								
rs16972227	A	0.70	-1.7015	0.7857	0.030	0.73	0.474	0.4914	0.335	0.72	-0.08	0.4334	0.854
rs16978176	A	0.59	-1.8986	0.8519	0.026								
rs16978193	A	0.82	-0.1991	1.1073	0.857								
rs16978197	A	0.18	-0.8184	1.1065	0.460								
rs16978198	A	0.81	-0.636	1.0684	0.552								
rs16982520	A					0.95	0.3787	1.2798	0.767	0.96	1.8473	1.1223	0.100
rs1699607	A	0.36	-0.3196	0.8332	0.701								
rs1708861	A	0.20	0.8633	1.0544	0.413	0.20	0.7154	0.5838	0.221	0.20	0.6196	0.51	0.224
rs17248007	A	0.86	0.4201	1.2662	0.740	0.90	-0.552	0.7705	0.474	0.89	-0.4983	0.6611	0.451
rs17248371	A					0.03	1.1159	1.4731	0.449	0.03	0.9323	1.3759	0.498
rs17249754	A	0.13	-3.7553	1.7812	0.035	0.10	1.1583	0.8674	0.182	0.10	0.2647	0.7924	0.738
rs17367504	A	0.86	-0.4736	1.3283	0.721	0.88	-0.8095	0.6907	0.241	0.88	-0.8049	0.6275	0.200
rs17545956	A	0.25	-0.2405	0.947	0.800	0.25	-1.205	0.545	0.027	0.24	-0.8287	0.4853	0.088
rs17553512	A	0.34	0.4684	0.8861	0.597								
rs17557592	C	0.77	0.0236	0.9852	0.981								
rs17561950	A	0.62	1.548	0.8089	0.056								
rs17581498	A	0.93	-2.2612	1.7905	0.207								
rs17614280	A	0.41	0.0219	0.8379	0.979								
rs17632208	A	0.86	-0.8047	1.4232	0.572	0.88	-0.1849	0.7245	0.799	0.88	-0.3183	0.6495	0.624
rs17656528	A	0.90	-0.3746	1.4646	0.798								
rs17657522	A	0.41	0.7608	0.8191	0.353								
rs17657767	A	0.45	0.6962	0.8682	0.423								
rs17736494	A	0.45	0.6962	0.8682	0.423								
rs17739056	A	0.59	-0.7795	0.7895	0.324								
rs17742707	A	0.63	1.6272	0.8323	0.051	0.65	-0.2402	0.4777	0.615	0.65	0.1675	0.421	0.691
rs1800299	A					0.02	-1.7138	1.9039	0.368	0.02	-0.2864	1.9337	0.882
rs1800303	A	0.04	-1.4654	3.0522	0.631	0.02	0.0137	1.8466	0.994	0.02	-1.159	1.577	0.462

rs1800304	A	0.41	0.4579	0.7223	0.526	0.37	-0.8655	0.4448	0.052	0.38	-0.5546	0.3999	0.166	
rs1800305	A	0.04	-1.4654	3.0522	0.631	0.02	0.1681	1.8533	0.928	0.02	-1.0405	1.5814	0.511	
rs1800307	A	0.06	-0.9445	2.693	0.726	0.02	3.3708	1.6285	0.038	0.02	2.1588	1.3401	0.107	
rs1800310	A	0.88	0.6322	1.1727	0.590	0.89	0.6715	0.7354	0.361	0.89	0.5726	0.6429	0.373	
rs1868821	A	0.34	0.1464	0.8611	0.865									
rs1869932	A	0.71	-1.927	0.7958	0.015	0.72	0.261	0.5005	0.602	0.72	-0.2577	0.4411	0.559	
rs1869934	A	0.37	-0.448	0.8842	0.612	0.30	0.1283	0.495	0.796	0.32	-0.0163	0.4381	0.970	
rs1870625	A	0.34	1.5775	0.7581	0.037	0.34	-0.6455	0.4544	0.156	0.34	-0.2341	0.4125	0.570	
rs1870990	A	0.25	0.3838	1.0088	0.704									
rs1871935	A	0.50	-1.5715	0.8406	0.062									
rs1873588	A	0.35	0.1798	0.9161	0.844									
rs1877677	A	0.67	-1.8215	0.8052	0.024	0.71	-0.0547	0.5127	0.915	0.70	-0.3026	0.4529	0.504	
rs1877926	A	0.23	1.9112	0.9922	0.054									
rs1878061	A	0.34	-0.1321	0.8958	0.883									
rs1879455	C	0.45	-1.1109	0.8477	0.190									
rs1879967	A	0.20	1.2416	1.0406	0.233									
rs1879968	A	0.34	0.4567	0.8882	0.607									
rs1905160	A	0.05	1.6722	2.1791	0.443									
rs1918974	A	0.82	0.9925	1.1181	0.375	0.81	-0.3472	0.5693	0.542	0.82	0.1439	0.5148	0.780	
rs1963982	A	0.57	-0.7979	0.9049	0.378	0.54	-0.0179	0.4574	0.969	0.55	-0.0538	0.4167	0.897	
rs1965780	A	0.53	1.7485	0.7965	0.028	0.54	-0.5787	0.4536	0.202	0.54	0.0452	0.4073	0.912	
rs1976492	A	0.33	0.3421	0.8731	0.695									
rs1993681	C	0.73	-0.2556	0.8525	0.764	0.69	-0.0234	0.4963	0.962	0.71	-0.1021	0.4477	0.820	
rs1997978	A	0.48	0.4931	0.8062	0.541									
rs2002573	A	0.22	-0.0584	1.052	0.956									
rs2003241	A	0.95	0.7807	2.0915	0.709									
rs2004381	A	0.62	-0.2073	0.784	0.792	0.56	-0.0606	0.4594	0.895	0.58	-0.057	0.4131	0.890	0.60
rs2004887	A	0.90	-1.2011	1.9067	0.529	0.94	-0.7521	1.1075	0.497	0.94	-0.7115	0.9574	0.457	
rs2009196	C	0.83	-1.469	1.098	0.181									
rs2013350	A	0.74	-1.7039	0.804	0.034	0.81	0.4542	0.587	0.439	0.79	-0.1393	0.497	0.779	
rs2014989	A	0.21	-0.775	0.8971	0.388	0.18	0.0675	0.5931	0.909	0.19	-0.1088	0.5152	0.833	
rs2016126	A	0.45	-1.1109	0.8477	0.190									
rs2028551	A	0.23	0.0045	1.0622	0.997									
rs2033715	A	0.54	1.0564	0.7878	0.180	0.51	-0.3122	0.4515	0.489	0.52	0.6008	0.4006	0.134	

rs2034310	A	0.30	-2.6022	0.8571	0.002								
rs2035181	C	0.26	0.9066	0.8893	0.308								
rs2044102	A	0.58	0.2061	0.8275	0.803								
rs2044103	A	0.45	0.6038	0.783	0.441								
rs2048058	A	0.22	2.458	1.2204	0.044								
rs2048753	A	0.06	3.2304	1.7773	0.069								
rs2053156	A	0.93	-2.533	1.7971	0.159								
rs2053159	A	0.09	-2.8252	1.5961	0.077								
rs2053160	A	0.09	-2.7239	1.6285	0.094								
rs2053508	A	0.51	1.1786	0.8275	0.154								
rs2053748	A	0.45	-1.1068	0.8491	0.192								
rs2056439	C	0.78	-1.2331	0.9715	0.204								
rs2060097	A					0.96	0.6438	1.2688	0.612	0.97	0.657	1.2551	0.601
rs2063785	A	0.92	3.5422	2.0678	0.087								
rs2066964	C	0.67	1.0078	0.8774	0.251								
rs2069528	A	0.83	-2.2871	1.109	0.039								
rs2069534	A	0.42	1.3444	0.8054	0.095								
rs2069536	A	0.16	-2.1921	1.1718	0.061								
rs2070871	A	0.22	-0.0229	0.9849	0.981								
rs2071148	A	0.43	-0.2872	0.8319	0.730								
rs2071192	A	0.34	-1.0531	0.848	0.214								
rs2071195	A	0.36	0.1928	0.8283	0.816								
rs2071214	A	0.79	-1.1844	1.6008	0.459								
rs2071654	A	0.23	1.9279	1.1438	0.092								
rs2073285	A	0.51	0.3988	0.7956	0.616								
rs2076949	A	0.87	0.5287	1.307	0.686	0.85	-0.0985	0.6999	0.888	0.87	0.3425	0.657	0.602
rs2077948	A	0.26	0.8597	0.9218	0.351								
rs2085351	C	0.83	0.127	1.0493	0.904	0.78	0.3248	0.5281	0.539	0.79	0.4171	0.4866	0.391
rs2090204	A	0.04	0.033	2.733	0.990								
rs2090205	A	0.09	-0.2699	1.9961	0.892								
rs2100896	A	0.63	-1.1692	0.8372	0.163								
rs2120886	A	0.22	1.6906	0.9477	0.074								
rs2121070	A	0.07	-1.7624	2.18	0.419	0.07	-0.4688	0.901	0.603	0.07	-0.347	0.8271	0.675
rs2124603	A	0.58	-0.251	0.7845	0.749	0.54	0.2293	0.46	0.618	0.56	0.3524	0.4085	0.388

rs2124604	A	0.53	-1.4481	0.7967	0.069	0.54	0.4566	0.4549	0.316	0.54	-0.0325	0.4078	0.936	
rs2124606	A	0.72	-0.8534	0.8895	0.337	0.81	0.3059	0.5864	0.602	0.79	-0.2554	0.4983	0.608	
rs2125345	A	0.57	0.4414	0.8469	0.602									
rs2131049	A	0.50	2.5761	0.7916	0.001									
rs2138125	A	0.87	1.52	1.1606	0.190									
rs2138126	A	0.87	1.52	1.1606	0.190									
rs2139461	A	0.26	0.8699	0.9239	0.346									
rs2165994	A	0.76	-0.3617	0.9355	0.699	0.78	0.0595	0.5515	0.914	0.78	-0.0072	0.4928	0.988	
rs2166703	A	0.45	-1.0801	0.8596	0.209									
rs2240769	A	0.49	-0.2781	0.8006	0.728									
rs2241886	A	0.20	1.4318	0.9412	0.128									
rs2242462	C	0.34	0.4587	0.8869	0.605									
rs2243538	A	0.43	0.7106	0.8088	0.380									
rs2244377	A	0.34	-1.0658	0.8473	0.208									
rs2246632	A	0.57	0.7086	0.8075	0.380									
rs2247007	A	0.43	0.7306	0.8111	0.368									
rs2255166	A	0.28	-0.4195	0.8919	0.638									
rs2256881	A	0.07	-0.1614	1.58	0.919	0.07	1.7081	0.8814	0.053	0.07	1.1307	0.8107	0.163	
rs2257020	A	0.43	1.0496	0.8788	0.232									
rs2269374	A	0.57	-0.4336	0.8282	0.601									
rs2271090	A	0.73	-0.2554	0.931	0.784									
rs2271602	A	0.22	2.0418	0.9967	0.041									
rs2277698	A	0.13	-2.0046	1.3316	0.132									
rs2277700	A	0.83	-1.2824	1.1093	0.248									
rs2278826	A	0.67	0.0929	0.8824	0.916									
rs2279052	C	0.68	0.8066	0.8466	0.341									
rs2279053	A	0.68	0.8066	0.8466	0.341									
rs2279056	A	0.54	0.456	0.8226	0.579									
rs2279308	A	0.51	-1.1317	0.8296	0.173									
rs2279914	A	0.06	0.9212	2.1173	0.664	0.05	-2.2581	1.1923	0.058	0.05	-1.9654	1.0401	0.059	0.05
rs2279920	A	0.04	1.2222	3.1087	0.694	0.05	-2.1956	1.1864	0.064	0.05	-1.2217	1.0227	0.232	0.05
rs2280147	A	0.49	0.1519	1.6327	0.926									
rs2280177	A	0.06	-0.3143	2.5142	0.901									
rs2280269	A	0.26	0.9692	0.9149	0.289									

rs2280271	A	0.03	-0.9603	3.4545	0.781									
rs2286586	A	0.51	-1.1102	0.8122	0.172									
rs2286593	A	0.30	0.4971	0.9188	0.589									
rs2289527	C	0.86	-0.3243	1.2007	0.787	0.87	0.4544	0.706	0.520	0.87	0.1717	0.6243	0.783	
rs2289529	A	0.09	0.5995	1.6546	0.717	0.08	-0.8997	0.8567	0.294	0.08	-0.6907	0.7874	0.380	
rs2289531	A	0.04	-0.0231	2.6231	0.993	0.03	-1.0865	1.5894	0.494	0.03	-1.0346	1.3513	0.444	
rs2289535	A	0.73	0.3046	0.9495	0.748									
rs2289536	A					0.02	-2.5722	3.0192	0.394	0.01	-1.3203	3.4486	0.702	
rs2289603	A	0.27	1.4903	0.9639	0.122									
rs2289606	A	0.72	0.965	0.9302	0.300									
rs2289728	A	0.23	-0.6701	0.9806	0.494									
rs2289751	C	0.44	0.114	0.8211	0.890									
rs2289759	A	0.80	1.424	1.0183	0.162									
rs2289762	A	0.20	1.5441	1.0113	0.127									
rs2290251	A	0.61	0.8858	0.8059	0.272									
rs2290455	A	0.14	0.7267	1.2709	0.568									
rs2290460	A	0.30	0.4934	0.9545	0.605									
rs2290566	A					0.02	0.9974	1.6557	0.547	0.02	1.616	1.4921	0.279	0.04
rs2290769	C	0.86	-2.546	1.241	0.040									
rs2290771	A	0.69	-1.4359	0.9075	0.114									
rs2290906	A	0.68	-0.1311	0.9109	0.886									
rs2290907	A	0.67	-0.0799	0.9216	0.931									
rs2291021	A	0.53	-1.2641	1.7002	0.457									
rs2291028	A	0.47	1.1377	0.898	0.205									
rs2291359	A	0.72	0.9375	0.889	0.292									
rs2292184	A	0.73	-0.121	0.9422	0.898									
rs2292642	A	0.65	1.5354	0.8341	0.066									
rs2292645	A	0.91	-0.4556	2	0.820									
rs2293189	A	0.77	0.3921	0.9244	0.671									
rs2293192	A	0.77	0.3788	0.9169	0.680									
rs2304830	A	0.53	-0.1217	0.7956	0.878									
rs2304852	A	0.84	-0.2046	0.9982	0.838	0.84	0.1054	0.6382	0.869	0.84	-0.0622	0.559	0.911	
rs2304854	A	0.84	-0.0759	0.9978	0.939	0.84	0.1193	0.6402	0.852	0.84	-0.0147	0.5604	0.979	
rs2304855	A	0.49	0.8339	0.8023	0.299									

rs2304856	A	0.69	0.7908	0.922	0.391									
rs2304921	A	0.06	1.8446	2.0257	0.363									
rs2305214	A	0.40	-1.0664	0.8556	0.213									
rs2305345	A	0.27	2.6902	0.9017	0.003									
rs2305346	A	0.27	2.7181	0.9005	0.003									
rs2305912	A	0.78	0.8927	1.0391	0.390									
rs2305913	A	0.55	0.2223	0.797	0.780									
rs2305936	A	0.68	-1.7407	0.9406	0.064									
rs2306219	A	0.91	0.3409	1.5707	0.828									
rs2306690	A	0.79	1.1747	0.9798	0.231									
rs2307008	A	0.13	-2.0568	1.3678	0.133									
rs2307010	A	0.06	1.7613	2.0314	0.386									
rs2311001	C	0.52	-1.3617	0.8698	0.118									
rs2311442	A	0.82	0.6366	1.2842	0.620									
rs2311443	C	0.18	0.4536	1.3009	0.727									
rs2315921	A	0.92	0.7405	1.6218	0.648									
rs2316058	C	0.79	1.1747	0.9798	0.231									
rs2333988	A	0.18	2.3243	1.057	0.028									
rs2361701	A	0.09	-0.22	1.2996	0.866	0.08	0.9651	0.8697	0.267	0.08	0.4307	0.7521	0.567	
rs2361710	A	0.52	1.1272	0.8147	0.167									
rs2362384	A	0.96	6.0738	3.5442	0.087	0.95	-0.0232	1.189	0.984	0.95	1.0343	1.0252	0.313	0.96
rs2376999	A	0.94	0.993	1.9691	0.614									
rs2377000	A	0.49	-1.4519	0.8477	0.087									
rs2377003	A	0.04	1.5737	2.2373	0.482									
rs2377309	A	0.25	-0.7685	0.9244	0.406	0.22	0.2425	0.59	0.681	0.23	-0.0597	0.5102	0.907	
rs2377394	A	0.96	-3.6542	3.1147	0.241	0.94	0.2794	1.1163	0.802	0.94	0.1083	1.0462	0.918	
rs2377397	A	0.61	1.3553	0.7768	0.081	0.64	-0.3801	0.4629	0.412	0.63	0.0556	0.4115	0.892	
rs2377400	A	0.15	-0.4119	1.1468	0.720	0.17	-0.9124	0.6395	0.154	0.16	-0.6772	0.5928	0.253	
rs2377404	A	0.68	-1.9561	0.8038	0.015	0.71	-0.1574	0.5144	0.760	0.70	-0.4475	0.4531	0.323	
rs2384550	A	0.24	1.4882	1.0537	0.158	0.20	-0.2232	0.6122	0.715	0.21	0.2349	0.5447	0.666	
rs2385067	A	0.53	0.6769	0.8208	0.410									
rs2385264	A	0.93	-2.533	1.7971	0.159									
rs2398162	A	0.64	-0.7005	0.8396	0.404	0.58	1.4637	0.4647	0.002	0.59	0.9564	0.4145	0.021	
rs2410427	A	0.72	1.493	0.9147	0.103	0.70	-0.1362	0.5008	0.786	0.71	0.1028	0.4499	0.819	

rs2411109	A	0.56	0.1596	0.819	0.846									
rs2411122	A	0.24	-0.854	0.9744	0.381									
rs2411131	A	0.30	0.4945	0.9094	0.587	0.33	0.5444	0.5027	0.279	0.32	0.5275	0.4485	0.240	
rs2411137	A	0.53	-0.1377	0.7836	0.861	0.56	-0.1468	0.4492	0.744	0.55	-0.2455	0.4019	0.541	
rs2435974	A	0.91	-1.8935	1.5896	0.234	0.90	0.7283	0.7562	0.336	0.91	0.4976	0.697	0.475	
rs2456582	A	0.94	1.0914	1.9524	0.576	0.94	1.7005	0.9678	0.079	0.94	1.4992	0.866	0.083	0.93
rs2457692	A	0.42	0.794	0.8079	0.326									
rs2466511	A	0.94	-0.6491	1.8943	0.732	0.93	0.9711	0.9429	0.303	0.93	1.3436	0.8648	0.120	
rs2509458	A	0.94	-0.5976	2.1592	0.782	0.94	-2.5138	1.0801	0.020	0.94	-1.7368	0.9908	0.080	
rs2515815	A	0.50	0.471	0.8197	0.566									
rs2567494	A	0.43	-0.7344	0.8022	0.360	0.42	-0.234	0.461	0.612	0.43	-0.8921	0.4091	0.029	
rs2574852	A	0.17	1.6001	1.0752	0.137									
rs2584100	A	0.50	-1.249	0.8277	0.131									
rs2589118	A	0.32	1.0703	0.8535	0.210									
rs2589119	A	0.04	3.4328	2.9299	0.241									
rs2589143	A	0.23	2.3204	0.9668	0.016									
rs2589148	A	0.04	2.7141	2.9923	0.364									
rs2589150	A	0.96	3.4328	2.9299	0.241									
rs2589153	A	0.04	3.7737	3.0254	0.212									
rs2589155	A	0.96	3.4328	2.9299	0.241									
rs2589157	A	0.03	6.7628	3.3214	0.042									
rs2598414	A	0.55	0.8429	0.7992	0.292									
rs2598420	C	0.40	0.002	0.8029	0.998									
rs2598435	A	0.45	0.9365	0.7989	0.241									
rs2598444	A	0.45	0.9365	0.7989	0.241									
rs2598450	A	0.45	0.6429	0.8031	0.423									
rs2606183	A	0.73	-0.3259	0.8778	0.710	0.75	-0.1069	0.5287	0.840	0.75	-0.4033	0.4667	0.388	
rs2606188	A	0.73	-0.2619	0.8738	0.764	0.76	-0.1503	0.5341	0.778	0.75	-0.4577	0.4701	0.330	
rs2608880	A	0.46	0.1406	0.799	0.860									
rs2608882	A	0.31	-0.7687	0.9021	0.394									
rs2612753	A	0.38	-0.4943	0.7709	0.521	0.43	-0.5602	0.4601	0.223	0.41	-0.6172	0.4096	0.132	
rs2612771	A	0.58	-0.4647	0.7523	0.537	0.57	-0.2499	0.4506	0.579	0.57	-0.4495	0.4008	0.262	
rs2612773	A	0.39	-0.776	0.7892	0.326	0.35	0.6889	0.4699	0.143	0.36	0.3837	0.4201	0.361	
rs2612782	A	0.15	0.2717	1.0106	0.788	0.17	0.3835	0.5978	0.521	0.17	0.5288	0.53	0.318	

rs2612788	A	0.68	1.7801	0.7655	0.020	0.69	-0.3363	0.4655	0.470	0.69	0.0124	0.4218	0.977	
rs2613514	A	0.80	0.0447	1.2926	0.972									
rs2613516	A	0.06	-0.947	1.8664	0.612									
rs2659003	C	0.07	-0.4769	1.5618	0.760	0.07	1.7066	0.8828	0.053	0.07	1.0431	0.8101	0.198	
rs2659028	A	0.05	1.2815	3.0037	0.670	0.02	5.5688	2.5217	0.027	0.02	1.9373	1.7364	0.265	0.01
rs2659029	A	0.08	0.0994	1.4918	0.947	0.08	1.2124	0.8348	0.146	0.08	0.8061	0.7651	0.292	
rs2659030	A	0.73	-1.0541	0.8636	0.222	0.71	0.0201	0.5255	0.970	0.72	-0.1053	0.4682	0.822	0.76
rs2661686	A	0.41	0.4114	0.8511	0.629									
rs2661694	A	0.17	-0.0178	1.1644	0.988									
rs2665972	A	0.45	0.9365	0.7989	0.241									
rs2665983	C	0.42	0.6903	0.8112	0.395									
rs2665993	A	0.54	-0.6365	0.7947	0.423									
rs2665998	A	0.55	0.9133	0.8045	0.256									
rs2666011	A	0.43	0.7086	0.8075	0.380									
rs2670827	A	0.76	0.4603	0.9451	0.626									
rs2672886	A	0.81	1.0314	0.9877	0.296									
rs2672890	A	0.58	1.5353	0.813	0.059									
rs2672893	A	0.08	2.0496	1.6652	0.218									
rs2678770	A	0.45	-0.2679	0.7915	0.735									
rs2681472	A	0.87	-3.7553	1.7812	0.035	0.90	1.1739	0.8669	0.176	0.90	0.2784	0.792	0.725	
rs2681492	A	0.87	-3.7553	1.7812	0.035	0.90	1.158	0.8677	0.182	0.90	0.2615	0.7926	0.742	
rs2707031	C	0.40	-0.0868	0.7723	0.911	0.38	0.7824	0.4623	0.091	0.39	0.4925	0.4104	0.230	
rs2713991	A	0.33	0.8789	0.8502	0.301	0.31	-0.9922	0.4978	0.046	0.31	-0.5237	0.4354	0.229	
rs2714011	A	0.64	-0.421	0.8635	0.626	0.64	-0.3409	0.4755	0.474	0.64	-0.3908	0.4229	0.356	
rs2725391	A	0.61	-1.6455	0.8357	0.049	0.60	-0.4688	0.4812	0.330	0.60	-0.6263	0.4246	0.140	0.62
rs2820037	A	0.05	1.6858	2.2388	0.452	0.07	-1.0018	0.8749	0.252	0.07	-0.5798	0.8225	0.481	
rs2854701	A	0.35	-0.0232	0.846	0.978									
rs2854704	A	0.73	-0.5056	0.9952	0.612									
rs28607597	A	0.73	1.0082	0.9362	0.282	0.79	-1.0582	0.5543	0.056	0.78	-0.5519	0.4864	0.257	0.75
rs2889619	A	0.73	-0.7488	0.9019	0.406	0.74	-0.1596	0.5207	0.759	0.74	-0.4301	0.4634	0.353	
rs2889620	A	0.42	0.5993	0.7625	0.432	0.39	-1.1144	0.4709	0.018	0.40	-0.7755	0.4146	0.061	0.41
rs2889622	A	0.78	-0.0234	0.9952	0.981	0.78	0.4787	0.5569	0.390	0.78	0.4833	0.4936	0.328	
rs2889645	A	0.03	0.1719	3.287	0.958	0.04	0.5287	1.2108	0.662	0.04	0.7167	1.1187	0.522	
rs2891607	A	0.80	1.0234	1.0516	0.330									

rs2891713	C	0.07	-2.7944	1.8193	0.125									
rs2891714	A	0.91	-2.8252	1.5961	0.077									
rs2898569	A	0.32	-0.2243	0.9236	0.808									
rs2898577	A	0.28	-0.2828	0.872	0.746									
rs296139	A	0.44	-0.5155	0.837	0.538	0.42	0.1826	0.4707	0.698	0.43	0.0127	0.4224	0.976	
rs3087664	A	0.56	1.8657	0.8186	0.023									
rs3096277	A	0.25	-0.035	0.9557	0.971	0.25	-0.0913	0.5222	0.861	0.25	-0.1708	0.4666	0.714	
rs312828	A	0.47	-0.5231	0.8731	0.549									
rs312834	A	0.52	-0.201	0.8629	0.816									
rs312853	A	0.85	-0.2134	1.1808	0.857									
rs312858	A	0.33	-0.5621	0.8272	0.497									
rs312863	A	0.39	0.1001	0.8282	0.904									
rs312884	A	0.21	-1.4216	0.9681	0.142									
rs312895	A	0.92	-0.6781	1.8455	0.713									
rs312896	C	0.92	-1.0011	1.8943	0.597									
rs3178300	A	0.71	-0.3128	1.0728	0.771									
rs3184504	A	0.20	-2.4396	1.2358	0.048	0.15	-0.1614	0.6349	0.799	0.16	-0.3913	0.5837	0.503	
rs3186520	A	0.14	0.5238	1.1669	0.654									
rs3208785	C	0.63	-1.205	0.8495	0.156									
rs3208787	A	0.87	1.7004	1.3582	0.211									
rs34237952	C	0.89	1.672	1.2518	0.182	0.87	-1.5238	0.6945	0.028	0.88	-0.8967	0.636	0.159	
rs34262564	A	0.20	0.4042	0.9872	0.682	0.20	0.2226	0.5904	0.706	0.20	0.2258	0.5245	0.667	
rs3432	A	0.21	-1.1959	1.0233	0.243									
rs34520486	A	0.75	-0.6968	0.8682	0.422	0.75	-0.5122	0.5133	0.318	0.75	-0.3783	0.4572	0.408	
rs34547368	A	0.30	-0.8699	0.8133	0.285	0.32	-0.5012	0.4805	0.297	0.32	-0.34	0.4288	0.428	
rs34664664	A	0.11	-2.3742	1.3555	0.080	0.16	0.187	0.6604	0.777	0.15	-0.3329	0.5929	0.575	0.13
rs346789	A	0.77	-1.7373	1.0008	0.083									
rs346801	A	0.15	0.3643	1.2658	0.774									
rs34768269	A	0.93	-2.9955	1.6964	0.077	0.90	-0.8227	0.775	0.289	0.91	-1.1996	0.7017	0.087	0.91
rs34939985	A	0.07	-2.7033	2.1286	0.204	0.07	-0.5994	0.963	0.534	0.07	-0.9889	0.8739	0.258	
rs35035542	A									0.01	-4.1599	2.8728	0.148	
rs35110805	C	0.60	0.9431	0.8254	0.253	0.53	0.1768	0.4612	0.701	0.55	0.1176	0.4093	0.774	0.56
rs35496088	A	0.86	0.2941	1.28	0.818	0.81	0.4618	0.574	0.421	0.82	0.5023	0.5256	0.339	
rs35578653	A	0.67	0.4957	0.7745	0.522	0.69	-0.7701	0.4748	0.105	0.68	-0.4652	0.4262	0.275	

rs35863760	A	0.75	-1.2043	0.939	0.200	0.75	0.3176	0.5512	0.565	0.75	-0.2058	0.4846	0.671
rs35926122	A					0.97	-0.6582	1.2643	0.603	0.97	-0.229	1.1815	0.846
rs36013558	A					0.02	-1.01	2.6785	0.706	0.02	-0.3375	1.9653	0.864
rs363796	A	0.46	1.2786	0.802	0.111								
rs3643	A	0.90	0.8045	1.5502	0.604								
rs372558	C	0.58	-0.0423	0.8407	0.960								
rs3744026	A	0.88	0.812	1.3919	0.560								
rs3744032	A	0.05	3.7432	3.2373	0.248								
rs3744036	A	0.28	1.2669	0.9218	0.169								
rs3744037	A	0.74	-0.8746	0.93	0.347								
rs3744043	A	0.07	1.9355	1.6479	0.240								
rs3744054	A	0.17	0.7177	1.0445	0.492								
rs3744166	A	0.12	1.2432	1.3242	0.348								
rs3744171	A	0.07	-1.8867	2.2369	0.399								
rs3744173	A	0.42	-0.6218	0.8017	0.438								
rs3744181	A	0.40	-1.2646	0.7511	0.092	0.41	0.7456	0.4543	0.101	0.41	0.2596	0.4047	0.521
rs3744182	A	0.05	-4.5476	2.4902	0.068	0.03	-0.1705	1.46	0.907	0.04	-1.2354	1.2786	0.334
rs3744186	A	0.43	-1.6581	0.7259	0.022	0.45	0.9299	0.4493	0.038	0.45	0.3238	0.3974	0.415
rs3744189	A	0.13	-0.4267	1.5842	0.788								
rs3744198	A	0.24	-0.2332	1.0004	0.816								
rs3744203	A	0.50	0.9538	0.8082	0.238								
rs3744204	A	0.46	0.6691	0.8196	0.414								
rs3744215	A	0.19	-0.7311	1.097	0.505								
rs3744216	A	0.95	1.6061	2.4215	0.507								
rs3744793	A	0.43	0.2746	0.8351	0.742								
rs3751932	A	0.88	-0.4432	1.8501	0.811								
rs3751934	A	0.55	-0.1177	0.7771	0.880								
rs3751947	A	0.08	-1.1585	1.754	0.509	0.06	-2.6892	1.1334	0.018	0.06	-2.0613	1.0097	0.041
rs3751955	A	0.50	-0.5834	0.8044	0.468								
rs3751956	A	0.14	1.5669	1.2282	0.202								
rs3751957	A	0.39	-1.2067	0.8529	0.157								
rs3751962	A	0.83	0.3668	1.3609	0.788								
rs3754777	A	0.34	0.0926	0.8158	0.910	0.32	1.171	0.4944	0.018	0.33	0.863	0.4353	0.047
rs3764377	A	0.82	0.5129	1.3383	0.702								

rs3764438	A	0.08	1.1168	1.7609	0.526	0.05	-0.5734	1.1429	0.616	0.05	-0.2027	1.024	0.843
rs3764440	A	0.89	0.8427	1.3833	0.542	0.90	-0.5318	0.8243	0.519	0.90	-0.1832	0.7482	0.807
rs3765123	A	0.09	-2.4426	1.5159	0.107								
rs3785446	A	0.07	1.7908	2.24	0.424								
rs3785529	A	0.41	-1.6004	0.8395	0.057								
rs379465	A	0.14	0.6644	1.1996	0.580								
rs3803737	A	0.33	0.9106	0.8427	0.280								
rs3803739	A	0.44	-1.3034	0.8504	0.125								
rs3803742	A	0.23	-0.175	1.2682	0.890								
rs3803767	A	0.05	-3.9623	2.7843	0.155								
rs3803780	A					0.98	1.8983	2.5403	0.455	0.98	0.4343	2.3873	0.856
rs3803783	A	0.81	-0.7662	1.0804	0.478								
rs3803786	C	0.90	-3.0765	2.0179	0.127								
rs3803792	A	0.36	1.4999	0.8223	0.068								
rs3809691	C	0.41	-0.2041	0.8425	0.809								
rs3809713	A	0.73	-2.759	0.8128	0.001	0.71	0.2505	0.4972	0.614	0.72	-0.4875	0.4409	0.269
rs3813063	A	0.30	0.4731	0.9319	0.612								
rs3816427	A	0.72	0.965	0.9302	0.300								
rs3817292	C	0.22	2.0418	0.9967	0.041								
rs381815	A	0.36	0.3693	0.8786	0.674	0.32	0.4458	0.498	0.371	0.33	0.4197	0.4418	0.342
rs3826274	A	0.29	0.0639	0.9242	0.945								
rs3826314	A	0.19	-0.8052	1.089	0.460								
rs3826552	A	0.43	0.6744	0.8164	0.409								
rs3829574	A	0.15	0.4896	1.2144	0.687								
rs3829611	A	0.31	0.5674	0.9074	0.532								
rs3829612	A	0.27	0.5254	0.9265	0.571								
rs3851021	A	0.16	1.4514	1.1954	0.225								
rs385689	A	0.23	2.2117	1.1191	0.048								
rs3869467	A	0.46	0.0127	0.8366	0.988								
rs387601	A	0.49	-0.449	0.8164	0.582								
rs387774	A	0.86	0.5223	1.1752	0.657								
rs388090	A	0.78	-0.0584	1.052	0.956								
rs3889146	A	0.71	0.4534	0.8991	0.614								
rs3893391	C	0.88	0.6375	1.3686	0.641								

rs3924327	A	0.42	1.6593	2.2931	0.469									
rs3934492	C	0.76	-1.4794	0.9472	0.118									
rs3934967	A	0.70	1.3027	0.8013	0.104	0.70	0.7186	0.4874	0.140	0.70	0.9936	0.4284	0.020	
rs3935192	A	0.14	-2.8373	1.2111	0.019									
rs3935543	A	0.23	-0.0009	1.0491	0.999									
rs3935648	C	0.79	0.686	1.0928	0.530									
rs3935674	C	0.61	-0.5784	0.8464	0.494									
rs3936118	A	0.50	-1.2491	0.8455	0.140									
rs3936523	A	0.49	-1.4388	0.8452	0.089									
rs397481	A	0.06	0.3283	1.9872	0.869									
rs402273	A	0.29	1.4472	0.9758	0.138									
rs403483	A	0.14	0.7005	1.1887	0.556									
rs4073111	C	0.45	-0.0889	0.7991	0.911									
rs4073996	A	0.05	-0.0792	2.7633	0.977	0.04	-0.7176	1.1977	0.549	0.04	-0.6526	1.0751	0.544	
rs4073997	C	0.62	2.8455	0.9078	0.002									
rs4074022	C	0.41	1.1157	0.7539	0.139	0.38	0.0896	0.4603	0.846	0.39	0.6128	0.4069	0.132	
rs4074023	A	0.65	1.2296	0.7879	0.119	0.67	-0.2511	0.468	0.592	0.67	0.3781	0.4193	0.367	
rs4074469	A	0.24	1.5794	0.9429	0.094	0.22	0.092	0.5653	0.871	0.22	0.4752	0.497	0.339	
rs4075482	A	0.24	0.2867	0.917	0.755									
rs4076427	C	0.26	0.3396	0.8861	0.702									
rs4076967	C	0.76	0.3148	0.9798	0.748									
rs4077126	A	0.44	0.5054	0.8119	0.534									
rs4077240	A	0.29	1.2928	0.893	0.148									
rs4077719	A	0.92	0.1267	2.0491	0.951									
rs4078259	A	0.38	-0.7614	0.8493	0.370									
rs4078429	A	0.94	-5.4598	3.1076	0.079									
rs4078474	C	0.63	-0.6451	0.8751	0.461									
rs4082919	A	0.65	1.2094	0.8695	0.164									
rs4103047	C	0.49	-1.4388	0.8452	0.089									
rs4129767	A	0.62	1.3867	0.8146	0.089									
rs4239025	A	0.57	0.3551	0.8131	0.662	0.62	-1.1615	0.4773	0.015	0.61	-0.8212	0.4189	0.050	0.59
rs4239026	C	0.41	0.6831	0.7662	0.373	0.38	-1.0992	0.4728	0.020	0.40	-0.7327	0.4168	0.079	0.41
rs4243249	A	0.23	0.1974	0.8863	0.824	0.19	0.388	0.5749	0.500	0.20	0.5923	0.5021	0.238	
rs4243251	A	0.74	0.2206	1.0055	0.826									

rs4255830	A	0.17	0.6042	1.0523	0.566								
rs4295	C	0.25	-0.1713	1.0543	0.871	0.19	-0.5812	0.589	0.324	0.20	-0.5123	0.519	0.324
rs4305	A	0.26	-0.2563	1.0372	0.805	0.22	-0.716	0.556	0.198	0.23	-0.6221	0.4928	0.207
rs4309	A	0.75	-0.3023	0.9585	0.753	0.75	-0.6602	0.5338	0.216	0.75	-0.477	0.4761	0.317
rs4318268	A	0.28	1.2058	0.9076	0.184								
rs4331	A	0.32	-0.4461	0.855	0.602	0.28	-0.1794	0.4963	0.718	0.29	-0.2291	0.4372	0.600
rs4333	A	0.32	-0.4724	0.8525	0.580	0.28	-0.1389	0.5013	0.782	0.28	-0.2123	0.4393	0.629
rs4335	A	0.68	-0.4562	0.8536	0.593	0.72	-0.1979	0.497	0.691	0.72	-0.2538	0.4377	0.562
rs4341	C	0.32	-0.4088	0.8516	0.631	0.28	-0.1707	0.4978	0.732	0.29	-0.2129	0.4369	0.626
rs4344	A	0.68	-0.4088	0.8516	0.631	0.71	-0.1901	0.4941	0.701	0.71	-0.2274	0.4351	0.601
rs4350602	A	0.88	-1.4616	1.2899	0.257								
rs4351	A	0.68	-0.4357	0.8576	0.611	0.72	-0.1084	0.5017	0.829	0.72	-0.1648	0.4409	0.709
rs4352096	A	0.55	-0.7896	0.8274	0.340								
rs4353	A	0.32	-0.4104	0.8493	0.629	0.28	-0.174	0.4936	0.724	0.29	-0.2154	0.4347	0.620
rs4362	A	0.31	-0.5862	0.854	0.493	0.28	-0.2177	0.497	0.662	0.28	-0.2818	0.4371	0.519
rs4363	A	0.69	-0.4705	0.8497	0.580	0.72	-0.1962	0.4946	0.692	0.72	-0.2569	0.4348	0.555
rs4370013	A	0.54	0.6164	0.833	0.459	0.59	-0.2878	0.4706	0.541	0.58	-0.1633	0.4196	0.697
rs4375697	A	0.60	1.0087	0.8301	0.224								
rs4411562	A	0.20	-0.0064	1.3014	0.996								
rs4424945	A	0.19	-0.349	1.0358	0.736								
rs443970	A	0.48	0.6845	0.8141	0.400								
rs4441315	A	0.29	1.989	0.8191	0.015	0.29	-0.7922	0.503	0.115	0.28	-0.052	0.4491	0.908
rs4444373	C	0.96	-3.6542	3.1147	0.241	0.93	0.4327	1.0578	0.683	0.93	0.2913	0.998	0.770
rs445507	A	0.86	1.3045	1.2288	0.288								
rs445683	A	0.55	-0.6002	0.8367	0.473								
rs4459614	A	0.22	-1.091	0.9335	0.243								
rs4462668	A	0.50	1.0755	0.7795	0.168	0.50	0.2695	0.4382	0.539	0.50	0.2588	0.3898	0.507
rs4465632	A	0.71	-0.138	0.9113	0.880								
rs448203	A	0.17	1.2151	1.169	0.299								
rs4482327	A	0.79	0.3254	0.9379	0.729	0.77	0.6394	0.5404	0.237	0.78	0.5861	0.4815	0.224
rs448378	A	0.81	1.0124	1.1183	0.365	0.80	-0.4855	0.5614	0.387	0.81	0.0446	0.5097	0.930
rs4485403	A	0.90	1.1548	1.3916	0.407	0.91	-1.2059	0.8309	0.147	0.91	-0.7589	0.759	0.317
rs4490057	A	0.39	1.0738	0.8502	0.207								
rs4491585	A	0.17	-0.5666	1.1796	0.631								

rs4491586	A	0.62	0.3071	0.828	0.711								
rs4505373	A	0.47	-0.3568	0.8083	0.659								
rs4516263	A	0.58	0.0444	0.8395	0.958								
rs4523953	A	0.57	0.5498	0.8935	0.538								
rs4531782	A	0.67	0.9647	0.8336	0.247								
rs4533317	A	0.88	-0.0301	1.3656	0.982	0.87	0.2548	0.7108	0.720	0.87	0.0263	0.6346	0.967
rs4538044	A	0.15	1.9825	1.2088	0.101								
rs454138	C	0.29	0.7406	0.937	0.429								
rs4542691	A	0.09	-2.3594	1.6065	0.142								
rs454845	A	0.14	0.5223	1.1752	0.657								
rs4558471	A	0.85	1.2868	1.1787	0.275								
rs4564643	A	0.17	0.4696	1.3624	0.730								
rs4592695	A	0.21	0.2177	0.9872	0.826	0.23	0.5385	0.5461	0.324	0.22	0.5748	0.4841	0.235
rs4622540	A	0.48	0.1267	0.7437	0.865	0.50	0.4301	0.4463	0.335	0.50	0.4205	0.3919	0.283
rs4624215	A	0.91	-2.8252	1.5961	0.077								
rs4788837	A	0.77	0.5258	0.9744	0.590								
rs4788840	A	0.85	0.1572	1.3432	0.907								
rs4788887	C	0.09	-2.456	1.5855	0.121								
rs4788889	A	0.07	-2.7944	1.8193	0.125								
rs4788890	A	0.07	-2.7944	1.8193	0.125								
rs4788891	A	0.07	-2.7944	1.8193	0.125								
rs4788893	A	0.33	0.8708	0.8729	0.319								
rs4788931	A	0.84	0.5252	1.2526	0.675								
rs4789012	A	0.39	-0.9054	0.894	0.311								
rs4789084	A	0.65	-0.17	0.8577	0.843								
rs4789096	C	0.45	-1.135	0.8478	0.181								
rs4789102	A	0.21	1.2128	0.9567	0.205								
rs4789114	A	0.30	-0.949	0.8282	0.252								
rs4789145	A	0.50	0.7844	0.8488	0.355								
rs4789170	A	0.09	-2.8252	1.5961	0.077								
rs4789172	A	0.35	1.0798	0.8493	0.204								
rs4789173	A	0.07	-3.0128	1.8154	0.097								
rs4789176	A	0.07	-3.0128	1.8154	0.097								
rs4789178	C	0.93	-2.533	1.7971	0.159								

rs4789181	A	0.07	-2.533	1.7971	0.159									
rs4789182	A	0.88	-1.2347	1.2843	0.336									
rs4789183	A	0.93	-2.533	1.7971	0.159									
rs4789186	A	0.93	-2.7944	1.8193	0.125									
rs4789188	A	0.07	-2.7944	1.8193	0.125									
rs4789213	C	0.35	-1.3104	0.9101	0.150									
rs4789251	A	0.53	-0.2072	0.8043	0.797									
rs4789274	A	0.33	0.6667	0.855	0.436									
rs4789291	A	0.43	-0.9358	0.8532	0.273									
rs4789302	A	0.70	1.1435	0.9257	0.217									
rs4789366	A	0.71	-0.059	0.8529	0.945									
rs4789378	A	0.32	0.0994	0.8806	0.910									
rs4789380	A	0.14	0.4117	1.1738	0.726									
rs4789418	C	0.17	-1.0033	1.1632	0.388									
rs4789462	A	0.42	1.021	0.8479	0.229	0.41	0.0344	0.4761	0.942	0.41	-0.0771	0.42	0.854	
rs4789531	A	0.68	-0.144	0.9083	0.874									
rs4789533	C	0.81	-0.1117	1.0717	0.917									
rs4789559	A	0.45	-0.9842	0.814	0.227									
rs4789649	A	0.26	-0.4025	0.9329	0.666									
rs4789671	A	0.77	-1.1673	0.9271	0.208									
rs4789763	A	0.62	-0.5241	0.8399	0.533									
rs4789853	C	0.52	0.6474	0.7518	0.389	0.56	0.3789	0.471	0.421	0.57	0.5625	0.4146	0.175	
rs4789860	A	0.57	-0.5244	0.8504	0.538									
rs4789874	A	0.37	-0.4068	0.8773	0.643	0.31	0.1613	0.4958	0.745	0.32	0.0291	0.438	0.947	
rs4789875	A	0.74	0.5866	0.9583	0.541	0.73	-0.3464	0.5173	0.503	0.73	-0.1575	0.4635	0.734	0.70
rs4789878	C	0.82	-0.342	1.0321	0.740	0.82	-0.6142	0.6012	0.307	0.81	-0.664	0.5352	0.215	0.82
rs4789879	A	0.18	-3.4445	1.0168	0.001	0.20	-0.0193	0.5868	0.974	0.20	-0.8068	0.5255	0.125	
rs4789883	A	0.96	3.7882	3.0322	0.212	0.95	0.0351	1.1199	0.975	0.95	0.4694	1.0108	0.642	
rs4789885	A	0.46	0.0085	0.7648	0.991	0.45	0.1466	0.4457	0.742	0.46	0.1568	0.401	0.696	
rs4789887	A	0.26	-0.6062	0.8578	0.480	0.28	-0.0859	0.5087	0.866	0.27	-0.2768	0.4531	0.541	
rs4789888	A	0.25	0.0355	0.8562	0.967	0.26	0.7906	0.5013	0.115	0.26	0.5619	0.4461	0.208	
rs4789890	A	0.86	0.4254	1.0411	0.683	0.84	0.1033	0.6577	0.875	0.84	0.0369	0.5759	0.949	0.84
rs4789892	A	0.19	0.5835	1.0918	0.593	0.13	-0.3427	0.7014	0.625	0.15	-0.3535	0.617	0.567	
rs4789893	C	0.43	1.2805	0.8081	0.113	0.42	0.0313	0.4664	0.947	0.42	0.2426	0.4197	0.563	

rs4789904	A	0.41	-0.7024	0.7951	0.377									
rs4789911	A	0.52	-1.7101	0.7465	0.022	0.47	0.321	0.4652	0.490	0.47	-0.1467	0.4098	0.720	
rs4789939	A	0.07	1.4186	1.7958	0.430									
rs4789940	A	0.83	-1.3069	1.109	0.239									
rs4789949	A	0.55	0.1572	0.739	0.832	0.59	0.7528	0.467	0.107	0.58	0.492	0.4134	0.234	
rs4789951	A	0.35	-0.141	0.7581	0.853	0.39	0.3137	0.4592	0.495	0.38	0.0317	0.4101	0.938	
rs4789962	A	0.74	0.6774	0.9616	0.481	0.73	-0.2282	0.5164	0.659	0.74	-0.0097	0.4628	0.983	
rs4789963	A	0.65	-0.7112	0.8755	0.417	0.69	-0.0799	0.4861	0.870	0.68	-0.2265	0.4306	0.599	
rs4789964	C	0.35	-0.4002	0.8152	0.623	0.32	0.0451	0.4824	0.926	0.33	-0.0441	0.4275	0.918	
rs4789965	A	0.25	0.7056	0.9671	0.466	0.26	-0.4094	0.5158	0.427	0.26	-0.1742	0.4627	0.707	0.29
rs4789966	A	0.37	-0.4068	0.8773	0.643	0.30	0.1657	0.4944	0.738	0.32	0.0325	0.4372	0.941	
rs4789967	C	0.10	3.36	1.3951	0.016	0.08	0.6831	0.9181	0.457	0.08	1.3206	0.8034	0.100	
rs4789968	A	0.06	6.0746	3.0332	0.045	0.06	-0.0771	1.0553	0.942	0.05	-0.0856	0.968	0.930	
rs4789976	A	0.17	0.9837	1.1733	0.402	0.19	0.0459	0.5939	0.938	0.18	0.213	0.5327	0.689	
rs4789977	A	0.64	0.6064	0.8016	0.449	0.68	0.1288	0.4942	0.794	0.67	0.1494	0.4278	0.727	0.65
rs4789978	A	0.25	0.8503	1.0463	0.416	0.22	0.0543	0.5725	0.924	0.23	0.3568	0.5025	0.478	
rs4789979	A	0.50	-0.1456	0.7432	0.845	0.53	-0.5856	0.4626	0.206	0.51	-0.6234	0.4049	0.124	0.52
rs4789980	C	0.57	-0.944	0.8034	0.240	0.56	0.1072	0.4525	0.813	0.56	-0.1913	0.3998	0.632	
rs4789981	A	0.26	-0.4727	0.906	0.602									
rs4789986	A	0.56	0.472	0.8283	0.569									
rs4789994	A	0.50	0.5809	0.7614	0.446	0.54	-0.1506	0.4588	0.743	0.53	-0.1162	0.4051	0.774	
rs4789997	A	0.55	-0.3739	0.8018	0.641	0.61	-0.6635	0.4672	0.156	0.60	-0.6688	0.4094	0.102	
rs4789998	A	0.56	0.087	0.7645	0.909	0.61	-0.5559	0.4639	0.231	0.60	-0.5502	0.408	0.178	
rs4789999	A	0.28	-1.4431	0.8591	0.093	0.29	-0.0364	0.4994	0.942	0.28	-0.2012	0.452	0.656	
rs4790000	A	0.16	-0.1689	1.0826	0.876	0.18	-0.2484	0.6117	0.685	0.17	-0.1007	0.5649	0.859	
rs4790001	A	0.46	0.0185	0.7723	0.981	0.48	0.0266	0.4514	0.953	0.46	0.0267	0.4085	0.948	
rs4790002	A	0.38	-0.2985	0.788	0.705	0.42	0.0338	0.4533	0.941	0.41	-0.0689	0.4098	0.866	
rs4790004	A					0.97	0.9813	1.529	0.521	0.97	1.5724	1.4042	0.263	0.96
rs4790005	A	0.39	-0.722	0.8407	0.390	0.42	0.2169	0.4665	0.642	0.41	-0.0046	0.422	0.991	
rs4790007	A	0.50	-0.7816	0.7358	0.288	0.48	0.5456	0.4503	0.226	0.49	0.224	0.3968	0.572	
rs4790009	A	0.36	0.3739	0.7969	0.639	0.38	0.5594	0.4802	0.244	0.38	0.3159	0.423	0.455	
rs4790013	A	0.50	0.0261	0.752	0.972	0.49	0.3805	0.4561	0.404	0.50	0.2876	0.404	0.476	
rs4790015	A	0.26	0.3112	0.9029	0.730									
rs4790018	A	0.69	-1.2668	0.8534	0.138	0.75	-0.2973	0.5345	0.578	0.74	-0.655	0.4629	0.157	0.76

rs4790019	A	0.24	-1.0112	0.9304	0.277								
rs4790024	A	0.65	0.756	0.8846	0.393	0.65	-0.7368	0.4847	0.129	0.64	-0.5452	0.434	0.209
rs4790032	A	0.63	0.3157	0.826	0.702	0.64	0.1972	0.49	0.687	0.64	0.2598	0.4365	0.552
rs4790036	C									0.02	-2.8131	3.0398	0.355
rs4790048	A	0.17	-2.2301	1.1555	0.054	0.23	-0.2284	0.5596	0.683	0.22	-0.4168	0.5032	0.408
rs4793297	A	0.10	1.0761	1.7283	0.534	0.08	0.8408	0.8592	0.328	0.09	1.1352	0.7703	0.141
rs4793316	A	0.45	-0.1133	0.7983	0.887	0.47	-1.0258	0.4508	0.023	0.47	-0.7435	0.3982	0.062
rs4796817	A	0.14	-0.4066	1.4108	0.773								
rs4889782	A	0.81	0.6233	1.0065	0.536								
rs4889784	A	0.24	0.5775	0.9051	0.524								
rs4889787	A	0.60	-0.7909	0.829	0.340								
rs4889809	A	0.27	-0.2945	0.8983	0.743	0.22	-0.0262	0.5567	0.963	0.23	-0.0514	0.4857	0.916
rs4889810	A	0.19	1.8137	0.9689	0.061	0.20	-0.0895	0.5932	0.880	0.20	0.4837	0.5238	0.356
rs4889811	A	0.22	0.4071	0.9561	0.670	0.18	0.5535	0.5846	0.344	0.19	0.6571	0.5078	0.196
rs4889815	A	0.11	0.0903	1.2573	0.943	0.08	1.0075	0.8399	0.230	0.09	0.7814	0.7186	0.277
rs4889839	A	0.40	1.0648	0.911	0.243								
rs4889852	A	0.11	1.7472	1.3628	0.200								
rs4889872	A	0.78	1.1702	0.9683	0.227								
rs4889875	A	0.79	1.1747	0.9798	0.231								
rs4889882	A	0.79	0.9872	0.9702	0.309								
rs4889938	A	0.33	-1.036	0.8974	0.248	0.26	-0.0155	0.5206	0.976	0.28	-0.1521	0.4561	0.739
rs4889940	A	0.37	-0.1721	0.7593	0.821	0.38	0.2245	0.4852	0.644	0.38	0.0766	0.427	0.858
rs4889944	A	0.79	0.312	0.9663	0.747	0.82	0.3881	0.5925	0.512	0.81	0.5106	0.5144	0.321
rs4889951	A	0.28	-0.3662	1.2204	0.764								
rs4889954	A	0.54	0.1375	0.7564	0.856	0.47	-0.2543	0.4315	0.556	0.49	-0.0913	0.3905	0.815
rs4889961	C	0.45	-0.7404	0.7553	0.327	0.51	0.4443	0.4333	0.305	0.49	0.313	0.391	0.423
rs4889968	A	0.85	-1.2742	1.4097	0.366								
rs4889990	A	0.30	0.7119	0.9811	0.468								
rs4889995	A	0.30	0.4731	0.9319	0.612								
rs4889996	A	0.70	0.4731	0.9319	0.612								
rs4889998	A	0.57	-0.2884	0.8299	0.728								
rs4890010	A	0.25	1.1273	0.8806	0.201								
rs4890012	C	0.75	1.1943	0.8805	0.175								
rs4890026	A	0.14	0.9262	1.2503	0.459								

rs493430	A	0.39	0.6035	0.839	0.472
rs4969147	A	0.36	0.2655	0.8125	0.744
rs4969168	A	0.27	1.2052	1.0855	0.267
rs4969178	A	0.35	1.5964	0.8333	0.055
rs4969186	C	0.35	1.5354	0.8341	0.066
rs4969187	A	0.35	1.4712	0.8382	0.079
rs4969189	A	0.83	0.2753	1.1464	0.810
rs4969219	A	0.74	2.2549	0.9405	0.017
rs4969227	A	0.26	-2.3505	0.9117	0.010
rs4969230	A	0.26	0.6014	0.9051	0.506
rs4969235	A	0.26	0.5742	0.9068	0.527
rs4969245	A	0.77	0.4747	0.9808	0.628
rs4969282	A	0.06	3.0377	2.4318	0.212
rs4969301	A	0.38	2.468	0.8614	0.004
rs4969303	A	0.15	1.3926	1.2001	0.246
rs4969310	A	0.15	1.3121	1.2002	0.274
rs4969311	C	0.65	2.4673	0.8174	0.003
rs4969322	C	0.40	-0.5322	0.8376	0.525
rs4969331	A	0.62	-0.4943	0.8488	0.560
rs4969355	A	0.18	-0.8098	1.0916	0.458
rs4969358	A	0.43	-0.7611	0.796	0.339
rs4969364	A	0.44	0.6009	0.8052	0.456
rs4969381	A	0.77	0.5493	0.9754	0.573
rs4969382	A	0.50	-0.931	0.8496	0.273
rs4969384	A	0.18	0.0074	1.0807	0.995
rs4969385	A	0.47	-0.8254	0.8103	0.308
rs4969387	C	0.83	0.6286	1.1278	0.577
rs4969429	A	0.20	0.5209	1.0027	0.603
rs4969441	A	0.79	0.1517	1.1384	0.894
rs509574	A	0.31	-0.3762	0.8894	0.672
rs509911	A	0.82	-0.3178	1.0923	0.771
rs521750	A	0.57	0.7343	0.8533	0.390
rs524536	A	0.90	-0.5917	1.6458	0.719
rs545652	A	0.10	0.4324	2.1656	0.842

rs546371	A	0.68	-0.4326	0.8231	0.599								
rs553781	A	0.39	0.2831	0.8405	0.736								
rs554012	A	0.64	0.1081	0.8024	0.893								
rs55714027	A	0.44	-1.3834	0.775	0.074	0.51	1.1461	0.4538	0.012	0.50	0.599	0.4053	0.139
rs56107536	A	0.39	-1.2642	0.8037	0.116	0.42	0.0622	0.4766	0.896	0.42	-0.2226	0.423	0.599
rs56259513	A	0.24	1.2581	0.9216	0.172	0.27	-0.084	0.5124	0.870	0.26	0.2072	0.459	0.652
rs56353542	A					0.96	-1.8288	1.3276	0.168	0.96	-0.4285	1.3169	0.745
rs56407805	A	0.89	0.1953	1.1196	0.862	0.89	0.8072	0.7406	0.276	0.89	0.4469	0.6428	0.487
rs567009	A	0.59	-0.2547	0.8447	0.763								
rs579238	A	0.31	-0.3131	0.9002	0.728								
rs581157	A	0.65	0.081	0.8581	0.925								
rs59652033	A	0.65	1.2557	0.7949	0.114	0.68	-1.1836	0.4947	0.017	0.68	-0.4977	0.439	0.257
rs60582626	A	0.43	-0.5355	0.7252	0.460	0.48	-0.3291	0.4596	0.474	0.46	-0.3077	0.4045	0.447
rs60684213	A	0.04	-0.8548	2.822	0.762	0.03	-0.0966	1.5583	0.951	0.03	-1.2465	1.2317	0.312
rs613075	A	0.37	0.0668	0.8063	0.934								
rs61729127	A									0.99	0.1779	3.325	0.957
rs61740509	A	0.05	-0.9493	2.7349	0.729	0.05	0.1679	1.0138	0.869	0.05	-0.0695	0.9517	0.942
rs61756761	A	0.97	-2.7935	3.2908	0.396	0.97	0.6335	1.6524	0.701	0.97	-0.1335	1.5059	0.929
rs62063818	A					0.04	-1.8109	1.3266	0.172	0.04	-0.4172	1.317	0.751
rs62063824	A					0.04	-1.2142	1.3839	0.380	0.03	-0.3102	1.3895	0.823
rs62063831	A	0.75	-2.5029	0.8492	0.003	0.74	-0.0809	0.5183	0.876	0.74	-0.7376	0.4583	0.108
rs62074551	A	0.56	0.4633	0.7309	0.526	0.56	-0.4162	0.4528	0.358	0.55	-0.0947	0.3988	0.812
rs631730	C	0.25	0.6538	0.9392	0.486								
rs633672	A	0.41	1.2766	0.7961	0.109								
rs6416847	A	0.37	-0.8393	0.8568	0.327								
rs6420480	A	0.86	1.3498	1.2311	0.273								
rs6420481	A	0.54	-0.7438	0.8135	0.361								
rs6420487	A	0.42	-0.5687	0.8557	0.506								
rs647727	A	0.90	1.4844	1.4299	0.299								
rs6495122	A	0.67	-0.4233	0.8474	0.617	0.72	-0.3382	0.5081	0.506	0.72	-0.2501	0.4541	0.582
rs6501243	A	0.49	-1.5932	0.8475	0.060								
rs6501265	A	0.51	-0.6769	0.8347	0.417								
rs6501267	A	0.57	0.4565	0.8177	0.577								
rs6501285	A	0.26	-0.3331	0.8287	0.688	0.28	-0.0526	0.51	0.918	0.27	-0.0217	0.4569	0.962

rs6501297	A	0.50	0.6975	0.7926	0.379									0.46
rs6501298	A					0.02	0.9002	2.3752	0.705	0.02	1.9371	1.8032	0.283	
rs6501571	A	0.56	-0.4482	0.7893	0.570	0.56	-0.979	0.4457	0.028	0.56	-0.7714	0.3941	0.050	
rs6501584	A	0.60	0.6058	0.826	0.463	0.55	-0.1179	0.4615	0.798	0.56	-0.2039	0.412	0.621	
rs6501587	A	0.48	-0.0188	0.7872	0.981	0.52	-0.2046	0.4594	0.656	0.51	-0.1439	0.4045	0.722	
rs6501760	A	0.35	-1.4341	0.8852	0.105									
rs6501805	A	0.39	-0.1511	0.8429	0.858									
rs6501812	A	0.40	0.0677	0.8618	0.937									
rs6501826	A	0.48	0.1423	0.7866	0.857									
rs6501830	C	0.32	0.2296	0.938	0.807									
rs6501868	A	0.55	0.1846	0.8107	0.820									
rs6501878	A	0.68	0.9282	0.8462	0.273									
rs6501882	A	0.33	0.8263	0.8474	0.330									
rs6501927	A	0.76	0.0447	0.8778	0.959									
rs6501993	A	0.59	-0.3884	0.8007	0.628	0.60	-0.2033	0.4642	0.661	0.60	-0.413	0.4105	0.314	
rs6502097	C	0.78	0.7911	0.9743	0.417									
rs653178	A	0.80	-2.4396	1.2358	0.048	0.85	-0.1614	0.6349	0.799	0.84	-0.3913	0.5837	0.503	
rs6565469	A	0.78	1.2663	0.9713	0.192									
rs6565472	A	0.08	-1.4742	2.0216	0.466									
rs6565476	A	0.07	0.5663	1.6767	0.736									
rs6565478	A	0.79	1.1784	0.9815	0.230									
rs6565480	A	0.86	0.3136	1.1226	0.780									
rs6565484	A	0.74	2.3262	0.9394	0.013									
rs6565495	A	0.85	1.426	1.2219	0.243									
rs6565499	A	0.15	1.3121	1.2002	0.274									
rs6565507	A	0.17	-0.8115	1.5801	0.608									
rs6565531	A	0.48	-0.0924	0.8042	0.909									
rs6565535	A	0.55	-0.6795	0.7918	0.391									
rs6565548	A	0.73	-0.3644	0.9288	0.695									
rs6565549	A	0.63	0.0908	0.8216	0.912									
rs6565550	A	0.83	-0.4056	1.0744	0.706									
rs6565570	A	0.17	-0.3688	1.2608	0.770									
rs6565571	A	0.17	-0.774	1.2052	0.521									
rs6565593	A	0.69	0.1745	0.8431	0.836									

rs6565604	A	0.76	0.0652	1.0444	0.950								
rs6565605	A	0.76	0.0652	1.0444	0.950								
rs6565612	A	0.26	-0.3306	0.9431	0.726								
rs6565616	A	0.74	-0.364	0.9425	0.699								
rs6565633	A	0.27	1.5382	0.8605	0.074	0.32	0.0301	0.5219	0.954	0.31	0.4234	0.4654	0.363
rs6565635	A	0.29	1.5909	0.8038	0.048	0.33	0.2056	0.4928	0.677	0.31	0.5407	0.4388	0.218
rs6565642	A	0.88	-0.508	1.3893	0.715								
rs6565643	A	0.51	0.6005	0.8189	0.463								
rs6565650	A	0.45	-0.1155	0.8356	0.890								
rs6565651	A	0.45	-0.0995	0.8267	0.904								
rs6565666	A	0.05	0.4267	1.8436	0.817								
rs6565677	A	0.71	0.8819	0.8481	0.299								
rs6565680	A	0.28	1.1695	0.8797	0.184								
rs6565681	A	0.64	0.572	0.812	0.481								
rs6565683	A	0.64	0.6433	0.8117	0.428								
rs6565686	A	0.68	0.8642	0.8341	0.300								
rs6565689	A	0.48	0.2773	0.7931	0.727								
rs6565697	A	0.15	0.4298	1.1087	0.698	0.17	-0.597	0.6054	0.324	0.17	-0.1914	0.5419	0.724
rs6711736	A	0.22	1.1745	0.9325	0.208	0.28	-0.2715	0.5026	0.589	0.26	0.1177	0.4495	0.794
rs67296984	A	0.13	1.6533	1.288	0.199	0.10	-1.3118	0.8562	0.126	0.09	-0.4535	0.7558	0.548
rs6729869	A	0.22	1.1047	0.9334	0.237	0.28	-0.2734	0.5016	0.586	0.27	0.1009	0.4487	0.822
rs674402	A	0.23	1.3102	1.1217	0.243								
rs6749447	A	0.43	0.0801	0.8033	0.921	0.43	-1.0755	0.4515	0.017	0.43	-0.7998	0.4041	0.048
rs685099	A	0.87	1.3378	1.2907	0.300								
rs685441	A	0.71	-0.3054	0.893	0.732								
rs689730	A	0.26	0.9302	0.8999	0.301								
rs689895	C	0.80	-0.1687	1.0686	0.875								
rs690124	A	0.16	-0.4144	1.1994	0.730								
rs690371	A	0.81	-0.7714	1.0867	0.478								
rs690418	A	0.18	-0.1326	1.0859	0.903								
rs690514	A	0.18	-0.3178	1.0923	0.771								
rs690533	A	0.82	-0.7827	1.0928	0.474								
rs691797	C	0.12	0.7192	1.2478	0.564								
rs707707	A	0.38	0.0954	0.8733	0.913								

rs715041	A	0.06	0.2372	1.8112	0.896	0.05	1.5603	1.036	0.132	0.05	1.2089	0.9004	0.179	
rs719430	A	0.79	0.5113	1.0311	0.620									
rs719781	A	0.10	-0.8457	1.4013	0.546									
rs7198	C	0.13	-1.4818	1.239	0.232									
rs7206926	A	0.40	-0.365	0.8181	0.656									
rs7207088	A	0.21	0.2767	0.938	0.768	0.23	0.7193	0.541	0.184	0.22	0.6297	0.482	0.191	
rs7207208	A	0.27	1.201	0.909	0.186									
rs7207596	A	0.09	-0.4087	1.6727	0.807									
rs7207683	A	0.70	0.9903	0.9338	0.289									
rs7207955	A	0.13	0.5354	1.2665	0.673									
rs7208049	A	0.92	1.244	1.4588	0.394	0.91	-1.003	0.8305	0.227	0.91	-0.6866	0.7559	0.364	
rs7208114	C	0.76	0.7225	0.9317	0.438									
rs7208218	C	0.57	-0.2884	0.8299	0.728									
rs7208264	C	0.26	0.9171	0.8705	0.292									
rs7208285	A	0.49	1.0909	0.8499	0.199									
rs7208391	C	0.43	0.7106	0.8088	0.380									
rs7208422	A	0.40	0.1715	0.8785	0.845									
rs7208502	A	0.16	-0.0646	1.0668	0.952									
rs7208536	A	0.14	0.4298	1.1272	0.703									
rs7208831	A	0.14	0.4298	1.1272	0.703									
rs7208892	A	0.46	0.4308	0.781	0.581									
rs7209183	C	0.39	-0.6159	0.8776	0.483									
rs7209235	A	0.76	-1.0168	0.9516	0.285									
rs7209293	A	0.21	0.382	0.9379	0.684	0.23	0.6932	0.5416	0.201	0.22	0.6412	0.4825	0.184	
rs7209406	A	0.15	0.5006	1.3378	0.708									
rs7209428	A	0.18	1.9769	1.0242	0.054	0.15	-0.5552	0.6618	0.402	0.15	0.2342	0.5789	0.686	
rs7209474	A	0.78	-1.2771	0.9457	0.177									
rs7209618	A	0.59	0.0952	0.8329	0.909	0.54	-0.0553	0.4563	0.904	0.55	0.1367	0.4093	0.738	0.55
rs7209710	A	0.89	-0.9202	1.4321	0.521	0.88	-0.5299	0.6949	0.446	0.88	-0.5694	0.6231	0.361	
rs7210122	A	0.24	0.0965	1.0763	0.929									
rs7210391	A	0.11	-1.4279	1.1668	0.221	0.13	0.0794	0.7075	0.911	0.12	-0.4575	0.6297	0.468	
rs7210539	A	0.27	0.656	0.9117	0.472	0.26	0.3838	0.5279	0.467	0.26	0.5455	0.4638	0.240	
rs7210574	A	0.56	-0.0933	0.83	0.911									
rs7210702	A	0.52	0.0599	0.8196	0.942	0.55	-0.5713	0.4704	0.225	0.54	-0.4304	0.4153	0.300	

rs7210742	A	0.40	-1.0297	0.8456	0.223									
rs7210946	A	0.47	-0.6093	0.8102	0.452	0.43	0.0599	0.4754	0.900	0.44	0.0012	0.4179	0.998	
rs7210947	A	0.51	0.6071	0.7563	0.422	0.48	-0.2087	0.4682	0.656	0.49	0.082	0.413	0.843	
rs7210951	A	0.75	2.717	0.937	0.004									
rs7211021	A	0.13	1.8424	1.1662	0.114	0.12	-1.5746	0.7048	0.025	0.12	-1.0474	0.6433	0.104	
rs7211095	A	0.16	-0.0646	1.0668	0.952									
rs7211532	A	0.60	0.0913	0.8165	0.911	0.58	0.0762	0.4811	0.874	0.58	0.3289	0.4225	0.436	0.60
rs7211674	A	0.54	-0.4361	0.9165	0.634									
rs7211773	A	0.43	-0.3494	0.8348	0.676									
rs7211818	A	0.93	0.5663	1.6767	0.736									
rs7211960	A	0.12	0.6925	1.6145	0.668									
rs7211963	A	0.17	0.5273	1.1081	0.634									
rs7211994	A	0.39	0.4102	0.8831	0.642									
rs7212069	C	0.90	-0.1397	2.0488	0.946									
rs7212142	A	0.13	2.0046	1.2175	0.100									
rs7212201	A	0.65	1.5354	0.8341	0.066									
rs7212305	A	0.21	0.3026	0.937	0.747	0.23	0.6405	0.5405	0.236	0.22	0.5796	0.4817	0.229	
rs7212486	A	0.68	-3.3047	0.7922	0.000	0.67	0.0568	0.4839	0.907	0.67	-0.534	0.4338	0.218	
rs7212620	A	0.32	0.2059	0.8865	0.816									
rs7212662	A	0.50	-0.602	0.8842	0.496									
rs7212688	A	0.78	0.2452	0.9822	0.803	0.77	0.6384	0.5377	0.235	0.77	0.6544	0.4775	0.171	
rs7212823	A	0.18	0.2279	1.0984	0.836									
rs7213204	A	0.54	-0.7081	0.8324	0.395									
rs7213410	A					0.94	0.2291	1.1191	0.838	0.95	0.9629	1.0478	0.358	
rs7213638	A	0.14	0.4298	1.1272	0.703									
rs7213696	A	0.75	0.2729	0.9156	0.766									
rs7213735	A	0.40	-1.2489	0.7595	0.100	0.35	0.0512	0.4699	0.913	0.37	-0.2561	0.4148	0.537	
rs7213859	A	0.05	0.2133	2.8149	0.940	0.05	0.2707	1.1225	0.809	0.05	0.1904	1.029	0.853	
rs7214286	A	0.21	-0.0937	1.019	0.927									
rs7214678	A	0.79	-0.728	1.0035	0.468									
rs7215260	A	0.51	-1.3408	0.8336	0.108									
rs7215451	A	0.38	0.434	0.8339	0.603									
rs7215470	A	0.66	-0.4727	0.7852	0.547	0.74	-0.2471	0.5095	0.628	0.72	-0.371	0.4433	0.403	
rs7215498	A	0.73	-0.6743	0.9083	0.458	0.81	0.2435	0.5898	0.680	0.80	-0.2158	0.5032	0.668	

rs7215534	A	0.15	0.7193	1.259	0.568									
rs7215669	A	0.68	0.7428	0.8977	0.408									
rs7215765	A	0.60	-1.4858	0.8001	0.063	0.62	0.1327	0.4739	0.780	0.62	-0.2552	0.417	0.541	
rs7215994	A	0.77	1.8813	0.9907	0.058									
rs7216240	A					0.96	-1.6875	1.3232	0.202	0.96	-1.2301	1.1467	0.283	
rs7216559	A	0.06	0.6308	2.4796	0.799									
rs7216577	A	0.42	-0.5687	0.8557	0.506									
rs7216635	A	0.64	-0.084	0.8349	0.920	0.61	-0.1183	0.4565	0.796	0.62	0.0055	0.4092	0.989	0.63
rs7216806	A	0.83	-1.372	1.2025	0.254	0.80	-0.7431	0.5905	0.208	0.81	-0.6577	0.5299	0.215	
rs7217172	A	0.95	-1.2042	2.7049	0.656	0.92	-0.4366	0.9315	0.639	0.93	-0.0587	0.8333	0.944	0.92
rs7217223	A	0.09	1.9964	1.5437	0.196									
rs7217395	A	0.36	-0.5993	0.8115	0.460									
rs7217421	A	0.53	0.4448	0.8139	0.585									
rs7217702	C	0.83	0.4976	1.0414	0.633									
rs7217721	C					0.02	-2.6927	2.0204	0.183	0.02	-2.5076	1.8134	0.167	
rs7217829	A	0.30	-2.6375	0.7968	0.001	0.32	0.4323	0.4803	0.368	0.31	-0.187	0.4287	0.663	
rs7218122	A	0.54	-0.8214	0.8122	0.312									
rs7218261	A	0.15	0.066	1.0867	0.952	0.17	-0.2168	0.6199	0.727	0.16	-0.0742	0.5597	0.895	
rs7218347	A	0.90	-0.1232	1.4809	0.934									
rs7218498	C	0.55	0.1406	0.799	0.860									
rs7218605	A	0.44	1.1232	0.8224	0.172									
rs7218729	C	0.39	0.2415	0.835	0.772									
rs7218755	A					0.01	1.6717	3.2475	0.607	0.01	1.8673	3.2243	0.563	
rs7218918	A	0.19	2.0623	0.9914	0.038	0.18	-0.5997	0.5798	0.301	0.18	0.0639	0.5244	0.903	
rs7218924	A	0.80	0.6525	1.0084	0.518									
rs7219247	A	0.19	-0.8052	1.089	0.460									
rs7219316	A	0.73	-0.2554	0.931	0.784									
rs7219318	A	0.40	-1.1793	0.8394	0.160									
rs7219370	C	0.75	-0.2824	0.9368	0.763									
rs7219382	A	0.74	-0.2697	0.9295	0.772									
rs7219452	A	0.89	-0.4047	1.8402	0.826	0.90	0.1098	0.8494	0.897	0.90	0.2094	0.7527	0.781	0.90
rs7219493	A	0.87	-2.1271	1.3756	0.122									
rs7220261	C	0.08	-0.8407	1.5354	0.584									
rs7220294	A	0.92	0.1619	1.4974	0.914									

rs7220465	A	0.34	-0.7498	0.8525	0.379									
rs7220493	A	0.27	0.3999	0.855	0.640	0.26	0.558	0.5258	0.289	0.26	0.5616	0.4596	0.222	
rs7220592	C	0.60	0.6887	0.7754	0.374	0.58	0.5245	0.4675	0.262	0.58	0.5081	0.4153	0.221	
rs7220955	A	0.48	-1.4459	0.8565	0.091									
rs7220985	C	0.38	-1.1349	0.726	0.118	0.37	0.6935	0.4712	0.141	0.37	0.3783	0.415	0.362	
rs7221014	A	0.82	0.4321	1.048	0.680									
rs7221291	A	0.71	1.2917	0.869	0.137									
rs7221314	A	0.60	0.4968	0.8212	0.545									
rs7221365	A	0.66	0.6434	0.9061	0.478									
rs7221604	A	0.42	0.5321	0.7237	0.462	0.37	-0.8708	0.4459	0.051	0.38	-0.5363	0.4005	0.181	
rs7221608	C	0.90	-3.1031	1.3834	0.025	0.87	0.1535	0.7243	0.832	0.88	-0.5111	0.6388	0.424	0.88
rs7221610	A	0.86	0.4298	1.1272	0.703									
rs7221665	A	0.79	0.0088	1.2766	0.995									
rs7221823	A	0.91	1.9497	1.5396	0.205									
rs7221948	A	0.90	0.7693	1.3835	0.578									
rs7222014	A	0.22	1.6597	0.9349	0.076									
rs7222024	A	0.43	-0.2884	0.8299	0.728									
rs7222366	A	0.75	2.5033	0.9429	0.008									
rs7222531	A	0.74	-0.1587	0.9526	0.868	0.67	0.2189	0.4801	0.648	0.68	0.1329	0.4344	0.760	
rs7222861	A	0.41	0.8809	0.8258	0.286	0.47	-0.0944	0.4545	0.836	0.46	0.1124	0.4058	0.782	
rs7222924	A	0.32	-1.4266	0.9536	0.135									
rs7223219	A	0.83	-1.6488	1.0956	0.132									
rs7223264	A	0.22	0.5987	0.9353	0.522	0.19	0.4587	0.5753	0.425	0.20	0.6994	0.4995	0.161	
rs7223304	A	0.77	-1.1933	0.9953	0.231									
rs7223613	A	0.23	-0.0305	1.0472	0.977									
rs7223756	A	0.49	0.7804	0.7551	0.301	0.44	-0.3295	0.455	0.469	0.45	-0.2153	0.4015	0.592	
rs7223939	A	0.74	-0.3545	0.9405	0.706									
rs7223992	A	0.57	0.1036	0.8401	0.902									
rs7224239	A	0.85	-0.0253	1.1948	0.983									
rs7224615	A	0.24	0.8123	0.9753	0.405									
rs7224668	A	0.66	0.1526	0.8457	0.857									
rs7224711	A	0.45	0.6136	0.8669	0.479									
rs7224728	A	0.92	-0.441	1.4705	0.764	0.94	-0.6724	0.9678	0.487	0.94	-0.2002	0.845	0.813	
rs7225131	A	0.33	0.9106	0.8427	0.280									

rs7225323	A	0.06	1.714	2.8871	0.553									
rs7225364	A	0.57	0.0207	0.7465	0.978	0.53	-0.0098	0.4588	0.983	0.55	0.0379	0.4028	0.925	0.62
rs7225574	A	0.17	-0.4378	1.0877	0.687									
rs7225592	A	0.51	-1.2573	0.8235	0.127									
rs7225655	A	0.55	-0.1072	0.7874	0.892	0.53	0.168	0.4591	0.714	0.54	0.0862	0.4136	0.835	
rs7225663	A	0.72	1.4423	0.93	0.121	0.69	-0.192	0.5131	0.708	0.72	0.2942	0.4728	0.534	0.73
rs7226135	A	0.15	-0.6091	1.2218	0.618									
rs7226158	A	0.24	1.2576	0.9733	0.196	0.26	0.2402	0.5382	0.655	0.25	0.6196	0.4966	0.212	0.24
rs72849330	C	0.81	0.3655	0.975	0.708	0.80	0.418	0.5814	0.472	0.80	0.4073	0.5175	0.431	
rs72852409	A	0.19	-0.1472	1.0234	0.886	0.20	0.8235	0.5801	0.156	0.19	0.5583	0.5254	0.288	
rs72853543	A	0.16	0.3128	1.0806	0.772	0.16	-0.4712	0.6097	0.440	0.16	-0.1009	0.5455	0.853	
rs729996	A	0.21	0.9872	0.9702	0.309									
rs73412102	A					0.98	-0.3192	2.22	0.886	0.98	-0.9116	2.1172	0.667	
rs7342	C	0.38	0.576	0.8423	0.494									
rs7342880	A	0.04	1.3396	2.2329	0.549									
rs734338	A	0.13	1.4744	1.1655	0.206									
rs7350896	A	0.55	0.0668	0.7719	0.931	0.52	0.0569	0.4537	0.900	0.54	0.0376	0.4088	0.927	
rs736100	A	0.17	0.771	1.1019	0.484									
rs736523	A	0.71	-0.3957	0.9151	0.666									
rs7370	A	0.36	-0.4833	0.841	0.566									
rs73999901	A					0.04	0.7808	1.3053	0.550	0.03	1.2653	1.1937	0.289	
rs74006007	A					0.01	-3.3225	3.158	0.293	0.01	-3.1088	3.0075	0.301	
rs7405450	A	0.53	1.329	0.7912	0.093									
rs7405469	C	0.42	1.4025	0.8081	0.083									
rs7405640	A	0.65	-0.2456	0.9578	0.798									
rs7405901	A	0.24	0.0652	1.0444	0.950									
rs7405966	A	0.42	1.4025	0.8081	0.083									
rs7406026	A	0.67	0.2434	0.824	0.768									
rs7406991	C	0.58	1.4025	0.8081	0.083									
rs7409	A	0.44	1.1585	0.8125	0.154									
rs7420	A	0.63	0.1003	0.8453	0.906									
rs745318	A	0.40	-1.0664	0.8556	0.213									
rs745666	C	0.39	0.476	0.8154	0.559									
rs746405	A	0.71	2.1373	0.9059	0.018									

rs747321	A	0.07	0.3447	1.6852	0.838									
rs747742	A	0.10	0.6245	1.4334	0.663									
rs7501499	A	0.12	-0.5583	1.3905	0.688	0.14	0.8919	0.6665	0.181	0.14	0.6747	0.5991	0.260	0.15
rs7501644	A	0.61	-0.3786	0.8549	0.658									
rs7501740	A	0.77	1.6521	0.9596	0.085									
rs7501761	A	0.28	0.1374	0.9327	0.883									
rs7502620	A	0.35	-0.7846	0.9369	0.402									
rs7502835	A	0.32	-2.6028	0.8971	0.004									
rs7503034	A	0.55	-0.2515	0.853	0.768									
rs7503237	A	0.60	-1.2685	0.84	0.131									
rs7503779	A	0.32	0.8749	0.9031	0.333									
rs7503819	A	0.51	-0.2268	0.9077	0.803									
rs7503865	A	0.44	-0.3601	0.7888	0.648	0.43	-0.7718	0.4461	0.084	0.43	-0.5899	0.3944	0.135	
rs7503911	A	0.14	0.2529	1.439	0.861									
rs750844	A	0.47	0.1627	0.8037	0.840									
rs751848	A	0.29	0.6219	0.8763	0.478	0.29	0.3079	0.4855	0.526	0.29	0.3338	0.438	0.446	
rs753403	A	0.23	0.9912	0.9276	0.285									
rs754708	A	0.34	0.0545	0.8677	0.950									
rs755340	A	0.41	0.7105	0.8108	0.381									
rs75560495	A	0.93	-0.1603	2.0967	0.939	0.94	-2.4612	1.1522	0.033	0.94	-1.7584	1.0519	0.095	
rs756075	A	0.16	-0.0646	1.0668	0.952									
rs7591163	A	0.73	0.6939	0.9458	0.463	0.69	-0.035	0.5179	0.946	0.70	0.085	0.4663	0.855	
rs76290800	A	0.50	0.8388	0.761	0.270	0.48	-0.3379	0.4534	0.456	0.47	0.0096	0.4076	0.981	
rs76299544	A	0.10	-2.1133	1.2474	0.090	0.12	-0.1718	0.7356	0.815	0.12	-0.6978	0.6533	0.286	
rs7651	A	0.88	0.6375	1.3686	0.641									
rs76524459	A	0.93	-1.6643	1.7143	0.332	0.93	-0.9458	1.0702	0.377	0.93	-1.0885	0.9739	0.264	
rs771722	A	0.18	-0.327	1.0012	0.744									
rs783230	A	0.57	0.655	0.8327	0.432									
rs783239	A	0.09	-0.3984	1.7633	0.821									
rs783241	A	0.39	0.6019	0.8382	0.473									
rs783250	A	0.57	0.7271	0.8257	0.379									
rs79087642	A	0.11	0.7513	1.4049	0.593	0.12	-0.2631	0.746	0.724	0.11	0.1456	0.6908	0.833	
rs80198744	A	0.06	0.0942	2.0536	0.963	0.07	-0.9492	0.9457	0.316	0.07	-0.6454	0.8669	0.457	
rs8064345	A	0.04	0.7573	2.8892	0.793									

rs8064357	A	0.33	0.9172	0.8204	0.264	0.37	0.0769	0.4966	0.877	0.36	0.4138	0.4399	0.347	
rs8064529	A	0.29	0.9196	0.9123	0.313									
rs8064769	A	0.21	0.3026	0.937	0.747	0.23	0.6345	0.5411	0.241	0.22	0.5775	0.4818	0.231	
rs8065144	A	0.59	1.024	0.8106	0.207									
rs8065364	A	0.85	0.4635	1.1337	0.683									
rs8065422	A	0.85	-0.3168	1.1766	0.788									
rs8065431	A	0.67	1.3014	0.8099	0.108	0.66	-0.4039	0.4673	0.387	0.66	0.0273	0.4242	0.949	
rs8065523	A	0.05	0.7097	2.0885	0.734									
rs8065598	A	0.07	0.5663	1.6767	0.736									
rs8065843	A	0.62	0.3722	0.8331	0.655									
rs8066695	A	0.54	-0.0514	0.8402	0.951									
rs8066768	A					0.05	-0.2084	1.1343	0.854	0.04	0.1212	1.0337	0.907	0.04
rs8066857	A	0.08	1.616	1.6679	0.333	0.12	-0.7104	0.7204	0.324	0.11	-0.0852	0.6679	0.899	
rs8066867	A	0.08	0.7398	1.6521	0.654									
rs8066993	A	0.28	-0.8195	1.0042	0.415									
rs8067167	A	0.38	1.126	0.8095	0.164	0.45	-0.1162	0.4711	0.805	0.43	0.2258	0.4189	0.590	
rs8067235	A	0.21	-0.5815	0.9951	0.559									
rs8067292	A	0.76	1.3387	0.882	0.129									
rs8067409	A					0.02	-1.7037	2.4631	0.489	0.02	-1.563	2.2522	0.488	
rs8067574	A	0.30	1.4301	0.8623	0.097									
rs8067885	A	0.76	-0.0061	0.8744	0.995	0.78	0.3009	0.5471	0.582	0.77	0.4413	0.4771	0.355	
rs8067947	A	0.52	0.2748	0.8068	0.733									
rs8067984	A	0.26	0.7856	0.9218	0.394									
rs8068064	A	0.19	0.7886	1.0142	0.437									
rs8068137	A	0.34	0.1106	0.888	0.901									
rs8068511	A	0.29	0.2114	0.8501	0.804									
rs8068514	A	0.94	1.7403	2.0288	0.391									
rs8068637	A	0.12	-0.004	1.3384	0.998									
rs8068796	A	0.38	-0.8974	0.8004	0.262									
rs8068871	A	0.29	0.0169	0.8986	0.985	0.23	0.4561	0.5418	0.400	0.24	0.4329	0.4724	0.359	0.25
rs8069348	C	0.10	-0.1232	1.4809	0.934									
rs8069502	A	0.23	-0.5761	0.9118	0.528	0.20	0.2823	0.5766	0.624	0.21	-0.0292	0.5048	0.954	
rs8069521	A	0.05	2.653	2.5516	0.299									
rs8070106	A	0.28	1.1939	0.8789	0.174									

rs8070127	A	0.18	-1.6305	1.6593	0.326									
rs8070274	A	0.97	0.1719	3.287	0.958	0.96	0.5691	1.1974	0.635	0.96	0.7265	1.1092	0.513	
rs8070383	A					0.02	-2.5558	1.9755	0.196	0.02	-1.9051	1.7677	0.281	
rs8070406	A					0.97	-2.1986	1.5751	0.163	0.97	-1.1695	1.4915	0.433	0.98
rs8070488	A	0.88	1.5099	1.3208	0.253									
rs8070953	A	0.74	0.5092	0.9109	0.576									
rs8070973	A	0.50	0.305	0.7395	0.680	0.50	-0.111	0.4539	0.807	0.50	0.0122	0.4042	0.976	0.51
rs8071004	A	0.96	-0.3847	2.7847	0.890	0.92	-0.4816	0.9639	0.617	0.93	-0.1184	0.8757	0.892	0.93
rs8071015	A	0.92	0.9241	1.6355	0.572									
rs8071198	A	0.68	-0.0943	0.9352	0.920									
rs8071668	A	0.25	0.1529	1.109	0.890									
rs8071693	A	0.06	2.6766	2.1947	0.223	0.05	-2.675	1.2528	0.033	0.04	-1.8337	1.078	0.089	0.05
rs8071885	C	0.62	-1.409	0.8163	0.084									
rs8071975	A	0.55	0.8429	0.7992	0.292									
rs8072027	A					0.98	-1.4438	2.8465	0.612	0.99	-1.3395	2.8783	0.642	
rs8072277	A	0.18	-0.5425	1.116	0.627									
rs8072347	A	0.43	-1.4328	0.7494	0.056	0.46	0.3527	0.4513	0.434	0.46	-0.0556	0.4013	0.890	
rs8072592	A	0.22	1.2663	0.9713	0.192									
rs8073077	A	0.21	-1.532	0.9837	0.119	0.21	-0.7914	0.6002	0.187	0.21	-0.7408	0.5332	0.165	0.14
rs8073182	A	0.58	-0.1667	0.9076	0.854									
rs8073197	A	0.92	-3.5722	1.5475	0.021	0.93	-2.2038	0.8819	0.012	0.93	-2.4034	0.7659	0.002	
rs8073414	A	0.20	0.695	1.0129	0.493									
rs8073529	A	0.26	-0.0064	0.9963	0.995	0.23	0.3943	0.552	0.475	0.23	0.4126	0.4826	0.393	
rs8073550	A	0.40	-0.1979	0.7471	0.791	0.38	-0.0116	0.464	0.980	0.39	-0.066	0.4103	0.872	
rs8073615	A	0.33	0.661	0.8982	0.462									
rs8073660	A	0.72	-0.5522	0.8985	0.539									
rs8073763	A	0.23	0.6091	0.995	0.540									
rs8073791	A					0.05	-0.2106	1.1347	0.853	0.04	0.0676	1.0302	0.948	0.04
rs8074418	A	0.78	0.2701	0.8794	0.759	0.78	0.0692	0.5343	0.897	0.78	0.3521	0.4716	0.455	
rs8074685	A	0.59	-0.3884	0.81	0.632									
rs8074821	A	0.67	0.9106	0.8427	0.280									
rs8074842	C	0.27	0.6948	0.9064	0.443									
rs8075102	A	0.42	1.4025	0.8081	0.083									
rs8075298	A	0.48	-1.3667	0.8123	0.092									

rs8075300	A	0.23	0.2766	0.9331	0.767	0.27	-0.2268	0.5048	0.653	0.27	0.152	0.4559	0.739	
rs8075376	A	0.25	0.4974	0.8751	0.570	0.25	0.6542	0.5349	0.221	0.25	0.6416	0.4685	0.171	
rs8075628	A	0.15	1.0657	1.1346	0.348									
rs8075657	A	0.29	-0.2384	0.8256	0.773	0.27	-0.5032	0.5366	0.348	0.28	-0.4218	0.4677	0.367	
rs8075839	A	0.81	1.7269	1.0756	0.108									
rs8075897	A	0.22	-1.0567	0.9625	0.272									
rs8076283	A	0.60	-0.4627	0.7462	0.535	0.61	0.0044	0.4619	0.992	0.61	-0.0984	0.4092	0.810	
rs8076334	A	0.21	-0.3472	0.906	0.702	0.23	-0.1275	0.525	0.808	0.23	-0.0357	0.473	0.940	
rs8076416	A	0.17	-0.3283	1.2777	0.797	0.18	-0.4805	0.6172	0.436	0.18	-0.3844	0.5642	0.496	0.16
rs8076595	A	0.70	0.3676	0.8936	0.681	0.73	-0.419	0.5275	0.427	0.72	-0.3137	0.4613	0.497	
rs8077024	A	0.49	-1.2043	0.7713	0.118									
rs8077046	A	0.33	0.0492	0.8824	0.956									
rs8077205	A	0.24	-0.832	0.9332	0.373	0.24	0.0964	0.5393	0.858	0.24	-0.0979	0.4751	0.837	
rs8077819	A	0.59	-1.3925	0.8552	0.104									
rs8077901	A	0.14	0.4298	1.1272	0.703									
rs8077911	C	0.29	-0.9833	0.8882	0.268	0.31	-0.1925	0.487	0.693	0.30	-0.2842	0.4346	0.513	
rs8078251	A	0.26	0.9937	0.8701	0.253									
rs8078334	A	0.69	-0.8404	0.8032	0.295	0.69	0.2742	0.5076	0.589	0.69	-0.1622	0.44	0.712	
rs8078532	A	0.37	-1.1692	0.8372	0.163									
rs8078577	A	0.38	-0.7933	0.847	0.349									
rs8078643	A	0.54	-0.8505	0.8138	0.296									
rs8078752	A	0.41	0.3159	0.7606	0.678	0.42	0.1472	0.4506	0.744	0.42	-0.0757	0.4019	0.851	
rs8078791	A	0.13	0.9	1.4504	0.535	0.17	-0.1288	0.6251	0.837	0.16	0.0153	0.5806	0.979	
rs8078851	A	0.24	1.1325	0.8953	0.206									
rs8078855	A	0.39	1.6375	1.1092	0.140									
rs8079156	A	0.81	0.7938	1.0243	0.438									
rs8079383	A	0.12	0.4096	1.3989	0.770	0.15	-0.3354	0.6759	0.620	0.14	-0.0678	0.6124	0.912	0.09
rs8079537	A	0.86	0.8599	1.2581	0.494									
rs8079626	A	0.67	0.5233	0.849	0.538									
rs8079646	A	0.23	-1.5226	0.8505	0.073	0.16	0.438	0.6102	0.473	0.18	-0.0651	0.5179	0.900	
rs8079757	A	0.84	0.6362	1.3159	0.629	0.80	0.528	0.6741	0.434	0.81	0.5316	0.5924	0.370	
rs8079781	A	0.46	-0.8351	0.8097	0.302									
rs8080101	A	0.55	-0.5799	0.7676	0.450	0.54	-0.1205	0.4584	0.793	0.55	-0.2568	0.409	0.530	
rs8080155	A	0.69	-1.0231	0.8084	0.206	0.69	0.2927	0.5069	0.564	0.69	-0.2047	0.4402	0.642	

rs8080265	A	0.07	-0.7874	2.3185	0.734									
rs8080597	A	0.08	0.5972	1.5707	0.704	0.09	-0.4751	0.8787	0.589	0.08	-0.4911	0.8122	0.545	
rs8081143	A	0.58	-0.2682	0.7823	0.732	0.54	0.2879	0.4605	0.532	0.55	0.4045	0.4088	0.322	
rs8081168	A	0.80	1.9653	1.0169	0.053									
rs8081176	A	0.70	-0.117	0.9452	0.902									
rs8081466	A	0.79	0.4168	0.9351	0.656	0.77	0.7217	0.5357	0.178	0.78	0.6629	0.4792	0.167	
rs8081669	A	0.35	1.335	0.8564	0.119									
rs8081783	A	0.78	-1.091	0.9335	0.243									
rs8081928	C	0.25	0.8897	0.9323	0.340									
rs8082005	A	0.36	-1.3481	0.8609	0.117									
rs8082181	A					0.05	-1.8103	1.1523	0.116	0.05	-0.4004	1.0811	0.711	
rs8082252	A	0.43	0.7977	0.8107	0.325									
rs8082289	A	0.22	0.1773	0.985	0.857	0.23	0.628	0.5452	0.249	0.22	0.6349	0.4836	0.189	
rs8082303	A	0.78	1.2663	0.9713	0.192									
rs8082640	A	0.42	-0.2946	0.7896	0.709	0.46	0.3092	0.4619	0.503	0.44	0.4043	0.4107	0.325	0.43
rs809740	A	0.57	0.6435	0.8304	0.438									
rs8150	C	0.31	1.2067	0.9156	0.188									
rs820129	A	0.62	0.0582	0.8664	0.946									
rs820134	A	0.11	0.4434	1.6721	0.791									
rs820145	A	0.11	1.0241	1.7837	0.566									
rs820146	A	0.11	0.5356	1.7009	0.753									
rs820152	A	0.81	1.2372	1.0587	0.243									
rs820155	C	0.38	0.0708	0.8686	0.935									
rs820157	A	0.89	0.5356	1.7009	0.753									
rs820184	A	0.81	1.2607	1.027	0.220									
rs820186	A	0.11	-0.0751	1.7589	0.966									
rs820190	A	0.11	0.5356	1.7009	0.753									
rs820196	A	0.65	-0.18	0.8838	0.839									
rs820201	A	0.38	-0.1699	0.8652	0.844									
rs820202	A	0.38	-0.1699	0.8652	0.844									
rs820210	A	0.62	-0.1699	0.8652	0.844									
rs820216	A	0.92	3.1876	1.9259	0.098									
rs820233	A	0.50	1.0546	0.8347	0.206									
rs820242	A	0.89	0.0308	1.438	0.983									

rs820264	A	0.10	-0.6789	1.3432	0.613									
rs866414	A	0.22	-1.8041	1.0985	0.101	0.22	0.2685	0.5726	0.639	0.22	-0.0549	0.509	0.914	
rs868432	A	0.53	0.261	0.8101	0.747									
rs870355	A	0.27	-0.3336	0.9994	0.739									
rs871443	A	0.31	0.8813	0.8971	0.326									
rs871741	A	0.40	-1.1014	0.7553	0.145	0.35	0.1502	0.4705	0.750	0.37	-0.1421	0.4149	0.732	
rs872640	A	0.81	-0.084	1.1033	0.939									
rs8746	A	0.13	4.1161	1.247	0.001									
rs878906	A	0.36	0.3658	0.8376	0.662									
rs880827	A	0.36	0.3658	0.8376	0.662									
rs881502	A	0.57	-0.9753	0.8442	0.248									
rs8836	C	0.55	0.865	0.8007	0.280									
rs883890	C					0.05	-1.856	1.1477	0.106	0.05	-0.4551	1.0768	0.673	
rs884204	A	0.79	1.1784	0.9815	0.230									
rs884446	A	0.23	-1.6139	0.8363	0.054	0.17	1.1543	0.6064	0.057	0.18	0.4193	0.5164	0.417	
rs884652	A	0.12	0.5576	1.2027	0.643	0.13	-0.111	0.6643	0.867	0.13	0.0452	0.5973	0.940	0.14
rs892960	A	0.45	-0.4094	0.8824	0.643									
rs894310	A	0.13	1.6533	1.288	0.199	0.10	-1.3118	0.8562	0.126	0.09	-0.4535	0.7558	0.548	
rs894545	A	0.80	-1.6987	1.0212	0.096									
rs894546	A	0.14	-2.519	1.2279	0.040									
rs894939	A	0.28	-0.9752	0.9564	0.308									
rs894945	A	0.26	0.7126	0.9123	0.435									
rs894949	A	0.33	-0.7703	1.1733	0.512									
rs894956	C	0.85	-0.6923	1.2834	0.590									
rs897587	A	0.22	1.5886	1.0142	0.117	0.24	0.2713	0.5484	0.621	0.22	0.5851	0.5097	0.251	
rs897588	A	0.28	-0.9265	0.9206	0.314	0.27	0.486	0.5014	0.332	0.27	0.1519	0.4452	0.733	
rs897593	A	0.62	-0.1631	0.8386	0.846	0.57	0.0029	0.4591	0.995	0.58	-0.1586	0.4119	0.700	
rs897594	A	0.30	0.4635	1.1817	0.695									
rs897595	A	0.41	-0.6782	0.7981	0.395	0.36	0.0602	0.4787	0.900	0.37	-0.1814	0.4265	0.671	
rs897597	C	0.63	-0.2931	0.8307	0.724	0.62	0.2691	0.4994	0.590	0.62	-0.0171	0.4408	0.969	0.57
rs897600	A	0.25	-1.6656	0.8248	0.043	0.19	0.5866	0.5937	0.323	0.21	0.0544	0.5041	0.914	
rs898085	A	0.66	-0.7902	0.8609	0.359									
rs898525	A	0.41	0.7194	0.7509	0.338	0.39	-0.367	0.4615	0.426	0.39	-0.016	0.409	0.969	
rs898533	A	0.78	-1.2612	0.9128	0.167	0.79	0.6286	0.5524	0.255	0.79	0.125	0.484	0.796	

rs898534	A	0.22	-1.4062	0.9056	0.121	0.20	0.5646	0.558	0.312	0.21	0.0316	0.4868	0.948	
rs898537	A	0.43	0.4541	0.8138	0.577									
rs899317	A	0.49	0.7428	0.851	0.383									
rs901065	A	0.13	0.3677	1.3517	0.786									
rs902731	A	0.54	0.8987	0.7845	0.252									
rs9038	A	0.18	0.7445	1.1812	0.529									
rs907898	A	0.73	-0.0738	1.0174	0.942	0.77	0.5256	0.5675	0.354	0.76	0.5157	0.4967	0.299	
rs907899	A	0.56	0.4557	0.7275	0.531	0.54	0.2197	0.4707	0.641	0.55	0.1215	0.4141	0.769	
rs907901	A	0.03	0.1719	3.287	0.958	0.04	0.5363	1.2097	0.658	0.04	0.7154	1.1185	0.522	
rs907910	A	0.17	0.445	1.2428	0.720									
rs907911	A	0.22	-1.9259	0.8486	0.023	0.17	0.5342	0.6022	0.375	0.18	-0.08	0.5169	0.877	
rs907915	A	0.73	0.4009	0.8567	0.640	0.74	0.6412	0.5288	0.225	0.74	0.599	0.462	0.195	
rs908241	A	0.55	-0.927	0.8119	0.254									
rs910557	A	0.16	-0.2534	1.2806	0.843									
rs920131	A	0.23	-0.8721	0.9973	0.382									
rs922838	C	0.44	0.6208	0.8202	0.449									
rs925607	A	0.83	-1.0019	1.1292	0.375	0.87	-1.0385	0.6969	0.136	0.87	-0.4513	0.6389	0.480	0.90
rs9302890	A	0.38	-1.1016	0.8169	0.178	0.37	0.0982	0.4749	0.836	0.37	-0.245	0.419	0.559	
rs9302897	A	0.79	1.1571	1.0791	0.284	0.78	0.1757	0.5904	0.766	0.80	0.3429	0.5434	0.528	
rs9302989	A	0.93	-3.0128	1.8154	0.097									
rs9302990	A	0.07	-3.0128	1.8154	0.097									
rs9302994	A	0.45	0.1861	0.7955	0.815									
rs9308945	A	0.78	1.1745	0.9325	0.208	0.72	-0.1907	0.5051	0.706	0.74	0.1718	0.4506	0.703	
rs9319623	C	0.53	-0.5588	0.7316	0.445	0.53	0.6517	0.4393	0.138	0.53	0.3588	0.393	0.361	
rs934668	A	0.57	2.0798	0.8245	0.012	0.62	0.251	0.4711	0.594	0.61	0.5043	0.4156	0.225	
rs935334	A	0.07	-1.7624	2.18	0.419	0.07	-1.002	0.9193	0.276	0.07	-0.8443	0.8423	0.316	
rs936056	A	0.42	-0.0354	0.8321	0.966									
rs936393	A	0.81	-1.0162	1.2075	0.400									
rs938283	A	0.85	0.0254	1.2186	0.983	0.84	0.3095	0.6148	0.615	0.85	0.2345	0.5527	0.671	
rs938348	A	0.62	1.1661	0.8219	0.156									
rs938350	A	0.64	1.6015	0.839	0.056									
rs939540	A	0.26	0.0707	0.9346	0.940									
rs939543	A	0.53	-0.3181	0.8379	0.704									
rs959260	A	0.93	-2.533	1.7971	0.159									

rs9635664	A	0.38	-0.0182	0.7967	0.982	0.41	-0.3876	0.4684	0.408	0.40	-0.3787	0.419	0.366	
rs9660	A	0.87	-2.1271	1.3756	0.122									
rs9674550	A	0.26	0.8597	0.9218	0.351									
rs9674559	A	0.93	0.5663	1.6767	0.736									
rs9675230	A	0.36	0.3176	0.8494	0.709									
rs9675239	A	0.67	-1.3018	0.7871	0.098	0.71	0.2653	0.5043	0.599	0.70	-0.1119	0.4424	0.800	
rs968493	A	0.74	0.5597	0.9708	0.564	0.72	-0.2931	0.5105	0.566	0.73	-0.1059	0.4598	0.818	
rs969413	A	0.28	-1.2229	0.8934	0.171	0.28	-0.0827	0.5334	0.877	0.28	-0.2367	0.4767	0.620	0.23
rs971625	A	0.53	0.3041	0.8486	0.720	0.56	-0.3534	0.4357	0.417	0.56	-0.2081	0.3959	0.599	0.56
rs971626	A	0.48	-0.5336	0.7752	0.491	0.43	-0.5321	0.4347	0.221	0.44	-0.3647	0.3948	0.356	
rs9747668	A	0.10	-1.7062	1.5188	0.261									
rs978415	A	0.24	-0.5376	0.8721	0.538	0.21	0.1126	0.5547	0.839	0.22	-0.0862	0.4863	0.859	
rs9789009	A	0.87	0.2424	1.7509	0.890	0.89	-1.6358	0.7544	0.030	0.89	-1.5447	0.6795	0.023	0.90
rs9807041	A	0.93	0.5663	1.6767	0.736									
rs9815354	A	0.13	-0.4091	1.4366	0.776	0.10	1.6809	0.7554	0.026	0.10	1.1283	0.6705	0.092	
rs981645	C	0.77	-1.0362	0.8641	0.231	0.74	0.3629	0.5203	0.486	0.75	0.0096	0.4612	0.984	
rs9890248	C	0.80	0.6312	1.0339	0.542	0.86	-0.4358	0.6612	0.510	0.84	-0.3659	0.5807	0.529	
rs9890550	A	0.52	0.7102	0.8353	0.395									
rs9890664	A	0.73	0.1971	1.097	0.857									
rs9891076	A	0.45	0.1595	0.8001	0.842									
rs9891160	A	0.07	0.5663	1.6767	0.736									
rs9892163	A	0.65	0.441	0.8793	0.616									
rs9892166	C	0.88	-1.3904	1.2888	0.281									
rs9892257	A	0.06	-1.6482	1.7724	0.352									
rs9892688	C	0.27	-0.3929	0.9273	0.672									
rs9893035	A	0.55	-1.0456	0.8482	0.218									
rs9893556	A	0.58	0.6158	0.807	0.445	0.62	1.0435	0.4552	0.022	0.61	0.6739	0.4064	0.097	0.60
rs9894131	A	0.11	0.5353	1.3733	0.697	0.12	-0.22	0.7622	0.773	0.11	-0.0645	0.6836	0.925	
rs9894139	A	0.48	-1.2854	0.7803	0.100	0.52	-0.68	0.4559	0.136	0.50	-1.0109	0.4052	0.013	0.48
rs9894429	A	0.77	-0.0305	1.0472	0.977									
rs9894736	A	0.13	2.0046	1.2175	0.100									
rs9894899	A	0.76	-0.5376	0.8721	0.538	0.79	0.0883	0.5579	0.874	0.79	-0.1133	0.4882	0.817	
rs9895584	A	0.86	0.4298	1.1272	0.703									
rs9895586	C	0.45	0.1344	0.7567	0.859	0.46	-0.1266	0.4576	0.782	0.46	-0.2101	0.4042	0.603	0.45

rs9895647	A	0.72	-0.0277	0.899	0.975	0.69	-0.0265	0.4907	0.957	0.70	0.1416	0.4372	0.746	
rs9895930	A	0.93	-2.2666	2.3108	0.327									
rs9895947	A	0.54	0.191	0.8025	0.812									
rs9896073	C	0.51	0.5444	0.8381	0.516									
rs9896146	A	0.24	-1.1161	0.9084	0.219	0.26	-0.2903	0.5176	0.575	0.25	-0.3137	0.4691	0.504	
rs9896771	A	0.79	1.4472	0.9816	0.140									
rs9896850	A	0.19	-0.7721	1.0815	0.475	0.21	-0.7062	0.5959	0.236	0.21	-0.717	0.5288	0.175	0.15
rs9897213	A	0.66	1.2831	0.8418	0.127									
rs9897367	C	0.50	1.1293	0.7977	0.157	0.44	-0.4716	0.4586	0.304	0.45	-0.3248	0.4049	0.422	
rs9897410	A	0.49	0.7526	0.8001	0.347									
rs9897453	A	0.22	1.3997	0.9702	0.149									
rs9897730	A	0.23	-0.8483	0.9072	0.350	0.26	0.1426	0.5092	0.779	0.25	-0.197	0.4546	0.665	
rs9897764	A	0.32	-1.1474	0.875	0.190									
rs9897826	A	0.04	4.5634	2.7221	0.094									
rs9897830	A	0.86	0.4298	1.1272	0.703									
rs9897914	A	0.47	0.7137	0.7822	0.362	0.50	0.6726	0.4544	0.139	0.49	0.361	0.3999	0.367	
rs9898046	A	0.25	0.3476	0.8844	0.694	0.24	0.7611	0.5409	0.159	0.25	0.6828	0.4736	0.149	
rs9898178	A	0.84	0.8799	1.1871	0.459									
rs9898301	A	0.16	-1.4371	1.6479	0.383									
rs9898379	C	0.71	-0.3701	0.9287	0.690									
rs9898441	A	0.86	0.4298	1.1272	0.703									
rs9898469	A	0.26	1.5071	0.8715	0.084	0.21	-0.9351	0.5493	0.089	0.23	-0.4336	0.4816	0.368	
rs9898470	A	0.94	-5.4598	3.1076	0.079									
rs9898803	A	0.14	0.8587	1.2732	0.500	0.10	1.2785	0.7669	0.096	0.11	1.1144	0.6572	0.090	
rs9899051	A	0.35	2.4673	0.8174	0.003									
rs9899178	A	0.45	-0.7139	0.8023	0.374									
rs9899531	A	0.06	-0.0036	2.8859	0.999	0.02	-1.4409	3.7733	0.703	0.03	-0.5045	2.3334	0.829	
rs9899673	A	0.28	1.0572	0.937	0.259									
rs9899687	A	0.93	-3.0128	1.8154	0.097									
rs9899843	A	0.07	0.5663	1.6767	0.736									
rs9899862	A	0.11	0.4701	1.4434	0.745									
rs9900002	A	0.92	-3.205	2.1724	0.140									
rs9900586	A	0.09	-2.8252	1.5961	0.077									
rs9900690	A	0.71	-0.423	0.7792	0.587	0.71	0.185	0.505	0.714	0.71	-0.0809	0.4428	0.855	

rs9900972	A	0.17	-1.3448	1.1119	0.227									
rs9901049	A	0.86	0.4298	1.1272	0.703									
rs9901361	A	0.16	-1.2266	1.1085	0.269									
rs9901434	A	0.07	-2.7944	1.8193	0.125									
rs9901514	A	0.74	-0.5847	0.9318	0.530									
rs9901648	A	0.40	-0.9307	0.8216	0.257									
rs9901846	A	0.40	-1.2685	0.84	0.131									
rs9902013	A	0.79	-1.3803	1.1384	0.225									
rs9902358	A	0.27	-0.0431	0.9028	0.962									
rs9902690	A	0.67	0.9109	0.8485	0.283									
rs9902702	A	0.55	-0.0311	0.808	0.969									
rs9902818	A	0.52	-0.6657	0.8346	0.425	0.54	-0.02	0.4598	0.965	0.54	0.1748	0.4087	0.669	
rs9902912	A					0.01	-4.0512	3.1893	0.204	0.01	-0.4453	2.933	0.879	
rs9903640	C	0.33	-0.5513	0.8714	0.527									
rs9903812	A	0.83	-1.0033	1.1632	0.388									
rs9904341	C	0.54	0.7943	0.8204	0.333									
rs9904414	A	0.70	1.4905	0.782	0.057	0.64	-0.7063	0.4771	0.139	0.65	-0.1411	0.4219	0.738	
rs9904630	A	0.74	0.5515	0.9614	0.566	0.73	-0.297	0.5168	0.566	0.74	-0.1025	0.4634	0.825	
rs9904772	A	0.89	-0.6446	1.3225	0.626	0.88	-0.8962	0.7386	0.225	0.88	-0.9066	0.6743	0.179	
rs9904783	A	0.76	0.0608	0.9619	0.950	0.74	0.2848	0.5212	0.585	0.74	0.1151	0.4702	0.807	
rs9904844	A	0.17	2.4196	1.0771	0.025									
rs9904969	C	0.27	1.2882	0.8623	0.135	0.22	-0.8575	0.5313	0.107	0.24	-0.4053	0.4696	0.388	
rs9905663	A	0.47	0.1009	0.8085	0.901									
rs9905685	C	0.46	0.1629	0.7574	0.830	0.53	-0.2479	0.4315	0.566	0.51	-0.085	0.3904	0.828	
rs9905852	A	0.47	-0.6127	0.8311	0.461	0.52	-0.5944	0.4579	0.194	0.51	-0.8563	0.4055	0.035	0.48
rs9905991	A	0.80	-1.6035	1.0722	0.135									
rs9906023	A	0.26	1.5042	0.8707	0.084	0.22	-0.9294	0.5432	0.087	0.23	-0.4209	0.4778	0.378	
rs9906330	A	0.62	-0.7637	0.885	0.388									
rs9906525	A	0.51	-0.7136	0.7735	0.356	0.48	-0.6844	0.4551	0.133	0.49	-0.8652	0.4034	0.032	0.51
rs9906700	A	0.67	1.3012	0.8093	0.108	0.69	-0.1445	0.5004	0.773	0.68	0.21	0.4383	0.632	
rs9906827	A	0.16	-0.15	1.0665	0.888									
rs9907094	A	0.56	-0.3843	0.773	0.619	0.53	-0.2	0.4509	0.657	0.54	-0.0616	0.4035	0.879	0.54
rs9907115	A	0.49	0.9839	0.8275	0.234									
rs9907318	A	0.46	-0.6484	0.8477	0.444	0.44	0.2366	0.4602	0.607	0.45	-0.109	0.4158	0.793	

rs9907519	A	0.68	-0.5834	0.8588	0.497									
rs9907544	A	0.22	-0.1947	0.9805	0.843	0.24	-0.441	0.5555	0.427	0.23	-0.5621	0.5088	0.269	0.21
rs9907837	A	0.47	-0.4797	0.7394	0.517	0.47	0.4903	0.4422	0.268	0.47	0.2624	0.396	0.508	
rs9908454	A	0.60	-0.5742	0.8758	0.512									
rs9908495	A	0.82	-0.335	1.0813	0.757									
rs9909462	A	0.26	-0.3488	1.0322	0.735									
rs9909805	A	0.48	-1.4082	0.815	0.084									
rs9910192	A	0.55	-0.0707	0.8011	0.930	0.53	-0.989	0.4508	0.028	0.53	-0.7038	0.3983	0.077	
rs9910454	C	0.25	0.3723	1.0305	0.718									
rs9910615	A	0.66	0.962	0.837	0.250	0.71	-0.4479	0.5035	0.374	0.69	-0.2653	0.4445	0.551	
rs9910745	A	0.14	0.424	1.294	0.743									
rs9910792	A	0.65	2.2206	0.8484	0.009	0.62	0.3053	0.462	0.509	0.62	0.8616	0.4151	0.038	
rs9911063	A	0.79	-0.7645	1.0024	0.446									
rs9911171	A	0.07	0.5663	1.6767	0.736									
rs9911245	A	0.61	0.0431	0.8074	0.957	0.62	0.3258	0.4628	0.481	0.62	0.2681	0.4157	0.519	0.66
rs9911346	A	0.29	0.3309	0.85	0.697	0.29	0.8079	0.4893	0.099	0.29	0.6541	0.4333	0.131	
rs9911502	C	0.29	0.9196	0.9123	0.313									
rs9911523	A	0.05	0.8396	1.8913	0.657									
rs9911538	A	0.31	-0.9528	0.8097	0.239	0.30	0.2347	0.5106	0.646	0.31	-0.2456	0.4423	0.579	
rs9911832	A	0.34	0.1549	0.8881	0.862									
rs9912051	A	0.92	0.5652	1.6028	0.724									
rs9912092	A	0.25	2.4009	0.9499	0.011									
rs9912608	C	0.91	-2.8252	1.5961	0.077									
rs9913009	A	0.86	0.4298	1.1272	0.703									
rs9913021	A	0.82	-1.1349	1.0685	0.288	0.79	-0.822	0.595	0.167	0.79	-0.8083	0.5287	0.126	
rs9913162	A	0.79	1.1747	0.9798	0.231									
rs9914011	C	0.34	0.8869	0.8212	0.280									
rs9914068	C	0.74	-0.8366	0.9249	0.366	0.82	0.5398	0.6062	0.373	0.81	-0.0433	0.5134	0.933	
rs9914201	A	0.26	-0.2932	1.1058	0.791	0.20	-0.5427	0.5987	0.365	0.21	-0.6568	0.5371	0.221	
rs9914874	A	0.46	0.3987	0.7902	0.614	0.49	-0.067	0.4616	0.885	0.49	0.1251	0.4092	0.760	
rs9915000	A	0.04	5.3173	2.2386	0.018	0.06	1.0851	1.0052	0.280	0.05	1.7343	0.9261	0.061	
rs9915162	A	0.86	0.8599	1.2581	0.494									
rs9916285	A	0.53	-1.1822	0.7685	0.124									
rs9916688	A	0.89	1.003	1.3741	0.465	0.90	-0.6982	0.791	0.377	0.90	-0.3242	0.7264	0.655	

rs9916764	A	0.48	-0.3125	0.8085	0.699								
rs9916886	A	0.55	-0.3803	0.8148	0.641								
rs9944501	A	0.10	0.3266	1.4044	0.816	0.11	-0.8615	0.7509	0.251	0.11	-0.689	0.6935	0.320
rs9988	A	0.43	5.9988	2.2697	0.008								
rs9989484	A	0.86	0.4298	1.1272	0.703								
